# Sulfur(iv)-mediated umpolung α-heterofunctionalization of 2-oxazolines†

**DOI:** 10.1039/d2sc00476c

**Published:** 2022-04-09

**Authors:** Qifeng Zhang, Yuchen Liang, Ruiqi Li, Ziyi Huang, Lichun Kong, Peng Du, Bo Peng

**Affiliations:** Key Laboratory of the Ministry of Education for Advanced Catalysis Materials, Zhejiang Normal University China pengbo@zjnu.cn dupeng@zjnu.edu.cn

## Abstract

The α-umpolung of carbonyl compounds significantly expands the boundaries of traditional carbonyl chemistry. Despite various umpolung methods available today, reversing the inherent reactivity of carbonyls still remains a substantial challenge. In this article, we report the first use of sulfonium salts, *in lieu* of well-established hypervalent iodines, for the carbonyl umpolung event. The protocol enables the incorporation of a wide variety of heteroatom nucleophiles into the α-carbon of 2-oxazolines. The success of this investigation hinges on the following factors: (1) the use of sulfoxides, which are abundant, structurally diverse and tunable, and easily accessible, ensures the identification of a superior oxidant namely phenoxathiin sulfoxide for the umpolung reaction; (2) the “assembly/deprotonation” protocol previously developed for rearrangement reactions in our laboratory was successfully applied here for the construction of α-umpoled 2-oxazolines.

## Abstract 2

Aryl sulfonium salts serving as a new type of carbonyl umpolung reagent enable direct α-heterofunctionalization of 2-oxazolines.

## Graphical abstract

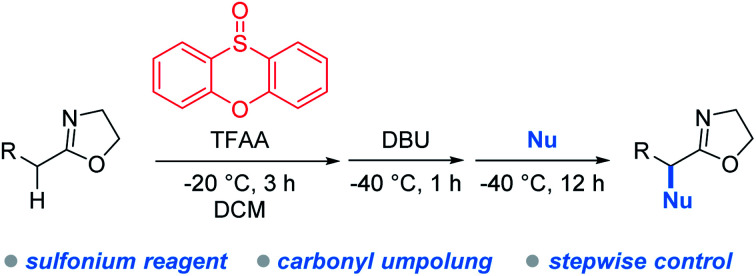

## Introduction

**Fig. 1:**
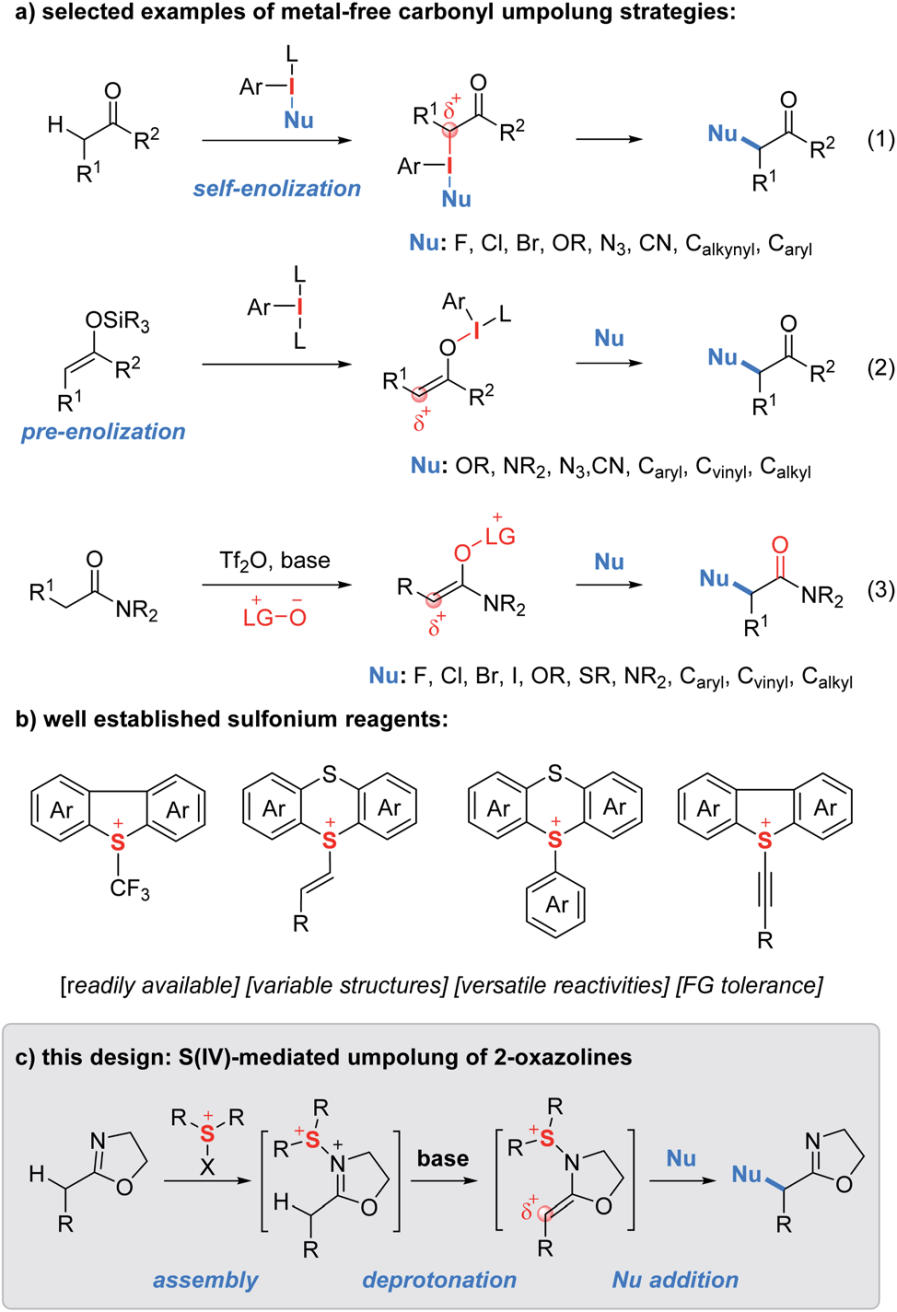
Background and this design.

The α-functionalization of carbonyl compounds represents a fundamental process in synthetic chemistry.^1^ Due to its innate nucleophilicity, the α-carbon of the carbonyl functionality reacts, typically as an enol/enolate intermediate, with electrophiles. In contrast, reversing the polarity of carbonyls with halogens, hypervalent iodines, transition metals and other oxidative mediators provides them with unnatural electrophilic properties and consequently greatly expands the scope of coupling partners from electrophiles to nucleophiles.^2^ In this context, umpolung tactics using hypervalent iodine reagents enabling α-functionalization of ketones with nucleophiles have gained enormous attention since their discovery in 1978 by Mizukami *et al.*^3^ In principle, there are two typical carbonyl sources used for iodine(iii)-mediated umpolung reactions.^4^ First, ketones can be treated with nucleophile-substituted aryl iodanes to implement the umpolung event (Fig. 1a, eqn (1)).^5^ However, the need for the presynthesis of chemically stable and/or isolable nucleophile-substituted aryl iodanes often restricts the scope of nucleophiles. In contrast, the use of enol silyl ethers as a carbonyl source enables the carbonyl umpolung event mediated by simple aryl iodanes which significantly expands the scope of nucleophiles (eqn (2)).^6^ In addition, Maulide and co-workers developed an electrophilic-amide-activation based umpolung protocol wherein the lutidine N-oxide as a nucleophile is used to trap the electrophilically activated amides leading to the formation of an electrophilic enolonium species thus enabling α-umpolung of amides (eqn (3)).^7^

Similar to hypervalent iodine(iii) reagents, sulfur(iv) reagents have also been extensively used to tune the reactivity of organic functionalities (Fig. 1b).^8^ As shown in Fig. 1b, a wide variety of unique sulfur(iv) reagents including alkyl, vinyl, aryl/heteroaryl and alkynyl sulfonium salts have been developed which often feature easy accessibility, structural variability, versatile reactivities and good functional group compatibility.^9–12^ However, to the best of our knowledge, there have been no reports on carbonyl umpolung by using sulfur(iv) reagents.

During the past years, we have devoted to the study of sulfonium or iodonium rearrangement reactions.^13^ In the program, we proposed an “assembly/deprotonation” protocol, the objective of which is to construct unprecedented unstable rearrangement precursors by dividing one reaction into multiple steps and finely tuning each step of the reaction. As a consequence, the tactic has enabled the development of [3,3]- and [5,5]-rearrangement of aryl sulfoxides and asymmetric rearrangement of aryl iodanes.^13*a*–*c*^ Encouraged by the success of the “assembly/deprotonation” protocol in the arrangement chemistry, we wondered if the protocol could be further utilized for developing other transformations such as the umpolung reaction. Specifically, the “assembly” of an electrophile-activated sulfoxide with 2-oxazoline and subsequent “deprotonation” of *in situ* generated N–S(iv) 2-oxazoline may lead to an umpoled 2-oxazoline (Fig. 1c). *In lieu* of a rearrangement process, the *in situ* generated sulfonium-enamine species may adopt an external nucleophile to achieve the umpolung α-functionalization of 2-oxazolines.

α-Heterofunctionalized 2-oxazolines are widely used as versatile synthetic intermediates to construct natural products, biologically active compounds and biomaterials.^14^ They also serve as useful ligands in transition metal catalysis.^15^ Moreover, 2-oxazoline as a masked carboxylic acid equivalent can be readily converted to other valuable functionalities such as acids, amides, ketones, polymers, *etc.*^16^ Despite their importance, the methods for the synthesis of α-heterofunctionalized 2-oxazolines rely heavily on the derivatization of α-heterofunctionalized carboxylic acids which are often not commercially available or difficult to synthesize.^17^ Therefore, the discovery of methods for direct α-heterofunctionalization of 2-oxazolines is a useful endeavor.

## Results and discussion

To verify our hypothesis, we first investigated the α-amination of 2-oxazoline 1a with 4-methoxyaniline. According to the “assembly/deprotonation” protocol previously developed for rearrangement reactions in our laboratory,^13*a*–*c*^ the optimization of the reaction conditions was conducted step by step in order to achieve a high efficiency of the whole process (Table 1). First, a wide variety of aryl sulfoxides and iodanes as oxidants were screened (entries 1–6). As a result, alkyl sulfoxides 2a–2e proved incompetent for the reaction since no desired product or extremely low yields were obtained in these cases (entries 1 and 2). In contrast, most of the aryl sulfoxides 2f–2n afforded the desired product 4a (entries 2–5). Among them, diaryl sulfoxides 2l and 2m were found to be the best two oxidants giving 4a in 59% and 67% yields, respectively (entries 3 and 4). The relatively higher efficiency of 2l and 2m could be attributed to their subtle electronic features that may not only meet the requirement of their electrophilic activation by anhydride but also possessed the durability in the system for the upcoming reaction sequences. In addition to aryl sulfoxides, the mostly used aryl iodanes 2o–2t are all ineffective for the reaction (entry 6). This is probably due to the compatibility issue of aryl iodanes when facing with the nucleophilic 4-methoxyaniline. Next, the choice of base was determined as another critical parameter for the reaction. DBU as base was superior to other tertiary amines (entries 4 and 7–9). The necessity of base was also proved since the reaction without base failed to give any product (entry 10). In addition, pyridines and inorganic bases were unsuitable for the reaction (for details, see the ESI†). Afterwards, the reaction temperature and reaction time were finely optimized step by step. As expected, obvious variation of chemical yields was observed when slightly tuning the reaction temperatures and reaction times for each step of the reaction (entries 11–22). This reflects the importance of the “assembly/deprotonation” protocol which enables stepwise and precise control of the reaction and thus ensures the whole efficiency of the transformation. Eventually, the reaction under the optimum conditions afforded the desired product in an excellent yield (84%) (entry 12).

**Table 1:** Optimization of reaction conditions^a^

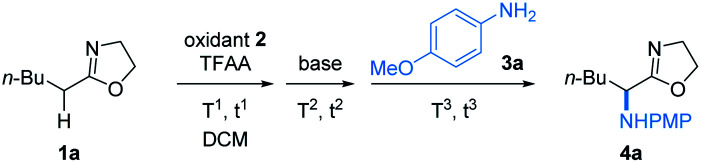						
Entry	Oxidant	Base	*T* ^1^, *t*^1^	*T* ^2^, *t*^2^	*T* ^3^, *t*^3^	Yield^b^
1	2a–2d, 2i	DBU	−40 °C, 3 h	−40 °C, 1 h	−40 °C, 12 h	0
2	2e–2h, 2j, 2k	DBU	−40 °C, 3 h	−40 °C, 1 h	−40 °C, 12 h	10 to 23
3	2l	DBU	−40 °C, 3 h	−40 °C, 1 h	−40 °C, 12 h	59
4	2m	DBU	−40 °C, 3 h	−40 °C, 1 h	−40 °C, 12 h	67
5	2n	DBU	−40 °C, 3 h	−40 °C, 1 h	−40 °C, 12 h	13
6	2o–2t	2-Methylpyridine	−40 °C, 3 h	−40 °C, 1 h	−40 °C, 12 h	Trace^c^
7	2m	NEt_3_	−40 °C, 3 h	−40 °C, 1 h	−40 °C, 12 h	66
8	2m	DIPEA	−40 °C, 3 h	−40 °C, 1 h	−40 °C, 12 h	56
9	2m	DABCO	−40 °C, 3 h	−40 °C, 1 h	−40 °C, 12 h	18
10	2m	None	−40 °C, 3 h	−40 °C, 1 h	−40 °C, 12 h	0
11	2m	DBU	−60 °C, 3 h	−40 °C, 1 h	−40 °C, 12 h	57
12	2m	DBU	−20 °C, 3 h	−40 °C, 1 h	−40 °C, 12 h	**84**
13	2m	DBU	0 °C, 3 h	−40 °C, 1 h	−40 °C, 12 h	57
14	2m	DBU	−20 °C, 6 h	−40 °C, 1 h	−40 °C, 12 h	68
15	2m	DBU	−20 °C, 10 min	−40 °C, 1 h	−40 °C, 12 h	67
16	2m	DBU	−20 °C, 3 h	−60 °C, 1 h	−40 °C, 12 h	64
17	2m	DBU	−20 °C, 3 h	−20 °C, 1 h	−40 °C, 12 h	68
18	2m	DBU	−20 °C, 3 h	−40 °C, 3 h	−40 °C, 12 h	60
19	2m	DBU	−20 °C, 3 h	−40 °C, 10 min	−40 °C, 12 h	63
20	2m	DBU	−20 °C, 3 h	−40 °C, 1 h	−60 °C, 12 h	60
21	2m	DBU	−20 °C, 3 h	−40 °C, 1 h	−20 °C, 12 h	68
22	2m	DBU	−20 °C, 3 h	−40 °C, 1 h	−40 °C, 6 h	30
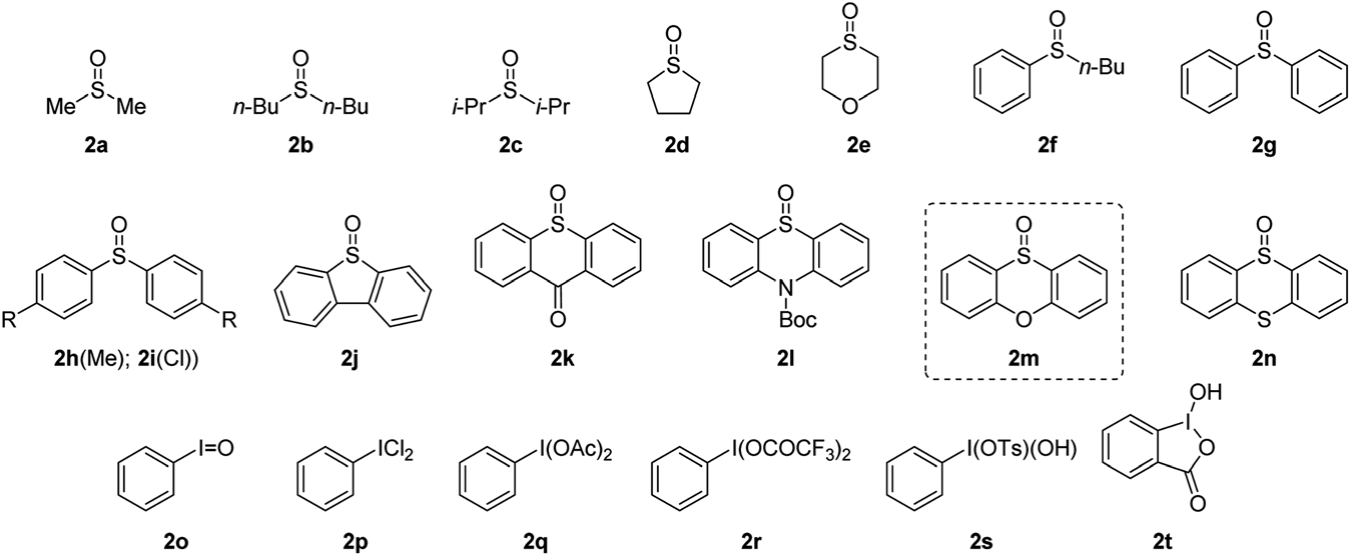						

a Reaction conditions: 2-oxazoline 1a (0.5 mmol), I(iii)/S(iv) reagent 2 (1.0 equiv.), TFAA (1.5 equiv.), DCM (0.1 M), *T*^1^ (h or min), *t*^1^ (°C); base (3.0 equiv.), *T*^2^ (h or min), *t*^2^ (°C); *p*-anisidine (2.0 equiv.), *T*^3^ (h or min), *t*^3^ (°C).

b Isolated yield.

c TMSOTf, TMSOCOCF_3_, or BF_3_·Et_2_O was used for activation of I(iii) reagents wherein 2-methylpyridine was used as the base. For more details, see the ESI.

With the optimized reaction conditions in hand, we set out to assess the generality of the reaction. As illustrated in Table 2, a wide variety of 2-oxazolines were studied under the optimum conditions. Regardless of the length of alkyl chains at the α-position of 2-oxazolines (1a–1d), the reaction afforded desired products (4a–4d) in good yields (62–84%). Remarkably, despite suffering from stereo hindrance, α-isopropyl 2-oxazoline 1e still furnished the desired product 4e albeit in a low yield (28%). To our pleasure, a set of susceptible groups, such as TBSO (4f), iodine (4g), nitrile (4j), Boc (4k), unsaturated carbonyl group (4r), and thiophene group (4v) remained intact under the conditions. Impressively, even nucleophilic functional groups such as alkene (4h), alkynyl (4i) and indolyl groups (4o) that can be readily oxidized by hypervalent iodine reagents were also tolerated in the reaction.^18^ The functional groups tolerated in the reaction provide a versatile platform for further elaboration of the products.

**Table 2:** Reaction scope^a^

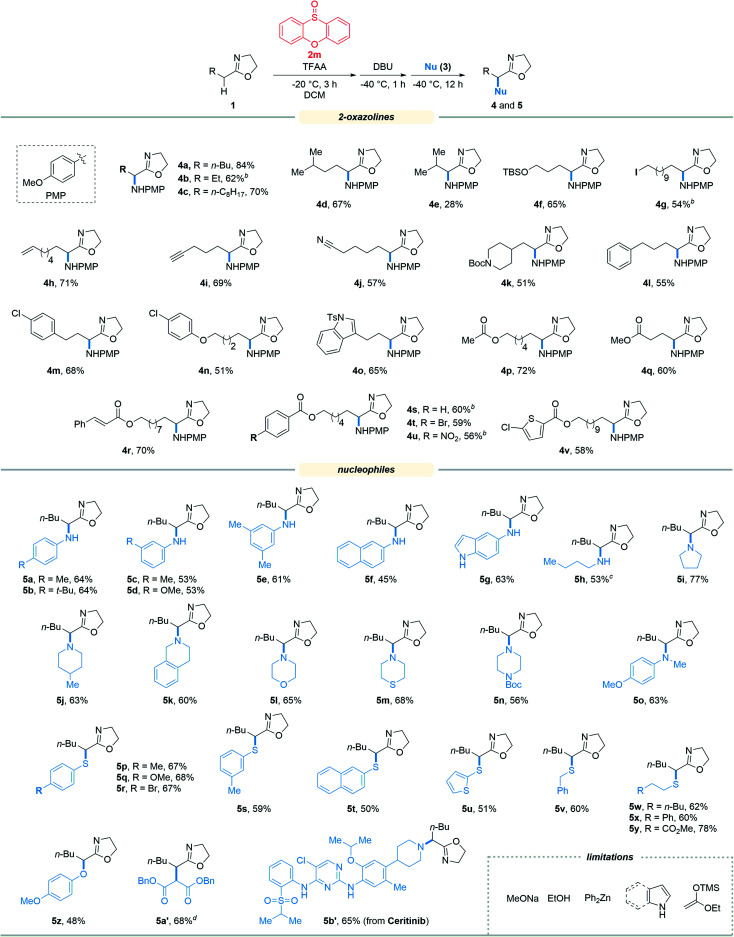

a Unless otherwise noted, the reaction was performed under the optimum conditions: 2-oxazoline 1 (0.5 mmol), 2m (1.0 equiv.), TFAA (1.5 equiv.), DCM (0.1 M), −20 °C for 3 h; then DBU (3.0 equiv.), −40 °C, 1 h; then nucleophile (2.0 equiv.), −40 °C, 12 h.

b *T* ^1^ = −40 °C, *t*^1^ = 3 h; *T*^2^ = −50 °C, *t*^2^ = 30 min.

c *n*-BuNH_2_ (3.0 equiv.) was used.

d Dibenzyl malonate was pretreated with NaH prior to be used in the reaction.

Next, the scope of nucleophiles was studied under the optimum conditions (Table 2). To our delight, both alkyl and aryl amines (3a–3o) were proved to be suitable for the reaction. In addition to primary amines (3a–3h), secondary amines (3i–3o) also afforded the desired products (5a–5o) in good yields (48–77%). Gratifyingly, both alkyl and aryl thiols (3p–3y) were suitable for the reaction. As a result, a wide variety of α-alkyl/arylamino and α-alkyl/arylthio 2-oxazolines were obtained with the protocol. The oxygen and carbon nucleophiles such as 4-methoxyphenol and dibenzyl malonate (3z and 3a′) were well adopted by the reaction. It should be noted that in the case of 3a′, dibenzyl malonate needs to be treated with NaH prior to submission to the reaction. Impressively, the practicality of the method was witnessed by the successful application of the method to a complex molecule namely ceritinib (LDK378), a novel ALK inhibitor for Non-Small Cell Lung Cancer (NSCLC), which bears multiple vulnerable functional groups such as aryl amines and pyrimidines. The limitations of the method were determined when using alkoxyl and carbon nucleophiles including sodium methoxide, ethanol, zinc reagents, indole, pyrrole and enol silyl ethers. These unsuccessful cases were probably due to the relatively weak nucleophilicity or the compatibility issues of nucleophiles towards the electrophilic reaction system.

**Fig. 2:**
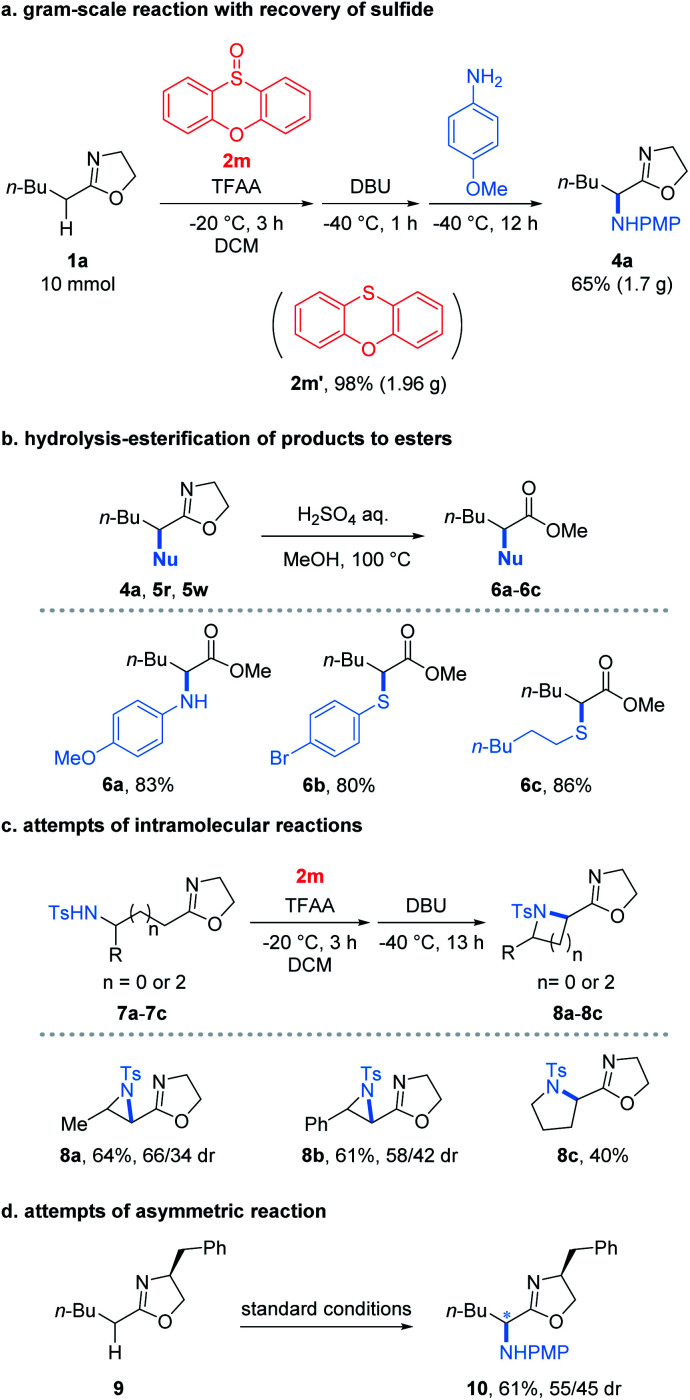
(a) Gram-scale reaction, (b) elaboration of products to esters, (c) intramolecular reaction and (d) asymmetric reaction.

To our delight, the reaction performed at the gram-scale still afforded a good chemical yield of 4a (65%) showing the practicality of the protocol (Fig. 2a). Remarkably, the sulfide 2m′ reduced from the oxidant 2m could be obtained in a nearly quantitative yield. Furthermore, the simple hydrolysis of α-heterofunctionalized 2-oxazolines furnished valuable α-N or α-S substituted esters 6a–6c in good yields (80–86%) which demonstrated the potential utility of products (Fig. 2b). Encouraged by the success of the intermolecular umpolung reaction, we attempted to examine the intramolecular version by introducing a nucleophilic NH-Ts group into 2-oxazoline substrates (Fig. 2c). Impressively, the reaction indeed afforded the desired aziridines (8a and 8b) and pyrrolidines (8c) under the conditions used for its intermolecular version. Despite the modest yields obtained, the preliminary results proved the possibility of applying the umpolung protocol for the synthesis of heterocycles. Moreover, the feasibility of the asymmetric umpolung reaction was explored by introducing chiral auxiliary into the oxazoline fragment (Fig. 2d). Although very low diastereoselectivity was determined, the concept of asymmetric umpolung of carbonyl compounds was proved.

**Fig. 3:**
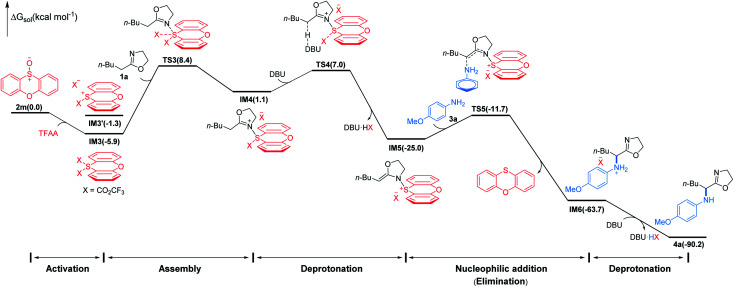
Simplified free energy profile for the conversion of 1a to 4a, calculated at the (SMD:DCM)M062X/6-311++G(d,p)//M062X/6-31G(d,p) level of theory. Energies in parentheses in kcal mol^−1^.

To gain insight into the mechanism of the conversion, we performed DFT mechanistic study using the reaction of 1a and 3a as an example. As depicted in Fig. 3, the reaction consists of five major stages. First, TFAA triggers electrophilic activation of sulfoxide 1a*via* a facile stepwise process giving an intermediate IM3 (for details, see the ESI†). It's worth noting that the zwitterionic sulfonium IM3′ is thermodynamically unfavored with respect to the neutral IM3 by about 4.6 kcal mol^−1^. The 2-oxazoline 1a is then trapped by *in situ* generated highly electrophilic λ^4^-sulfane IM3 leading to IM4 wherein the acidity of the α-proton of 2-oxazoline is dramatically increased. As a consequence, the α-deprotonation of IM4 by DBU proceeds smoothly to afford N–S(iv) enamine IM5 which is now becoming an electrophile achieving the umpolung of 2-oxazoline. Not surprisingly, the nucleophilic *p*-methoxyaniline attacks IM5 affording IM6 with C–N bond formation meanwhile releasing phenoxathiin as a side product. Finally, IM6 converts to product 4a*via* deprotonation by DBU. Overall, the nucleophilic attack of 2-oxazoline to λ^4^-sulfane IM3 by crossing TS3 is the rate determining step of the whole transformation with an energy cost of 14.3 kcal mol^−1^ and the reaction is highly exergonic by 90.2 kcal mol^−1^.^18^ The energetic results are in good agreement with the experimental observations that the reaction proceeds readily at low temperature.

## Conclusions

In summary, we have developed a sulfur(iv)-mediated umpolung strategy which enables α-heterofunctionalization of 2-oxazolines. The success of the reaction relies on the choice of a subtle oxidant called phenoxathiin sulfoxide and the use of the “assembly/deprotonation” protocol for constructing umpoled 2-oxazoline species, presumably an N–S(iv)-enamine intermediate. Prominent features of the reaction include mild conditions, excellent functional group compatibility, and broad substrate scope for both coupling partners. In addition to the intermolecular reaction, the feasibility of the intramolecular version was proved as a potential approach for the synthesis of N-heterocycles. The whole reaction sequence consisting of five stages is supported by computational studies. Efforts to develop other sulfur(iv)-mediated umpolung reactions are currently underway in our laboratory.

## Data availability

All experimental and characterization data, as well as DFT calculation data are available in the ESI.†

## Author contributions

B. P. conceived the project and co-directed the project with P. D. Q. Z. developed the reaction and performed the experiments with Y. L., R. L., Z. H, L. K and P. D. Y. L. performed DFT calculations. B. P. prepared the manuscript.

## Conflicts of interest

There are no conflicts to declare.

## Supplementary Material

SC-013-D2SC00476C-s001

## References

[cit1] (a) Guillena G. , in Comprehensive Enantioselective Organocatalysis: Catalysts, Reactions, and Applications, ed. P. I. Dalko, Wiley, Weinheim, 2013, pp. 757–790

[cit2] Seebach D., Corey E. J. (1975). J. Org. Chem..

[cit3] Mizukami F., Ando M., Tanaka T., Imamura J. (1978). Bull. Chem. Soc. Jpn..

[cit4] Merritt E. A., Olofsson B. (2011). Synthesis.

[cit5] Li Y., Hari D. P., Vita M. V., Waser J. (2016). Angew. Chem., Int. Ed..

[cit6] Arava S., Kumar J. N., Maksymenko S., Iron M. A., Parida K. N., Fristrup P., Szpilman A. M. (2017). Angew. Chem., Int. Ed..

[cit7] Gonçalves C. R., Lemmerer M., Teskey C. J., Adler P., Kaiser D., Maryasin B., González L., Maulide N. (2019). J. Am. Chem. Soc..

[cit8] Kozhushkov S. I., Alcarazo M. (2020). Eur. J. Inorg. Chem..

[cit9] Umemoto T., Ishihara S. (1993). J. Am. Chem. Soc..

[cit10] Cowper P., Jin Y., Turton M. D., Kociok-Kçhn G., Lewis S. E. (2016). Angew. Chem., Int. Ed..

[cit11] Berger F., Plutschack M. B., Riegger J., Yu W., Speicher S., Ho M., Frank N., Ritter T. (2019). Nature.

[cit12] Höfer M., Liska R. (2009). J. Polym. Sci., Part A: Polym. Chem..

[cit13] Shang L., Chang Y., Luo F., He J.-N., Huang X., Zhang L., Kong L., Li K., Peng B. (2017). J. Am. Chem. Soc..

[cit14] Yokokawa F., Sameshima H., In Y., Minoura K., Ishidab T., Shioiri T. (2002). Tetrahedron.

[cit15] Connon R., Roche B., Rokade B. V., Guiry P. J. (2021). Chem. Rev..

[cit16] Meyers V. A. I., Mihelich E. D. (1976). Angew. Chem., Int. Ed..

[cit17] (d) Wirth T. , Topics in Current Chemistry: Hypervalent Iiodine Chemistry, ed. T. Wirth, Springer, Switzerland, 2003, pp. 99–136

[cit18] The formation of α-S(iv)-oxazoline *in lieu* of N-S(iv) enamine IM5 cannot be completely excluded. However, the barrier for the nucleophilic substitution of α-S(iv)-oxazoline is calculated to be 24.3 kcal mol^−1^ which is much higher than the barrier of 13.3 kcal mol^−1^ for that of IM5. Therefore, IM5 is more likely to be the key umpoled intermediate

