# Supplementary material for: Sulfur(iv)-mediated umpolung α-heterofunctionalization of 2-oxazolines
Source: Chem Sci. 2022 Apr 9;13(18):5164–70. doi: 10.1039/d2sc00476c (PMC9093176; doi:10.1039/d2sc00476c)
Supplement: SC-013-D2SC00476C-s001 [file SC-013-D2SC00476C-s001.pdf]

Electronic Supplementary Material (ESI) for Chemical Science.
This journal is © The Royal Society of Chemistry 2022

# Supporting Information

## Sulfur(IV)-Mediated Umpolung α-Heterofunctionalization of 2-Oxazolines

Qifeng Zhang,[†] Yuchen Liang,[†] Ruiqi Li,[†] Ziyi Huang,[†] Lichun Kong,[†] Peng Du,*[, †] Bo Peng*[,†]

[†]Key Laboratory of the Ministry of Education for Advanced Catalysis Materials, Zhejiang Normal University, Jinhua 321004, China

*pengbo@zjnu.cn

*dupeng@zinu.edu.cn

## Table of Contents

**Contents**

1. General information ..........2

2. General procedure for the synthesis of starting materials. ..........2

3. Development of the umpolung α-heterofunctionalization of 2-alkyl oxazoline 1a. ..........7

4. General procedure for α-heterofunctionalization of 2-oxazolines. ..........10

5. Gram-scale synthesis, elaboration of products, intramolecualr reactions and asymmetric version. ..........31

6. Density functional theory (DFT) calculations ..........36

7.References ..........48

8. NMR spectra ..........49

# 1. General information

Unless otherwise indicated, all glassware was oven dried by heat gun before use and all reactions were performed under an atmosphere of Nitrogen. All solvents were distilled from appropriate drying agents prior to use. All reagents were used as received from commercial suppliers unless otherwise stated. Reaction progress was monitored by thin layer chromatography (TLC) performed on plastic plates coated with silica gel GF254 with 0.2 mm thickness. Chromatograms were visualized by fluorescence quenching with UV light at 254 nm or by staining using potassium permanganate. Flash column chromatography was performed using silica gel 60 (160-200 mesh). Neat infra-red spectra were recorded using a NEXUS670 FT-IR spectrometer. Wavelengths (ν) are reported in $cm^{-1}$. Mass spectra were obtained using a TOF MS instrument. All $^{1}H$ NMR, $^{13}C$ NMR spectra were recorded on Bruker AV-400 or AV-600. Chemical shifts were reported in parts per million (ppm), and the residual solvent peak was used as an internal reference: proton (chloroform *δ* 7.26), carbon (chloroform *δ* 77.16). Multiplicity was indicated as follows: s (singlet), d (doublet), t (triplet), q (quartet), m (multiplet), dd (doublet of doublet). Coupling constants were reported in Hertz (Hz).

# 2. General procedure for the synthesis of starting materials.

Sulfoxides **2a**, **2b**, **2d** and **2g-2j**, hypervalent iodine reagents **2o-2t**, 2-oxazolines **1c**, **1d**, **1j** and **1n** are commercially available. Sulfoxides **2c**[1], **2e**[2], **2f**[3], **2k**[4], **2l**[5], **2m**[2] and **2n**[5], 2-oxazolines **1a-1e**[6], **1g-h**[6], **1i**[7], **1j**[6], **1m-1n**[6], **1p-1q**[8] and **1r-1v**[6] are known compounds.

***Tert*-butyl 4-(2-(4,5-dihydrooxazol-2-yl)ethyl)piperidine-1-carboxylate (1k):**

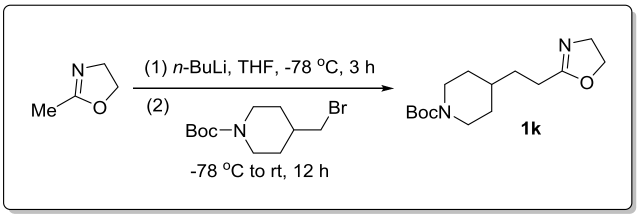


To a solution of 2-methyl-4,5-dihydrooxazole (1.70 g, 20 mmol) in THF (67 mL) was added *n*-

BuLi (2.5 M, 8.8 mL) at −78 °C. After stirring for 3 h, to the mixture was added a solution of *tert*-butyl 4-(bromomethyl)piperidine-1-carboxylate (5.54 g, 1.0 equiv) in THF (12 mL) slowly at −78 °C. The mixture was stirred at the same tempearture for 1 h. Subsequently, the mixture was warmed to rt and stirred 12 h. The mixture was then treated with water and extracted with DCM. The organic layer was dried over $Na_2SO_4$ and concentrated. The obtained residue was purified by silica gel column chromatography to afford the title compound **1k** as white solid, m.p. 59 – 60 °C, 3.01 g, 53% yield. ($R_f$ = 0.26, eluent: EtOAc).

**$^1$H NMR (600 MHz, CDCl$_3$):** $\delta$ 4.21 (t, $J$ = 9.5 Hz, 2H), 4.08 – 4.07 (m, 2H), 3.81 (t, $J$ = 9.5 Hz, 2H), 2.73 – 2.57 (m, 2H), 2.28 (t, $J$ = 7.9 Hz, 2H), 1.66 (d, $J$ = 12.3 Hz, 2H), 1.61 – 1.54 (m, 2H), 1.48 – 1.35 (m, 10H), 1.14 – 1.02 (m, 2H).

**$^{13}$C NMR (151 MHz, CDCl$_3$):** $\delta$ 168.6, 155.0, 79.4, 67.3, 54.5, 35.7, 32.6, 31.9, 28.6, 25.3.

**IR (neat, cm$^{-1}$):** 2977, 2845, 1684, 1664, 1420, 1361, 1156, 958.

**HRMS (ESI-TOF):** calculated for [$C_{15}H_{27}N_2O_3$ (M + H$^+$)]: 283.2017, found: 283.2016.

**2-(4-((*tert*-butyldimethylsilyl)oxy)butyl)-4,5-dihydrooxazole (1f):**

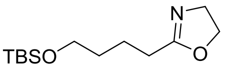


Following the procedure used for the synthesis of **1k**, compound **1f** was prepared from 20 mmol of corresponding alkyl bromide and was obtained as light yellow oil, 3.19 g, 62% yield. ($R_f$ = 0.40, eluent: PE/EtOAc = 3/1).

**$^1$H NMR (600 MHz, CDCl$_3$):** $\delta$ 4.20 (t, $J$ = 9.5 Hz, 2H), 3.80 (t, $J$ = 9.5 Hz, 2H), 3.61 (t, $J$ = 6.4 Hz, 2H), 2.28 (t, $J$ = 7.5 Hz, 2H), 1.71 – 1.63 (m, 2H), 1.61 – 1.51 (m, 2H), 0.87 (s, 9H), 0.03 (s, 6H).

**$^{13}$C NMR (151 MHz, CDCl$_3$):** $\delta$ 168.6, 67.3, 62.8, 54.5, 32.4, 27.9, 26.1, 22.5, 18.5, −5.2.

**IR (neat, cm$^{-1}$):** 2927, 2855, 1667, 1471, 1386, 1252, 1093, 987, 833, 772.

**HRMS (ESI-TOF):** calculated for [$C_{13}H_{28}NO_2Si$ (M + H$^+$)]: 258.1884, found: 258.1883.

**2-(4-phenylbutyl)-4,5-dihydrooxazole (1l):**

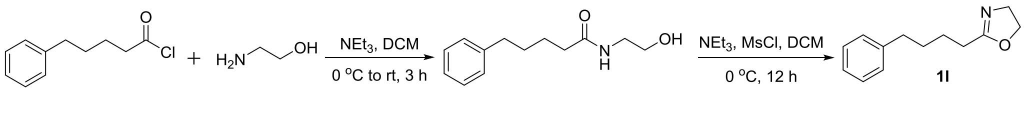


To a solution of 2-aminoethan-1-ol (1.47 g, 1.2 equiv) and $NEt_3$ (3.3 mL, 1,2 equiv) in DCM (67

mL) was added 5-phenylpentanoyl chloride (3.92 g, 20 mmol) dropwise at 0 $^{o}$C. The mixture was then warmed to rt. After stirring for 3 h, to the mixture was added DCM (33 mL) and $NEt_3$ (13.9 mL, 5.0 equiv). MsCl (1.9 mL, 1.2 equiv) was then added to the mixture dropwise at 0 $^{o}$C. The mixture was stirred for 12 h and then quenched with water, extracted with DCM, dried over $Na_2SO_4$, and concentrated. The obtained residue was purified by silica gel column chromatography to afford the title compound **1l** as light yellow oil, 3.17 g, 78% yield. ($R_f$ = 0.24, eluent: PE/EtOAc = 1/1).

**$^1$H NMR (600 MHz, $CDCl_3$):** $\delta$ 7.29 – 7.25 (m, 2H), 7.21 – 7.12 (m, 3H), 4.21 (t, $J$ = 9.5 Hz, 2H), 3.81 (t, $J$ = 9.5 Hz, 2H), 2.64 (t, $J$ = 7.0 Hz, 2H), 2.33 – 2.27 (m, 2H), 1.73 – 1.66 (m, 4H).

**$^{13}$C NMR (151 MHz, $CDCl_3$):** $\delta$ 168.5, 142.3, 128.4, 128.3, 125.8, 67.3, 54.5, 35.7, 31.1, 27.9, 25.7.

**IR (neat, cm$^{-1}$):** 3024, 2933, 2858, 1664, 1452, 1359, 1170, 983, 743.

**HRMS (ESI-TOF):** calculated for [$C_{13}H_{18}NO$ (M + $H^+$)]: 204.1383, found: 204.1380.

**2-(3-(1-tosyl-1H-indol-3-yl)propyl)-4,5-dihydrooxazole (1o):**

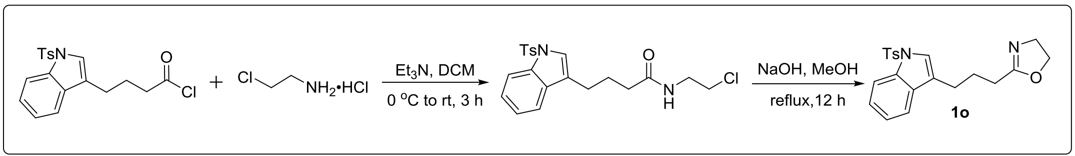


To a solution of 2-chloroethylamine hydrochloride (1.02 g, 1.1 equiv) and $NEt_3$ (2.8 mL, 2.5 equiv) in DCM (27 mL) was added 4-(1-tosyl-1*H*-indol-3-yl)butanoyl chloride (3.00 g, 8 mmol) dropwise at 0 $^{o}$C. The mixture was then warmed to rt and stirred for 3 h. Subsequently, the mixture was concentrated and purified by recrystallization (DCM and PE) to give *N*-(2-chloroethyl)-4-(1-tosyl-1*H*-indol-3-yl)butanamide as white solid, 2.74 g, 82% yield. ($R_f$ = 0.50, eluent: PE/EtOAc = 1/1).

To a solution of *N*-(2-chloroethyl)-4-(1-tosyl-1*H*-indol-3-yl)butanamide (2.74 g, 6.5 mmol) in MeOH (43 mL) was added NaOH (364.0 mg, 1.4 equiv). The mixture was refluxed for 12 h and was then concentrated, treated with saturated aqueous $NaHCO_3$ solution and extracted with DCM. The organic layer was dried over $Na_2SO_4$ and concentrated. The obtained residue was purified by silica gel column chromatography to afford the title compound **1o** as white solid, m.p. 95 – 96 $^{o}$C, 1.24 g, 50% yield. ($R_f$ = 0.18, eluent: PE/EtOAc = 1/1).

**$^{1}$H NMR (600 MHz, CDCl$_3$):** $\delta$ 7.97 (d, $J$ = 8.3 Hz, 1H), 7.73 (d, $J$ = 8.3 Hz, 2H), 7.47 (d, $J$ = 7.8 Hz, 1H), 7.34 (s, 1H), 7.31 – 7.27 (m, 1H), 7.24 – 7.16 (m, 3H), 4.21 (t, $J$ = 9.5 Hz, 2H), 3.82 (t, $J$ = 9.5 Hz, 2H), 2.72 (t, $J$ = 7.5 Hz, 2H), 2.36 – 2.29 (m, 5H), 2.06 – 1.99 (m, 2H).

**$^{13}$C NMR (151 MHz, CDCl$_3$):** $\delta$ 168.1, 144.8, 135.5, 135.4, 131.0, 129.9, 126.8, 124.7, 123.1, 123.0, 122.5, 119.6, 113.9, 67.3, 54.5, 27.6, 25.2, 24.5, 21.6.

**IR (neat, cm$^{-1}$):** 2952, 2880, 1663, 1595, 1447, 1357, 1164, 1094, 961, 743.

**HRMS (ESI-TOF):** calculated for [$C_{21}H_{23}N_2O_3S$ (M + H$^+$)]: 383.1424, found: 383.1429.

**(*R*)-*N*-(1-(4,5-dihydrooxazol-2-yl)propan-2-yl)-4-methylbenzenesulfonamide (7a):**

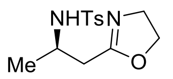


Following the procedure used for the synthesis of **1o**, compound **7a** was prepared from 10 mmol of corresponding acyl chloride and was obtained as white solid, m.p. 151 – 153 °C, 1.36 g, 48% yield. ($R_f$ = 0.37, eluent: PE/EtOAc =1:2).

**$^{1}$H NMR (600 MHz, CDCl$_3$):** $\delta$ 7.76 (d, $J$ = 8.3 Hz, 2H), 7.28 (d, $J$ = 8.0 Hz, 2H), 5.90 (d, $J$ = 8.0 Hz, 1H), 4.17 – 4.05 (m, 2H), 3.76 (t, $J$ = 9.3 Hz, 2H), 3.68 – 3.60 (m, 1H), 2.41 (s, 3H), 2.32 (d, $J$ = 5.5 Hz, 2H), 1.16 (d, $J$ = 6.7 Hz, 3H).

**$^{13}$C NMR (151 MHz, CDCl$_3$):** $\delta$ 165.2, 143.1, 138.2, 129.6, 127.0, 67.0, 54.2, 46.9, 34.2, 21.5, 21.2.

**IR (neat, cm$^{-1}$):** 3057, 2864, 1659, 1457, 1323, 1158, 1091, 992, 901, 808.

**HRMS (ESI-TOF):** calculated for [$C_{13}H_{19}N_2O_3S$ (M + H$^+$)]: 283.1111, found: 283.1113.

**(*S*)-N-(2-(4,5-dihydrooxazol-2-yl)-1-phenylethyl)-4-methylbenzenesulfonamide (7b):**

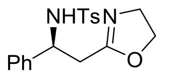


Following the procedure used for the synthesis of **1o**, compound **7b** was prepared from 10 mmol of corresponding acyl chloride and was obtained as white solid, m.p. 130 – 132 °C, 1.89 g, 55% yield. ($R_f$ = 0.30, eluent: PE/EtOAc =1:2).

**$^{1}$H NMR (400 MHz, CDCl$_3$):** $\delta$ 7.58 (d, $J$ = 8.2 Hz, 2H), 7.21 – 7.11 (m, 7H), 6.68 (s, 1H), 4.69 (d, $J$ = 5.1 Hz, 1H), 4.13 – 4.03 (m, 2H), 3.74 – 3.66 (m, 2H), 2.74 – 2.68 (m, 1H), 2.64 – 2.59 (m, 1H), 2.36 (s, 3H).

**$^{13}$C NMR (101 MHz, CDCl$_3$):** $\delta$ 164.9, 142.9, 139.8, 137.7, 129.3, 128.3, 127.5, 127.0, 126.5, 67.1, 54.7, 54.1, 34.9, 21.4.

**IR (neat, cm$^{-1}$):** 3031, 2849, 1661, 1595, 1498, 1314, 841, 814, 701.

**HRMS (ESI-TOF):** calculated for [$C_{18}H_{21}N_2O_3S$ (M + $H^+$)]: 345.1273, found: 345.1267.

***N*-(5-(4,5-dihydrooxazol-2-yl)pentyl)-4-methylbenzenesulfonamide (7c):**

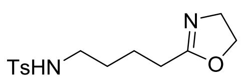


Following the procedure used for the synthesis of **1o**, compound **7c** was prepared from 10 mmol of corresponding acyl chloride and was obtained as white solid, m.p. 68 – 70 °C, 2.07 g, 70% yield. ($R_f$ = 0.30, eluent: PE/EtOAc=1:1).

**$^1$H NMR (400 MHz, CDCl$_3$):** $\delta$ 7.67 (d, $J$ = 8.2 Hz, 2H), 7.23 (d, $J$ = 8.0 Hz, 2H), 5.28 (s, 1H), 4.14 (t, $J$ = 9.5 Hz, 2H), 3.73 (t, $J$ = 9.4 Hz, 2H), 2.90 – 2.81 (m, 2H), 2.35 (s, 3H), 2.16 (t, $J$ = 6.8 Hz, 2H), 1.59 – 1.45 (m, 4H).

**$^{13}$C NMR (101 MHz, CDCl$_3$)**: $\delta$ 168.1, 143.3, 137.0, 129.7, 127.1, 54.1, 42.6, 28.8, 27.1, 22.5, 21.6.

**IR (neat, cm$^{-1}$):** 3051, 2939, 1654, 1594, 1449, 1323, 1158, 821.

**HRMS (ESI-TOF):** calculated for [$C_{14}H_{21}N_2O_3S$ (M + $H^+$)]: 297.1273, found: 297.1267.

**(*S*)-4-benzyl-2-pentyl-4,5-dihydrooxazole (9):**

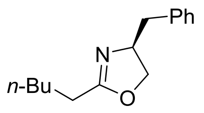


Following the procedure used for the synthesis of **1o**, compound **9** was prepared from 10 mmol of corresponding acyl chloride and was obtained as colorless oil, 1.15 g, 50% yield. ($R_f$ = 0.35, eluent: PE/EtOAc=3:1).

**$^1$H NMR (400 MHz, CDCl$_3$):** $\delta$ 7.32 – 7.28 (m, 2H), 7.24 – 7.20 (m, 3H), 4.41 – 4.38 (m, 1H), 4.17 – 4.12 (m, 1H), 3.94 (t, $J$ = 7.8 Hz, 1H), 3.14 – 3.09 (m, 1H), 2.67 – 2.62 (m, 1H), 2.26 (t, $J$ = 7.7 Hz, 2H), 1.67 – 1.56 (m, 2H), 1.37 – 1.27 (m, 4H), 0.93 – 0.88 (m, 3H).

**$^{13}$C NMR (101 MHz, CDCl$_3$)**: $\delta$ 168.1, 129.1, 128.3, 126.3, 71.3, 67.0, 31.3, 27.9, 25.6, 22.2, 13.8.

**IR (neat, cm$^{-1}$):** 3027, 2955, 1665, 1496, 1454, 732, 699.

**HRMS (ESI-TOF):** calculated for [$C_{15}H_{22}NO$ (M + $H^+$)]: 232.1701, found: 232.1696.

# 3. Development of the umpolung α-heterofunctionalization of 2-alkyl oxazoline 1a.

## 3.1. Identification of suitable oxidant.

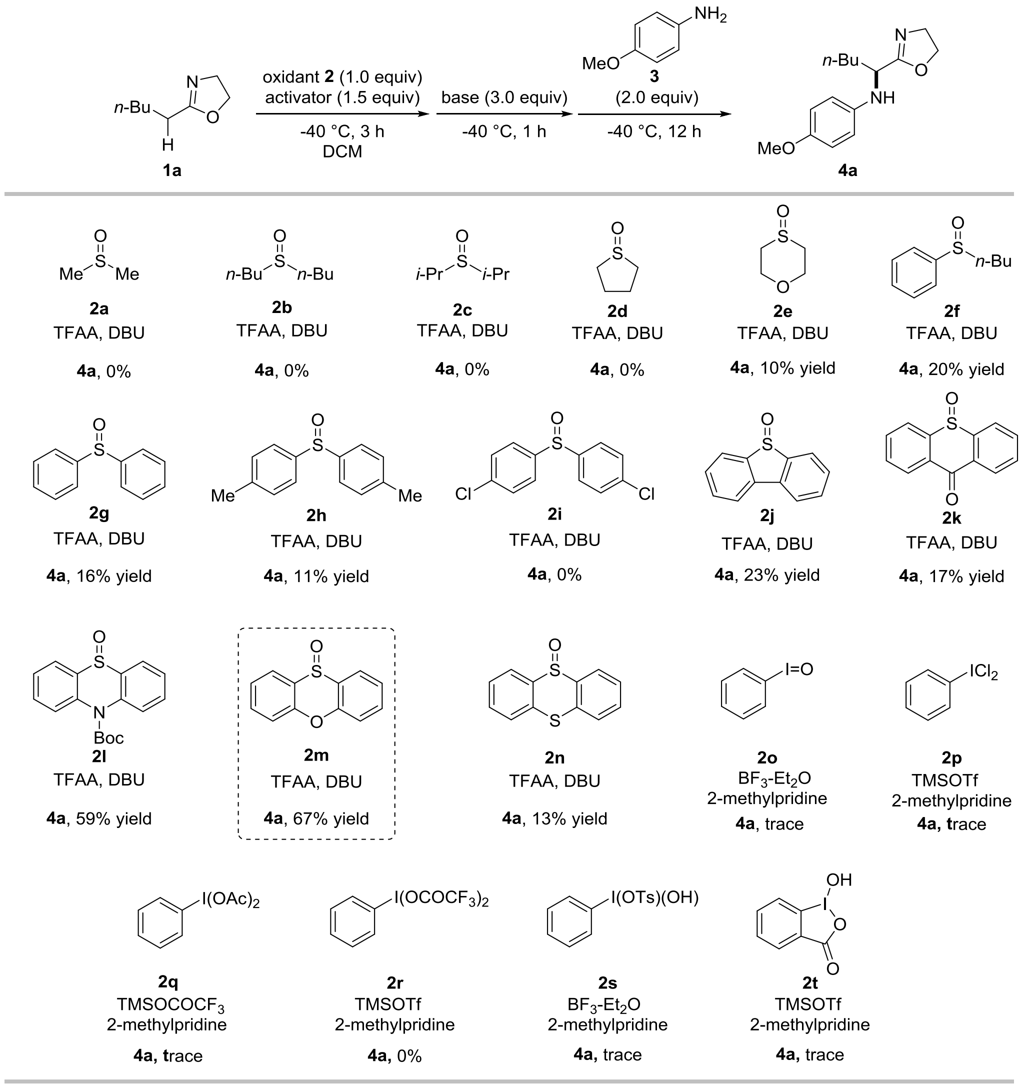


**General procedure:** To a mixture of 2-oxazoline **1a** (0.5 mmol) and oxidant **2** (1.0 equiv) in DCM (5 mL) was added activator (1.5 equiv) at –40 °C. The mixture was stirred at –40 °C for 3 h. After that, to the mixture was added a solution of base (3.0 equiv) in DCM (1 mL) dropwise in 10 min using syringe pump at –40 °C. After stirring for 1 h, to the mixture was added a solution of *p*-anisidine (2.0 equiv) in DCM (1 mL) dropwise in 10 min using syringe pump. The mixture was stirred at the same temperature for 12 h. After that, the mixture was treated with saturated aqueous $NaHCO_3$ solution and extracted with DCM. The organic layer was dried over $Na_2SO_4$ and concentrated. The obtained residue was purified by silica gel column chromatography to afford the title compound **4a**.

### 3.2. Optimization of activators, bases, thempers and times.

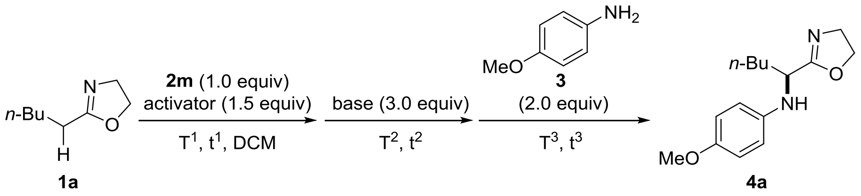


| entry | activator | base | $T^1$, $t^1$ | $T^2$, $t^2$ | $T^3$, $t^3$ | yield |
|---|---|---|---|---|---|---|
| 1 | TFAA | DBU | –40 °C, 3 h | –40 °C,1 h | –40 °C, 12 h | 67% |
| 2 | $Tf_2O$ | DBU | –40 °C, 3 h | –40 °C,1 h | –40 °C, 12 h | 16% |
| 3 | $O(COCClF_2)_2$ | DBU | –40 °C, 3 h | –40 °C,1 h | –40 °C, 12 h | 47% |
| 4 | TCAA | DBU | –40 °C, 3 h | –40 °C,1 h | –40 °C, 12 h | 24% |
| 5 | TFAA | – | –40 °C, 3 h | –40 °C,1 h | –40 °C, 12 h | 0% |
| 6 | TFAA | *N*-Ethylmorpholine | –40 °C, 3 h | –40 °C,1 h | –40 °C, 12 h | 16% |
| 7 | TFAA | 2-Picoline | –40 °C, 3 h | –40 °C,1 h | –40 °C, 12 h | 47% |
| 8 | TFAA | DABCO | –40 °C, 3 h | –40 °C,1 h | –40 °C,12 h | 18% |
| 9 | TFAA | $NEt_3$ | –40 °C, 3 h | –40 °C,1 h | –40 °C,12 h | 66% |
| 10 | TFAA | DIPEA | –40 °C, 3 h | –40 °C,1 h | –40 °C,12 h | 56% |
| 11 | TFAA | $K_2CO_3$ | –40 °C, 3 h | –40 °C,1 h | –40 °C,12 h | 0% |
| 12 | TFAA | $K_3$PO4 | –40 °C, 3 h | –40 °C,1 h | –40 °C,12 h | 0% |
| 13 | TFAA | *t*-BuOK | –40 °C, 3 h | –40 °C,1 h | –40 °C,12 h | trace |
| 14 | TFAA | $CH_3CO_2Na$ | –40 °C, 3 h | –40 °C,1 h | –40 °C,12 h | 0% |
| 15 | TFAA | DBU | –60 °C, 3 h | –40 °C,1 h | –40 °C,12 h | 57% |
| 16 | TFAA | DBU | –20 °C, 3 h | –40 °C,1 h | –40 °C,12 h | **84%** |
| 17 | TFAA | DBU | 0 °C, 3 h | –40 °C,1 h | –40 °C,12 h | 57% |
| 18 | TFAA | DBU | –20 °C, 6 h | –40 °C,1 h | –40 °C,12 h | 68% |
| 19 | TFAA | DBU | –20 °C, 10 min | –40 °C,1 h | –40 °C,12 h | 67% |
| 20 | TFAA | DBU | –20 °C, 3 h | –60 °C, 1 h | –40 °C,12 h | 64% |
| 21 | TFAA | DBU | –20 °C, 3 h | –20 °C, 1 h | –40 °C,12 h | 68% |
| 22 | TFAA | DBU | –20 °C, 3 h | –40 °C, 3 h | –40 °C,12 h | 60% |
| 23 | TFAA | DBU | –20 °C, 3 h | –40 °C, 10 min | –40 °C,12 h | 63% |
| 24 | TFAA | DBU | –20 °C, 3 h | –40 °C,1 h | –60 °C, 12 h | 60% |
| 25 | TFAA | DBU | –20 °C, 3 h | –40 °C,1 h | –20 °C, 12 h | 68% |
| 26 | TFAA | DBU | –20 °C, 3 h | –40 °C,1 h | –40 °C, 6 h | 30% |

**General procedure:** To a mixture of 2-oxazoline **1a** (0.5 mmol) and oxidant **2m** (1.0 equiv) in DCM (5 mL) was added activator (1.5 equiv) at the indicated temperature ($T^1$). The mixture was stirred at temperature ($T^1$) for the indicated time ($t^1$). After that, to the mixture was added a solution of base (3.0 equiv) in DCM (1 mL) dropwise in 10 min using syringe pump at the

indicated temperature ($T^2$). After stirring for the indicated time ($t^2$), to the mixture was added a solution of *p*-anisidine (2.0 equiv) in DCM (1 mL) dropwise in 10 min using syringe pump at the indicated temperature ($T^3$). The mixture was stirred at the same temperature for the indicated time ($t^3$). After that, the mixture was treated with saturated aqueous $NaHCO_3$ solution and extracted with DCM. The organic layer was dried over $Na_2SO_4$ and concentrated. The obtained residue was purified by silica gel column chromatography to afford the title compound **4a**.

## 4. General procedure for α-heterofunctionalization of 2-oxazolines.

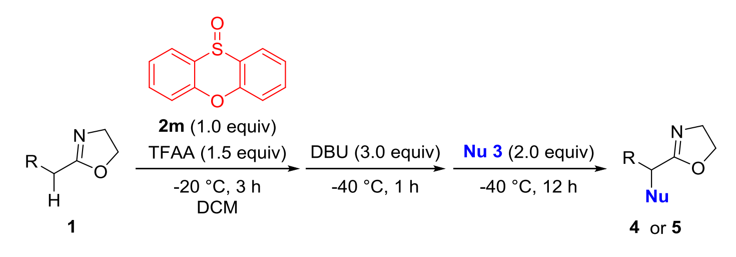


**General procedure:** To a mixture of 2-oxazoline **1** (0.5 mmol) and sulfoxide **2m** (108.0 mg, 1.0 equiv) in DCM (5 mL) was added TFAA (104 μL, 1.5 equiv) at −20 °C. After stirring for 3 h, the reaction mixture was then cooled to −40 °C. To the mixture was added a solution of DBU (228.4 mg, 3.0 equiv) in DCM (1 mL) dropwise in 10 min using syringe pump. After stirring for 1 h, to the mixture was added a solution of nucleophile (1.0 mmol, 2.0 equiv) in DCM (1 mL) dropwise in 10 min using syringe pump. The mixture was then stirred for 12 h. After that, the mixture was treated with saturated aqueous $NaHCO_3$ solution and extracted with DCM. The organic layer was dried over $Na_2SO_4$ and concentrated. The obtained residue was purified by silica gel column chromatography to afford the title compound **4** or **5**.

***N*-(1-(4,5-dihydrooxazol-2-yl)pentyl)-4-methoxyaniline (4a):**

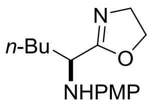

Following the general procedure, the title compound was obtained as light yellow solid, m.p. 73 – 75 °C, 110.9 mg, 84% yield. ($R_f$ = 0.20, eluent: PE/EtOAc = 3/1).

**$^1$H NMR (600 MHz, CDCl$_3$):** δ 6.76 (d, *J* = 8.9 Hz, 2H), 6.63 (d, *J* = 8.9 Hz, 2H), 4.30 – 4.14 (m, 2H), 4.11 – 4.03 (m, 1H), 3.82 (t, *J* = 9.4 Hz, 2H), 3.77 (s, 1H), 3.73 (s, 3H), 1.81 – 1.71 (m, 2H), 1.46 – 1.31 (m, 4H), 0.90 (t, *J* = 7.1 Hz, 3H).

**$^{13}$C NMR (151 MHz, CDCl$_3$):** δ 169.1, 152.6, 141.3, 115.2, 114.9, 67.8, 55.8, 54.3, 53.1, 33.5, 28.0, 22.6, 14.1.

**IR (neat, cm$^{-1}$):** 3275, 2955, 2830, 1654, 1508, 1440, 1235, 1178, 982, 812, 772.

**HRMS (ESI-TOF):** calculated for [$C_{15}H_{23}N_2O_2$ (M + H$^+$)]: 263.1754, found: 263.1759.

***N*-(1-(4,5-dihydrooxazol-2-yl)propyl)-4-methoxyaniline (4b):**

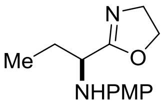

Following a procedure similar to the general procedure wherein $T^1$ = −40 °C, $t^1$ =

3 h and $T^2 = -50$ °C, $t^2 = 30$ min, the title compound was obtained as light yellow solid, m.p. 65 – 68 °C, 73.1 mg, 62% yield. ($R_f = 0.16$, eluent: PE/EtOAc = 1/1).

**$^1$H NMR (600 MHz, $CDCl_3$):** $\delta$ 6.75 (d, $J$ = 8.9 Hz, 2H), 6.63 (d, $J$ = 8.9 Hz, 2H), 4.29 – 4.15 (m, 2H), 4.03 (t, $J$ = 6.4 Hz, 1H), 3.82 (t, $J$ = 9.4 Hz, 2H), 3.72 (s, 3H), 1.88 – 1.75 (m, 2H), 0.99 (t, $J$ = 7.4 Hz, 3H).

**$^{13}$C NMR (151 MHz, $CDCl_3$):** $\delta$ 168.9, 152.5, 141.2, 115.1, 114.9, 67.8, 55.8, 54.22, 54.20, 26.6, 10.2.

**IR (neat, cm$^{-1}$):** 3269, 2960, 2831, 1656, 1508, 1458, 1230, 1141, 953, 816.

**HRMS (ESI-TOF):** calculated for [$C_{13}H_{19}N_2O_2$ (M + $H^+$)]: 235.1441, found: 235.1452.

***N*-(1-(4,5-dihydrooxazol-2-yl)nonyl)-4-methoxyaniline (4c):**

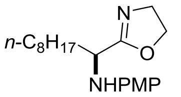


Following the general procedure, the title compound was obtained as light yellow solid, m.p. 82 – 84 °C, 112.2 mg, 70% yield. ($R_f = 0.30$, eluent: PE/EtOAc = 3/1).

**$^1$H NMR (600 MHz, $CDCl_3$):** $\delta$ 6.75 (d, $J$ = 8.8 Hz, 2H), 6.63 (d, $J$ = 8.8 Hz, 2H), 4.30 – 4.13 (m, 2H), 4.07 (t, $J$ = 6.5 Hz, 1H), 3.89 – 3.74 (m, 3H), 3.72 (s, 3H), 1.83 – 1.69 (m, 2H), 1.48 – 1.34 (m, 2H), 1.31 – 1.16 (m, 10H), 0.87 (t, $J$ = 7.0 Hz, 3H).

**$^{13}$C NMR (151 MHz, $CDCl_3$):** $\delta$ 169.1, 152.6, 141.2, 115.1, 114.9, 67.7, 55.8, 54.2, 53.0, 33.7, 31.9, 29.5, 29.4, 29.3, 25.8, 22.7, 14.2.

**IR (neat, cm$^{-1}$):** 3257, 2918, 2849, 1658, 1467, 1230, 1036, 951, 828, 717.

**HRMS (ESI-TOF):** calculated for [$C_{19}H_{31}N_2O_2$ (M + $H^+$)]: 319.2380, found: 319.2383.

***N*-(1-(4,5-dihydrooxazol-2-yl)-4-methylpentyl)-4-methoxyaniline (4d):**

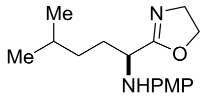


Following the general procedure, the title compound was obtained as light yellow solid, m.p. 101 – 104 °C, 92.3 mg, 67% yield. ($R_f = 0.23$, eluent: PE/EtOAc = 2/1).

**$^1$H NMR (600 MHz, $CDCl_3$):** $\delta$ 6.75 (d, $J$ = 8.9 Hz, 2H), 6.63 (d, $J$ = 8.9 Hz, 2H), 4.29 – 4.15 (m, 2H), 4.04 (t, $J$ = 6.3 Hz, 1H), 3.85 – 3.75 (m, 3H), 3.72 (s, 3H), 1.85 – 1.70 (m, 2H), 1.58 – 1.52 (m, 1H), 1.38 – 1.22 (m, 2H), 0.91 – 0.84 (m, 6H).

**$^{13}$C NMR (151 MHz, $CDCl_3$):** $\delta$ 169.1, 152.6, 141.2, 115.1, 114.9, 67.7, 55.8, 54.2, 53.3, 34.8, 31.6, 28.0, 22.61, 22.59.

**IR (neat, cm$^{-1}$):** 3283, 2953, 2868, 1655, 1510, 1464, 1305, 1234, 949, 821.

**HRMS (ESI-TOF):** calculated for [$C_{16}H_{25}N_2O_2$ (M + $H^+$)]: 277.1911, found: 277.1909.

***N*-(1-(4,5-dihydrooxazol-2-yl)-2-methylpropyl)-4-methoxyaniline (4e):**

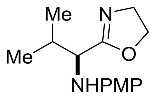


Following the general procedure, the title compound was obtained as light yellow solid, m.p. 128 – 130 °C, 34.2 mg, 28% yield. (Rf = 0.20, eluent: PE/EtOAc = 2/1).

**$^1$H NMR (600 MHz, CDCl$_3$):** $\delta$ 6.75 (d, $J$ = 8.7 Hz, 2H), 6.63 (d, $J$ = 8.7 Hz, 2H), 4.26 – 4.16 (m, 2H), 3.92 – 3.77 (m, 4H), 3.72 (s, 3H), 2.09 – 2.02 (m, 1H), 1.05 (d, $J$ = 6.8 Hz, 3H), 1.00 (d, $J$ = 6.7 Hz, 3H).

**$^{13}$C NMR (151 MHz, CDCl$_3$):** $\delta$ 168.3, 152.4, 141.6, 115.1, 114.8, 67.6, 58.9, 55.7, 54.1, 31.7, 19.2, 19.0.

**IR (neat, cm$^{-1}$):** 3277, 2954, 2903, 1654, 1509, 1463, 1228, 1038, 984, 824.

**HRMS (ESI-TOF):** calculated for [$C_{14}H_{21}N_2O_2$ (M + $H^+$)]: 249.1598, found: 249.1603.

***N*-(4-((*tert*-butyldimethylsilyl)oxy)-1-(4,5-dihydrooxazol-2-yl)butyl)-4-methoxyaniline (4f):**

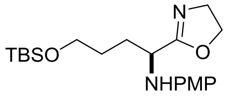


Following the general procedure, the title compound was obtained as light yellow solid, m.p. 62 – 63 °C, 123.6 mg, 65% yield. ($R_f$ = 0.17, eluent: PE/EtOAc = 3/1).

**$^1$H NMR (600 MHz, CDCl$_3$):** $\delta$ 6.77 (d, $J$ = 8.9 Hz, 2H), 6.65 (d, $J$ = 8.9 Hz, 2H), 4.32 – 4.16 (m, 2H), 4.11 (t, $J$ = 6.5 Hz, 1H), 3.89 – 3.78 (m, 3H), 3.74 (s, 3H), 3.65 (t, $J$ = 6.2 Hz, 2H), 1.93 – 1.82 (m, 2H), 1.70 – 1.59 (m, 2H), 0.90 (s, 9H), 0.05 (s, 6H).

**$^{13}$C NMR (151 MHz, CDCl$_3$):** $\delta$ 169.0, 152.6, 141.2, 115.1, 114.9, 67.8, 62.7, 55.8, 54.2, 52.9, 30.0, 29.0, 26.1, 18.4, –5.2.

**IR (neat, cm$^{-1}$):** 3313, 2952, 2855, 1651, 1521, 1463, 1234, 1103, 986, 835.

**HRMS (ESI-TOF):** calculated for [$C_{20}H_{35}N_2O_3Si$ (M + $H^+$)]: 379.2411, found: 379.2419.

***N*-(1-(4,5-dihydrooxazol-2-yl)-12-iodododecyl)-4-methoxyaniline (4g):**

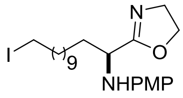


Following a procedure similar to the general procedure wherein $T^1$ = −40 °C,

$t^1$ = 3 h and $T^2$ = −50 °C, $t^2$ = 30 min, the title compound was obtained as light yellow solid, m.p. 89 – 90 °C, 130.8 mg, 54% yield. ($R_f$ = 0.29, eluent: PE/EtOAc = 2/1).

**$^1$H NMR (600 MHz, CDCl$_3$):** $\delta$ 6.74 (d, $J$ = 8.9 Hz, 2H), 6.62 (d, $J$ = 8.9 Hz, 2H), 4.27 – 4.15 (m, 2H), 4.06 (t, $J$ = 6.5 Hz, 1H), 3.87 – 3.74 (m, 3H), 3.71 (s, 3H), 3.17 (t, $J$ = 7.0 Hz, 2H), 1.85 – 1.68 (m, 4H), 1.45 – 1.33 (m, 4H), 1.31 – 1.19 (m, 12H).

**$^{13}$C NMR (151 MHz, CDCl$_3$):** $\delta$ 169.0, 152.5, 141.2, 115.0, 114.8, 67.7, 55.7, 54.2, 53.0, 33.62, 33.60, 30.5, 29.5, 29.46, 29.43, 29.39, 28.6, 25.7, 7.4.

**IR (neat, cm$^{-1}$):** 3284, 2912, 2846, 1655, 1508, 1466, 1230, 1033, 981, 719.

**HRMS (ESI-TOF):** calculated for [$C_{22}H_{36}N_2O_2I$ (M + $H^+$)]: 487.1816, found: 487.1817.

***N*-(1-(4,5-dihydrooxazol-2-yl)oct-7-en-1-yl)-4-methoxyaniline (4h):**

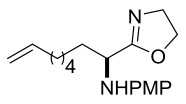


Following the general procedure, the title compound was obtained as light yellow solid, m.p. 72 – 73 °C, 107.2 mg, 71% yield. ($R_f$ = 0.22, eluent: PE/EtOAc = 2/1).

**$^1$H NMR (600 MHz, CDCl$_3$):** $\delta$ 6.75 (d, $J$ = 8.9 Hz, 2H), 6.62 (d, $J$ = 8.9 Hz, 2H), 5.82 – 5.73 (m, 1H), 4.99 – 4.96 (m, 1H), 4.93 – 4.91 (m, 1H), 4.29 – 4.14 (m, 2H), 4.07 (t, $J$ = 6.7 Hz, 1H), 3.86 – 3.74 (m, 3H), 3.72 (s, 3H), 2.06 – 2.00 (m, 2H), 1.82 – 1.70 (m, 2H), 1.46 – 1.32 (m, 6H).

**$^{13}$C NMR (151 MHz, CDCl$_3$):** $\delta$ 169.0, 152.5, 141.1, 139.0, 115.0, 114.8, 114.4, 67.7, 55.7, 54.2, 53.0, 33.7, 33.6, 28.9, 28.7, 25.6.

**IR (neat, cm$^{-1}$):** 3271, 2922, 2851, 1655, 1508, 1462, 1230, 1141, 953, 816;.

**HRMS (ESI-TOF):** calculated for [$C_{18}H_{27}N_2O_2$ (M + $H^+$)]: 303.2067, found: 303.2066.

***N*-(1-(4,5-dihydrooxazol-2-yl)hex-5-yn-1-yl)-4-methoxyaniline (4i):**

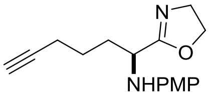


Following the general procedure, the title compound was obtained as light yellow solid, m.p. 92 – 94 °C, 94.1 mg, 69% yield. ($R_f$ = 0.29, eluent: PE/EtOAc = 1/1).

**$^1$H NMR (600 MHz, CDCl$_3$):** $\delta$ 6.74 (d, $J$ = 8.8 Hz, 2H), 6.62 (d, $J$ = 8.8 Hz, 2H), 4.27 – 4.16 (m, 2H), 4.09 (t, $J$ = 6.4 Hz, 1H), 3.89 – 3.75 (m, 3H), 3.71 (s, 3H), 2.28 – 2.14 (m, 2H), 1.95 (t, $J$ = 2.4 Hz, 1H), 1.93 – 1.81 (m, 2H), 1.73 – 1.56 (m, 2H).

**$^{13}$C NMR (151 MHz, CDCl$_3$):** $\delta$ 168.7, 152.6, 141.0, 115.1, 114.8, 83.8, 68.9, 67.8, 55.7, 54.2,

52.6, 32.5, 24.7, 18.2.

**IR (neat, cm$^{-1}$):** 3260, 2950, 2833, 1655, 1508, 1438, 1233, 1178, 1030, 980, 821.

**HRMS (ESI-TOF):** calculated for [$C_{16}H_{21}N_2O_2$ (M + $H^+$)]: 273.1598, found: 273.1606.

**6-(4,5-dihydrooxazol-2-yl)-6-((4-methoxyphenyl)amino)hexanenitrile (4j):**

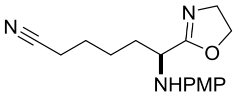


Following the general procedure, the title compound was obtained as light yellow solid, m.p. 102 – 104 °C, 81.4 mg, 57% yield. ($R_f$ = 0.29, eluent: EtOAc).

**$^1$H NMR (600 MHz, CDCl$_3$):** $\delta$ 6.76 (d, $J$ = 8.9 Hz, 2H), 6.62 (d, $J$ = 8.9 Hz, 2H), 4.35 – 4.16 (m, 2H), 4.09 (t, $J$ = 6.4 Hz, 1H), 3.82 (t, $J$ = 9.4 Hz, 3H), 3.72 (s, 3H), 2.34 (t, $J$ = 7.1 Hz, 2H), 1.90 – 1.75 (m, 2H), 1.73 – 1.64 (m, 2H), 1.62 – 1.49 (m, 2H).

**$^{13}$C NMR (151 MHz, CDCl$_3$):** $\delta$ 168.5, 152.7, 140.8, 119.6, 115.2, 114.9, 67.9, 55.7, 54.2, 52.7, 32.6, 25.2, 24.8, 17.1.

**IR (neat, cm$^{-1}$):** 3368, 3261, 2929, 2243, 1665, 1507, 1234, 1183, 947, 826.

**HRMS (ESI-TOF):** calculated for [$C_{16}H_{22}N_3O_2$ (M + $H^+$)]: 288.1707, found: 288.1706.

***Tert*-butyl-4-(2-(4,5-dihydrooxazol-2-yl)-2-((4-methoxyphenyl)amino)ethyl)piperidine-1-carboxylate (4k):**

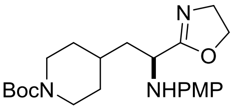


Following the general procedure, the title compound was obtained as light yellow solid, m.p. 130 – 132 °C, 103.1 mg, 51% yield. ($R_f$ = 0.30, eluent: EtOAc).

**$^1$H NMR (600 MHz, CDCl$_3$):** $\delta$ 6.73 (d, $J$ = 8.7 Hz, 2H), 6.61 (d, $J$ = 8.7 Hz, 2H), 4.24 – 4.12 (m, 3H), 4.11 – 3.94 (m, 2H), 3.78 (t, $J$ = 9.4 Hz, 2H), 3.70 (s, 3H), 2.64 (s, 2H), 1.75 – 1.60 (m, 5H), 1.42 (s, 9H), 1.17 – 1.06 (m, 2H).

**$^{13}$C NMR (151 MHz, CDCl$_3$):** $\delta$ 169.0, 154.8, 152.6, 140.9, 115.1, 114.8, 79.3, 67.7, 55.7, 54.2, 50.4, 40.5, 32.7, 32.3, 31.8, 28.5.

**IR (neat, cm$^{-1}$):** 3288, 2926, 2845, 1696, 1526, 1464, 1363, 1037, 955, 818.

**HRMS (ESI-TOF):** calculated for [$C_{22}H_{34}N_3O_4$ (M + $H^+$)]: 404.2544, found: 404.2544.

***N*-(1-(4,5-dihydrooxazol-2-yl)-4-phenylbutyl)-4-methoxyaniline (4l):**

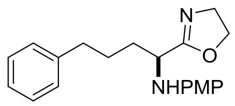


Following the general procedure, the title compound was obtained as light yellow solid, m.p. 95 – 97 °C, 89.1 mg, 55% yield. ($R_f$ = 0.22, eluent: PE/EtOAc = 2/1).

**$^1$H NMR (600 MHz, $CDCl_3$):** $\delta$ 7.28 (d, *J* = 7.0 Hz, 2H), 7.22 – 7.13 (m, 3H), 6.76 (d, *J* = 8.4 Hz, 2H), 6.63 (d, *J* = 8.5 Hz, 2H), 4.27 – 4.15 (m, 2H), 4.11 (s, 1H), 3.81 (t, *J* = 9.3 Hz, 2H), 3.73 (s, 3H), 2.65 (t, *J* = 6.7 Hz, 2H), 1.90 – 1.71 (m, 4H).

**$^{13}$C NMR (151 MHz, $CDCl_3$):** $\delta$ 169.0, 152.6, 142.0, 141.1, 128.5, 128.4, 126.0, 115.2, 114.9, 67.8, 55.8, 54.2, 53.0, 35.6, 33.2, 27.5.

**IR (neat, $cm^{-1}$):** 3284, 2934, 2859, 1651, 1524, 1457, 1229, 1038, 947, 816.

**HRMS (ESI-TOF):** calculated for [$C_{20}H_{25}N_2O_2$ (M + $H^+$)]: 325.1911, found: 325.1920.

***N*-(3-(4-chlorophenyl)-1-(4,5-dihydrooxazol-2-yl)propyl)-4-methoxyaniline (4m):**

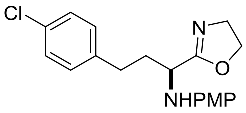


Following the general procedure, the title compound was obtained as light yellow solid, m.p. 96 – 98 °C, 117.6 mg, 68% yield. ($R_f$ = 0.24, eluent: PE/EtOAc = 1/1).

**$^1$H NMR (600 MHz, $CDCl_3$):** $\delta$ 7.24 (d, *J* = 8.3 Hz, 2H), 7.12 (d, *J* = 8.3 Hz, 2H), 6.76 (d, *J* = 8.9 Hz, 2H), 6.60 (d, *J* = 8.9 Hz, 2H), 4.29 – 4.14 (m, 2H), 4.08 (t, *J* = 6.0 Hz, 1H), 3.85 (s, 1H), 3.80 (t, *J* = 9.5 Hz, 2H), 3.73 (s, 3H), 2.74 (t, *J* = 7.0 Hz, 2H), 2.14 – 2.01 (m, 2H).

**$^{13}$C NMR (151 MHz, $CDCl_3$):** $\delta$ 168.7, 152.7, 140.9, 139.7, 131.8, 130.0, 128.6, 115.2, 114.9, 67.9, 55.8, 54.2, 52.2, 34.9, 31.3.

**IR (neat, $cm^{-1}$):** 3268, 2929, 2833, 1654, 1507, 1235, 1089, 1011, 949, 813.

**HRMS (ESI-TOF):** calculated for [$C_{19}H_{22}ClN_2O_2$ (M + $H^+$)]: 345.1364, found: 345.1368.

***N*-(5-(4-chlorophenoxy)-1-(4,5-dihydrooxazol-2-yl)pentyl)-4-methoxyaniline (4n):**

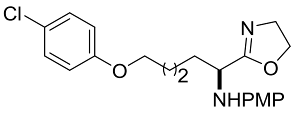


Following the general procedure, the title compound was obtained as light yellow solid, m.p. 111 – 114 °C, 98.9 mg, 51% yield. ($R_f$= 0.21, eluent: PE/EtOAc = 1/1).

**$^1$H NMR (600 MHz, $CDCl_3$):** $\delta$ 7.20 (d, *J* = 9.0 Hz, 2H), 6.80 – 6.74 (m, 4H), 6.64 (d, *J* = 8.9 Hz, 2H), 4.29 – 4.15 (m, 2H), 4.11 (t, *J* = 6.5 Hz, 1H), 3.90 (t, *J* = 6.4 Hz, 2H), 3.82 (t, *J* = 9.4 Hz, 2H), 3.73 (s, 3H), 1.91 – 1.77 (m, 4H), 1.67 – 1.52 (m, 2H).

**$^{13}$C NMR (151 MHz, CDCl$_3$):** $\delta$ 168.9, 157.7, 152.6, 141.0, 129.3, 125.4, 115.8, 115.2, 114.9, 67.9, 67.8, 55.8, 54.2, 52.9, 33.2, 29.0, 22.3.

**IR (neat, cm$^{-1}$):** 3266, 2919, 2853, 1653, 1507, 1467, 1246, 1036, 822, 667.

**HRMS (ESI-TOF):** calculated for [$C_{21}H_{26}ClN_2O_3$ (M + H$^+$)]: 389.1626, found: 389.1622.

***N*-(1-(4,5-dihydrooxazol-2-yl)-3-(1-tosyl-1H-indol-3-yl)propyl)-4-methoxyaniline(4o):**

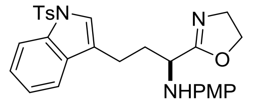


Following the general procedure, the title compound was obtained as light yellow solid, m.p. 153 – 154 °C, 163.2 mg, 65% yield. ($R_f$ = 0.32, eluent: PE/EtOAc = 1/1).

**$^1$H NMR (600 MHz, CDCl$_3$):** $\delta$ 7.98 (d, *J* = 8.3 Hz, 1H), 7.74 (d, *J* = 8.3 Hz, 2H), 7.43 (d, *J* = 7.8 Hz, 1H), 7.36 (s, 1H), 7.32 – 7.28 (m, 1H), 7.23 – 7.15 (m, 3H), 6.74 (d, *J* = 8.8 Hz, 2H), 6.59 (d, *J* = 8.8 Hz, 2H), 4.28 – 4.19 (m, 2H), 4.17 – 4.11 (m, 1H), 3.94 (s, 1H), 3.83 (t, *J* = 9.5 Hz, 2H), 3.73 (s, 3H), 2.86 – 2.77 (m, 2H), 2.29 (s, 3H), 2.25 – 2.10 (m, 2H).

**$^{13}$C NMR (101 MHz, CDCl$_3$):** $\delta$ 168.6, 152.7, 144.8, 140.9, 135.4, 135.3, 130.9, 129.9, 126.8, 124.8, 123.1, 123.0, 122.2, 119.5, 115.2, 114.9, 113.8, 67.9, 55.8, 54.2, 52.4, 32.5, 21.6, 21.1.

**IR (neat, cm$^{-1}$):** 3272, 3108, 2917, 1664, 1506, 1447, 1363, 1232, 1039, 815.

**HRMS (ESI-TOF):** calculated for [$C_{28}H_{30}N_3O_4S$ (M + H$^+$)]: 504.1952, found: 504.1950.

**7-(4,5-dihydrooxazol-2-yl)-7-((4-methoxyphenyl)amino)heptyl acetate (4p):**

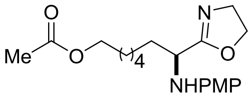


Following the general procedure, the title compound was obtained as light yellow solid, m.p. 85 – 87 °C, 125.6 mg, 72% yield. ($R_f$ = 0.20, eluent: PE/EtOAc = 1/1).

**$^1$H NMR (600 MHz, CDCl$_3$):** $\delta$ 6.73 (d, *J* = 8.8 Hz, 2H), 6.60 (d, *J* = 8.8 Hz, 2H), 4.26 – 4.15 (m, 2H), 4.05 (t, *J* = 6.5 Hz, 1H), 4.01 (t, *J* = 6.7 Hz, 2H), 3.79 (t, *J* = 9.4 Hz, 2H), 3.70 (s, 3H), 2.01 (s, 3H), 1.84 – 1.69 (m, 2H), 1.61 – 1.55 (m, 2H), 1.44 – 1.29 (m, 6H).

**$^{13}$C NMR (151 MHz, CDCl$_3$):** $\delta$ 171.3, 169.0, 152.6, 141.1, 115.1, 114.9, 67.8, 64.6, 55.8, 54.2, 52.9, 33.5, 29.1, 28.6, 25.8, 25.6, 21.1.

**IR (neat, cm$^{-1}$):** 3284, 2927, 2849, 1729, 1661, 1506, 1248, 1033, 830, 732.

**HRMS (ESI-TOF):** calculated for [$C_{19}H_{29}N_2O_4$ (M + H$^+$)]: 349.2122, found: 349.2124.

**4-(4,5-dihydrooxazol-2-yl)-4-((4-methoxyphenyl)amino)butanoate (4q):**

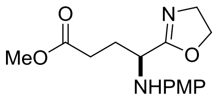


Following the general procedure, the title compound was obtained as light yellow solid, m.p. 84 – 86 °C, 87.2 mg, 60% yield. ($R_f$ = 0.11, eluent: PE/EtOAc = 1/1).

**$^{1}$H NMR (600 MHz, CDCl$_3$):** $\delta$ 6.74 (d, $J$ = 8.8 Hz, 2H), 6.62 (d, $J$ = 8.8 Hz, 2H), 4.29 – 4.17 (m, 2H), 4.13 (s, 1H), 3.91 (s, 1H), 3.81 (t, $J$ = 9.5 Hz, 2H), 3.71 (s, 3H), 3.64 (s, 3H), 2.47 (t, $J$ = 6.7 Hz, 2H), 2.15 – 2.05 (m, 2H).

**$^{13}$C NMR (151 MHz, CDCl$_3$):** $\delta$ 173.6, 168.4, 152.7, 140.8, 115.2, 114.9, 67.9, 55.7, 54.2, 52.3, 51.8, 30.3, 28.4.

**IR (neat, cm$^{-1}$):** 3307, 2920, 2833, 1724, 1653, 1507, 1228, 1173, 1032, 824.

**HRMS (ESI-TOF):** calculated for [$C_{15}H_{21}N_2O_4$ (M + $H^+$)]: 293.1496, found: 293.1499.

**10-(4,5-dihydrooxazol-2-yl)-10-((4-methoxyphenyl)amino)decyl cinnamate (4r):**

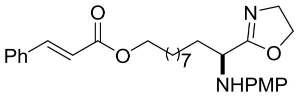


Following the general procedure, the title compound was obtained as light yellow solid, m.p. 74 – 75 °C, 168.2 mg, 70% yield. ($R_f$ = 0.35, eluent: PE/EtOAc = 3/1).

**$^{1}$H NMR (600 MHz, CDCl$_3$):** $\delta$ 7.67 (d, $J$ = 16.0 Hz, 1H), 7.51 (d, $J$ = 3.7 Hz, 2H), 7.40 – 7.32 (m, 3H), 6.75 (d, $J$ = 8.7 Hz, 2H), 6.63 (d, $J$ = 8.7 Hz, 2H), 6.44 (d, $J$ = 16.0 Hz, 1H), 4.27 – 4.14 (m, 4H), 4.07 (t, $J$ = 6.4 Hz, 1H), 3.80 (t, $J$ = 9.4 Hz, 2H), 3.71 (s, 3H), 1.84 – 1.63 (m, 4H), 1.46 – 1.24 (m, 12H).

**$^{13}$C NMR (151 MHz, CDCl$_3$):** $\delta$ 169.0, 167.1, 152.5, 144.6, 141.2, 134.5, 130.2, 128.9, 128.1, 118.3, 115.0, 114.8, 67.7, 64.7, 55.7, 54.2, 52.9, 33.6, 29.4, 29.38, 29.36, 29.2, 28.7, 26.0, 25.7.

**IR (neat, cm$^{-1}$):** 3265, 2920, 2849, 1701, 1632, 1526, 1462, 1238, 1163, 980, 827.

**HRMS (ESI-TOF):** calculated for [$C_{29}H_{39}N_2O_4$ (M + $H^+$)]: 479.2904, found: 479.2908.

**7-(4,5-dihydrooxazol-2-yl)-7-((4-methoxyphenyl)amino)heptyl benzoate (4s):**

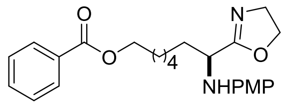


Following a procedure similar to the general procedure wherein $T^1$ = −40 °C, $t^1$ = 3 h and $T^2$ = −50 °C, $t^2$ = 30 min, the title compound was obtained as light yellow solid, m.p. 76 – 77 °C, 124.6 mg, 60% yield. ($R_f$ = 0.14, eluent: PE/EtOAc = 2/1).

**$^{1}$H NMR (600 MHz, CDCl$_3$):** $\delta$ 8.03 (d, $J$ = 7.3 Hz, 2H), 7.56 – 7.51 (m, 1H), 7.45 – 7.40 (m,

2H), 6.75 (d, $J$ = 8.9 Hz, 2H), 6.63 (d, $J$ = 8.9 Hz, 2H), 4.29 (t, $J$ = 6.6 Hz, 2H), 4.26 – 4.17 (m, 2H), 4.08 (t, $J$ = 6.6 Hz, 1H), 3.81 (t, $J$ = 9.3 Hz, 2H), 3.72 (s, 3H), 1.84 – 1.72 (m, 4H), 1.48 – 1.36 (m, 6H).

**$^{13}$C NMR (151 MHz, CDCl$_3$):** $\delta$ 169.0, 166.7, 152.6, 141.1, 132.9, 130.5, 129.6, 128.4, 115.1, 114.9, 67.7, 65.0, 55.7, 54.2, 52.9, 33.5, 29.1, 28.7, 26.0, 25.6.

**IR (neat, cm$^{-1}$):** 3268, 2934, 2854, 1710, 1654, 1510, 1235, 1037, 819, 707.

**HRMS (ESI-TOF):** calculated for [$C_{24}H_{31}N_2O_4$ (M + H$^+$)]: 411.2278, found: 411.2272.

**7-(4,5-dihydrooxazol-2-yl)-7-((4-methoxyphenyl)amino)heptyl 4-bromobenzoate (4t):**

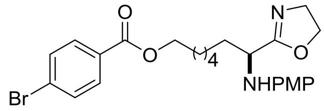


Following the general procedure, the title compound was obtained as light yellow solid, m.p. 116 – 118 °C, 144.7 mg, 59% yield. ($R_f$ = 0.21, eluent: PE/EtOAc = 2/1).

**$^1$H NMR (600 MHz, CDCl$_3$):** $\delta$ 7.88 (d, $J$ = 8.3 Hz, 2H), 7.56 (d, $J$ = 8.4 Hz, 2H), 6.75 (d, $J$ = 8.7 Hz, 2H), 6.63 (d, $J$ = 8.7 Hz, 2H), 4.28 (t, $J$ = 6.6 Hz, 2H), 4.26 – 4.15 (m, 2H), 4.07 (t, $J$ = 6.4 Hz, 1H), 3.81 (t, $J$ = 9.5 Hz, 2H), 3.72 (s, 3H), 1.86 – 1.71 (m, 4H), 1.51 – 1.35 (m, 6H).

**$^{13}$C NMR (151 MHz, CDCl$_3$):** $\delta$ 169.0, 166.0, 152.6, 141.1, 131.8, 131.2, 129.4, 128.0, 115.1, 114.9, 67.8, 65.3, 55.8, 54.2, 52.9, 33.5, 29.1, 28.7, 26.0, 25.6.

**IR (neat, cm$^{-1}$):** 3272, 2934, 2865, 1712, 1653, 1507, 1475, 1103, 1007, 823.

**HRMS (ESI-TOF):** calculated for [$C_{24}H_{30}BrN_2O_4$ (M + H$^+$)]: 489.1383, found: 489.1389.

**7-(4,5-dihydrooxazol-2-yl)-7-((4-methoxyphenyl)amino)heptyl 4-nitrobenzoate (4u):**

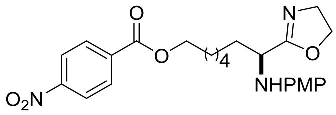


Following a procedure similar to the general procedure wherein $T^1$ = −40 °C, $t^1$ = 3 h and $T^2$ = −50 °C, $t^2$ = 30 min, the title compound was obtained as light yellow solid, m.p. 103 – 106 °C, 129.5 mg, 56% yield. ($R_f$ = 0.23, eluent: PE/EtOAc = 1/1).

**$^1$H NMR (600 MHz, CDCl$_3$):** $\delta$ 8.26 (d, $J$ = 8.7 Hz, 2H), 8.18 (d, $J$ = 8.7 Hz, 2H), 6.74 (d, $J$ = 8.8 Hz, 2H), 6.61 (d, $J$ = 8.8 Hz, 2H), 4.34 (t, $J$ = 6.6 Hz, 2H), 4.26 – 4.17 (m, 2H), 4.07 (t, $J$ = 6.3 Hz, 1H), 3.81 (t, $J$ = 9.4 Hz, 2H), 3.71 (s, 3H), 1.87 – 1.71 (m, 4H), 1.49 – 1.35 (m, 6H).

**$^{13}$C NMR (151 MHz, CDCl$_3$):** $\delta$ 168.9, 164.7, 152.5, 150.5, 141.0, 135.8, 130.7, 123.5, 115.0, 114.8, 67.7, 66.0, 55.7, 54.1, 52.8, 33.4, 29.0, 28.5, 25.9, 25.6.

**IR (neat, cm$^{-1}$):** 3254, 2934, 2852, 1714, 1524, 1350, 1273, 1037, 827, 716.

**HRMS (ESI-TOF):** calculated for [$C_{24}H_{30}N_3O_6$ (M + $H^+$)]: 456.2129, found: 456.2126.

**12-(4,5-dihydrooxazol-2-yl)-12-((4-methoxyphenyl)amino)dodecyl-5-chlorothiophene-2-carboxylate (4v):**

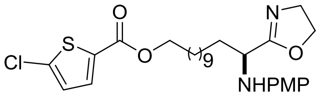


Following the general procedure, the title compound was obtained as light yellow solid, m.p. 80 – 82 °C, 149.8 mg, 58% yield. ($R_f$ = 0.15, eluent: PE/EtOAc = 3/1).

**$^1$H NMR (600 MHz, CDCl$_3$):** $\delta$ 7.57 (d, $J$ = 4.0 Hz, 1H), 6.92 (d, $J$ = 4.0 Hz, 1H), 6.76 (d, $J$ = 8.9 Hz, 2H), 6.63 (d, $J$ = 8.9 Hz, 2H), 4.30 – 4.18 (m, 4H), 4.07 (t, $J$ = 6.7 Hz, 1H), 3.82 (t, $J$ = 9.3 Hz, 2H), 3.73 (s, 3H), 1.82 – 1.68 (m, 4H), 1.48 – 1.35 (m, 4H), 1.33 – 1.23 (m, 12H).

**$^{13}$C NMR (151 MHz, CDCl$_3$):** $\delta$ 169.2, 161.5, 152.6, 141.2, 137.2, 132.9, 132.3, 127.3, 115.1, 114.9, 67.8, 65.9, 55.8, 54.2, 53.1, 33.7, 29.6, 29.59, 29.56, 29.49, 29.3, 28.7, 26.0, 25.8.

**IR (neat, cm$^{-1}$):** 3259, 2916, 2849, 1708, 1655, 1510, 1424, 1235, 1089, 826, 743.

**HRMS (ESI-TOF):** calculated for [$C_{27}H_{38}ClN_2O_4S$ (M + $H^+$)]: 521.2235, found: 521.2244.

***N*-(1-(4,5-dihydrooxazol-2-yl)pentyl)-4-methylaniline (5a):**

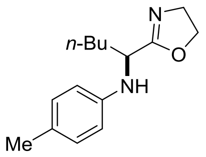


Following the general procedure, the title compound was obtained as light yellow solid, m.p. 82 – 83 °C, 79.3 mg, 64% yield. ($R_f$ = 0.24, eluent: PE/EtOAc = 3/1).

**$^1$H NMR (600 MHz, CDCl$_3$):** $\delta$ 6.98 (d, $J$ = 8.2 Hz, 2H), 6.59 (d, $J$ = 8.4 Hz, 2H), 4.30 – 4.18 (m, 2H), 4.16 – 4.10 (m, 1H), 3.92 (s, 1H), 3.83 (t, $J$ = 9.3 Hz, 2H), 2.23 (s, 3H), 1.89 – 1.71 (m, 2H), 1.45 – 1.30 (m, 4H), 0.90 (t, $J$ = 7.1 Hz, 3H).

**$^{13}$C NMR (151 MHz, CDCl$_3$):** $\delta$ 169.1, 144.8, 129.8, 127.3, 113.7, 67.8, 54.3, 52.3, 33.4, 27.9, 22.6, 20.5, 14.1.

**IR (neat, cm$^{-1}$):** 3279, 2915, 2855, 1657, 1522, 1262, 1183, 980, 951, 807.

**HRMS (ESI-TOF):** calculated for [$C_{15}H_{23}N_2O$ (M + $H^+$)]: 247.1805, found: 247.1810.

**4-(*tert*-butyl)-*N*-(1-(4,5-dihydrooxazol-2-yl)pentyl)aniline (5b):**

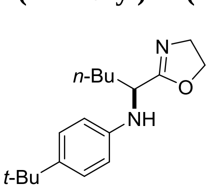


Following the general procedure, the title compound was obtained as light

yellow solid, m.p. 76 – 78 °C, 92.6 mg, 64% yield. ($R_f$ = 0.25, eluent: PE/EtOAc = 3/1).

**$^1$H NMR (600 MHz, CDCl$_3$):** $\delta$ 7.20 (d, $J$ = 8.5 Hz, 2H), 6.63 (d, $J$ = 8.6 Hz, 2H), 4.30 – 4.18 (m, 2H), 4.17 – 4.13 (m, 1H), 4.00 (d, $J$ = 7.3 Hz, 1H), 3.84 (t, $J$ = 9.1 Hz, 2H), 1.90 – 1.68 (m, 2H), 1.45 – 1.34 (m, 4H), 1.28 (s, 9H), 0.91 (t, $J$ = 7.1 Hz, 3H).

**$^{13}$C NMR (151 MHz, CDCl$_3$):** $\delta$ 169.1, 144.7, 140.6, 126.1, 113.1, 67.7, 54.2, 52.0, 33.9, 33.4, 31.6, 27.9, 22.6, 14.1.

**IR (neat, cm$^{-1}$):** 3317, 2949, 2858, 1613, 1523, 1464, 1214, 1156, 982, 813.

**HRMS (ESI-TOF):** calculated for [$C_{18}H_{29}N_2O$ (M + H$^+$)]: 289.2274, found: 289.2265.

***N*-(1-(4,5-dihydrooxazol-2-yl)pentyl)-3-methylaniline (5c):**

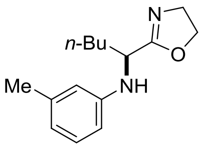


Following the general procedure, the title compound was obtained as light yellow solid, m.p. 60 – 61 °C, 65.2 mg, 53% yield. ($R_f$ = 0.23, eluent: PE/EtOAc = 3/1).

**$^1$H NMR (600 MHz, CDCl$_3$):** $\delta$ 7.08 – 7.03 (m, 1H), 6.54 (d, $J$ = 7.4 Hz, 1H), 6.51 – 6.44 (m, 2H), 4.32 – 4.19 (m, 2H), 4.19 – 4.13 (m, 1H), 4.00 (s, 1H), 3.84 (t, $J$ = 9.2 Hz, 2H), 2.26 (s, 3H), 1.87 – 1.70 (m, 2H), 1.43 – 1.32 (m, 4H), 0.90 (t, $J$ = 7.1 Hz, 3H).

**$^{13}$C NMR (151 MHz, CDCl$_3$):** $\delta$ 169.0, 147.1, 139.1, 129.2, 119.0, 114.4, 110.5, 67.8, 54.3, 51.9, 33.4, 27.9, 22.6, 21.7, 14.1.

**IR (neat, cm$^{-1}$):** 3267, 2937, 2858, 1657, 1587, 1480, 1365, 1225, 1184, 949, 769.

**HRMS (ESI-TOF):** calculated for [$C_{15}H_{23}N_2O$ (M + H$^+$)]: 247.1805, found: 247.1800.

***N*-(1-(4,5-dihydrooxazol-2-yl)pentyl)-3-methoxyaniline (5d):**

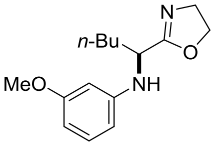


Following the general procedure, the title compound was obtained as light yellow oil, 69.0 mg, 53% yield. ($R_f$ = 0.22, eluent: PE/EtOAc = 2/1).

**$^1$H NMR (600 MHz, CDCl$_3$):** $\delta$ 7.10 – 7.00 (m, 1H), 6.31 – 6.25 (m, 2H), 6.24 – 6.19 (m, 1H), 4.30 – 4.20 (m, 2H), 4.18 – 4.07 (m, 2H), 3.84 (t, $J$ = 9.2 Hz, 2H), 3.75 (s, 3H), 1.89 – 1.70 (m, 2H), 1.43 – 1.31 (m, 4H), 0.90 (t, $J$ = 7.1 Hz, 3H).

**$^{13}$C NMR (151 MHz, CDCl$_3$):** $\delta$ 168.9, 160.8, 148.5, 130.1, 106.4, 103.4, 99.4, 67.9, 55.2, 54.2, 51.9, 33.3, 27.8, 22.6, 14.1.

**IR (neat, cm$^{-1}$):** 3276, 2935, 2867, 1657, 1588, 1493, 1202, 1162, 947, 751.

**HRMS (ESI-TOF):** calculated for [$C_{15}H_{23}N_2O_2$ (M + $H^+$)]: 263.1754, found: 263.1759.

***N*-(1-(4,5-dihydrooxazol-2-yl)pentyl)-3,5-dimethylaniline (5e):**

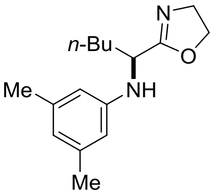


Following the general procedure, the title compound was obtained as light yellow solid, m.p. 87 – 88 °C, 79.5 mg, 61% yield. ($R_f$ = 0.24, eluent: PE/EtOAc = 3/1).

**$^1$H NMR (600 MHz, CDCl$_3$):** $\delta$ 6.39 (s, 1H), 6.31 (s, 2H), 4.32 – 4.20 (m, 2H), 4.20 – 4.14 (m, 1H), 3.98 (s, 1H), 3.84 (t, $J$ = 9.4 Hz, 2H), 2.23 (s, 6H), 1.87 – 1.70 (m, 2H), 1.44 – 1.31 (m, 4H), 0.91 (t, $J$ = 7.1 Hz, 3H).

**$^{13}$C NMR (151 MHz, CDCl$_3$):** $\delta$ 169.0, 147.1, 138.9, 120.0, 111.3, 67.8, 54.2, 51.8, 33.4, 27.8, 22.5, 21.6, 14.1.

**IR (neat, cm$^{-1}$):** 3266, 2955, 2858, 1659, 1599, 1462, 1332, 1186, 947, 855.

**HRMS (ESI-TOF):** calculated for [$C_{16}H_{25}N_2O$ (M + $H^+$)]: 261.1961, found: 261.1956.

***N*-(1-(4,5-dihydrooxazol-2-yl)pentyl)naphthalen-2-amine (5f):**

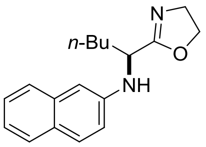


Following the general procedure, the title compound was obtained as light yellow solid, m.p. 112 – 114 °C, 67.2 mg, 45% yield. ($R_f$ = 0.20, eluent: PE/EtOAc = 3/1).

**$^1$H NMR (600 MHz, CDCl$_3$):** $\delta$ 7.67 (d, $J$ = 8.1 Hz, 1H), 7.64 (d, $J$ = 8.6 Hz, 2H), 7.40 – 7.33 (m, 1H), 7.24 – 7.18 (m, 1H), 6.94 (d, $J$ = 8.7 Hz, 1H), 6.91 (s, 1H), 4.35 – 4.19 (m, 4H), 3.85 (t, $J$ = 8.9 Hz, 2H), 1.98 – 1.80 (m, 2H), 1.50 – 1.35 (m, 4H), 0.93 (t, $J$ = 7.2 Hz, 3H).

**$^{13}$C NMR (151 MHz, CDCl$_3$):** $\delta$ 168.8, 144.7, 135.1, 129.1, 127.8, 127.7, 126.4, 126.2, 122.3, 118.2, 105.5, 67.8, 54.2, 51.9, 33.2, 27.8, 22.6, 14.1.

**IR (neat, cm$^{-1}$):** 3269, 2936, 2857, 1655, 1543, 1468, 1225, 950, 833, 742.

**HRMS (ESI-TOF):** calculated for [$C_{18}H_{23}N_2O$ (M + $H^+$)]: 283.1805, found: 283.1804.

***N*-(1-(4,5-dihydrooxazol-2-yl)pentyl)-1H-indol-5-amine (5g):**

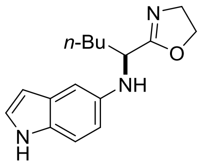


Following the general procedure, the title compound was obtained as light yellow solid, m.p. 107 – 109 °C, 92.2 mg, 63% yield. ($R_f$ = 0.18, eluent:

PE/EtOAc = 1/1).

**$^{1}$H NMR (600 MHz, CDCl$_3$):** $\delta$ 8.24 (s, 1H), 7.15 (d, $J$ = 8.6 Hz, 1H), 7.11 – 7.02 (m, 1H), 6.92 (d, $J$ = 2.1 Hz, 1H), 6.65 (dd, $J$ = 8.6, 2.2 Hz, 1H), 6.44 – 6.30 (m, 1H), 4.28 – 4.13 (m, 3H), 3.90 – 3.73 (m, 3H), 1.90 – 1.78 (m, 2H), 1.51 – 1.34 (m, 4H), 0.92 (t, $J$ = 7.2 Hz, 3H).

**$^{13}$C NMR (151 MHz, CDCl$_3$):** $\delta$ 169.5, 140.9, 130.6, 128.7, 124.6, 112.8, 111.8, 103.7, 101.8, 67.7, 54.2, 53.5, 33.6, 28.1, 22.6, 14.1.

**IR (neat, cm$^{-1}$):** 3243, 2951, 2852, 1655, 1462, 1236, 1161, 1070, 955, 802, 722.

**HRMS (ESI-TOF):** calculated for [$C_{16}H_{22}N_3O$ (M + H$^+$)]: 272.1757, found: 272.1753.

***N*-butyl-1-(4,5-dihydrooxazol-2-yl)pentan-1-amine (5h):**

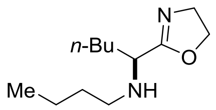


Following a procedure similar to the general procedure wherein 3.0 equiv of *n*-BuNH$_2$ was used in the reaction, the title compound was obtained as light yellow oil, 56.4 mg, 53% yield. ($R_f$ = 0.17, eluent: EtOAc).

**$^{1}$H NMR (600 MHz, CDCl$_3$):** $\delta$ 4.30 – 4.15 (m, 2H), 3.88 – 3.76 (m, 2H), 3.31 (t, $J$ = 6.9 Hz, 1H), 2.60 – 2.42 (m, 2H), 1.67 – 1.55 (m, 3H), 1.47 – 1.34 (m, 2H), 1.35 – 1.19 (m, 6H), 0.92 – 0.78 (m, 6H).

**$^{13}$C NMR (151 MHz, CDCl$_3$):** $\delta$ 169.7, 67.4, 56.7, 54.2, 47.6, 33.4, 32.4, 28.1, 22.7, 20.5, 14.0.

**IR (neat, cm$^{-1}$):** 3304, 2927, 2871, 1720, 1660, 1464, 1153, 979, 947.

**HRMS (ESI-TOF):** calculated for [$C_{12}H_{25}N_2O$ (M + H$^+$)]: 213.1961, found: 213.1962.

**2-(1-(pyrrolidin-1-yl)pentyl)-4,5-dihydrooxazole (5i):**

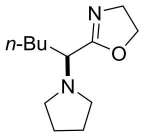


Following the general procedure, the title compound was obtained as light yellow oil, 80.9 mg, 77% yield. ($R_f$ = 0.31, eluent: DCM/MeOH = 15/1).

**$^{1}$H NMR (600 MHz, CDCl$_3$):** $\delta$ 4.29 – 4.16 (m, 2H), 3.90 – 3.76 (m, 2H), 3.16 – 3.15 (m, 1H), 2.72 – 2.59 (m, 2H), 2.58 – 2.46 (m, 2H), 1.82 – 1.67 (m, 6H), 1.35 – 1.20 (m, 4H), 0.87 (t, $J$ = 6.8 Hz, 3H).

**$^{13}$C NMR (151 MHz, CDCl$_3$):** $\delta$ 168.0, 67.2, 62.3, 54.0, 51.3, 315, 28.4, 23.4, 22.8, 14.1.

**IR (neat, cm$^{-1}$):** 2955, 2873, 1653, 1458, 1301, 1231, 1141, 980, 948, 729.

**HRMS (ESI-TOF):** calculated for [$C_{12}H_{23}N_2O$ (M + H$^+$)]: 211.1805, found: 211.1803.

**2-(1-(4-methylpiperidin-1-yl)pentyl)-4,5-dihydrooxazole (5j):**

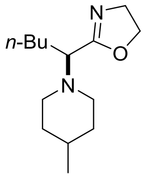

Following the general procedure, the title compound was obtained as light yellow oil, 75.0 mg, 63% yield. ($R_f$ = 0.31, eluent: PE/EtOAc = 1/1).

**$^1$H NMR (600 MHz, CDCl$_3$):** $\delta$ 4.20 (t, $J$ = 9.5 Hz, 2H), 3.88 – 3.76 (m, 2H), 3.26 – 3.16 (m, 1H), 2.86 – 2.77 (m, 2H), 2.26 – 2.16 (m, 1H), 2.11 – 2.02 (m, 1H), 1.76 – 1.60 (m, 2H), 1.60 – 1.51 (m, 2H), 1.32 – 1.09 (m, 7H), 0.89 – 0.74 (m, 6H).

**$^{13}$C NMR (151 MHz, CDCl$_3$):** $\delta$ 167.4, 66.8, 63.1, 54.0, 52.5, 48.4, 34.8, 34.7, 31.1, 29.7, 28.8, 22.7, 22.0, 14.1.

**IR (neat, cm$^{-1}$):** 2922, 2870, 1653, 1456, 1375, 1259, 1149, 1082, 980, 732.

**HRMS (ESI-TOF):** calculated for [$C_{14}H_{27}N_2O$ (M + $H^+$)]: 239.2118, found: 239.2119.

**2-(1-(3,4-dihydroisoquinolin-2(1*H*)-yl)pentyl)-4,5-dihydrooxazole(5k):**

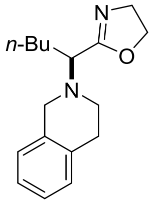

Following the general procedure, the title compound was obtained as light yellow oil, 82.7 mg, 60% yield. ($R_f$ = 0.28, eluent: PE/EtOAc = 3/1).

**$^1$H NMR (600 MHz, CDCl$_3$):** $\delta$ 7.15 – 7.04 (m, 3H), 7.04 – 6.98 (m, 1H), 4.23 (t, $J$ = 9.2 Hz, 2H), 3.92 – 3.73 (m, 4H), 3.49 (t, $J$ = 6.0 Hz, 1H), 3.02 – 2.93 (m, 1H), 2.92 – 2.82 (m, 2H), 2.79 – 2.71 (m, 1H), 1.91 – 1.76 (m, 2H), 1.43 – 1.29 (m, 4H), 0.90 (t, $J$ = 6.8 Hz, 3H).

**$^{13}$C NMR (151 MHz, CDCl$_3$):** $\delta$ 167.0, 135.3, 134.6, 128.8, 126.6, 126.0, 125.6, 66.9, 62.3, 54.1, 52.4, 47.3, 30.0, 29.7, 28.7, 22.7, 14.1.

**IR (neat, cm$^{-1}$):** 2954, 2872, 1653, 1456, 1376, 1142, 1090, 982, 948, 741.

**HRMS (ESI-TOF):** calculated for [$C_{17}H_{25}N_2O$ (M + $H^+$)]: 273.1967, found: 273.1960

**4-(1-(4,5-dihydrooxazol-2-yl)pentyl)morpholine (5l):**

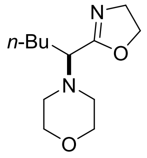

Following the general procedure, the title compound was obtained as light yellow oil, 73.5 mg, 65% yield. ($R_f$ = 0.17, eluent: EtOAc).

**$^1$H NMR (600 MHz, CDCl$_3$):** $\delta$ 4.22 (t, $J$ = 9.6 Hz, 2H), 3.87 – 3.79 (m, 2H), 3.74 – 3.59 (m, 4H), 3.25 – 3.17 (m, 1H), 2.62 – 2.48 (m, 4H), 1.74 – 1.61 (m, 2H), 1.34 – 1.20 (m,

4H), 0.86 (t, *J* = 7.0 Hz, 3H).

**$^{13}$C NMR (151 MHz, $CDCl_3$):** $\delta$ 166.8, 67.4, 67.0, 63.0, 54.0, 50.3, 29.1, 28.5, 22.7, 14.1.

**IR (neat, cm$^{-1}$):** 2954, 2854, 1653, 1452, 1351, 1261, 1114, 982, 949, 856.

**HRMS (ESI-TOF):** calculated for [$C_{12}H_{23}N_2O_2$ (M + $H^+$)]: 227.1754, found: 227.1750.

**2-(1-thiomorpholinopentyl)-4,5-dihydrooxazole (5m):**

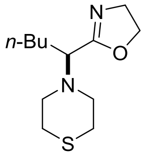


Following the general procedure, the title compound was obtained as light yellow oil, 81.9 mg, 68% yield. ($R_f$ = 0.23, eluent: PE/EtOAc = 1/1).

**$^{1}$H NMR (400 MHz, $CDCl_3$):** $\delta$ 4.20 (t, *J* = 9.6 Hz, 2H), 3.81 (t, *J* = 9.9 Hz, 2H), 3.18 (t, *J* = 7.6 Hz, 1H), 2.95 – 2.84 (m, 2H), 2.78 – 2.67 (m, 2H), 2.66 – 2.53 (m, 4H), 1.72 – 1.55 (m, 2H), 1.30 – 1.18 (m, 4H), 0.84 (t, *J* = 6.9 Hz, 3H).

**$^{13}$C NMR (101 MHz, $CDCl_3$):** $\delta$ 166.8, 66.8, 63.6, 54.0, 52.1, 29.2, 28.6, 28.5, 22.5, 14.1.

**IR (neat, cm$^{-1}$):** 2954, 2871, 1653, 1455, 1330, 1278, 1131, 986, 945, 731.

**HRMS (ESI-TOF):** calculated for [$C_{12}H_{23}N_2OS$ (M + $H^+$)]: 243.1526, found: 243.1525.

***Tert*-butyl-4-(1-(4,5-dihydrooxazol-2-yl)pentyl)piperazine-1-carboxylate (5n):**

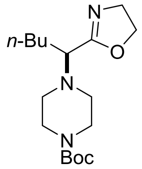


Following the general procedure, the title compound was obtained as light yellow oil, 91.4 mg, 56% yield. ($R_f$ = 0.17, eluent: PE/EtOAc = 2/1).

**$^{1}$H NMR (400 MHz, $CDCl_3$):** $\delta$ 4.23 – 4.13 (m, 2H), 3.84 – 3.79 (m, 2H), 3.45 – 3.31 (m, 4H), 3.27 – 3.23 (m, 1H), 2.53 – 2.52 (m, 2H), 2.46 – 2.41 (m, 2H), 1.74 – 1.58 (m, 2H), 1.40 (s, 9H), 1.34 – 1.24 (m, 4H), 0.85 (t, *J* = 6.9 Hz, 3H).

**$^{13}$C NMR (101 MHz, $CDCl_3$)** $\delta$ 166.7, 154.6, 79.4, 66.78, 62.47, 53.8, 49.4, 29.2, 28.4, 28.3, 22.4, 13.9.

**IR (neat, cm$^{-1}$):** 2957, 2680, 1690, 1655, 1456, 1365, 731.

**HRMS (ESI-TOF):** calculated for [$C_{17}H_{32}N_3O_3$ (M + $H^+$)]: 326.2444, found: 326.2438.

***N*-(1-(4,5-dihydrooxazol-2-yl)pentyl)-4-methoxy-N-methylaniline (5o):**

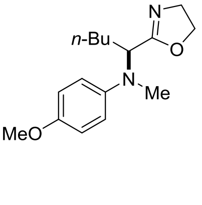


Following the general procedure, the title compound was obtained as light yellow oil, 87.1 mg, 63% yield. ($R_f$ = 0.17, eluent: EtOAc).

**$^{1}$H NMR (400 MHz, CDCl$_3$):** $\delta$ 6.84 – 6.77 (m, 4H), 4.31 – 4.25 (m, 1H), 4.22 – 4.15 (m, 2H), 3.83 (t, $J$ = 9.5 Hz, 2H), 3.74 (s, 3H), 2.79 (s, 3H), 1.93 – 1.80 (m, 2H), 1.40 – 1.29 (m, 4H), 0.89 – 0.86 (m, 3H).

**$^{13}$C NMR (101 MHz, CDCl$_3$)**: $\delta$ 167.9, 152.0, 144.8, 115.5, 114.5, 67.1, 58.0, 55.5, 54.1, 32.8, 29.8, 28.5, 22.4, 13.9.

**IR (neat, cm$^{-1}$):** 2954, 1658, 1511, 1465, 1244, 1038, 955, 816.

**HRMS (ESI-TOF):** calculated for [$C_{16}H_{25}N_2O_2$ (M + H$^+$)]: 277.1916, found: 277.1911.

**2-(1-(*p*-tolylthio)pentyl)-4,5-dihydrooxazole (5p):**

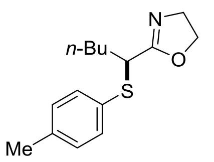


Following the general procedure, the title compound was obtained as light yellow oil, 88.4 mg, 67% yield. ($R_f$ = 0.28, eluent: PE/EtOAc = 3/1).

**$^{1}$H NMR (600 MHz, CDCl$_3$):** $\delta$ 7.33 (d, $J$ = 8.0 Hz, 2H), 7.09 (d, $J$ = 7.9 Hz, 2H), 4.23 (t, $J$ = 9.5 Hz, 2H), 3.80 – 3.74 (m, 2H), 3.72 (t, $J$ = 7.6 Hz, 1H), 2.31 (s, 3H), 1.92 – 1.70 (m, 2H), 1.49 – 1.34 (m, 2H), 1.34 – 1.26 (m, 2H), 0.87 (t, $J$ = 7.2 Hz, 3H).

**$^{13}$C NMR (151 MHz, CDCl$_3$):** $\delta$ 167.5, 138.0, 133.3, 129.9, 129.7, 67.7, 54.4, 46.2, 31.9, 29.5, 22.3, 21.2, 14.0.

**IR (neat, cm$^{-1}$):** 2953, 2870, 1656, 1491, 1242, 1161, 982, 951, 808.

**HRMS (ESI-TOF):** calculated for [$C_{15}H_{22}NOS$ (M + H$^+$)]: 264.1417, found: 264.1420.

**2-(1-((4-methoxyphenyl)thio)pentyl)-4,5-dihydrooxazole (5q):**

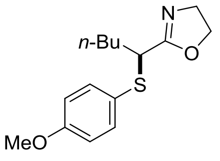


Following the general procedure, the title compound was obtained as light yellow oil, 95.2 mg, 68% yield. ($R_f$ = 0.15, eluent: PE/EtOAc = 3/1).

**$^{1}$H NMR (600 MHz, CDCl$_3$):** $\delta$ 7.38 (d, $J$ = 8.8 Hz, 2H), 6.81 (d, $J$ = 8.8 Hz, 2H), 4.22 (t, $J$ = 9.5 Hz, 2H), 3.77 (s, 3H), 3.76 – 3.71 (m, 2H), 3.63 (t, $J$ = 7.5 Hz, 1H), 1.87 – 1.68 (m, 2H), 1.49 – 1.33 (m, 2H), 1.33 – 1.27 (m, 2H), 0.87 (t, $J$ = 7.2 Hz, 3H).

**$^{13}$C NMR (151 MHz, CDCl$_3$):** $\delta$ 167.5, 160.0, 136.2, 123.6, 114.4, 67.6, 55.4, 54.4, 46.9, 31.8, 29.5, 22.4, 14.0.

**IR (neat, cm$^{-1}$):** 2954, 2871, 1655, 1590, 1491, 1241, 1170, 1028, 982, 827.

**HRMS (ESI-TOF):** calculated for [$C_{15}H_{22}NO_2S$ (M + H$^+$)]: 280.1366, found: 280.1370.

**2-(1-((4-bromophenyl)thio)pentyl)-4,5-dihydrooxazole (5r):**

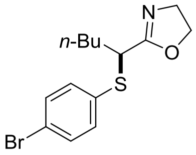


Following the general procedure, the title compound was obtained as light yellow oil, 110.1 mg, 67% yield. ($R_f$ = 0.24, eluent: PE/EtOAc = 5/1).

**$^1$H NMR (600 MHz, CDCl$_3$):** $\delta$ 7.40 (d, $J$ = 8.6 Hz, 2H), 7.31 (d, $J$ = 8.6 Hz, 2H), 4.30 – 4.17 (m, 2H), 3.86 – 3.72 (m, 3H), 1.96 – 1.82 (m, 1H), 1.82 – 1.74 (m, 1H), 1.49 – 1.29 (m, 4H), 0.89 (t, $J$ = 7.2 Hz, 3H).

**$^{13}$C NMR (151 MHz, CDCl$_3$):** $\delta$ 167.3, 133.9, 133.3, 132.0, 121.9, 67.8, 54.4, 45.8, 32.0, 29.5, 22.4, 14.0.

**IR (neat, cm$^{-1}$):** 2951, 2869, 1724, 1653, 1473, 1384, 1238, 1090, 948, 522.

**HRMS (ESI-TOF):** calculated for [$C_{14}H_{19}BrNO$ (M + H$^+$)]: 328.0365, found: 328.0357.

**2-(1-(*m*-tolylthio)pentyl)-4,5-dihydrooxazole (5s):**

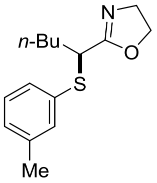


Following the general procedure, the title compound was obtained as light yellow oil, 77.4 mg, 59% yield. ($R_f$ = 0.23, eluent: PE/EtOAc = 5/1).

**$^1$H NMR (600 MHz, CDCl$_3$):** δ 7.33 – 7.22 (m, 2H), 7.21 – 7.11 (m, 1H), 7.05 (d, $J$ = 7.5 Hz, 1H), 4.24 (t, $J$ = 9.5 Hz, 2H), 3.86 – 3.66 (m, 3H), 2.31 (s, 3H), 1.93 – 1.74 (m, 2H), 1.50 – 1.29 (m, 4H), 0.88 (t, $J$ = 7.2 Hz, 3H).

**$^{13}$C NMR (151 MHz, CDCl$_3$):** δ 167.6, 138.6, 133.8, 133.1, 129.4, 128.7, 128.5, 67.7, 54.4, 45.8, 32.1, 29.5, 22.4, 21.4, 14.0.

**IR (neat, cm$^{-1}$):** 2954, 2871, 1656, 1590, 1474, 1355, 1241, 1160, 982, 775.

**HRMS (ESI-TOF):** calculated for [$C_{15}H_{22}NOS$ (M + H$^+$)]: 264.1417, found: 264.1421.

**2-(1-(naphthalen-2-ylthio)pentyl)-4,5-dihydrooxazole (5t):**

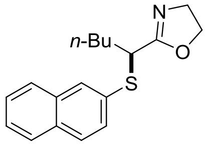


Following the general procedure, the title compound was obtained as light yellow oil, 74.9 mg, 50% yield. ($R_f$ = 0.30, eluent: PE/EtOAc = 3/1).

**$^1$H NMR (600 MHz, CDCl$_3$):** $\delta$ 7.95 (s, 1H), 7.85 – 7.72 (m, 3H), 7.56 – 7.41 (m, 3H), 4.24 (t, $J$ = 9.2 Hz, 2H), 3.98 – 3.92 (m, 1H), 3.82 – 3.71 (m, 2H), 1.99 – 1.81 (m, 2H), 1.52 – 1.32 (m, 4H), 0.90 (t, $J$ = 7.3 Hz, 3H).

**$^{13}$C NMR (151 MHz, CDCl$_3$):** $\delta$ 167.6, 133.7, 132.5, 131.6, 131.1, 129.6, 128.5, 127.8, 127.6, 126.6, 126.3, 67.8, 54.5, 45.8, 32.2, 29.6, 22.4, 14.0.

**IR (neat, cm$^{-1}$):** 2953, 2857, 1655, 1499, 1354, 1241, 1159, 982, 812, 742.

**HRMS (ESI-TOF):** calculated for [$C_{18}H_{22}NOS$ (M + $H^+$)]: 300.1417, found: 300.1422.

**2-(1-(thiophen-2-ylthio)pentyl)-4,5-dihydrooxazole (5u):**

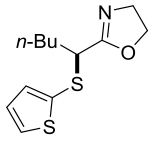

Following the general procedure, the title compound was obtained as light yellow oil, 64.5 mg, 51% yield. ($R_f$ = 0.28, eluent: PE/EtOAc = 2/1).

**$^1$H NMR (600 MHz, CDCl$_3$):** $\delta$ 7.39 (d, $J$ = 5.3 Hz, 1H), 7.16 (d, $J$ = 3.5 Hz, 1H), 7.04 – 6.94 (m, 1H), 4.27 (t, $J$ = 9.5 Hz, 2H), 3.79 (t, $J$ = 9.5 Hz, 2H), 3.60 (t, $J$ = 7.5 Hz, 1H), 1.93 – 1.81 (m, 1H), 1.81 – 1.73 (m, 1H), 1.51 – 1.29 (m, 4H), 0.89 (t, $J$ = 7.2 Hz, 3H).

**$^{13}$C NMR (151 MHz, CDCl$_3$):** $\delta$ 166.9, 136.4, 131.1, 130.8, 127.6, 67.8, 54.6, 48.5, 31.4, 29.4, 22.4, 14.0.

**IR (neat, cm$^{-1}$):** 2953, 2870, 1655, 1464, 1215, 1163, 1104, 982, 847, 700.

**HRMS (ESI-TOF):** calculated for [$C_{12}H_{18}NOS_2$ (M + $H^+$)]: 256.0824, found: 256.0816.

**2-(1-(benzylthio)pentyl)-4,5-dihydrooxazole (5v):**

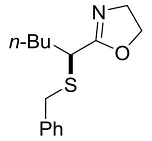

Following the general procedure, the title compound was obtained as light yellow oil, 86.8 mg, 60% yield. ($R_f$ = 0.20, eluent: PE/EtOAc = 5/1).

**$^1$H NMR (600 MHz, CDCl$_3$):** $\delta$ 7.34 (d, $J$ = 7.4 Hz, 2H), 7.32 – 7.27 (m, 2H), 7.24 – 7.20 (m, 1H), 4.23 – 4.15 (m, 2H), 3.82 – 3.73 (m, 4H), 3.37 (t, $J$ = 7.6 Hz, 1H), 1.86 – 1.77 (m, 1H), 1.70 – 1.60 (m, 1H), 1.38 – 1.20 (m, 4H), 0.84 (t, $J$ = 7.1 Hz, 3H).

**$^{13}$C NMR (151 MHz, CDCl$_3$):** $\delta$ 167.8, 138.2, 129.1, 128.5, 127.0, 67.6, 54.4, 41.6, 35.9, 31.4, 29.5, 22.3, 14.0.

**IR (neat, cm$^{-1}$):** 2953, 2870, 1725, 1653, 1452, 1242, 983, 951, 699.

**HRMS (ESI-TOF):** calculated for [$C_{15}H_{22}NOS$ (M + $H^+$)]: 264.1417, found: 264.1432.

**2-(1-(hexylthio)pentyl)-4,5-dihydrooxazole (5w):**

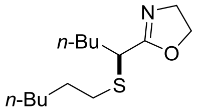

Following the general procedure, the title compound was obtained as light yellow oil, 80.8 mg, 62% yield. ($R_f$ = 0.31, eluent: PE/EtOAc = 5/1).

**$^1$H NMR (600 MHz, CDCl$_3$):** $\delta$ 4.31 – 4.18 (m, 2H), 3.81 (t, $J$ = 9.5 Hz, 2H), 3.37 (t, $J$ = 7.6 Hz, 1H), 2.61 – 2.44 (m, 2H), 1.86 – 1.60 (m, 2H), 1.58 – 1.45 (m, 2H), 1.43 – 1.14 (m, 10H), 0.89 –

0.75 (m, 6H).

**$^{13}$C NMR (151 MHz, CDCl$_3$):** $\delta$ 168.1, 67.6, 54.3, 42.0, 31.7, 31.44, 31.41, 29.6, 29.4, 28.6, 22.6, 22.3, 14.1, 13.9.

**IR (neat, cm$^{-1}$):** 2926, 2857, 1654, 1458, 1355, 1243, 1163, 984, 951, 732.

**HRMS (ESI-TOF):** calculated for [$C_{14}H_{28}NOS$ (M + H$^+$)]: 258.1886, found: 258.1893.

**2-(1-(phenethylthio)pentyl)-4,5-dihydrooxazole (5x):**

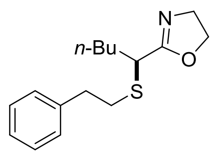


Following the general procedure, the title compound was obtained as light yellow oil, 83.5 mg, 60% yield. ($R_f$ = 0.27, eluent: PE/EtOAc = 5/1).

**$^1$H NMR (600 MHz, CDCl$_3$):** $\delta$ 7.32 – 7.24 (m, 2H), 7.23 – 7.15 (m, 3H), 4.34 – 4.21 (m, 2H), 3.85 (t, $J$ = 9.5 Hz, 2H), 3.50 – 3.43 (m, 1H), 2.94 – 2.75 (m, 4H), 1.90 – 1.79 (m, 1H), 1.74 – 1.65 (m, 1H), 1.46 – 1.29 (m, 4H), 0.89 (t, $J$ = 7.1 Hz, 3H).

**$^{13}$C NMR (151 MHz, CDCl$_3$):** $\delta$ 168.0, 140.5, 128.6, 128.5, 126.4, 67.7, 54.3, 42.1, 36.2, 32.9, 31.7, 29.7, 22.4, 14.0.

**IR (neat, cm$^{-1}$):** 3026, 2954, 2858, 1653, 1603, 1496, 1244, 1163, 983, 733.

**HRMS (ESI-TOF):** calculated for [$C_{16}H_{24}NOS$ (M + H$^+$)]: 278.1573, found: 278.1571.

**Methyl-3-((1-(4,5-dihydrooxazol-2-yl)pentyl)thio)propanoate (5y):**

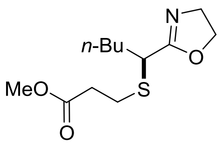


Following the general procedure, the title compound was obtained as light yellow oil, 101.1 mg, 78% yield. ($R_f$ = 0.21, eluent: PE/EtOAc = 2/1).

**$^1$H NMR (600 MHz, CDCl$_3$):** $\delta$ 4.34 – 4.22 (m, 2H), 3.83 (t, $J$ = 9.5 Hz, 2H), 3.65 (s, 3H), 3.46 – 3.38 (m, 1H), 2.88 – 2.75 (m, 2H), 2.64 – 2.53 (m, 2H), 1.86 – 1.77 (m, 1H), 1.71 – 1.62 (m, 1H), 1.42 – 1.26 (m, 4H), 0.86 (t, $J$ = 7.1 Hz, 3H).

**$^{13}$C NMR (151 MHz, CDCl$_3$):** $\delta$ 172.3, 167.8, 67.8, 54.3, 51.8, 42.0, 34.5, 31.6, 29.6, 26.3, 22.3, 13.9.

**IR (neat, cm$^{-1}$):** 2954, 2873, 1735, 1654, 1436, 1357, 1244, 1167, 982, 951.

**HRMS (ESI-TOF):** calculated for [$C_{12}H_{22}NO_3S$ (M + H$^+$)]: 260.1315, found: 260.1313.

**2-(1-(4-methoxyphenoxy)pentyl)-4,5-dihydrooxazole (5z):**

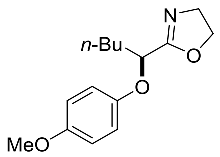


Following the general procedure, the title compound was obtained as light yellow oil, 62.6 mg, 48% yield. ($R_f$ = 0.20, eluent: PE/EtOAc = 2/1).

**$^1$H NMR (600 MHz, CDCl$_3$):** $\delta$ 6.92 (d, $J$ = 9.1 Hz, 2H), 6.79 (d, $J$ = 9.1 Hz, 2H), 4.73 – 4.69 (m, 1H), 4.34 – 4.18 (m, 2H), 3.92 – 3.81 (m, 2H), 3.75 (s, 3H), 2.01 – 1.81 (m, 2H), 1.58 – 1.32 (m, 4H), 0.91 (t, $J$ = 7.2 Hz, 3H).

**$^{13}$C NMR (151 MHz, CDCl$_3$):** $\delta$ 167.2, 154.4, 152.3, 116.8, 114.7, 74.6, 67.8, 55.7, 54.4, 33.1, 27.5, 22.5, 14.1.

**IR (neat, cm$^{-1}$):** 2953, 2871, 1663, 1504, 1464, 1213, 1180, 1035, 954, 823.

**HRMS (ESI-TOF):** calculated for [$C_{15}H_{22}NO_3$ (M + H$^+$)]: 264.1594, found: 264.1583.

**Dibenzyl-2-(1-(4,5-dihydrooxazol-2-yl)pentyl)malonate (5a'):**

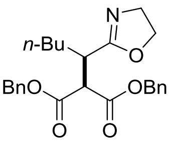


Following the general procedure, the title compound was obtained as light yellow oil, 152.2 mg, 68% yield. ($R_f$ = 0.22, eluent: PE/EtOAc = 3/1). (Dibenzyl malonate is pretreated by NaH as a nucleophile).

**$^1$H NMR (600 MHz, CDCl$_3$):** $\delta$ 7.39 – 7.27 (m, 10H), 5.23 – 5.08 (m, 4H), 4.12 – 4.00 (m, 2H), 3.87 (d, $J$ = 10.4 Hz, 1H), 3.75 – 3.57 (m, 2H), 3.25 – 3.16 (m, 1H), 1.57 – 1.46 (m, 2H), 1.30 – 1.17 (m, 4H), 0.81 (t, $J$ = 6.9 Hz, 3H).

**$^{13}$C NMR (151 MHz, CDCl$_3$):** $\delta$ 168.2, 167.7, 167.6, 135.5, 135.3, 128.6, 128.5, 128.4, 128.34, 128.32, 67.4, 67.3, 67.2, 54.7, 54.2, 38.5, 30.2, 28.7, 22.5, 13.9.

**IR (neat, cm$^{-1}$):** 3032, 2955, 2871, 1731, 1663, 1454, 1214, 1154, 981, 732.

**HRMS (ESI-TOF):** calculated for [$C_{25}H_{30}NO_5$ (M + H$^+$)]: 424.2118, found: 424.2127.

**5-chloro-N2-(4-(1-(1-(4,5-dihydrooxazol-2-yl)pentyl)piperidin-4-yl)-2-isopropoxy-5-methylphenyl)-N4-(2-(isopropylsulfonyl)phenyl)pyrimidine-2,4-diamine (5b'):**

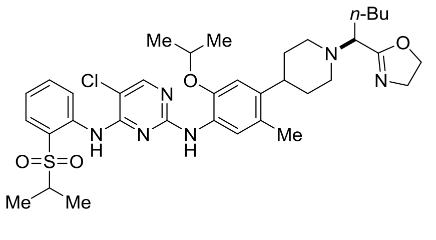


Following the general procedure, the title compound was obtained as light yellow oil, 226.3 mg, 65% yield. ($R_f$ = 0.25, eluent: PE/EtOAc = 1/1).

**$^1$H NMR (400 MHz, CDCl$_3$):** $\delta$ 9.44 (s, 1H), 8.50 (d, $J$ = 8.3 Hz, 1H), 8.08 (s, 1H), 7.87 – 7.82 (m, 2H), 7.54 – 7.51 (m, 2H), 7.20 – 7.15 (m, 1H), 6.78 (s, 1H), 4.54 – 4.48 (m, 1H), 4.38 – 4.31 (m, 2H), 3.92 – 3.86 (m, 2H), 3.53 – 3.39 (m, 1H), 3.21 –

3.15 (m, 1H), 3.08 – 3.06 (m, 2H), 2.63 – 2.58 (m, 1H), 2.41 – 2.36 (m, 2H), 2.07 (s, 3H), 1.86 – 1.68 (m, 6H), 1.28 – 1.22 (m, 16H), 0.84 (t, *J* = 8.0 Hz, 3H).

**$^{13}$C NMR (101 MHz, CDCl$_3$):** $\delta$ 157.4, 155.2, 155.1, 144.8, 138.3, 134.6, 131.1, 127.1, 126.5, 124.6, 123.5, 123.0, 120.7, 110.8, 105.5, 71.2, 67.9, 63.0, 55.3, 53.0, 52.4, 49.9, 37.8, 32.7, 28.8, 28.6, 22.5, 22.1, 18.9, 15.2, 13.9.

**IR (neat, cm$^{-1}$):** 2961, 2358, 1598, 1560, 1507, 1447， 1260, 1093, 1031, 866, 798， 776.

**HRMS (ESI-TOF):** calculated for [$C_{36}H_{50}ClN_6O_4S$ (M + H$^+$)]: 697.3303, found: 697.3312.

# 5. Gram-scale synthesis, elaboration of products, intramolecualr reactions and asymmetric version.

### 5.1 Gram-scale synthesis:

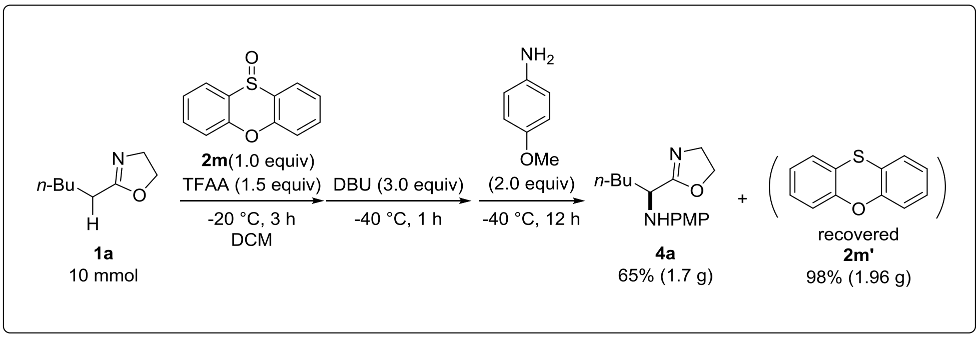


To a mixture of 2-oxazoline **1a** (1.41 g, 10 mmol) and sulfoxide **2m** (2.16 g, 1.0 equiv) in DCM (100 mL) was added TFAA (2.1 mL, 1.5 equiv) at –20 $^{o}$C slowly. The mixture was stirred at –20 $^{o}$C for 3 h. After that, to the mixture was added a solution of DBU (4.57 g, 3.0 equiv) in DCM (20 mL) dropwise in 10 min using syringe pumpat –40 $^{o}$C. The mixture was then stirred for another 1 h. Afterwards, to the mixture was added a solution of *p*-methoxyaniline (2.46 g, 2.0 equiv) in DCM (20 mL) dropwise in 10 min using syringe pumpat. After stirring for 12 h, the mixture was then treated with saturated aqueous $NaHCO_3$ solution and extracted with DCM. The organic layer was dried over $Na_2SO_4$ and concentrated. The obtained residue was purified by Biotage automatic flash system to afford the title compound **4a** (1.7 g, 65%) and phenoxathiine **2m'** as recovered product (1.96 g, 98%).

### 5.2 Elaboration of products to methyl esters:

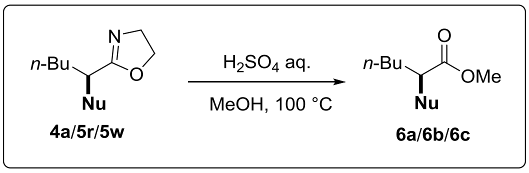


**Methyl-2-((4-methoxyphenyl)amino)hexanoate (6a):**

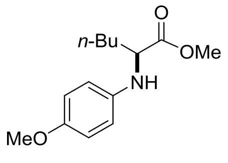


To a solution of **4a** (131 mg, 0.5 mmol) in MeOH (2 mL) was added $H_2SO_4$ (3 M, 1 mL). After refluxing 12 h, the mixture was diluted with DCM and brought to basic by $NaHCO_3$ aqueous solution and the resulting

suspension was extracted with DCM. The organic layer was dried over $Na_2SO_4$ and concentrated. The obtained residue was purified by silica gel column chromatography to afford the title compound as brown oil, 104.3 mg, 83% yield. ($R_f$ = 0.3, eluent: PE/EtOAc = 15/1).

**$^1$H NMR (400 MHz, CDCl$_3$):** $\delta$ 6.76 (d, $J$ = 8.8 Hz, 2H), 6.59 (d, $J$ = 8.8 Hz, 2H), 3.97 (t, $J$ = 6.5 Hz, 1H), 3.73 (s, 3H), 3.70 (s, 3H), 1.85 – 1.63 (m, 2H), 1.44 – 1.30 (m, 4H), 0.91 (t, $J$ = 6.9 Hz, 3H).

**$^{13}$C NMR (101 MHz, CDCl$_3$):** $\delta$ 175.2, 152.7, 141.0, 115.0, 114.9, 57.8, 55.7, 52.0, 33.0, 27.9, 22.5, 14.0。

**IR (neat, cm$^{-1}$):** 3375, 2954, 2860, 1735, 1510, 1464, 1235, 1152, 820。

**HRMS (ESI-TOF):** calculated for [$C_{14}H_{22}NO_3$ (M + H$^+$)]: 252.1600, found: 252.1594.

**Methyl-2-((4-bromophenyl)thio)hexanoate (6b):**

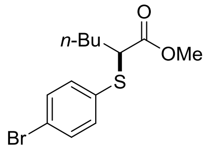


Following the procedure used for the synthesis of **6a**, the title compound **6b** was prepared from from **5r** as light yellow oil, 126.9 mg, 80% yield. ($R_f$ = 0.25, eluent: PE/EtOAc = 15/1).

**$^1$H NMR (400 MHz, CDCl$_3$):** $\delta$ 7.42 – 7.40 (m, 2H), 7.30 – 7.26 (m, 2H), 3.66 (s, 3H), 3.62 – 3.58 (m, 1H), 1.91 – 1.82 (m, 1H), 1.77 – 1.62 (m, 1H), 1.45 – 1.24 (m, 4H), 0.88 (t, $J$ = 7.0 Hz 3H).

**$^{13}$C NMR (101 MHz, CDCl$_3$):** $\delta$ 172.5, 134.2, 132.6, 132.0, 122.2, 52.2, 50.6, 31.2, 29.3, 22.2, 13.8; **IR (neat, cm$^{-1}$):** 2953, 2929, 1732, 1566, 1473, 1260, 1154, 815;.

**HRMS (ESI-TOF):** calculated for [$C_{13}H_{18}BrO_2S$ (M + H$^+$)]: 317.0211, 319.0190, found: 317.0205, 319.0185.

**Methyl-2-(hexylthio)hexanoate (6c):**

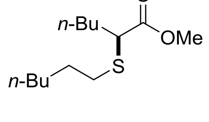


Following the procedure used for the synthesis of **6a**, the title compound **6c** was prepared from **5w** as light yellow oil, 106 mg, 86% yield. ($R_f$ = 0.3, eluent: DCM/MeOH = 20/1).

**$^1$H NMR (400 MHz, CDCl$_3$):** $\delta$ 3.70 (s, 3H), 3.22 – 3.18 (m, 1H), 2.60 – 2.49 (m, 2H), 1.88 – 1.79 (m, 1H), 1.67 – 1.45 (m, 3H), 1.39 – 1.22 (m, 10H), 0.86 (q, $J$ = 6.8 Hz, 6H).

**$^{13}$C NMR (101 MHz, CDCl$_3$):** $\delta$ 173.4, 52.0, 46.5, 31.3, 31.2, 31.1, 29.5, 29.2, 28.4, 22.4, 22.2,

13.9, 13.8.

**IR (neat, cm$^{-1}$):** 2955, 2926, 1730, 1458, 1259, 1153.

**HRMS (ESI-TOF):** calculated for [$C_{13}H_{27}O_2S$ (M + $H^+$)]: 247.1732, found: 247.1726.

**5.3 Intramolecualr reactions:**

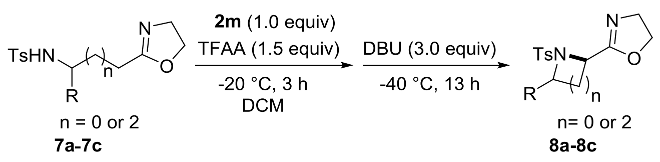


**2-(3-methyl-1-tosylaziridin-2-yl)-4,5-dihydrooxazole (8a):**

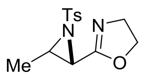


To a mixture of 2-oxazoline **7a** (140 mg, 0.5 mmol) and sulfoxide **2m** (108.0 mg, 1.0 equiv) in DCM (5 mL) was added TFAA (104 µL, 1.5 equiv) at -20 °C. After stirring for 3 h, the reaction mixture was then cooled to -40 °C. To the mixture was added a solution of DBU (228.4 mg, 3.0 equiv) in DCM (1 mL) dropwise in 10 min using syringe pump. The mixture was then stirred for 13 h. After that, the mixture was treated with saturated aqueous $NaHCO_3$ solution and extracted with DCM. The organic layer was dried over $Na_2SO_4$ and concentrated. The obtained residue was purified by silica gel column chromatography to afford the title compound **8a** as lightyellow oil, 89.7 mg, 64% yield (dr: 66/34) ($R_f$= 0.20, eluent: PE/EtOAc = 2/1).

**$^1$H NMR (600 MHz, CDCl$_3$):** $\delta$ 7.82 (d, $J$ = 8.3 Hz, 2H), 7.32 – 7.28 (m, 2H), 4.26 – 4.18 (m, 2H), 3.85 – 3.78 (m, 2H), 3.48 – 3.47 (m, 1H), 3.18 – 3.15 (m, 0.32 H), 3.06 – 3.02 (m, 0.67 H), 2.41 (d, $J$ = 7.3 Hz, 3 H), 1.70 (d, $J$ = 6.0 Hz, 1H), 1.31 (d, $J$ = 5.8 Hz, 2 H).

**$^{13}$C NMR (151 MHz, CDCl$_3$)**: $\delta$ 162.51, 161.49, 144.9, 144.3, 137.1, 134.4, 129.8, 129.6, 128.0, 127.5, 68.01, 67.95, 54.59, 54.57, 44.2, 41.4, 40.2, 38.5, 21.7, 21.6, 13.6, 12.5.

**IR (neat, cm$^{-1}$):** 2933, 1663, 1597, 1449, 1325, 1159, 1090, 956, 901, 815.

**HRMS (ESI-TOF):** calculated for [$C_{13}H_{17}N_2O_3S$ (M + $H^+$)]: 281.0960, found: 281.0957.

**2-(3-phenyl-1-tosylaziridin-2-yl)-4,5-dihydrooxazole (8b):**

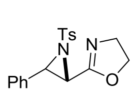


Following the procedure used for the synthesis of **8a**, the title compound **8b** was

prepared from **7b** as light yellow oil, 104.4 mg, 61% yield (dr: 58/42) ($R_f$ = 0.25, eluent: PE/EtOAc = 1/1).

**$^{1}$H NMR (400 MHz, CDCl$_3$):** $\delta$ 7.84 (d, $J$ = 8.2 Hz, 2H), 7.69 (d, $J$ = 8.2 Hz, 1H), 7.27 – 7.13 (m, 12H), 4.41 – 4.22 (m, 2H), 4.04 – 4.00 (m, 1H), 3.97 – 3.78 (m, 3H), 3.74 (d, $J$ = 7.3 Hz, 1H), 3.63 – 3.52 (m, 2H), 2.34 (s, 2.89H), 2.32 (s, 2.10H).

**$^{13}$C NMR (101 MHz, CDCl$_3$):** $\delta$ 161.1, 160.3, 145.0, 144.2, 136.7, 133.8, 132.7, 131.3, 129.8, 129.4, 128.7, 128.4, 128.2, 128.04, 127.96, 127.4, 127.2, 68.3, 67.7, 54.6, 54.2, 46.8, 45.1, 43.5, 40.1, 21.6, 21.5.

**IR (neat, cm$^{-1}$):** 3055, 1664, 1598, 1421, 1332, 1162, 1092, 815, 730, 700.

**HRMS (ESI-TOF):** calculated for [$C_{18}H_{19}N_2O_3S$ (M + H$^+$)]: 343.1116, found: 343.1111.

**2-(1-tosylpyrrolidin-2-yl)-4,5-dihydrooxazole (8c):**

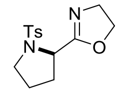


Following the procedure used for the synthesis of **8a**, the title compound **8c** was prepared from **7c** as light yellow oil, 58.8 mg, 40% yield. ($R_f$ = 0.30, eluent: PE/EtOAc = 1/2).

**$^{1}$H NMR (400 MHz, CDCl$_3$):** $\delta$ 7.74 (d, $J$ = 8.2 Hz, 2H), 7.30 (d, $J$ = 8.0 Hz, 2H), 4.40 – 4.37 (m, 1H), 4.27 – 4.21 (m, 1H), 4.17 – 4.10 (m, 1H), 3.87 – 3.73 (m, 1H), 3.55 – 3.48 (m, 1H), 3.34 – 3.28 (m, 1H), 2.41 (s, 3H), 2.04 – 1.87 (m, 3H), 1.76 – 1.67 (m, 1H).

**$^{13}$C NMR (101 MHz, CDCl$_3$)**: $\delta$ 167.4, 143.6, 134.8, 129.1, 127.6, 68.0, 56.3, 54.3, 48.8, 30.9, 24.4, 21.6.

**IR (neat, cm$^{-1}$):** 2924, 1666, 1597, 1494, 1460, 1344, 1156, 708, 664.

**HRMS (ESI-TOF):** calculated for [$C_{14}H_{19}N_2O_3S$ (M + H$^+$)]: 295.1116, found: 295.1111.

## 5.4 Asymmetric version:

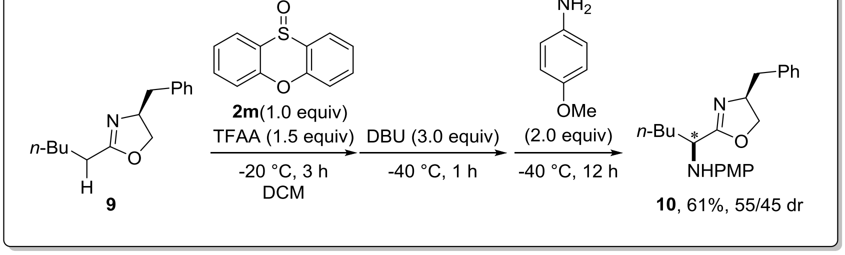


***N*-((*S*)-1-((*S*)-4-benzyl-4,5-dihydrooxazol-2-yl)pentyl)-4-methoxyaniline (10):**

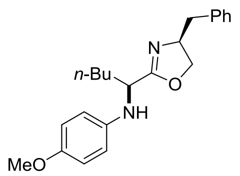

To a mixture of 2-oxazoline **9** (115.7 mg, 0.5 mmol) and sulfoxide **2m** (108.0 mg, 1.0 equiv) in DCM (5 mL) was added TFAA (104 μL, 1.5 equiv) at −20 °C. After stirring for 3 h, the reaction mixture was then cooled to −40 °C. To the mixture was added a solution of DBU (228.4 mg, 3.0 equiv) in DCM (1 mL) dropwise in 10 min using syringe pump. After stirring for 1 h, to the mixture was added a solution of nucleophile (1.0 mmol, 2.0 equiv) in DCM (1 mL) dropwise in 10 min using syringe pump. The mixture was then stirred for 12 h. After that, the mixture was treated with saturated aqueous $NaHCO_3$ solution and extracted with DCM. The organic layer was dried over $Na_2SO_4$ and concentrated. The obtained residue was purified by silica gel column chromatography to afford the title compound **10** as light yellow oil, 107.5 mg, 61% yield (dr: 55/45). ($R_f$= 0.25, eluent: PE/EtOAc = 1/1).

**$^1$H NMR (400 MHz, CDCl$_3$):** *δ* 7.20 – 7.05 (m, 4H), 7.01 (d, *J* = 7.2 Hz, 1H), 6.70 – 6.66 (m, 2H), 6.58 – 6.54 (m, 2H), 4.31 – 4.22 (m, 1H), 4.09 – 4.04 (m, 1H), 4.00 – 3.95 (m, 1H), 3.90 – 3.81 (m, 1.06 H), 3.75 – 3.68 (m, 0.87 H), 3.64 (s, 3H), 2.96 – 2.81 (m, 2H), 2.54 – 2.45 (m, 1H), 1.72 – 1.59 (m, 2H), 1.37 – 1.20 (m, 4H), 0.84 – 0.79 (m, 3H).

**$^{13}$C NMR (101 MHz, CDCl$_3$)**: *δ* 168.3, 152.3, 141.0, 140.9, 137.6, 137.5, 129.2, 129.1, 128.34, 128.30, 126.32, 126.30, 115.0, 114.57, 114.56, 71.69, 71.67, 66.9, 66.8, 55.5, 52.8, 52.8, 41.6, 41.5, 33.1, 27.8, 27.7, 22.34, 22.32, 13.8.

**IR (neat, cm$^{-1}$):** 3372, 2930, 2859, 1662, 1510, 1454, 1234, 1038, 819, 731, 700.

**HRMS (ESI-TOF):** calculated for [$C_{22}H_{29}N_2O_2$ (M + H$^+$)]: 353.2229, found: 353.2224;

# 6. Density functional theory (DFT) calculations

## Computational Details

All structures were optimized at M062X[9] /6-31G(d,p) level with solvent effects accounted by the SMD[10] solvent model, using the experimental solvent (dichloromethane). Harmonic frequency analysis calculations were subsequently performed to verify the optimized geometries to be minima (no imaginary frequency) or transition states (TSs, having unique one imaginary frequency). The energies were then improved by M062X/6-311++G(d,p)//M062X/6-31G(d,p) single-point calculations with solvent effects accounted by the SMD solvent model, using the experimental solvent (dichloromethane). The refined energies were then corrected to enthalpies and free energies at experimental temperature (233.15K) and 1 atm, using the M062X/6-31G(d,p) harmonic frequencies. Total energies and Cartesian coordinates of all optimized structures are given below in this Supporting Information. All standard DFT calculations were carried out using Gaussian 09 program.[11]

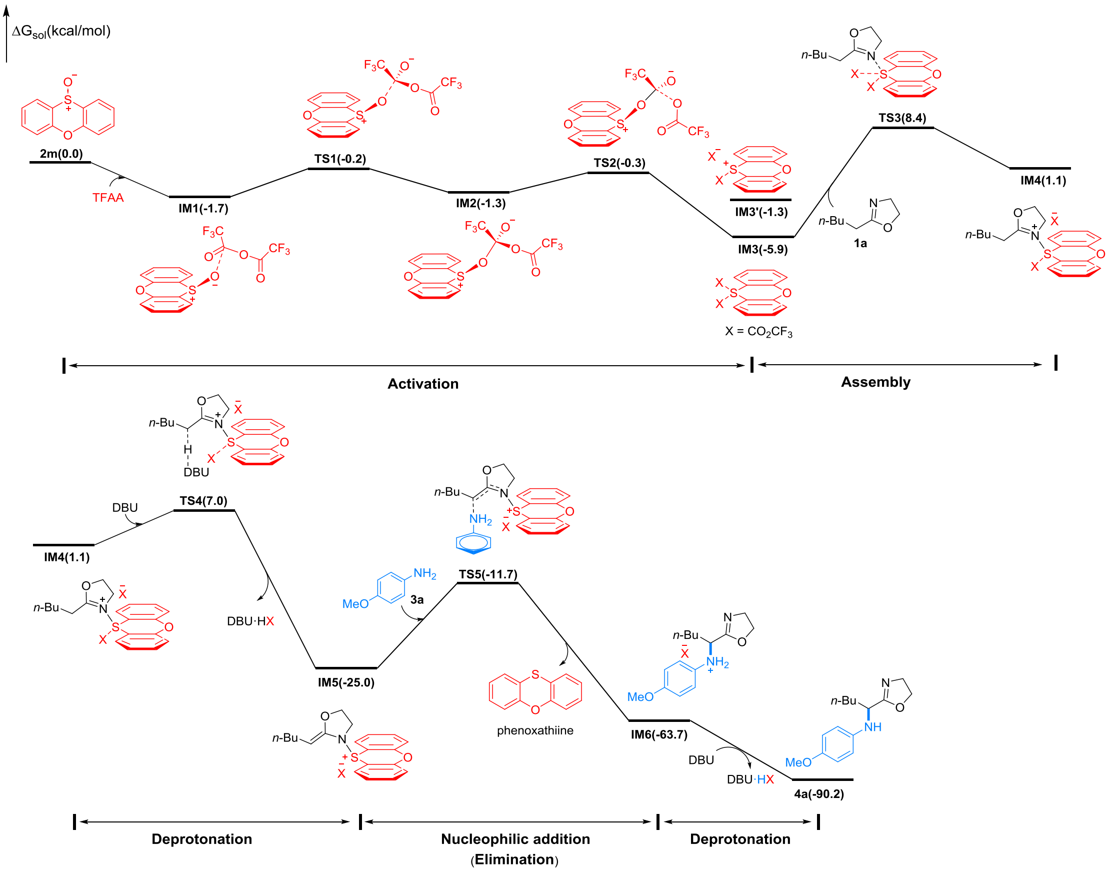


Fig S1. Free energy profile for the conversion of **1a** to **4a**.

## Cartesian Coordinates (in Å), SCF Energies, and Free Energies (in a.u.) at 233.15 K and 1 atm for the Optimized Structures

BSI=6-31G(d,p)

BSII=6-311++(d,p)

**2m**

M062x/BSI SCF energy: -1010.408084 a.u.
M062x/BSII SCF energy in solution:
-2165.726397a.u.
M062x/BSII free energy in solution:
-2165.614701 a.u.

| | | | |
|---|---|---|---|
| C | 0.098579 | -0.619019 | -3.663653 |
| C | 0.942218 | -0.598161 | -2.562931 |
| C | 0.467569 | -0.174276 | -1.318081 |
| C | -0.863894 | 0.218670 | -1.184800 |
| C | -1.714401 | 0.212321 | -2.290328 |
| C | -1.227820 | -0.201440 | -3.521997 |
| C | -0.863894 | 0.218670 | 1.184800 |
| C | 0.467569 | -0.174276 | 1.318081 |
| C | 0.942218 | -0.598161 | 2.562931 |
| H | 1.981841 | -0.901104 | 2.656887 |
| C | 0.098579 | -0.619019 | 3.663653 |
| C | -1.227820 | -0.201440 | 3.521997 |
| C | -1.714401 | 0.212321 | 2.290328 |
| H | 0.468566 | -0.951048 | -4.627223 |
| H | 1.981841 | -0.901104 | -2.656887 |
| H | -2.746455 | 0.519367 | -2.157526 |
| H | -1.892789 | -0.210035 | -4.379435 |
| H | 0.468566 | -0.951048 | 4.627223 |
| H | -1.892789 | -0.210035 | 4.379435 |
| H | -2.746455 | 0.519367 | 2.157526 |
| O | -1.421378 | 0.603510 | 0.000000 |
| S | 1.653002 | 0.028738 | 0.000000 |
| O | 2.109207 | 1.467574 | 0.000000 |

**TFAA**

M062x/BSI SCF energy: -976.803949 a.u.
M062x/BSII SCF energy in solution:
-977.135279 a.u.
M062x/BSII free energy in solution:
-977.109196 a.u.

| | | | |
|---|---|---|---|
| C | -0.081715 | 1.190101 | 0.696519 |
| O | 0.000000 | 0.000000 | 0.026183 |
| C | 0.081715 | -1.190101 | 0.696519 |
| C | 0.115382 | 2.318094 | -0.329780 |
| C | -0.115382 | -2.318094 | -0.329780 |
| F | -0.795997 | 2.224835 | -1.295585 |
| F | 1.324693 | 2.221310 | -0.878454 |
| F | 0.000000 | 3.491002 | 0.271189 |
| F | -1.324693 | -2.221310 | -0.878454 |
| F | 0.000000 | -3.491002 | 0.271189 |
| F | 0.795997 | -2.224835 | -1.295585 |
| O | -0.285337 | 1.353604 | 1.852560 |
| O | 0.285337 | -1.353604 | 1.852560 |

**$X^-$**

M062x/BSI SCF energy: -526.137238 a.u.
M062x/BSII SCF energy in solution:
-526.343737 a.u.
M062x/BSII free energy in solution:
-526.338458 a.u.

| | | | |
|---|---|---|---|
| O | 1.578166 | 1.129183 | -0.000038 |
| O | 1.507484 | -1.142493 | -0.000015 |
| C | -0.516032 | 0.015889 | -0.000033 |
| F | -1.015137 | -0.625315 | -1.079135 |
| F | -1.063700 | 1.243507 | -0.000818 |
| F | -1.014964 | -0.623811 | 1.080067 |
| C | 1.042534 | 0.010285 | -0.000068 |

**1a**

M062x/BSI SCF energy: -443.671807982 a.u.
M062x/BSII SCF energy in solution:
-443.788158739 a.u.
M062x/BSII free energy in solution:
-443.5903587 a.u.

| | | | |
|---|---|---|---|
| C | 3.207338 | 1.083726 | -0.058483 |
| C | 3.597700 | -0.289654 | 0.527460 |
| C | 1.522569 | -0.392136 | -0.201537 |
| H | 3.801901 | 1.369293 | -0.929045 |
| H | 3.854350 | -0.230751 | 1.589147 |
| H | 4.447673 | -0.738673 | 0.005832 |
| H | 3.229749 | 1.892122 | 0.675646 |
| N | 2.402145 | -1.125275 | 0.349741 |
| O | 1.842796 | 0.896133 | -0.484670 |
| C | 0.131975 | -0.788055 | -0.572460 |
| C | -0.923570 | 0.062396 | 0.143420 |
| H | 0.007909 | -1.846443 | -0.330733 |
| H | 0.019420 | -0.671355 | -1.657177 |
| C | -2.343916 | -0.336323 | -0.247444 |
| H | -0.795537 | -0.042985 | 1.228207 |

| | | | |
|---|---|---|---|
| H | -0.756702 | 1.119034 | -0.095012 |
| C | -3.411512 | 0.493382 | 0.462508 |
| H | -2.464183 | -0.230241 | -1.334494 |
| H | -2.500099 | -1.399776 | -0.020721 |
| H | -3.292394 | 0.380987 | 1.547556 |
| H | -3.247428 | 1.555575 | 0.241488 |
| C | -4.827700 | 0.096379 | 0.056741 |
| H | -5.020832 | -0.956551 | 0.287932 |
| H | -5.579055 | 0.696129 | 0.578532 |
| H | -4.979466 | 0.233200 | -1.019215 |

**3a**

M062x/BSI SCF energy: -401.968855 a.u.
M062x/BSII SCF energy in solution:
-402.079561 a.u.
M062x/BSII free energy in solution:
-401. 952378 a.u.

| | | | |
|---|---|---|---|
| C | 0.421247 | -1.005925 | -0.004206 |
| C | -0.961983 | -1.194501 | -0.008518 |
| C | -1.843865 | -0.111638 | -0.010099 |
| C | -1.299114 | 1.181962 | -0.006634 |
| C | 0.073260 | 1.376099 | -0.001066 |
| C | 0.948313 | 0.285043 | -0.000394 |
| H | 1.068549 | -1.875291 | -0.001503 |
| H | -1.360625 | -2.205637 | -0.011975 |
| H | -1.965709 | 2.040393 | -0.008496 |
| H | 0.491894 | 2.377764 | 0.005097 |
| N | -3.230082 | -0.304899 | -0.078364 |
| H | -3.533498 | -1.187367 | 0.315758 |
| H | -3.762498 | 0.458735 | 0.320743 |
| O | 2.279792 | 0.584278 | 0.007204 |
| C | 3.183389 | -0.502225 | 0.006313 |
| H | 3.066500 | -1.120480 | -0.892072 |
| H | 4.184514 | -0.069429 | 0.015470 |
| H | 3.055630 | -1.131511 | 0.895518 |

**DBU**

M062x/BSI SCF energy: -461.910609 a.u.
M062x/BSII SCF energy in solution:
-462.025284 a.u.
M062x/BSII free energy in solution:
-461.802844 a.u.

| | | | |
|---|---|---|---|
| C | -0.347141 | 0.723066 | -0.320057 |
| N | -0.358760 | -0.651930 | -0.369513 |
| C | -1.514880 | -1.369193 | 0.158647 |
| C | -2.772197 | -0.545179 | -0.065430 |
| H | -1.384946 | -1.581055 | 1.230885 |
| H | -1.585880 | -2.330682 | -0.359105 |
| H | -3.631268 | -1.035207 | 0.401443 |
| H | -2.967621 | -0.475009 | -1.141279 |
| C | -2.555951 | 0.849303 | 0.509228 |
| H | -2.550449 | 0.796847 | 1.607686 |
| H | -3.392684 | 1.503451 | 0.239611 |
| N | -1.319572 | 1.478765 | 0.059974 |
| C | 0.855379 | -1.432324 | -0.589866 |
| C | 1.922309 | -1.284703 | 0.500825 |
| C | 2.797780 | -0.044218 | 0.326019 |
| C | 2.040704 | 1.283290 | 0.353962 |
| C | 0.941328 | 1.413041 | -0.714379 |
| H | 1.291765 | -1.181474 | -1.564967 |
| H | 1.427547 | -1.274161 | 1.480719 |
| H | 3.321267 | -0.127002 | -0.636749 |
| H | 1.590817 | 1.440876 | 1.342355 |
| H | 0.537139 | -2.475944 | -0.651907 |
| H | 2.564035 | -2.172800 | 0.477925 |
| H | 3.571258 | -0.032197 | 1.102164 |
| H | 2.760516 | 2.095374 | 0.206799 |
| H | 0.682428 | 2.464921 | -0.845812 |
| H | 1.310418 | 1.037713 | -1.676697 |

**DBU·HX**

M062x/BSI SCF energy: -988.566394 a.u.
M062x/BSII SCF energy in solution:
-988.86931 a.u.
M062x/BSII free energy in solution:
-988.613666 a.u.

| | | | |
|---|---|---|---|
| C | 1.569031 | 0.205740 | 0.215816 |
| N | 2.848550 | 0.548021 | 0.356671 |
| C | 3.304029 | 1.909392 | 0.041490 |
| C | 2.171645 | 2.907825 | 0.220228 |
| H | 3.688550 | 1.930364 | -0.985409 |
| H | 4.129633 | 2.142727 | 0.716962 |
| H | 2.494291 | 3.890921 | -0.128280 |
| H | 1.916665 | 2.985713 | 1.281624 |
| C | 0.957389 | 2.439064 | -0.562841 |

| | | | |
|---|---|---|---|
| H | 1.140635 | 2.506382 | -1.641121 |
| H | 0.072054 | 3.034923 | -0.332117 |
| N | 0.660174 | 1.056241 | -0.206811 |
| C | 3.880785 | -0.450180 | 0.675890 |
| C | 4.090073 | -1.492720 | -0.423984 |
| C | 3.066761 | -2.628558 | -0.404718 |
| C | 1.615232 | -2.186374 | -0.584578 |
| C | 1.126538 | -1.202391 | 0.493866 |
| H | 3.636674 | -0.936782 | 1.626857 |
| H | 4.086655 | -0.983865 | -1.396127 |
| H | 3.153521 | -3.156487 | 0.554303 |
| H | 1.471132 | -1.737191 | -1.574613 |
| H | 4.800997 | 0.114692 | 0.832005 |
| H | 5.089934 | -1.918847 | -0.290968 |
| H | 3.320248 | -3.352899 | -1.185827 |
| H | 0.968799 | -3.067770 | -0.544917 |
| H | 0.034304 | -1.198558 | 0.552067 |
| H | 1.501849 | -1.509640 | 1.476477 |
| O | -1.930668 | 0.645494 | -0.610785 |
| C | -2.552617 | -0.141460 | 0.142839 |
| C | -4.093247 | -0.132012 | -0.021817 |
| F | -4.553038 | -1.361718 | -0.312916 |
| F | -4.527290 | 0.689801 | -0.985391 |
| F | -4.691708 | 0.246008 | 1.122049 |
| O | -2.142536 | -0.928149 | 1.002492 |
| H | -0.346764 | 0.750953 | -0.310403 |

**Phenoxathiine**

M062x/BSI SCF energy: -935.266789 a.u.
M062x/BSII SCF energy in solution:
-935.432305 a.u.
M062x/BSII free energy in solution:
-935.291358 a.u.

| | | | |
|---|---|---|---|
| C | 1.338630 | 0.664131 | 0.102164 |
| C | -1.338633 | 0.664159 | 0.102137 |
| C | 2.563512 | 1.159210 | -0.343658 |
| C | 1.180612 | -0.709576 | 0.287105 |
| C | -1.180596 | -0.709563 | 0.287103 |
| C | 3.625612 | 0.288707 | -0.573495 |
| H | 2.682368 | 2.227070 | -0.501148 |
| C | 2.230924 | -1.585093 | 0.038254 |
| C | 3.459162 | -1.081276 | -0.380126 |
| H | 4.578941 | 0.681975 | -0.909966 |
| H | 2.069795 | -2.647748 | 0.186704 |
| H | 4.282428 | -1.763552 | -0.564073 |
| S | 0.000010 | 1.760083 | 0.501991 |
| O | -0.000009 | -1.248788 | 0.747785 |
| C | -2.563515 | 1.159195 | -0.343678 |
| H | -2.682404 | 2.227050 | -0.501183 |
| C | -3.625626 | 0.288691 | -0.573500 |
| H | -4.578948 | 0.682017 | -0.909922 |
| C | -3.459178 | -1.081281 | -0.380110 |
| H | -4.282427 | -1.763587 | -0.564004 |
| C | -2.230915 | -1.585061 | 0.038255 |
| H | -2.069775 | -2.647713 | 0.186741 |

**IM1**

M062x/BSI SCF energy: -1987.233196 a.u.
M062x/BSII SCF energy in solution:
-1987.755162 a.u.
M062x/BSII free energy in solution:
-1987.567896 a.u.

| | | | |
|---|---|---|---|
| O | 0.015577 | -0.350909 | 0.381731 |
| C | -2.794957 | 0.454208 | -0.381990 |
| O | -2.623242 | -0.818587 | 0.038209 |
| C | -1.699339 | -1.618653 | -0.600122 |
| C | -3.980462 | 1.056935 | 0.389177 |
| C | -1.240367 | -2.740151 | 0.339814 |
| F | -3.734371 | 1.041924 | 1.698142 |
| F | -5.082040 | 0.342121 | 0.160527 |
| F | -4.183357 | 2.306242 | -0.000832 |
| F | -1.251477 | -2.390474 | 1.616075 |
| F | -0.022181 | -3.142548 | -0.005885 |
| F | -2.078467 | -3.775867 | 0.184494 |
| O | -2.155148 | 1.048730 | -1.189922 |
| O | -1.465512 | -1.608576 | -1.766301 |
| C | 2.528269 | -0.647688 | -0.405456 |
| C | 1.403089 | 1.748288 | -0.458719 |
| C | 2.825337 | -1.894083 | -0.963229 |
| C | 3.396586 | -0.071106 | 0.521193 |
| C | 2.392156 | 2.077247 | 0.468491 |
| C | 3.980916 | -2.563327 | -0.589875 |
| H | 2.134944 | -2.331864 | -1.679014 |
| C | 4.553943 | -0.746127 | 0.908219 |
| C | 4.835404 | -1.987206 | 0.354950 |
| H | 4.213328 | -3.530042 | -1.022280 |

| | | | |
|---|---|---|---|
| H | 5.216830 | -0.277955 | 1.628022 |
| H | 5.738344 | -2.508777 | 0.655380 |
| S | 0.938935 | 0.060946 | -0.761744 |
| O | 3.193810 | 1.157174 | 1.079586 |
| C | 0.658872 | 2.757403 | -1.077213 |
| H | -0.108976 | 2.484503 | -1.795041 |
| C | 0.893078 | 4.086196 | -0.756978 |
| H | 0.317907 | 4.870172 | -1.236627 |
| C | 1.868679 | 4.405500 | 0.191676 |
| H | 2.053793 | 5.443300 | 0.449297 |
| C | 2.621441 | 3.411994 | 0.801206 |
| H | 3.398152 | 3.643712 | 1.522188 |

**TS1**

M062x/BSI SCF energy: -1987.231627 a.u.
M062x/BSII SCF energy in solution:
-1987.754373 a.u.
M062x/BSII free energy in solution:
-1987.565408 a.u.

| | | | |
|---|---|---|---|
| O | -0.036385 | -0.310192 | 0.299497 |
| C | -2.740609 | 0.292212 | -0.430623 |
| O | -2.408788 | -0.875888 | 0.106081 |
| C | -1.214679 | -1.522387 | -0.366800 |
| C | -4.123501 | 0.710244 | 0.096769 |
| C | -0.868272 | -2.587846 | 0.682775 |
| F | -4.117442 | 0.758580 | 1.429739 |
| F | -5.049566 | -0.171510 | -0.283715 |
| F | -4.450981 | 1.906257 | -0.374209 |
| F | -0.981476 | -2.153170 | 1.935360 |
| F | 0.377719 | -3.018723 | 0.488351 |
| F | -1.696501 | -3.629813 | 0.531289 |
| O | -2.110207 | 0.968332 | -1.192310 |
| O | -1.040456 | -1.728107 | -1.546738 |
| C | 2.425035 | -0.516454 | -0.608359 |
| C | 1.245203 | 1.865196 | -0.364661 |
| C | 2.701165 | -1.711733 | -1.279856 |
| C | 3.307629 | -0.020807 | 0.350967 |
| C | 2.265162 | 2.104271 | 0.558485 |
| C | 3.862852 | -2.408246 | -0.987484 |
| H | 1.989135 | -2.090071 | -2.008067 |
| C | 4.471911 | -0.726189 | 0.650989 |
| C | 4.738857 | -1.913072 | -0.015967 |
| H | 4.083475 | -3.335607 | -1.503548 |

| | | | |
|---|---|---|---|
| H | 5.149626 | -0.323333 | 1.395586 |
| H | 5.647036 | -2.458276 | 0.219306 |
| S | 0.849925 | 0.226297 | -0.878363 |
| O | 3.114275 | 1.147151 | 1.025017 |
| C | 0.446093 | 2.915244 | -0.829865 |
| H | -0.341144 | 2.707622 | -1.547529 |
| C | 0.657763 | 4.198974 | -0.353529 |
| H | 0.039871 | 5.016484 | -0.707102 |
| C | 1.664239 | 4.430060 | 0.589710 |
| H | 1.829265 | 5.433298 | 0.968696 |
| C | 2.470045 | 3.395938 | 1.041606 |
| H | 3.267297 | 3.561736 | 1.757831 |

**IM2**

M062x/BSI SCF energy: -1987.232564 a.u.
M062x/BSII SCF energy in solution:
-1987.755745 a.u.
M062x/BSII free energy in solution:
-1987.567188 a.u.

| | | | |
|---|---|---|---|
| O | -0.066969 | -0.242704 | 0.319571 |
| C | -2.703240 | 0.287442 | -0.437723 |
| O | -2.343077 | -0.821783 | 0.163832 |
| C | -0.988770 | -1.380830 | -0.174465 |
| C | -4.174792 | 0.597686 | -0.108754 |
| C | -0.787641 | -2.423907 | 0.941369 |
| F | -4.356735 | 0.661036 | 1.212197 |
| F | -4.968480 | -0.361729 | -0.591095 |
| F | -4.536789 | 1.757989 | -0.643558 |
| F | -0.979292 | -1.932877 | 2.168771 |
| F | 0.460247 | -2.899074 | 0.874331 |
| F | -1.631528 | -3.445263 | 0.768559 |
| O | -2.049827 | 1.011697 | -1.145032 |
| O | -0.799708 | -1.736733 | -1.344294 |
| C | 2.320454 | -0.527329 | -0.739040 |
| C | 1.239413 | 1.888057 | -0.295068 |
| C | 2.493906 | -1.721293 | -1.450709 |
| C | 3.276663 | -0.101011 | 0.183871 |
| C | 2.323224 | 2.043647 | 0.570573 |
| C | 3.633972 | -2.478141 | -1.240528 |
| H | 1.714334 | -2.049196 | -2.131600 |
| C | 4.420892 | -0.868305 | 0.394456 |
| C | 4.590848 | -2.047217 | -0.315146 |
| H | 3.776232 | -3.405650 | -1.783125 |

| | | | |
|---|---|---|---|
| H | 5.157181 | -0.517736 | 1.109350 |
| H | 5.484047 | -2.639720 | -0.145745 |
| S | 0.791506 | 0.315689 | -0.933267 |
| O | 3.181800 | 1.045669 | 0.909704 |
| C | 0.433047 | 2.981513 | -0.636746 |
| H | -0.404898 | 2.835565 | -1.310644 |
| C | 0.703672 | 4.223534 | -0.088954 |
| H | 0.083458 | 5.074475 | -0.345591 |
| C | 1.774338 | 4.371049 | 0.799233 |
| H | 1.985356 | 5.342759 | 1.233612 |
| C | 2.584900 | 3.295287 | 1.126749 |
| H | 3.430308 | 3.397475 | 1.798232 |

**TS2**

M062x/BSI SCF energy: -1987.226534 a.u.
M062x/BSII SCF energy in solution:
-1987.753537 a.u.
M062x/BSII free energy in solution:
-1987.565618 a.u.

| | | | |
|---|---|---|---|
| O | 0.099930 | -0.475457 | 0.385976 |
| C | -2.656555 | 0.123326 | -0.516646 |
| O | -2.480434 | -0.940262 | 0.127490 |
| C | -0.537501 | -1.656358 | 0.018845 |
| C | -4.128164 | 0.599384 | -0.480926 |
| C | -0.842803 | -2.460748 | 1.287291 |
| F | -4.529216 | 0.812726 | 0.781758 |
| F | -4.942506 | -0.329052 | -1.005016 |
| F | -4.316236 | 1.733237 | -1.161198 |
| F | -1.204270 | -1.706750 | 2.317192 |
| F | 0.283486 | -3.108591 | 1.628142 |
| F | -1.781015 | -3.364511 | 1.045491 |
| O | -1.854452 | 0.815461 | -1.153685 |
| O | -0.384022 | -2.162866 | -1.060643 |
| C | 2.403293 | -0.377837 | -0.877143 |
| C | 1.080779 | 1.839977 | -0.164298 |
| C | 2.700747 | -1.422749 | -1.763559 |
| C | 3.336153 | 0.045341 | 0.072045 |
| C | 2.168519 | 2.014934 | 0.698088 |
| C | 3.934609 | -2.043722 | -1.685276 |
| H | 1.954588 | -1.743292 | -2.484332 |
| C | 4.572815 | -0.590908 | 0.153446 |
| C | 4.860119 | -1.628563 | -0.720324 |
| H | 4.176392 | -2.854744 | -2.362229 |

| | | | |
|---|---|---|---|
| H | 5.287845 | -0.248251 | 0.893120 |
| H | 5.825771 | -2.119031 | -0.655302 |
| S | 0.788872 | 0.294700 | -0.929579 |
| O | 3.120211 | 1.072977 | 0.933419 |
| C | 0.168333 | 2.879809 | -0.394598 |
| H | -0.675537 | 2.707950 | -1.053396 |
| C | 0.343201 | 4.085185 | 0.261754 |
| H | -0.359191 | 4.893736 | 0.096584 |
| C | 1.413175 | 4.248507 | 1.148224 |
| H | 1.543708 | 5.192018 | 1.667844 |
| C | 2.325159 | 3.227480 | 1.366236 |
| H | 3.171150 | 3.345708 | 2.034243 |

**IM3**

M062x/BSI SCF energy: -1987.23555 a.u.
M062x/BSII SCF energy in solution:
-1987.23555 a.u.
M062x/BSII free energy in solution:
-1987.574508 a.u.

| | | | |
|---|---|---|---|
| O | -1.785325 | 0.174268 | -0.722787 |
| C | 2.668900 | -0.859180 | -0.743496 |
| O | 2.223373 | -1.884394 | -1.213258 |
| C | -2.331655 | -0.956764 | -1.085942 |
| C | 4.201383 | -0.688931 | -0.671720 |
| C | -3.855259 | -0.794838 | -1.240290 |
| F | 4.714657 | -0.719690 | -1.905637 |
| F | 4.731644 | -1.703151 | 0.017081 |
| F | 4.573068 | 0.450466 | -0.093479 |
| F | -4.140576 | 0.155232 | -2.134435 |
| F | -4.401079 | -0.440313 | -0.073224 |
| F | -4.412758 | -1.932794 | -1.637821 |
| O | 2.018470 | 0.157882 | -0.269908 |
| O | -1.781404 | -2.014358 | -1.276640 |
| C | -0.109667 | -0.682871 | 1.039209 |
| C | 0.066309 | 1.778316 | -0.158541 |
| C | 0.176947 | -2.040289 | 1.191790 |
| C | -0.634763 | 0.068605 | 2.089047 |
| C | -0.478118 | 2.211508 | 1.045781 |
| C | -0.053267 | -2.645544 | 2.418467 |
| H | 0.565377 | -2.601370 | 0.348754 |
| C | -0.865751 | -0.549597 | 3.316955 |
| C | -0.576123 | -1.896873 | 3.475166 |
| H | 0.164713 | -3.699566 | 2.546491 |

| | | | |
|---|---|---|---|
| H | -1.268157 | 0.049470 | 4.126592 |
| H | -0.759248 | -2.368889 | 4.434706 |
| S | 0.100224 | 0.054315 | -0.558372 |
| O | -0.969328 | 1.387768 | 2.013271 |
| C | 0.556371 | 2.681958 | -1.100788 |
| H | 0.985081 | 2.315424 | -2.028485 |
| C | 0.482899 | 4.040968 | -0.837225 |
| H | 0.857435 | 4.752201 | -1.564261 |
| C | -0.076128 | 4.482862 | 0.364882 |
| H | -0.135568 | 5.545461 | 0.575570 |
| C | -0.550487 | 3.579811 | 1.304748 |
| H | -0.973667 | 3.906316 | 2.248532 |

**IM3’**

M062x/BSI SCF energy: -1987.227174 a.u.
M062x/BSII SCF energy in solution:
-1987.755286 a.u.
M062x/BSII free energy in solution:
-1987.567172 a.u.

| | | | |
|---|---|---|---|
| O | 0.167932 | -0.574529 | 0.381233 |
| C | -2.671716 | -0.015949 | -0.536501 |
| O | -2.570430 | -1.086462 | 0.088585 |
| C | -0.325518 | -1.798624 | 0.027411 |
| C | -4.099546 | 0.584312 | -0.511083 |
| C | -0.741589 | -2.575606 | 1.282855 |
| F | -4.584344 | 0.636793 | 0.739497 |
| F | -4.946351 | -0.173894 | -1.229066 |
| F | -4.154361 | 1.824652 | -1.011834 |
| F | -1.182501 | -1.799509 | 2.261087 |
| F | 0.355239 | -3.212537 | 1.727143 |
| F | -1.651203 | -3.486538 | 0.980417 |
| O | -1.823120 | 0.626768 | -1.184224 |
| O | -0.183014 | -2.298248 | -1.049056 |
| C | 2.445342 | -0.255393 | -0.885281 |
| C | 0.944875 | 1.831614 | -0.134322 |
| C | 2.833314 | -1.247749 | -1.797375 |
| C | 3.333888 | 0.212387 | 0.085844 |
| C | 2.010903 | 2.068558 | 0.742100 |
| C | 4.111279 | -1.771943 | -1.720409 |
| H | 2.124821 | -1.602956 | -2.539670 |
| C | 4.615611 | -0.326799 | 0.165832 |
| C | 4.991500 | -1.314122 | -0.732177 |
| H | 4.424228 | -2.540566 | -2.417313 |

| | | | |
|---|---|---|---|
| H | 5.295149 | 0.049397 | 0.922408 |
| H | 5.991704 | -1.729734 | -0.668769 |
| S | 0.782639 | 0.286033 | -0.935701 |
| O | 3.025670 | 1.194018 | 0.971275 |
| C | -0.040597 | 2.807206 | -0.350812 |
| H | -0.869841 | 2.583125 | -1.012188 |
| C | 0.043696 | 4.009083 | 0.328971 |
| H | -0.714296 | 4.767950 | 0.174388 |
| C | 1.092802 | 4.232517 | 1.227493 |
| H | 1.150782 | 5.172360 | 1.766483 |
| C | 2.073662 | 3.275414 | 1.434950 |
| H | 2.903172 | 3.441373 | 2.113195 |

**TS3**

M062x/BSI SCF energy: -2430.900647 a.u.
M062x/BSII SCF energy in solution:
-2431.547531 a.u.
M062x/BSII free energy in solution:
-2431.142082 a.u.

| | | | |
|---|---|---|---|
| C | -2.872819 | -1.131941 | -2.620901 |
| C | -1.388690 | -1.008956 | -3.012190 |
| C | -1.934027 | 0.685804 | -1.713948 |
| H | -3.082244 | -2.025173 | -2.030750 |
| H | -1.238079 | -0.953701 | -4.095163 |
| H | -0.808336 | -1.845069 | -2.614850 |
| H | -3.557720 | -1.071982 | -3.470875 |
| N | -0.938456 | 0.237033 | -2.375967 |
| O | -3.099993 | 0.018434 | -1.783658 |
| C | -1.944946 | 1.888296 | -0.832843 |
| C | -3.093490 | 2.856219 | -1.132702 |
| H | -0.979523 | 2.395553 | -0.934461 |
| H | -2.041558 | 1.499325 | 0.188978 |
| C | -3.054576 | 4.078272 | -0.219076 |
| H | -3.042574 | 3.178693 | -2.180650 |
| H | -4.046679 | 2.330193 | -1.008031 |
| C | -4.204353 | 5.051293 | -0.470817 |
| H | -3.079850 | 3.747754 | 0.828563 |
| H | -2.098723 | 4.603359 | -0.354921 |
| H | -4.190164 | 5.361621 | -1.523457 |
| H | -5.157550 | 4.530742 | -0.314435 |
| O | 2.947930 | 0.633907 | -0.173111 |
| C | -2.022881 | -1.623329 | 0.715591 |
| O | -2.427356 | -0.571433 | 1.223277 |

| | | | |
|---|---|---|---|
| C | 3.185547 | 1.904178 | 0.171648 |
| C | -2.557583 | -2.940622 | 1.339223 |
| C | 4.703002 | 2.119331 | 0.329225 |
| F | -3.337137 | -2.760187 | 2.414673 |
| F | -1.544244 | -3.744975 | 1.723279 |
| F | -3.282842 | -3.641080 | 0.442308 |
| F | 5.320827 | 1.866063 | -0.822859 |
| F | 5.185536 | 1.295822 | 1.257982 |
| F | 4.943768 | 3.370219 | 0.688923 |
| O | -1.230553 | -1.844793 | -0.226853 |
| O | 2.367585 | 2.761312 | 0.342257 |
| C | 0.711582 | -0.063678 | 1.110376 |
| C | 1.532924 | -1.282267 | -1.154337 |
| C | 0.196432 | 0.998371 | 1.870079 |
| C | 0.814052 | -1.352124 | 1.648850 |
| C | 1.522134 | -2.423216 | -0.347769 |
| C | -0.191425 | 0.770256 | 3.175160 |
| H | 0.118060 | 1.985855 | 1.426754 |
| C | 0.410469 | -1.572915 | 2.963884 |
| C | -0.084031 | -0.517408 | 3.713329 |
| H | -0.586773 | 1.583573 | 3.772142 |
| H | 0.479268 | -2.578622 | 3.362312 |
| H | -0.398767 | -0.700944 | 4.735478 |
| S | 1.247681 | 0.294361 | -0.496545 |
| O | 1.285029 | -2.427828 | 0.981236 |
| C | 1.806888 | -1.363453 | -2.527706 |
| H | 1.779415 | -0.462209 | -3.132660 |
| C | 2.091415 | -2.593466 | -3.086957 |
| H | 2.309189 | -2.670295 | -4.145746 |
| C | 2.080477 | -3.739830 | -2.278877 |
| H | 2.289503 | -4.709248 | -2.719304 |
| C | 1.798339 | -3.662874 | -0.926153 |
| H | 1.776509 | -4.543138 | -0.293687 |
| C | -4.137847 | 6.282047 | 0.428332 |
| H | -4.969631 | 6.965942 | 0.236486 |
| H | -4.175286 | 5.996579 | 1.484996 |
| H | -3.206052 | 6.834703 | 0.267302 |

**IM4**

M062x/BSI SCF energy: -1904.74349391 a.u.

M062x/BSII SCF energy in solution:
-1905.19701178 a.u.

M062x/BSII free energy in solution:
-1904.815295 a.u.

| | | | |
|---|---|---|---|
| C | -1.936539 | -3.606860 | 0.778101 |
| C | -0.559537 | -2.927974 | 0.682816 |
| C | -2.204093 | -1.506169 | -0.000523 |
| H | -2.218973 | -3.876614 | 1.794498 |
| H | 0.112478 | -3.430971 | -0.015630 |
| H | -0.070053 | -2.822267 | 1.653355 |
| H | -2.046258 | -4.460791 | 0.111053 |
| N | -0.918006 | -1.598285 | 0.160668 |
| O | -2.874197 | -2.584043 | 0.325975 |
| C | -2.967688 | -0.340124 | -0.528043 |
| C | -4.386000 | -0.250318 | 0.041703 |
| H | -3.005816 | -0.462098 | -1.619033 |
| H | -2.408428 | 0.578168 | -0.327093 |
| C | -5.138170 | 0.947872 | -0.530258 |
| H | -4.928970 | -1.172785 | -0.187912 |
| H | -4.333639 | -0.171228 | 1.133856 |
| C | -6.561351 | 1.055995 | 0.012791 |
| H | -4.588321 | 1.870388 | -0.301280 |
| H | -5.171039 | 0.867073 | -1.625130 |
| H | -7.102416 | 0.127494 | -0.208591 |
| H | -6.524355 | 1.140902 | 1.106068 |
| O | 1.582393 | 1.232196 | -0.670009 |
| C | 1.196713 | 2.390632 | -0.211358 |
| C | 2.162554 | 3.506235 | -0.652093 |
| F | 2.188871 | 3.592099 | -1.985410 |
| F | 3.403119 | 3.245743 | -0.232050 |
| F | 1.786088 | 4.680644 | -0.159006 |
| O | 0.222062 | 2.626137 | 0.466074 |
| C | 1.032858 | -0.280576 | 1.359046 |
| C | 1.326752 | -1.228622 | -1.196883 |
| C | 0.288225 | 0.117235 | 2.469003 |
| C | 2.345621 | -0.728961 | 1.483539 |
| C | 2.596914 | -1.562372 | -0.736365 |
| C | 0.873402 | 0.068958 | 3.724742 |
| H | -0.731150 | 0.467831 | 2.338932 |
| C | 2.928372 | -0.767159 | 2.749368 |
| C | 2.194706 | -0.367600 | 3.856679 |
| H | 0.307490 | 0.378974 | 4.595562 |
| H | 3.950052 | -1.120190 | 2.836718 |
| H | 2.656873 | -0.402221 | 4.837502 |
| S | 0.302470 | -0.134641 | -0.250638 |
| O | 3.128379 | -1.136213 | 0.443941 |
| C | 0.842213 | -1.704826 | -2.415191 |

| | | | |
|---|---|---|---|
| H | -0.155582 | -1.430827 | -2.746238 |
| C | 1.652164 | -2.521160 | -3.189463 |
| H | 1.290510 | -2.893144 | -4.140963 |
| C | 2.934285 | -2.849847 | -2.740326 |
| H | 3.571476 | -3.485617 | -3.345582 |
| C | 3.406984 | -2.380796 | -1.523381 |
| H | 4.395436 | -2.636424 | -1.157124 |
| C | -7.319370 | 2.244401 | -0.570240 |
| H | -8.334628 | 2.307365 | -0.168736 |
| H | -6.809165 | 3.185658 | -0.340577 |
| H | -7.394885 | 2.164814 | -1.659772 |

**TS4**

M062x/BSI SCF energy: -2366.661982 a.u.
M062x/BSII SCF energy in solution:
-2367.229021 a.u.
M062x/BSII free energy in solution:
-2366.608633 a.u.

| | | | |
|---|---|---|---|
| C | 2.620130 | -1.083582 | 0.173719 |
| N | 2.733760 | -2.242623 | 0.851616 |
| C | 2.064652 | -2.422830 | 2.147575 |
| C | 0.913271 | -1.442115 | 2.304792 |
| H | 2.797291 | -2.297344 | 2.954686 |
| H | 1.696847 | -3.451906 | 2.187049 |
| H | 0.535337 | -1.479461 | 3.329044 |
| H | 0.090125 | -1.719864 | 1.636623 |
| C | 1.406292 | -0.045203 | 1.950877 |
| H | 2.158167 | 0.283773 | 2.680668 |
| H | 0.598638 | 0.695156 | 1.971899 |
| N | 1.987672 | -0.033844 | 0.615761 |
| C | 3.619220 | -3.326978 | 0.411431 |
| C | 5.100953 | -2.947495 | 0.358174 |
| C | 5.506551 | -2.191658 | -0.907089 |
| C | 4.797647 | -0.854451 | -1.112700 |
| C | 3.265640 | -0.973405 | -1.188002 |
| H | 3.290040 | -3.708911 | -0.563706 |
| H | 5.346916 | -2.364206 | 1.254150 |
| H | 5.298250 | -2.832455 | -1.774626 |
| H | 5.059867 | -0.152717 | -0.311138 |
| H | 3.476760 | -4.134914 | 1.131422 |
| H | 5.682712 | -3.873765 | 0.411444 |
| H | 6.588312 | -2.023298 | -0.893831 |
| H | 5.149729 | -0.409253 | -2.048160 |
| H | 2.855085 | -0.078436 | -1.663567 |
| H | 2.988743 | -1.835358 | -1.807369 |
| O | -2.147570 | -0.426720 | 1.398507 |
| C | -1.980074 | 0.564226 | 2.180314 |
| C | -2.054632 | 0.227525 | 3.685851 |
| F | -0.862023 | 0.466740 | 4.259835 |
| F | -2.368822 | -1.046123 | 3.925309 |
| F | -2.955255 | 1.000638 | 4.302820 |
| O | -1.720949 | 1.729899 | 1.895844 |
| C | -2.879905 | 0.838809 | -0.874538 |
| C | -1.496656 | -1.505077 | -0.939705 |
| C | -3.012420 | 2.213237 | -1.065343 |
| C | -3.980056 | -0.011638 | -0.925807 |
| C | -2.773280 | -2.058948 | -0.963016 |
| C | -4.270363 | 2.743071 | -1.309425 |
| H | -2.136081 | 2.853748 | -1.012350 |
| C | -5.243060 | 0.529380 | -1.162680 |
| C | -5.382076 | 1.896998 | -1.348138 |
| H | -4.386928 | 3.810797 | -1.455276 |
| H | -6.093351 | -0.142781 | -1.200702 |
| H | -6.368902 | 2.309431 | -1.529629 |
| S | -1.267960 | 0.197914 | -0.487239 |
| O | -3.923321 | -1.362871 | -0.751069 |
| C | -0.366186 | -2.279428 | -1.200252 |
| H | 0.613896 | -1.811737 | -1.194840 |
| C | -0.514110 | -3.631378 | -1.468742 |
| H | 0.358767 | -4.240506 | -1.674860 |
| C | -1.791451 | -4.198841 | -1.466741 |
| H | -1.913853 | -5.257528 | -1.669283 |
| C | -2.914191 | -3.422383 | -1.223256 |
| H | -3.913911 | -3.842506 | -1.237315 |
| C | 0.401349 | 0.828186 | -4.093028 |
| C | -0.879525 | 0.407456 | -3.375904 |
| C | 0.546776 | 1.552790 | -1.952092 |
| H | 0.234105 | 1.373860 | -5.019080 |
| H | -1.104884 | -0.651158 | -3.510930 |
| H | -1.746543 | 1.017060 | -3.644819 |
| H | 1.085934 | -0.007367 | -4.252312 |
| N | -0.484204 | 0.687275 | -1.986586 |
| O | 1.045547 | 1.738604 | -3.153506 |
| C | 1.139635 | 2.126887 | -0.799005 |
| C | 2.261476 | 3.132276 | -1.016775 |
| H | 1.617176 | 1.081353 | -0.205891 |
| H | 0.394569 | 2.424156 | -0.053074 |

| | | | |
|---|---|---|---|
| C | 2.979595 | 3.443788 | 0.295834 |
| H | 2.982046 | 2.723905 | -1.735839 |
| H | 1.875096 | 4.062030 | -1.454036 |
| C | 4.103002 | 4.463426 | 0.126108 |
| H | 2.252203 | 3.819453 | 1.028299 |
| H | 3.388052 | 2.513040 | 0.712991 |
| H | 4.826279 | 4.083153 | -0.606408 |
| H | 3.690022 | 5.388072 | -0.296579 |
| C | 4.814614 | 4.771528 | 1.439711 |
| H | 5.616011 | 5.502695 | 1.300919 |
| H | 4.114764 | 5.178139 | 2.177219 |
| H | 5.257815 | 3.865745 | 1.866738 |

**IM5**

M062x/BSI SCF energy: -1904.283337 a.u.
M062x/BSII SCF energy in solution:
-1904.751857 a.u.
M062x/BSII free energy in solution:
-1904.384471 a.u.

| | | | |
|---|---|---|---|
| O | 1.824309 | 1.257678 | -0.869274 |
| C | 1.256654 | 2.280255 | -0.412587 |
| C | 1.974991 | 3.621989 | -0.706710 |
| F | 1.293602 | 4.325533 | -1.631453 |
| F | 3.222506 | 3.469705 | -1.167683 |
| F | 2.047597 | 4.391948 | 0.391158 |
| O | 0.196632 | 2.394040 | 0.216059 |
| C | 1.111066 | -0.364852 | 1.269477 |
| C | 1.264156 | -1.526121 | -1.189163 |
| C | 0.451558 | 0.159007 | 2.382080 |
| C | 2.450397 | -0.736031 | 1.338366 |
| C | 2.593356 | -1.720467 | -0.826465 |
| C | 1.137162 | 0.297356 | 3.579624 |
| H | -0.586448 | 0.465309 | 2.292999 |
| C | 3.137359 | -0.585914 | 2.542900 |
| C | 2.482495 | -0.072343 | 3.652251 |
| H | 0.631585 | 0.707415 | 4.446424 |
| H | 4.181223 | -0.878154 | 2.579167 |
| H | 3.026666 | 0.043106 | 4.583780 |
| S | 0.178265 | -0.497419 | -0.237277 |
| O | 3.178111 | -1.230889 | 0.299193 |
| C | 0.748376 | -2.084382 | -2.360131 |
| H | -0.293650 | -1.918929 | -2.618034 |
| C | 1.570900 | -2.845409 | -3.175871 |
| H | 1.175257 | -3.279443 | -4.086915 |
| C | 2.909010 | -3.035392 | -2.820359 |
| H | 3.560136 | -3.625821 | -3.456287 |
| C | 3.418467 | -2.482700 | -1.655864 |
| H | 4.451249 | -2.628984 | -1.358198 |
| C | -2.159900 | -3.425228 | 0.927507 |
| C | -0.734284 | -2.894176 | 0.872837 |
| C | -2.292632 | -1.182199 | 0.501136 |
| H | -2.360630 | -4.041386 | 1.803387 |
| H | -0.058812 | -3.541875 | 0.312137 |
| H | -0.330520 | -2.695216 | 1.872852 |
| H | -2.417347 | -3.970485 | 0.014388 |
| N | -0.992681 | -1.649640 | 0.141237 |
| O | -2.968141 | -2.241911 | 1.017289 |
| C | -2.799396 | 0.047070 | 0.381601 |
| C | -4.229777 | 0.375906 | 0.706079 |
| H | -2.155838 | 0.843453 | 0.016070 |
| C | -5.043734 | 0.760875 | -0.534931 |
| H | -4.703058 | -0.479162 | 1.200609 |
| H | -4.261661 | 1.210897 | 1.418525 |
| C | -6.485483 | 1.140447 | -0.204971 |
| H | -4.552142 | 1.600345 | -1.044627 |
| H | -5.036150 | -0.078698 | -1.242343 |
| H | -6.972959 | 0.299137 | 0.304101 |
| H | -6.484684 | 1.974830 | 0.508008 |
| C | -7.287618 | 1.524658 | -1.444724 |
| H | -8.319285 | 1.786595 | -1.192307 |
| H | -6.837585 | 2.385380 | -1.950492 |
| H | -7.318198 | 0.697774 | -2.162280 |

**TS5**

M062x/BSI SCF energy: -2306.248766 a.u.
M062x/BSII SCF energy in solution:
-2306.828953 a.u.
M062x/BSII free energy in solution:
-2306.315751 a.u.

| | | | |
|---|---|---|---|
| C | -1.317340 | -2.159861 | -0.103037 |
| C | -1.822487 | -0.387358 | 1.836101 |
| C | -1.561253 | -2.836538 | -1.311540 |
| C | -0.436157 | -2.715106 | 0.834703 |
| C | -0.868421 | -1.116036 | 2.553337 |
| C | -0.953991 | -4.052398 | -1.562227 |
| H | -2.240787 | -2.393243 | -2.034214 |

| | | | |
|---|---|---|---|
| C | 0.164888 | -3.953243 | 0.582285 |
| C | -0.093690 | -4.612282 | -0.606375 |
| H | -1.150333 | -4.572969 | -2.492731 |
| H | 0.828331 | -4.367873 | 1.334527 |
| H | 0.379920 | -5.569918 | -0.796771 |
| S | -2.048987 | -0.604873 | 0.109313 |
| O | -0.132626 | -2.147158 | 2.031798 |
| C | -2.551505 | 0.628341 | 2.469435 |
| H | -3.302087 | 1.164825 | 1.893446 |
| C | -2.302862 | 0.920047 | 3.803666 |
| H | -2.863326 | 1.708027 | 4.294470 |
| C | -1.330753 | 0.204856 | 4.507029 |
| H | -1.129014 | 0.436861 | 5.547570 |
| C | -0.622192 | -0.817859 | 3.889537 |
| H | 0.125881 | -1.397054 | 4.420060 |
| C | 0.547520 | 1.801557 | 1.193782 |
| C | -0.363081 | 1.881947 | -0.042838 |
| C | 0.867530 | 0.022502 | -0.158816 |
| H | -0.007985 | 1.667231 | 2.127048 |
| H | -0.016552 | 2.642909 | -0.751336 |
| H | -1.417150 | 2.081692 | 0.170924 |
| H | 1.217539 | 2.658758 | 1.285720 |
| N | -0.201163 | 0.587837 | -0.725974 |
| O | 1.344609 | 0.626198 | 0.964078 |
| C | 1.465845 | -1.126568 | -0.635808 |
| C | 2.639913 | -1.715795 | 0.054164 |
| H | 1.075330 | -1.547757 | -1.556978 |
| C | 3.291670 | -2.875709 | -0.688954 |
| H | 3.364140 | -0.904295 | 0.227093 |
| H | 2.325448 | -2.022615 | 1.067344 |
| C | 4.470162 | -3.469841 | 0.077002 |
| H | 2.544425 | -3.657063 | -0.874043 |
| H | 3.626085 | -2.520545 | -1.671254 |
| H | 5.209103 | -2.681748 | 0.271126 |
| H | 4.122759 | -3.816932 | 1.058759 |
| C | 2.601018 | 3.453991 | -1.121102 |
| C | 2.565930 | 2.291338 | -1.892914 |
| C | 3.509009 | 1.276747 | -1.715876 |
| C | 4.497283 | 1.451539 | -0.733307 |
| C | 4.532679 | 2.601102 | 0.040604 |
| C | 3.582627 | 3.612108 | -0.142140 |
| H | 1.858810 | 4.224564 | -1.297352 |
| H | 1.792656 | 2.173387 | -2.648412 |
| H | 5.245008 | 0.675645 | -0.586173 |
| H | 5.296978 | 2.737117 | 0.799217 |
| N | 3.423343 | 0.081587 | -2.438372 |
| H | 2.955614 | 0.176984 | -3.332226 |
| H | 4.312560 | -0.391552 | -2.550317 |
| O | 3.697922 | 4.700664 | 0.670402 |
| C | 2.747211 | 5.733784 | 0.501427 |
| H | 1.725635 | 5.374747 | 0.678059 |
| H | 2.990586 | 6.498320 | 1.239968 |
| H | 2.804445 | 6.171677 | -0.502401 |
| C | 5.129386 | -4.621839 | -0.673907 |
| H | 5.506348 | -4.289876 | -1.646990 |
| H | 5.970928 | -5.037821 | -0.112949 |
| H | 4.413599 | -5.430854 | -0.853965 |
| O | -3.498340 | 2.021165 | -0.352990 |
| O | -5.092415 | 0.628673 | 0.449824 |
| C | -4.817033 | 0.852180 | -1.895285 |
| F | -4.928331 | 1.935473 | -2.682238 |
| F | -5.960522 | 0.163295 | -2.008900 |
| F | -3.845473 | 0.082092 | -2.440107 |
| C | -4.452010 | 1.217942 | -0.430527 |

**IM6**

M062x/BSI SCF energy: -844.891304 a.u.
M062x/BSII SCF energy in solution:
-845.106955 a.u.
M062x/BSII free energy in solution:
-844.768735 a.u.

| | | | |
|---|---|---|---|
| C | 0.457002 | 2.375609 | -1.505754 |
| C | 0.804620 | 3.428966 | -0.432574 |
| C | 1.189600 | 1.428573 | 0.375517 |
| H | 1.026370 | 2.479401 | -2.429820 |
| H | -0.045599 | 4.067478 | -0.182497 |
| H | 1.635873 | 4.074141 | -0.729243 |
| H | -0.610488 | 2.326855 | -1.733382 |
| N | 1.202090 | 2.640337 | 0.745786 |
| O | 0.831273 | 1.120888 | -0.884978 |
| C | 1.486499 | 0.267520 | 1.288734 |
| C | 1.962684 | -0.988375 | 0.578758 |
| H | 2.214527 | 0.601092 | 2.031724 |
| C | 3.318697 | -0.758011 | -0.090504 |
| H | 2.048338 | -1.794288 | 1.318502 |
| H | 1.224720 | -1.293873 | -0.170002 |
| C | 3.854794 | -2.022007 | -0.758591 |

| | | | |
|---|---|---|---|
| H | 3.223226 | 0.036068 | -0.841528 |
| H | 4.040362 | -0.404917 | 0.657366 |
| H | 3.955345 | -2.813507 | -0.005776 |
| H | 3.123103 | -2.379734 | -1.493242 |
| C | -2.920700 | 0.384620 | 0.094085 |
| C | -1.795166 | 0.689138 | 0.854711 |
| C | -0.982094 | -0.334785 | 1.314126 |
| C | -1.263890 | -1.669527 | 1.036202 |
| C | -2.382187 | -1.974711 | 0.280000 |
| C | -3.215507 | -0.951982 | -0.196859 |
| H | -3.556479 | 1.188838 | -0.254710 |
| H | -1.563017 | 1.725067 | 1.089593 |
| H | -0.620692 | -2.460146 | 1.411462 |
| H | -2.634697 | -3.003471 | 0.048215 |
| N | 0.216765 | 0.003671 | 2.091864 |
| H | 0.030068 | 0.840727 | 2.660265 |
| H | 0.420019 | -0.750882 | 2.760845 |
| O | -4.279017 | -1.352992 | -0.919563 |
| C | -5.162173 | -0.354274 | -1.410790 |
| H | -4.643509 | 0.333901 | -2.086995 |
| H | -5.940157 | -0.884177 | -1.959358 |
| H | -5.616315 | 0.209040 | -0.588449 |
| C | 5.198338 | -1.785190 | -1.440808 |
| H | 5.948718 | -1.445289 | -0.719407 |
| H | 5.574186 | -2.698354 | -1.910313 |
| H | 5.112963 | -1.018581 | -2.217982 |

**4a**

M062x/BSI SCF energy: -844.440921 a.u.
M062x/BSII SCF energy in solution:
-844.663331 a.u.
M062x/BSII free energy in solution:
-844.33858 a.u.

| | | | |
|---|---|---|---|
| C | 0.294494 | 2.215906 | -1.509726 |
| C | 0.686415 | 3.323360 | -0.511825 |
| C | 1.148079 | 1.363883 | 0.374405 |
| H | 0.780096 | 2.301789 | -2.482959 |
| H | -0.156494 | 3.973068 | -0.263149 |
| H | 1.497781 | 3.956696 | -0.884409 |
| H | -0.786980 | 2.128181 | -1.646593 |
| N | 1.138577 | 2.596800 | 0.684425 |
| O | 0.760214 | 1.008237 | -0.872791 |
| C | 1.449201 | 0.227317 | 1.324501 |
| C | 2.088453 | -0.965447 | 0.621420 |
| H | 2.148921 | 0.621429 | 2.067658 |
| C | 3.435426 | -0.622740 | -0.012557 |
| H | 2.213129 | -1.760284 | 1.366038 |
| H | 1.409101 | -1.342192 | -0.150923 |
| C | 4.097499 | -1.833565 | -0.667498 |
| H | 3.296288 | 0.162958 | -0.767215 |
| H | 4.107853 | -0.209566 | 0.751514 |
| H | 4.238585 | -2.617977 | 0.086668 |
| H | 3.418020 | -2.248342 | -1.422524 |
| C | -3.062623 | 0.313519 | 0.344859 |
| C | -1.909945 | 0.576731 | 1.087402 |
| C | -0.938306 | -0.402259 | 1.294115 |
| C | -1.140200 | -1.670698 | 0.734030 |
| C | -2.267831 | -1.937693 | -0.026186 |
| C | -3.239049 | -0.948280 | -0.224991 |
| H | -3.801100 | 1.095866 | 0.215592 |
| H | -1.765465 | 1.565847 | 1.516701 |
| H | -0.408254 | -2.452016 | 0.914002 |
| H | -2.428363 | -2.917642 | -0.464471 |
| N | 0.227996 | -0.139266 | 2.076312 |
| H | 0.024556 | 0.635930 | 2.702772 |
| O | -4.315184 | -1.311987 | -0.971513 |
| C | -5.324026 | -0.340303 | -1.174911 |
| H | -4.935709 | 0.534374 | -1.709965 |
| H | -6.092455 | -0.820451 | -1.781352 |
| H | -5.765016 | -0.015588 | -0.225079 |
| C | 5.437023 | -1.493194 | -1.312572 |
| H | 6.140385 | -1.099175 | -0.571385 |
| H | 5.895776 | -2.372669 | -1.773756 |
| H | 5.315424 | -0.732194 | -2.090703 |
| H | 5.112963 | -1.018581 | -2.217982 |

# 7.References


[1] J. C. Dyer, S. A. Evans, *J. Org. Chem.*, 1980, **45**, 5350.

[2] M. Hanit, A. Svetlana, P. Yanay, G. Michael, *J. Org. Chem.*, 2011, **76**, 5240.

[3] Z. Tian, C. Zhang, *Chem. Commun.*, 2019, **55**, 11936.

[4] A. S. Touchy, S. M. A. H. Siddiko, W. Onodera, K. Kon, K. I. Shimizu, *Green Chem.*, 2016, **18**, 2554.

[5] A. K. Clarke, A. Parkin, R. J. K. Taylor, W. P. Unsworth, A. J. A. Rossi, *ACS Catal.*, 2020, **10**, 5814.

[6] J. Tian, F. Luo, Q. Zhang, Y. Liang, D. Li, Y. Zhan, L. Kong, Z. Wang, B. Peng B, *J. Am. Chem. Soc.*, 2020, **142**, 6884.

[7] M. C. Sheikhm, T. Iwasawa, A. Nakajima, A. Kitao, N. Tsubaki, R. Miyatake, T. Yoshimura, H. Morita, *Synthesis.*, 2014, **46**, 42.

[8] X. Huang, W. Zhao, D. Chen, Y. Zhan, T. Zeng, H. Jin, B. Peng, *Chem. Commun.*, 2019, **57**, 2070.

[9] (a) Y. Zhao and D. G. Truhlar, *Theor. Chem. Acc.*, **2008**, **120**, 215; (b) Y. Zhao and D. G. Truhlar, *Acc. Chem. Res.*, **2008**, 41, 157; (c) R. Valero, R. Costa, I. D. P. R. Moreira, D. G. Truhlar and F. Illas, J. *Chem. Phys.*, **2008**, **128**, 114103.

[10] A. V. Marenich, C. J. Cramer, D. G. Truhlar, *J. Phys. Chem. B.*, **2009**, **113**, 6378.

[11] Gaussian 09, Revision A.01, M. J. Frisch, G. W. Trucks, H. B. Schlegel, G. E. Scuseria, M. A. Robb, J. R. Cheeseman, G. Scalmani, V. Barone, B. Mennucci, G. A. Petersson, H. Nakatsuji, M. Caricato, X. Li, H. P. Hratchian, A. F. Izmaylov, J. Bloino, G. Zheng, J. L. Sonnenberg, M. Hada, M. Ehara, K. Toyota, R. Fukuda, J. Hasegawa, M. Ishida, T. Nakajima, Y. Honda, O. Kitao, H. Nakai, T. Vreven, J. A. Montgomery, Jr. J. E. Peralta, F. Ogliaro, M. Bearpark, J. J. Heyd, E. Brothers, K. N. Kudin, V. N. Staroverov, T. Keith, R. Kobayashi, J. Normand, K. Raghavachari, A. Rendell, J. C. Burant, S. S. Iyengar, J. Tomasi, M. Cossi, N. Rega, J. M. Millam, M. Klene, J. E. Knox, J. B. Cross, V. Bakken, C. Adamo, J. Jaramillo, R. Gomperts, R. E. Stratmann, O. Yazyev, A. J. Austin, R. Cammi, C. Pomelli, J. W. Ochterski, R. L. Martin, K. Morokuma, V. G. Zakrzewski, G. A. Voth, P. Salvador, J. J. Dannenberg, S. Dapprich, A. D. Daniels, O. Farkas, J. B. Foresman, J. V. Ortiz, J. Cioslowski, and D. J. Fox, Gaussian, Inc., Wallingford CT, 2009.

# 8. NMR spectra

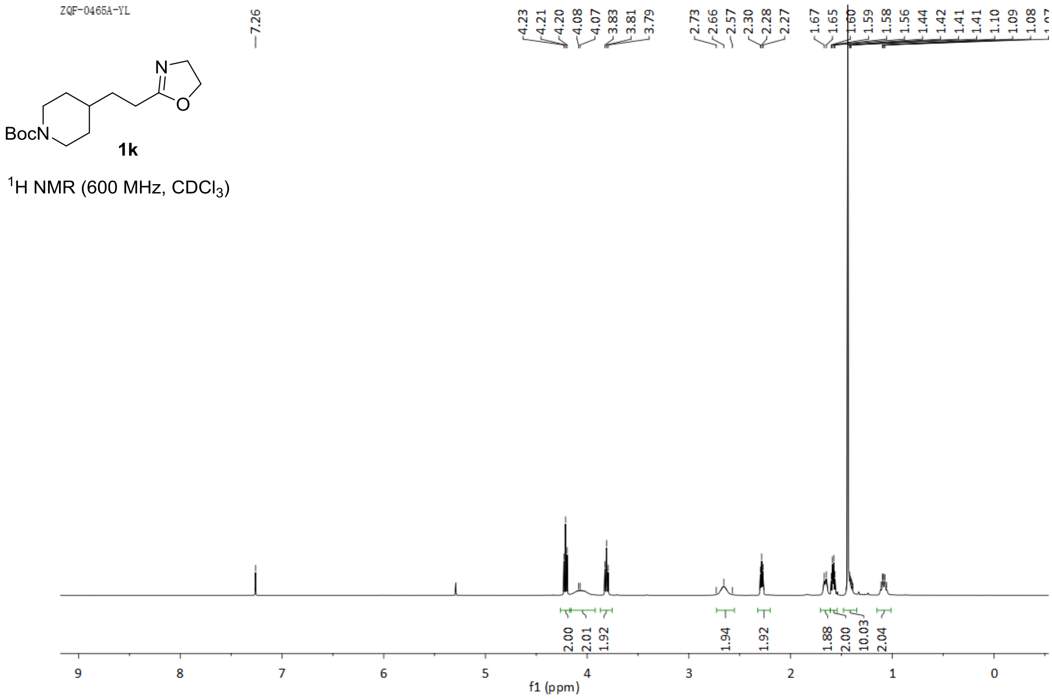


**1k**

$^{1}H$ NMR (600 MHz, $CDCl_3$)

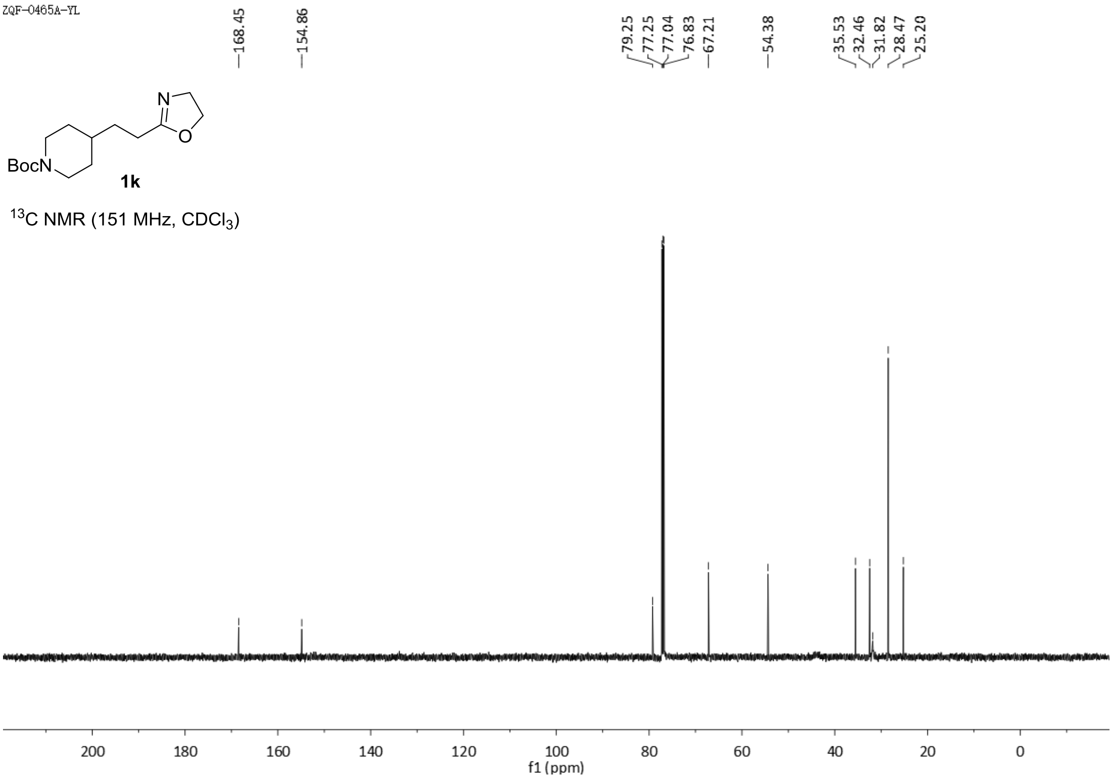


**1k**

$^{13}C$ NMR (151 MHz, $CDCl_3$)

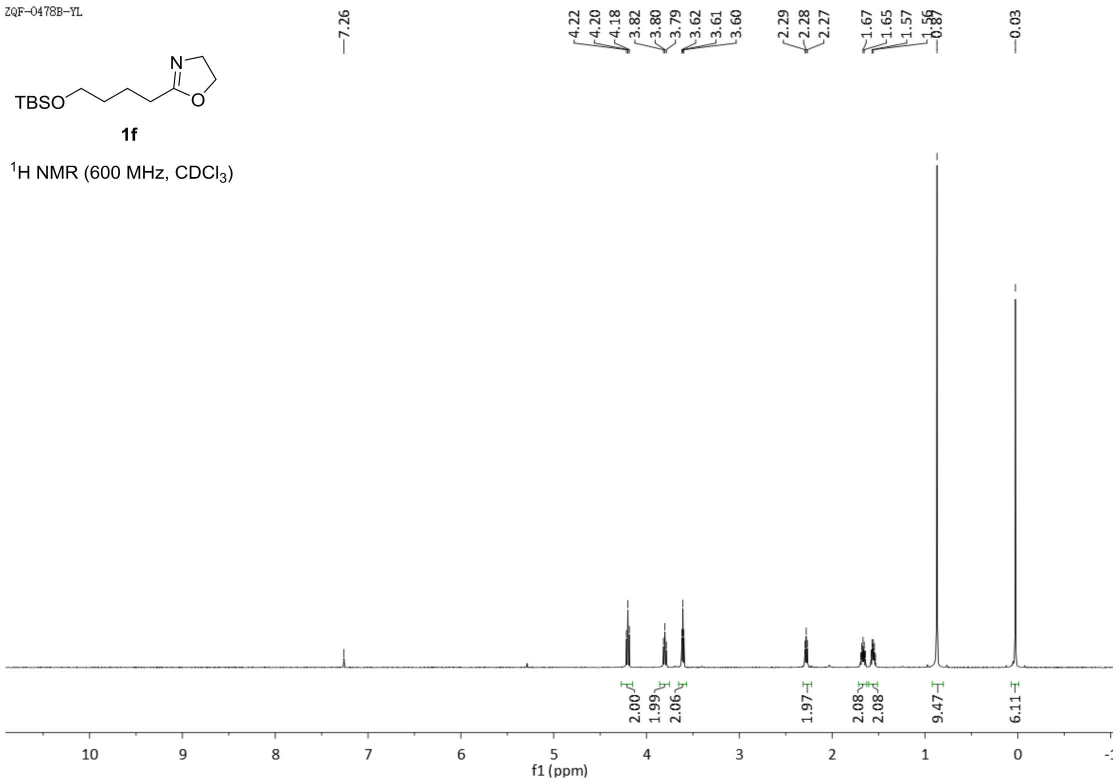


1f

$^{1}H$ NMR (600 MHz, $CDCl_3$)

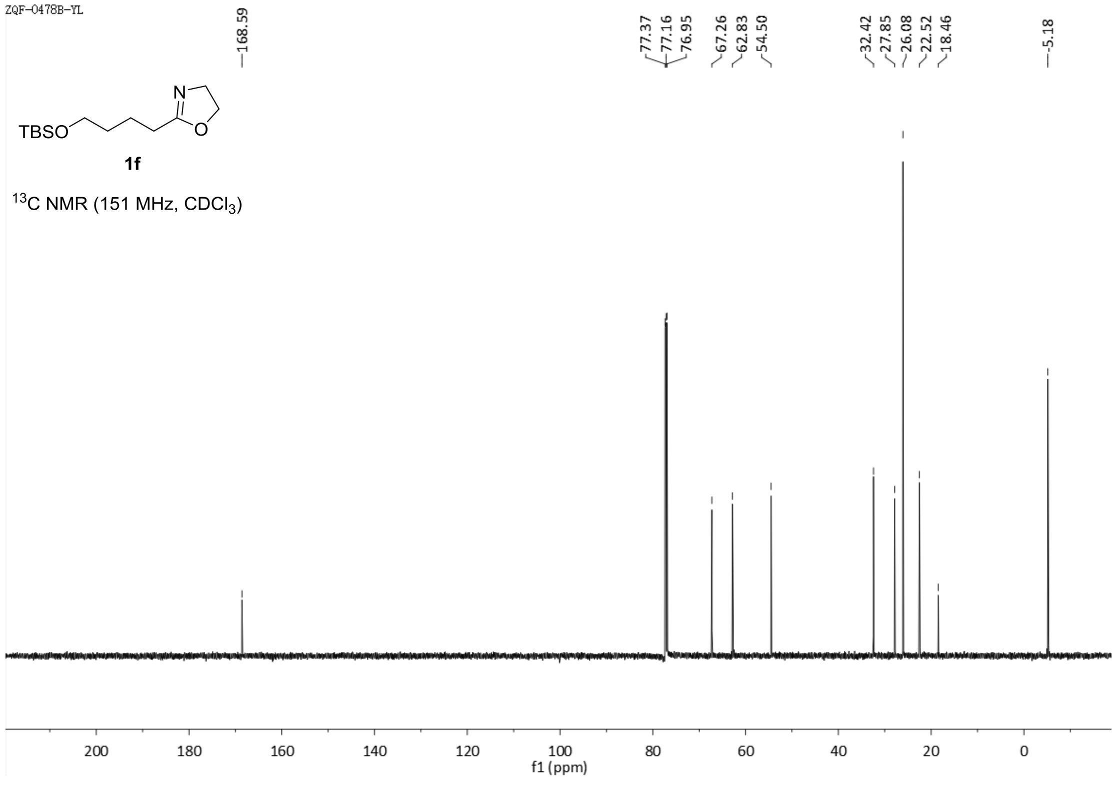


1f

$^{13}C$ NMR (151 MHz, $CDCl_3$)

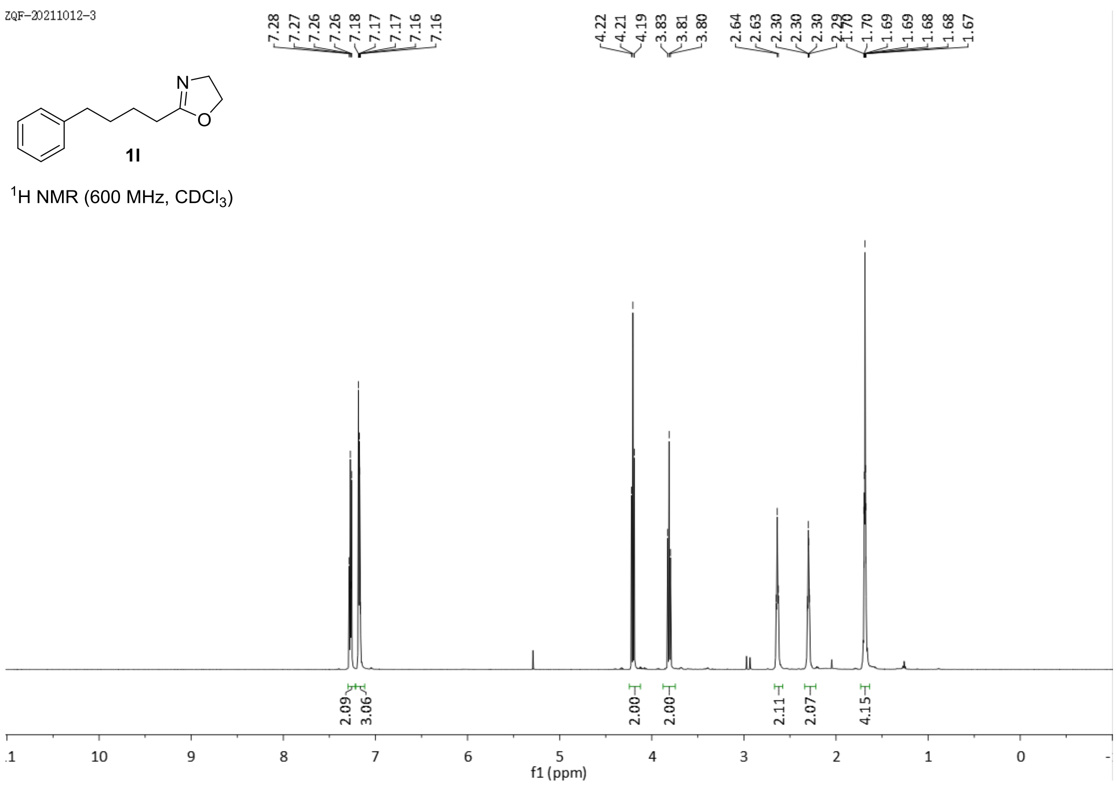
ZQF-20211012-3
7.28 7.27 7.26 7.26 7.18 7.17 7.17 7.16 7.16
4.22 4.21 4.19 3.83 3.81 3.80
2.64 2.63 2.30 2.30 2.30 2.29 1.70 1.70 1.69 1.69 1.68 1.68 1.67
N
O
1l
$^1$H NMR (600 MHz, CDCl$_3$)
2.09 3.06 2.00 2.00 2.11 2.07 4.15
f1 (ppm)

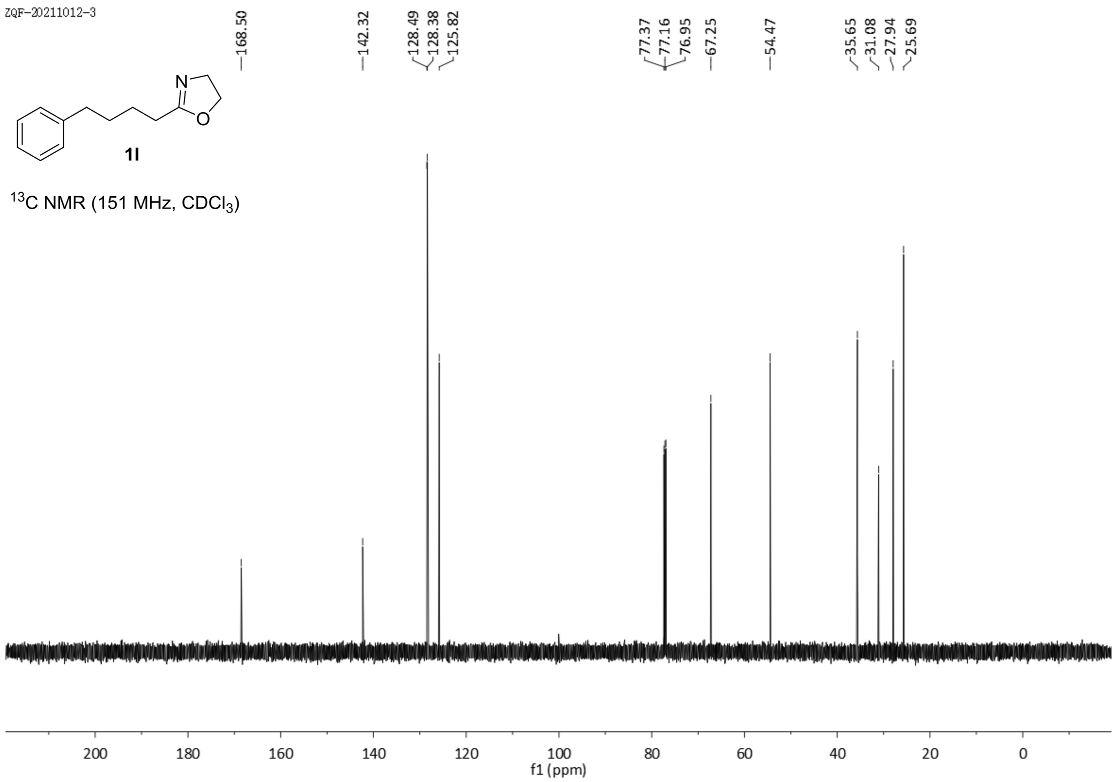
ZQF-20211012-3
168.50
142.32
128.49 128.38 125.82
77.37 77.16 76.95 67.25
54.47
35.65 31.08 27.94 25.69
N
O
1l
$^{13}$C NMR (151 MHz, CDCl$_3$)
f1 (ppm)

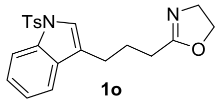

**1o**

$^1$H NMR (600 MHz, $CDCl_3$)

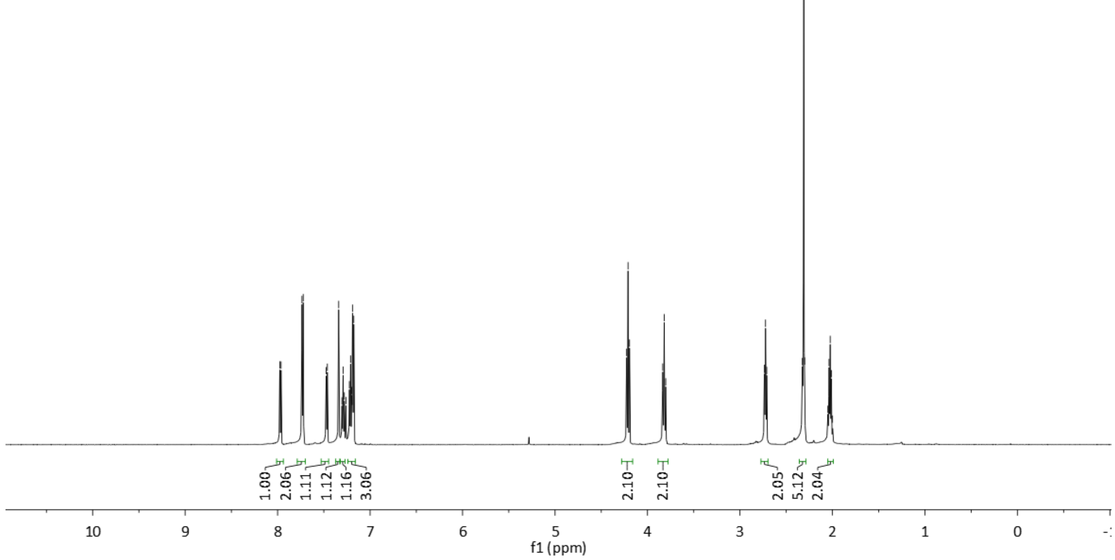


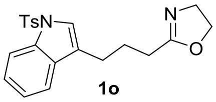

**1o**

$^{13}$C NMR (151 MHz, $CDCl_3$)

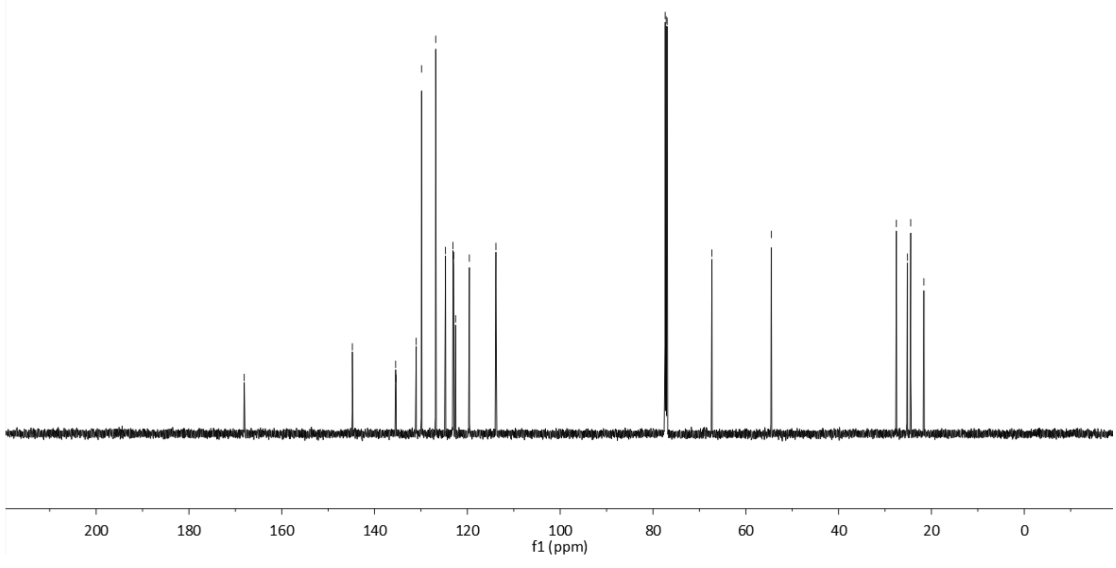

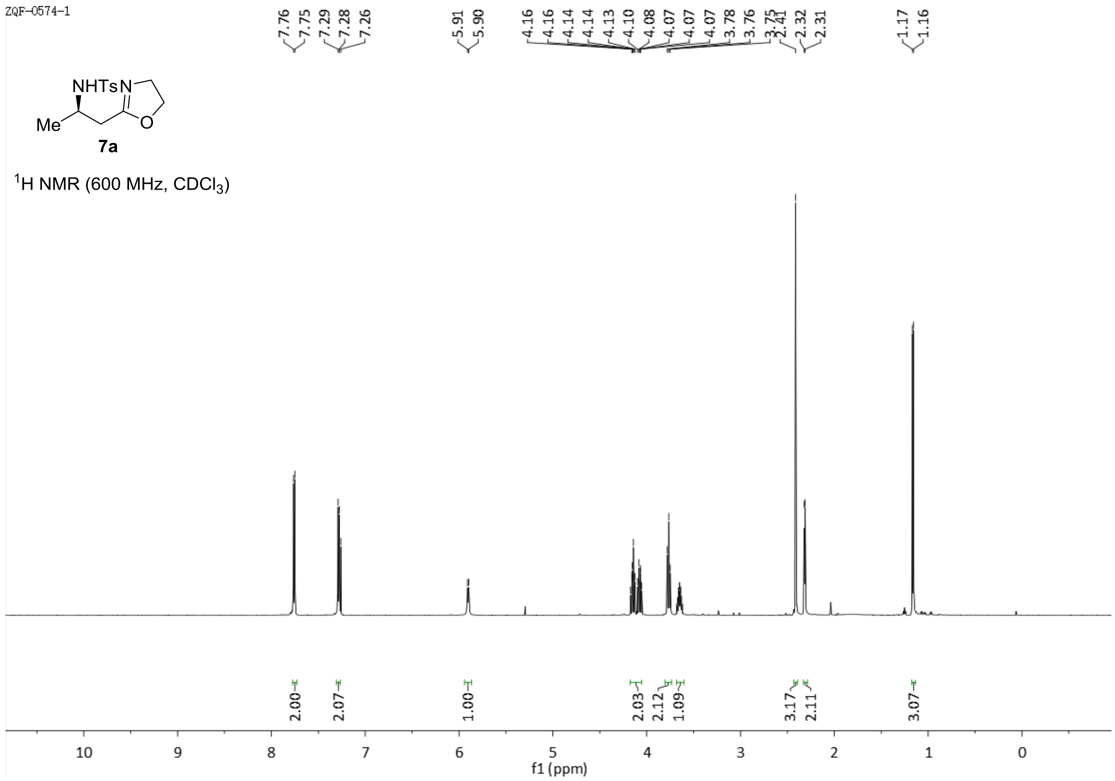
ZQF-0574-1
7.76
7.75
7.29
7.28
7.26
5.91
5.90
4.16
4.16
4.14
4.14
4.13
4.10
4.08
4.07
4.07
4.07
3.78
3.76
3.75
2.41
2.32
2.31
1.17
1.16
NHTs
N
Me
O
7a
$^1$H NMR (600 MHz, $CDCl_3$)
2.00
2.07
1.00
2.03
2.12
1.09
3.17
2.11
3.07
f1 (ppm)

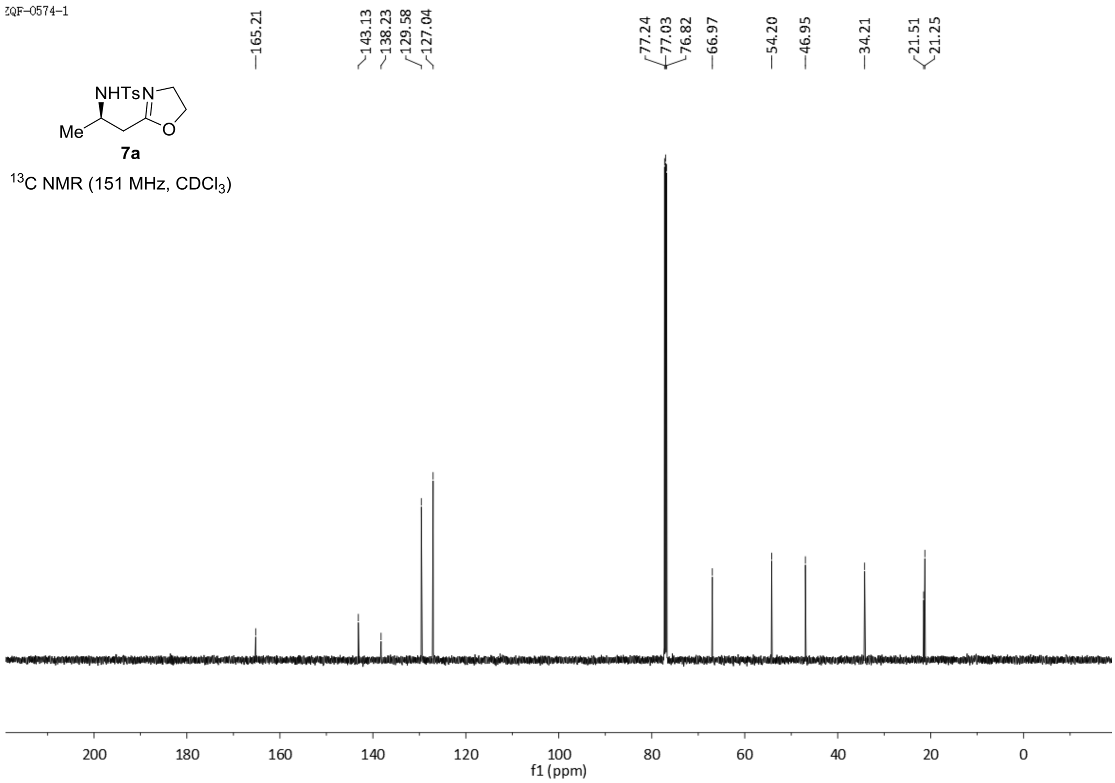
ZQF-0574-1
165.21
143.13
138.23
129.58
127.04
77.24
77.03
76.82
66.97
54.20
46.95
34.21
21.51
21.25
NHTs
N
Me
O
7a
$^{13}$C NMR (151 MHz, $CDCl_3$)
f1 (ppm)

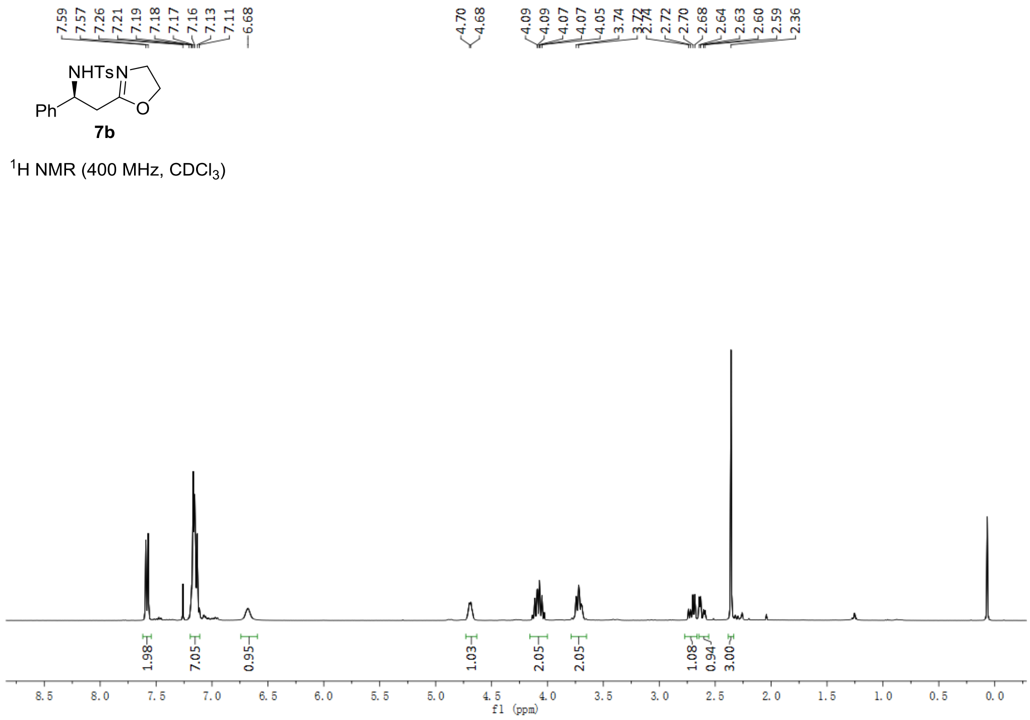


**7b**

$^{1}$H NMR (400 MHz, $CDCl_3$)

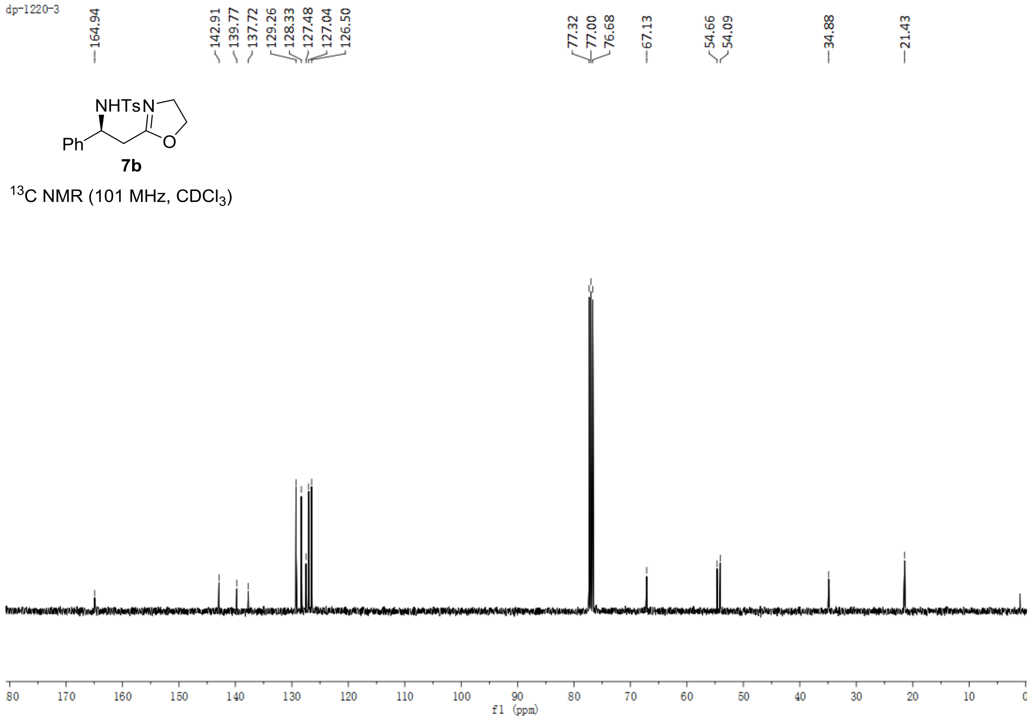


**7b**

$^{13}$C NMR (101 MHz, $CDCl_3$)

dp-1208-3

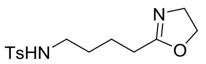

**7c**

$^{1}$H NMR (400 MHz, $CDCl_3$)

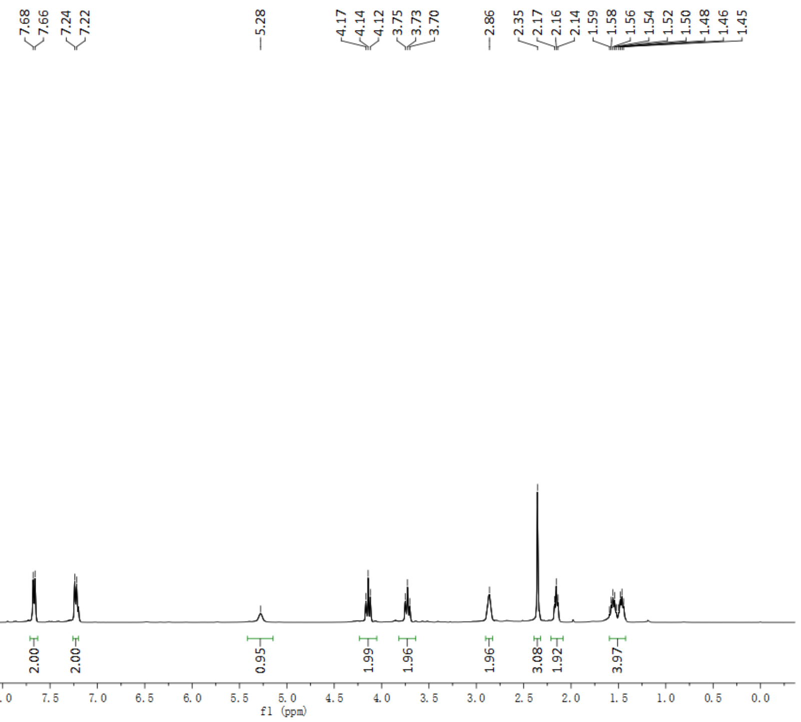


dp-1208-3

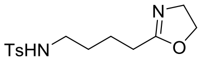

**7c**

$^{13}$C NMR (101 MHz, $CDCl_3$)

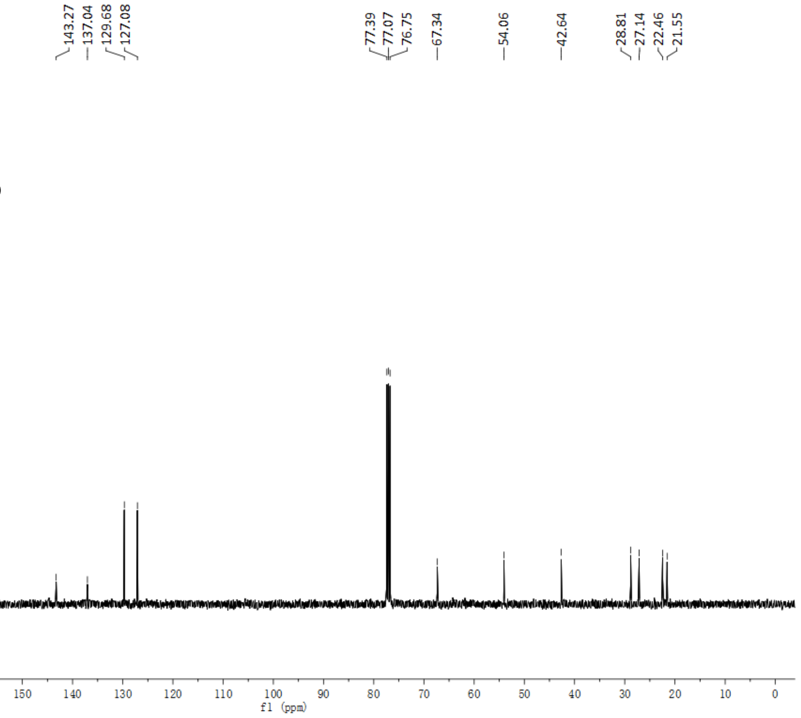

dp-1208-6

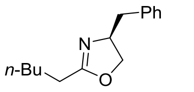


**9**

$^{1}H$ NMR (400 MHz, $CDCl_3$)

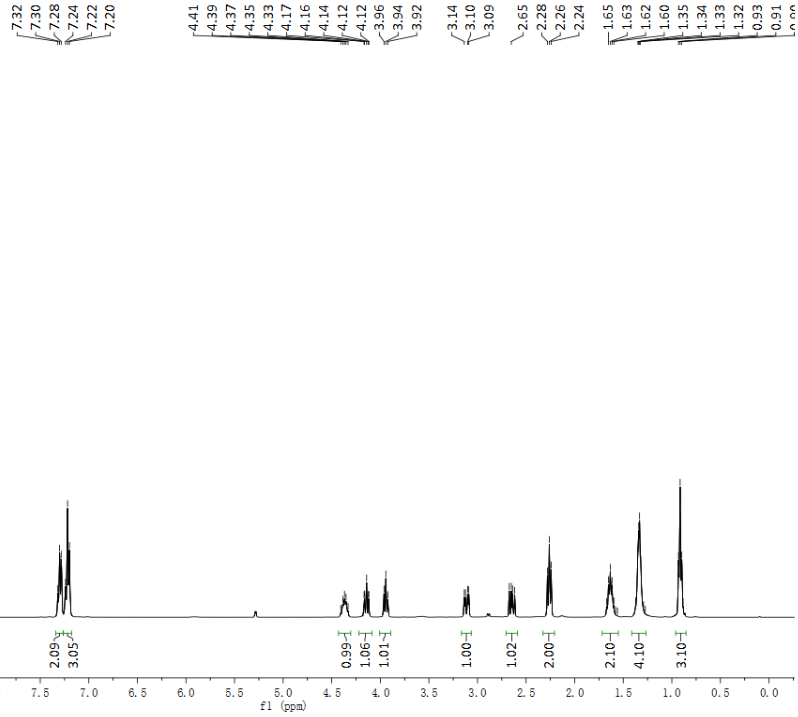


dp-1208-6

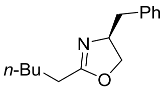


**9**

$^{13}C$ NMR (101 MHz, $CDCl_3$)

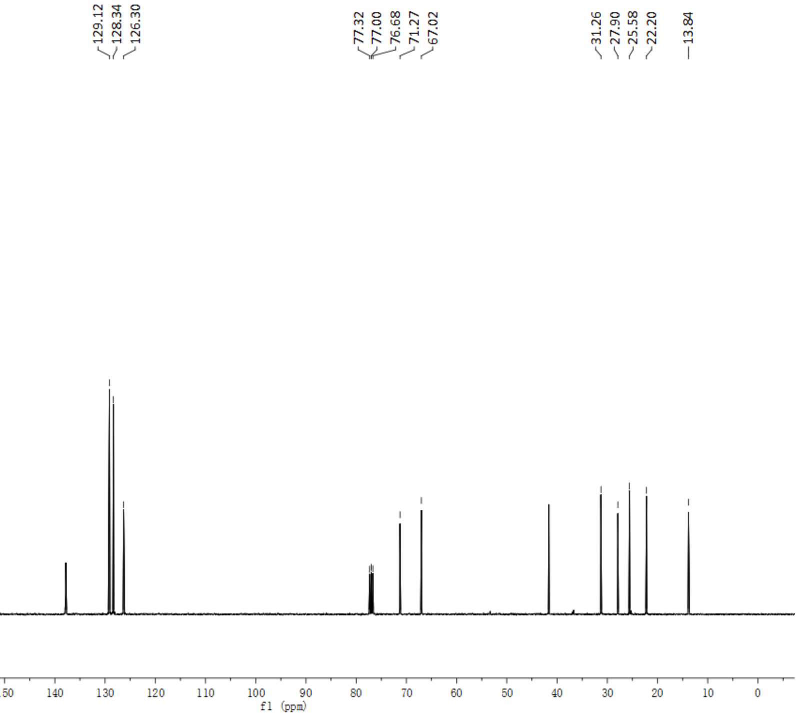

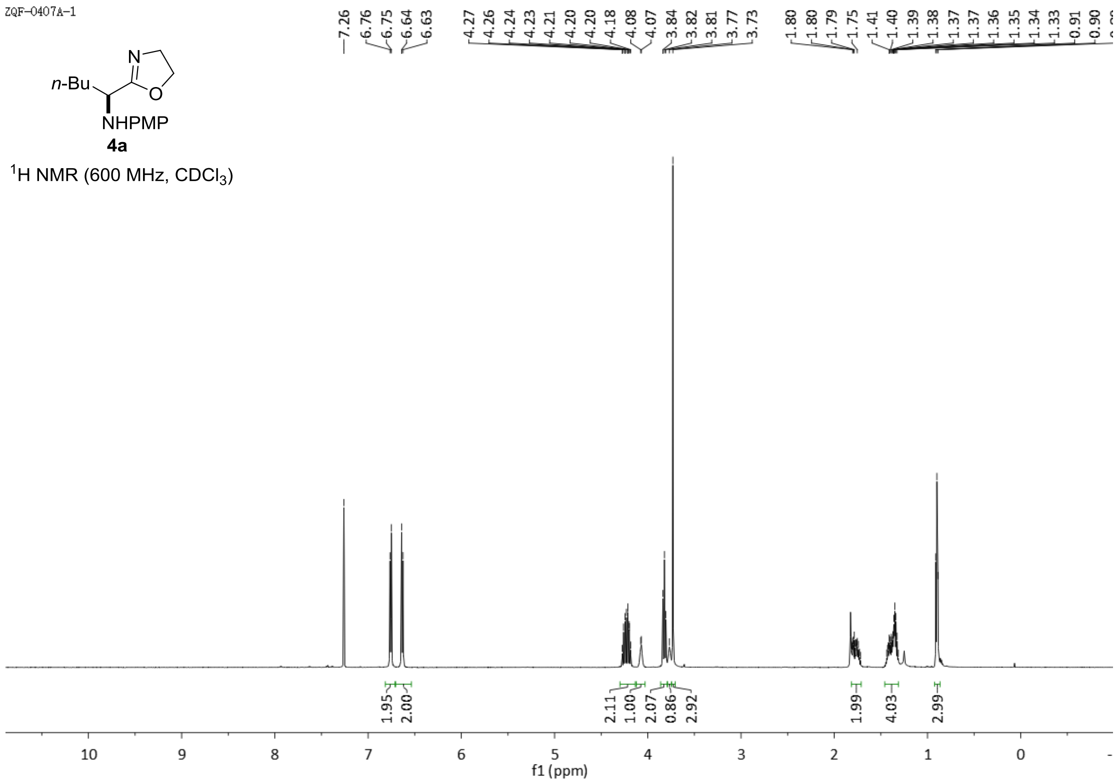
ZQF-0407A-1
7.26
6.76
6.75
6.64
6.63
4.27
4.26
4.24
4.23
4.21
4.20
4.20
4.18
4.08
4.07
3.84
3.82
3.81
3.77
3.73
1.80
1.80
1.79
1.75
1.41
1.40
1.39
1.38
1.37
1.37
1.36
1.35
1.34
1.33
0.91
0.90
N
O
n-Bu
NHPMP
4a
1H NMR (600 MHz, CDCl3)
1.95
2.00
2.11
1.00
2.07
0.86
2.92
1.99
4.03
2.99
10
9
8
7
6
5
4
3
2
1
0
f1 (ppm)

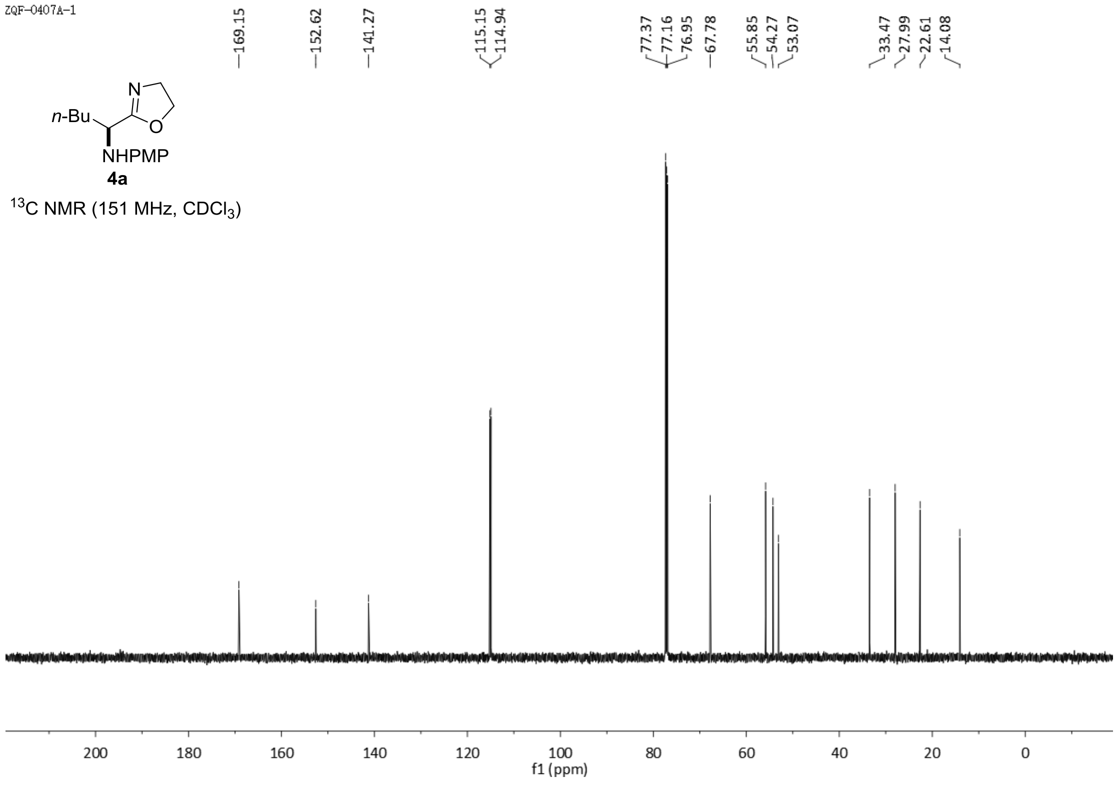
ZQF-0407A-1
169.15
152.62
141.27
115.15
114.94
77.37
77.16
76.95
67.78
55.85
54.27
53.07
33.47
27.99
22.61
14.08
N
O
n-Bu
NHPMP
4a
13C NMR (151 MHz, CDCl3)
200
180
160
140
120
100
80
60
40
20
0
f1 (ppm)

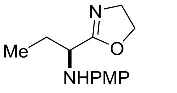

**4b**

$^1H$ NMR (600 MHz, $CDCl_3$)

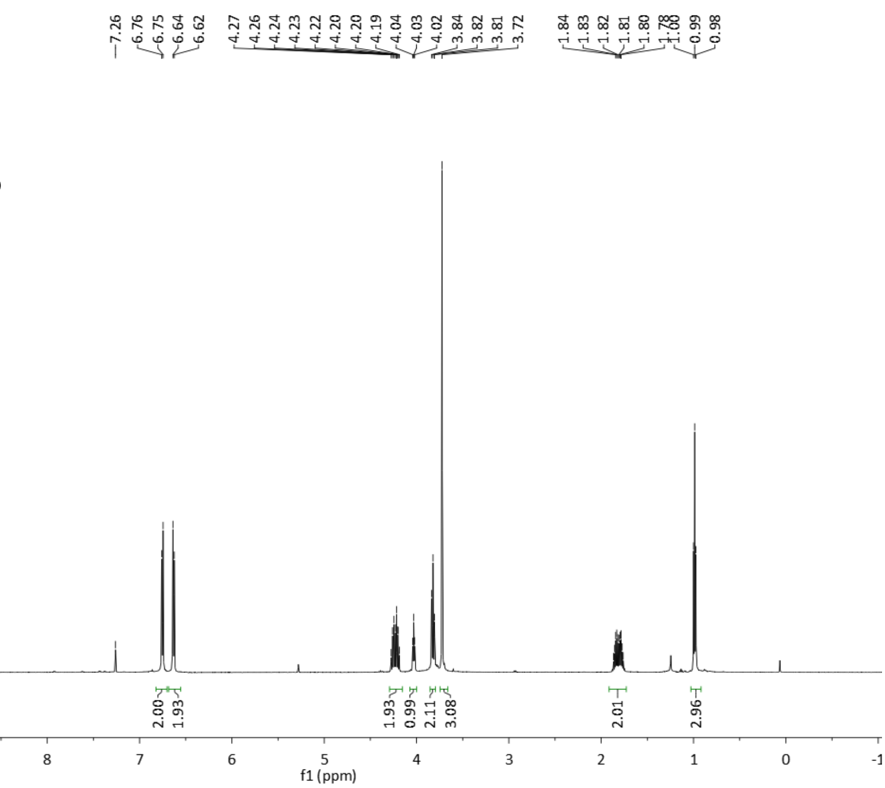


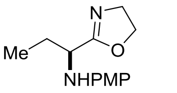

**4b**

$^{13}C$ NMR (151 MHz, $CDCl_3$)

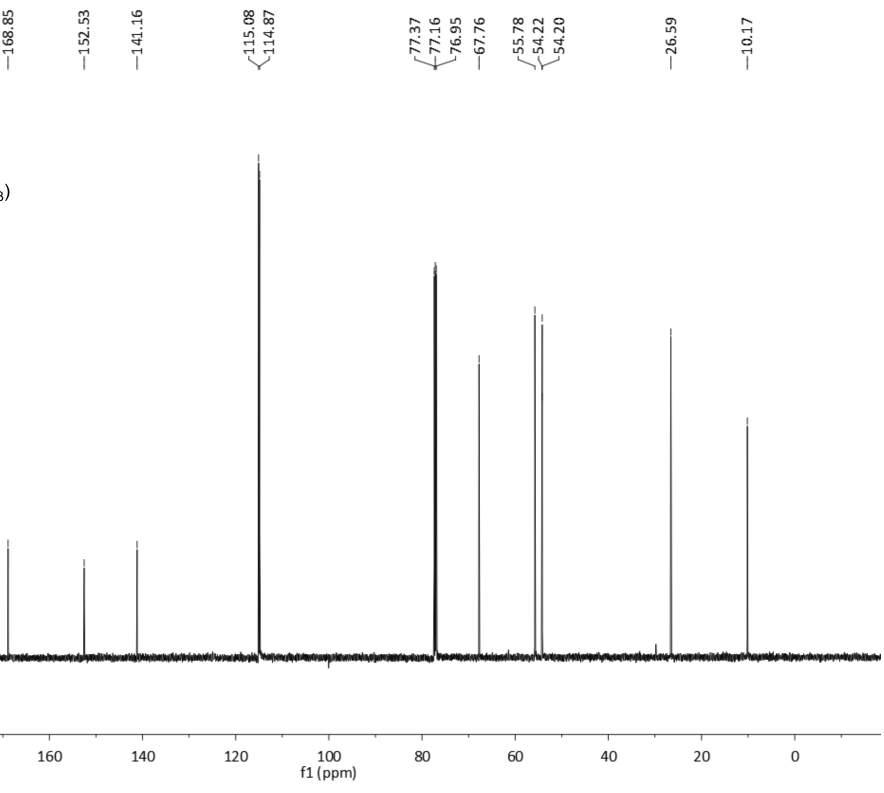

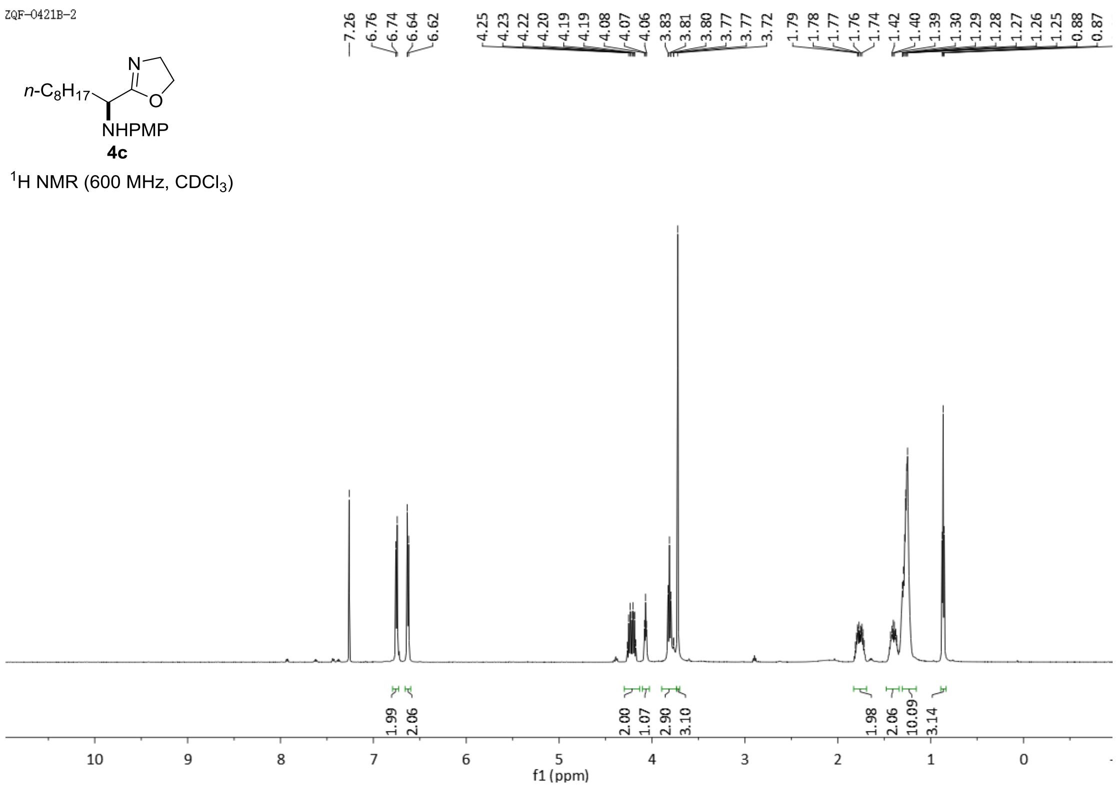


**4c**

$^{1}H$ NMR (600 MHz, $CDCl_3$)

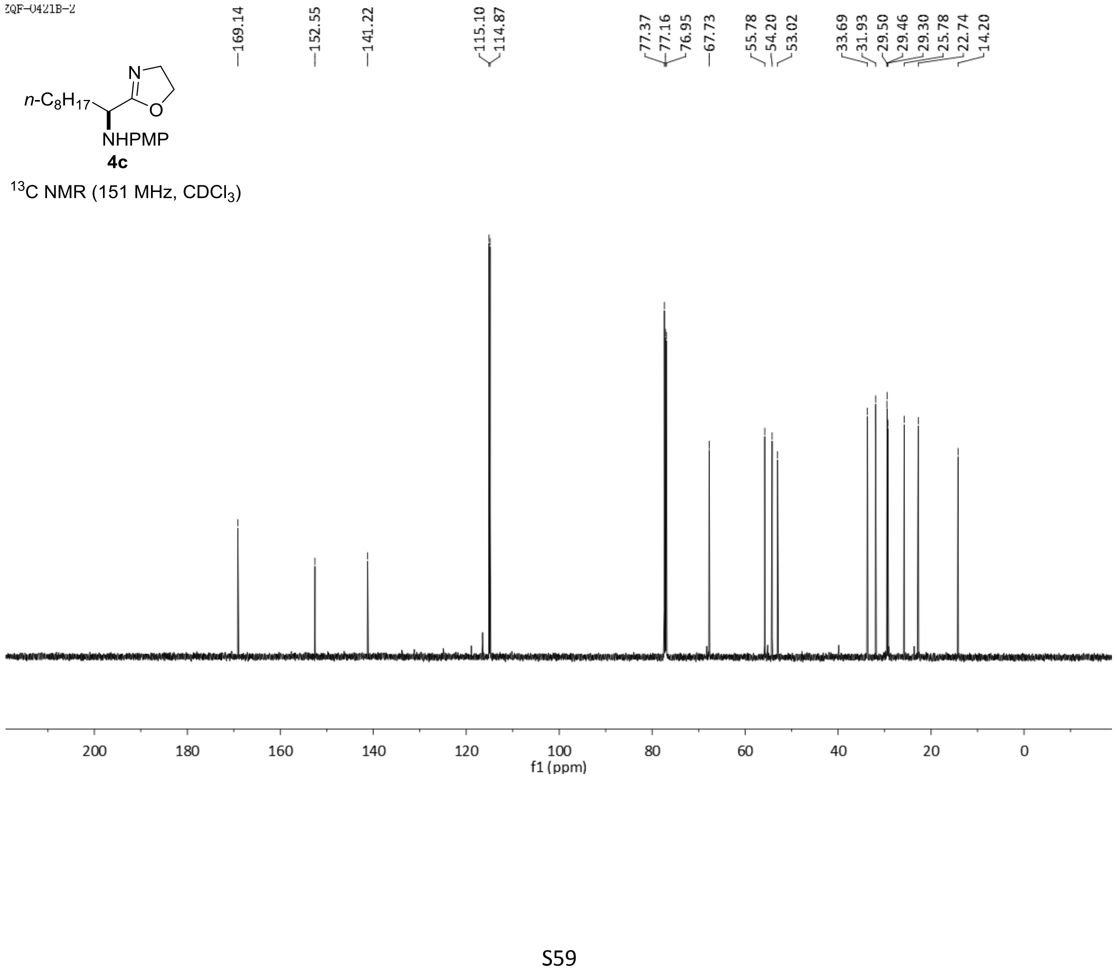


**4c**

$^{13}C$ NMR (151 MHz, $CDCl_3$)

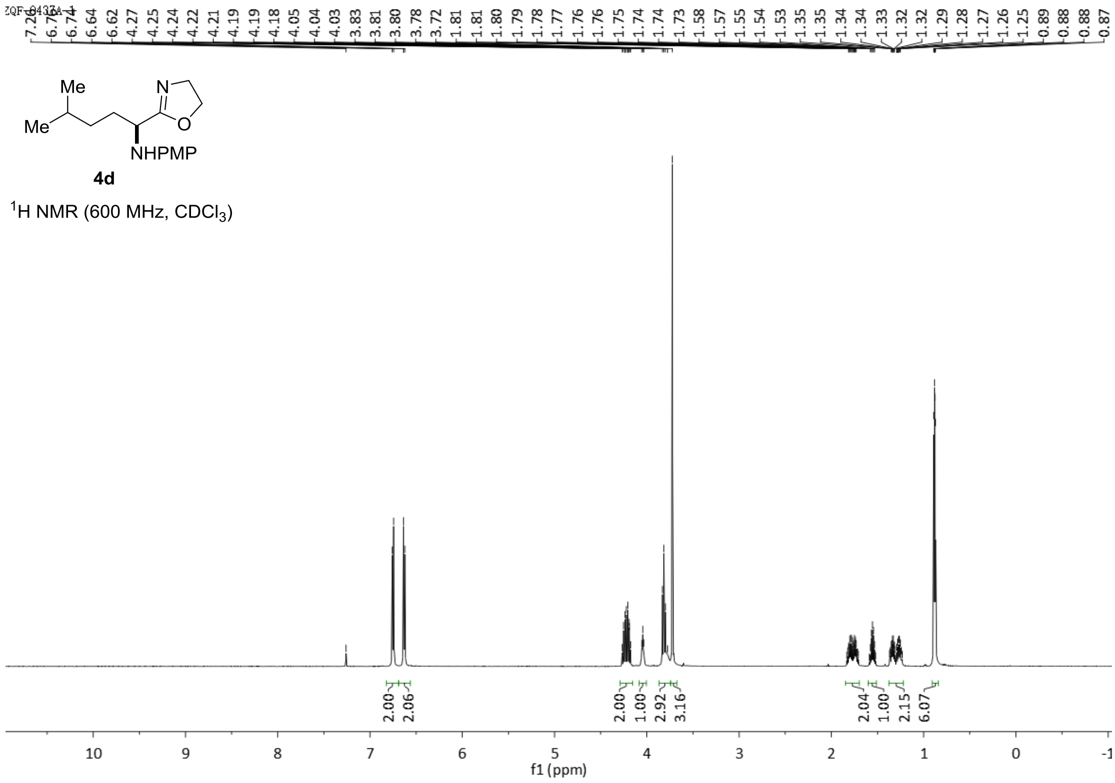

**4d**

$^{1}H$ NMR (600 MHz, $CDCl_3$)

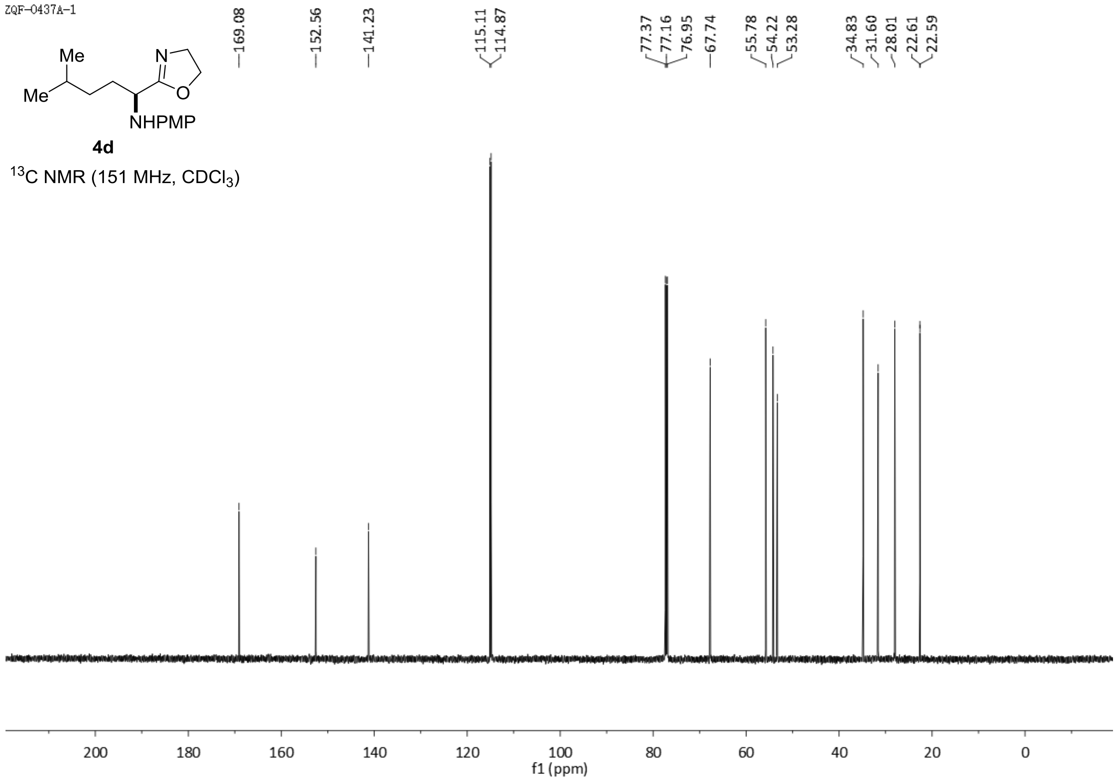

**4d**

$^{13}C$ NMR (151 MHz, $CDCl_3$)

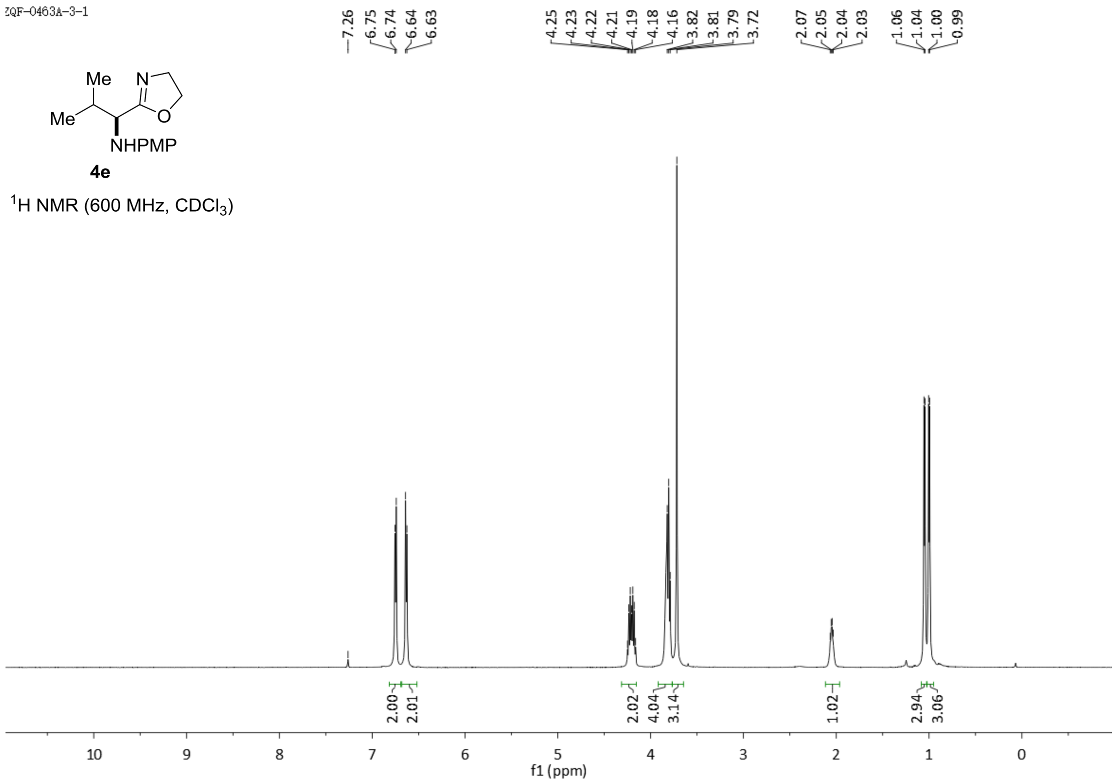


**4e**

$^{1}$H NMR (600 MHz, $CDCl_3$)

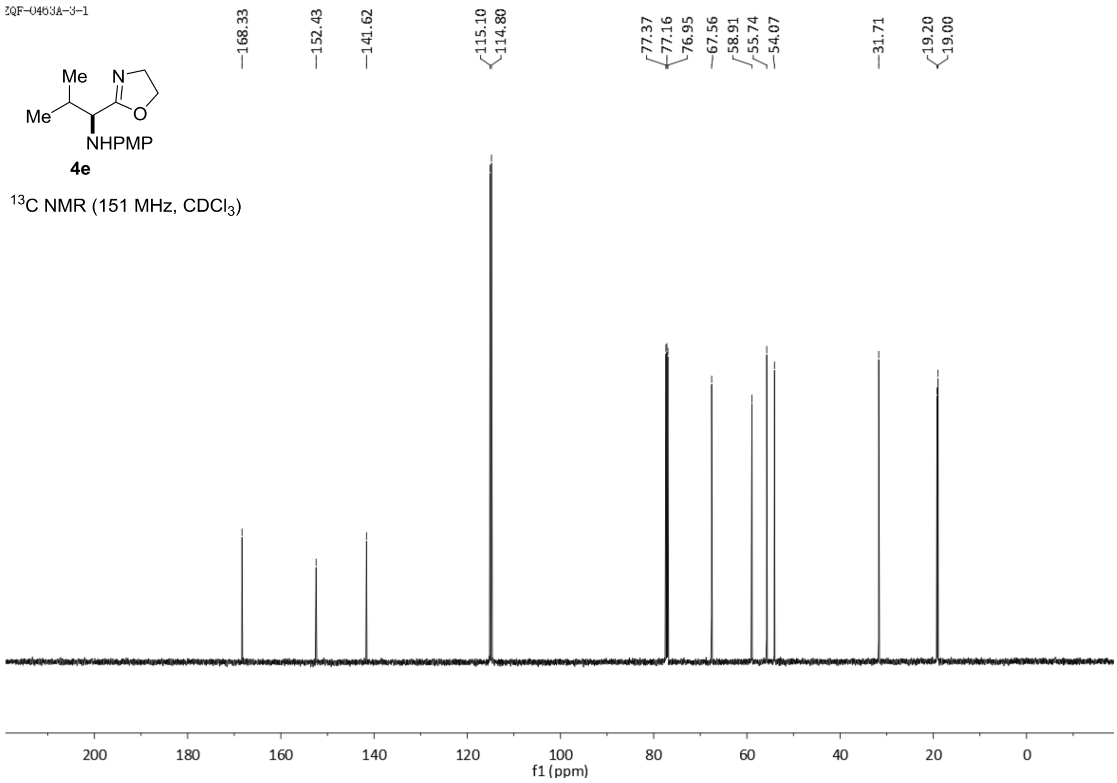


**4e**

$^{13}$C NMR (151 MHz, $CDCl_3$)

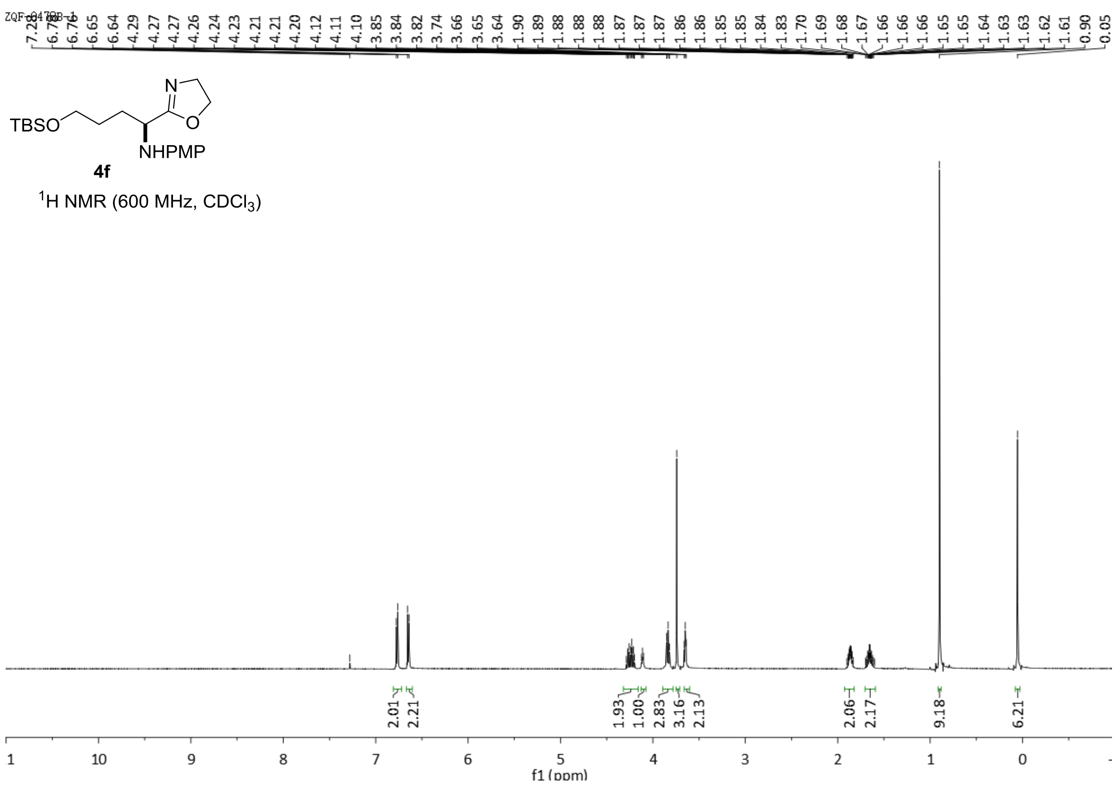
ZQF-0478B-1
6.65 6.64 4.29 4.27 4.27 4.26 4.24 4.23 4.21 4.21 4.20 4.12 4.11 4.10 3.85 3.84 3.82 3.74 3.66 3.65 3.64 1.90 1.89 1.88 1.88 1.88 1.87 1.87 1.87 1.86 1.86 1.85 1.85 1.84 1.83 1.70 1.69 1.68 1.67 1.66 1.66 1.66 1.65 1.65 1.64 1.63 1.63 1.62 1.61 0.90 0.05
TBSO
N
O
NHPMP
4f
$^{1}$H NMR (600 MHz, $CDCl_3$)
2.01 2.21 1.93 1.00 2.83 3.16 2.13 2.06 2.17 9.18 6.21
f1 (ppm)

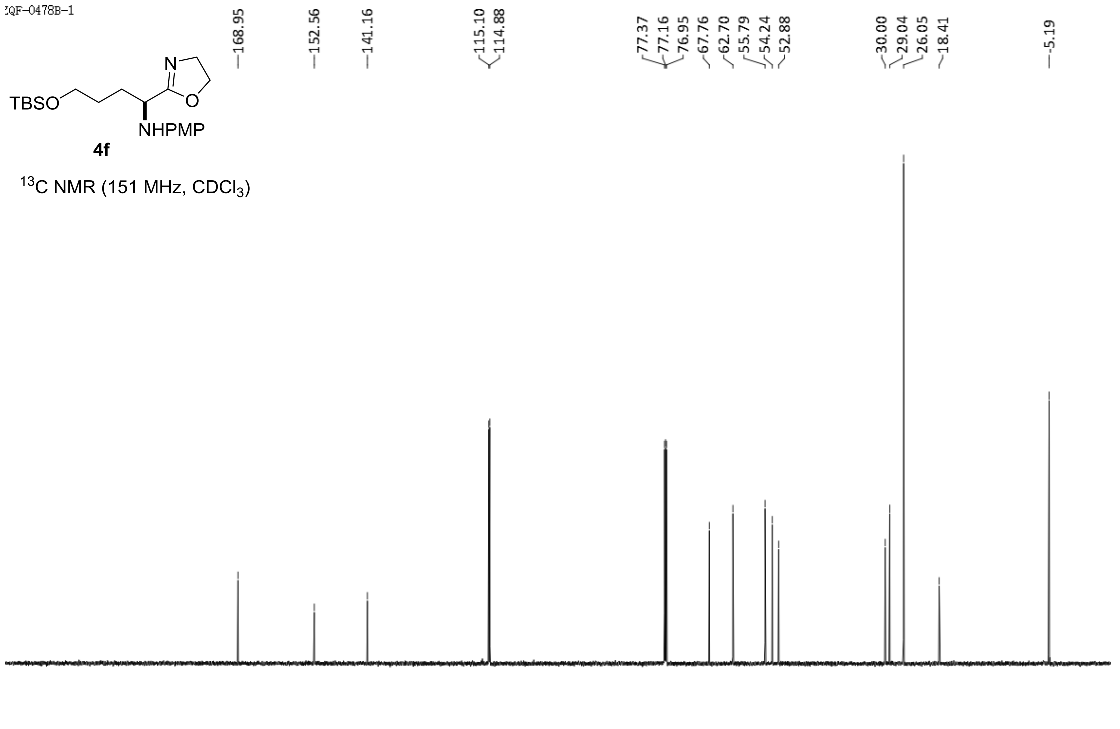
ZQF-0478B-1
168.95 152.56 141.16 115.10 114.88 77.37 77.16 76.95 67.76 62.70 55.79 54.24 52.88 30.00 29.04 26.05 18.41 -5.19
TBSO
N
O
NHPMP
4f
$^{13}$C NMR (151 MHz, $CDCl_3$)
200 180 160 140 120 100 80 60 40 20 0
f1 (ppm)

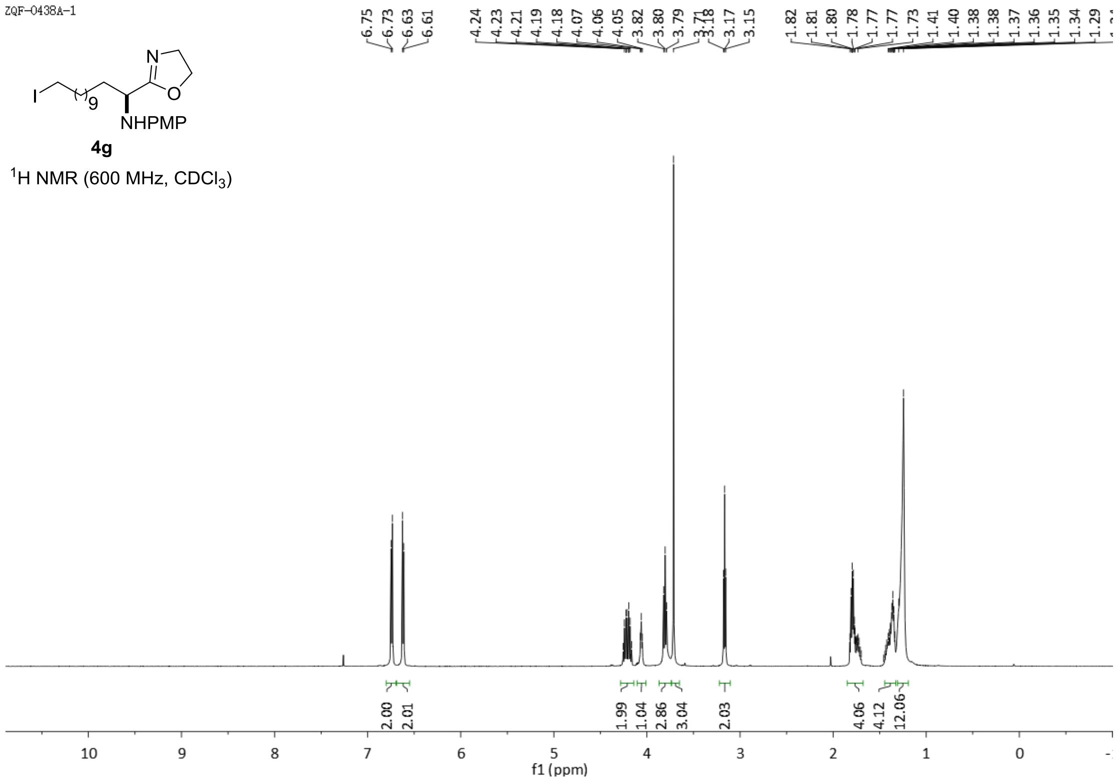
ZQF-0438A-1
4g
$^1$H NMR (600 MHz, $CDCl_3$)
f1 (ppm)

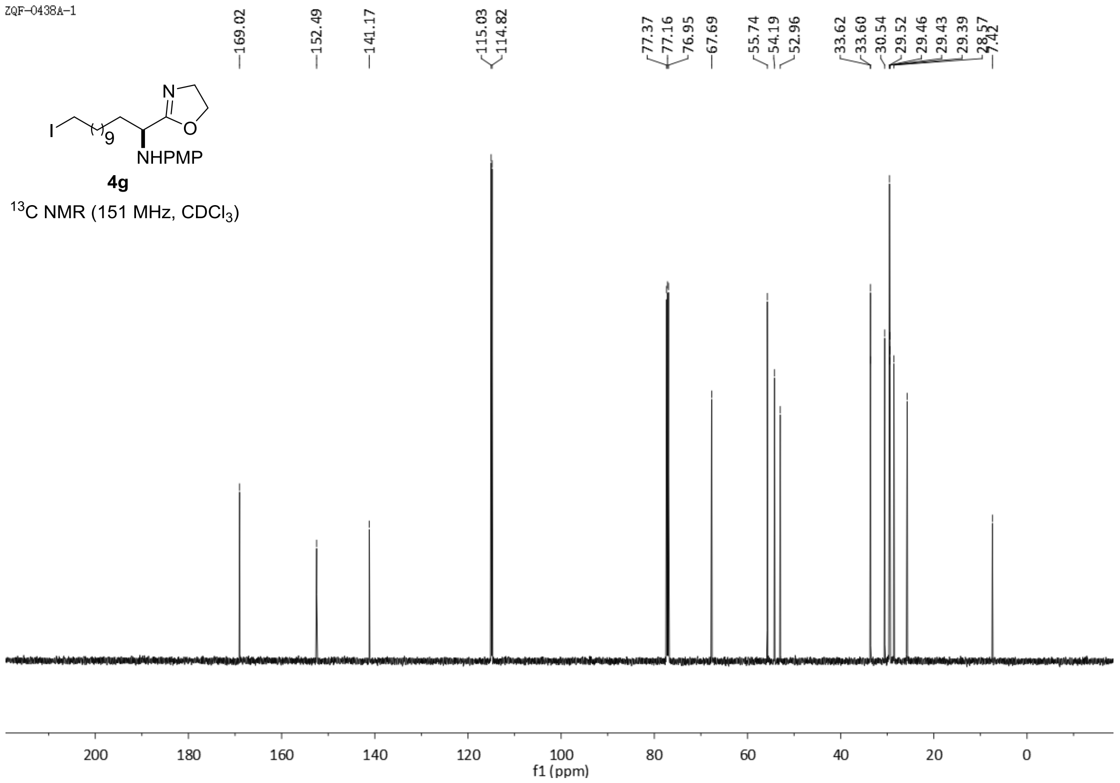
ZQF-0438A-1
4g
$^{13}$C NMR (151 MHz, $CDCl_3$)
f1 (ppm)

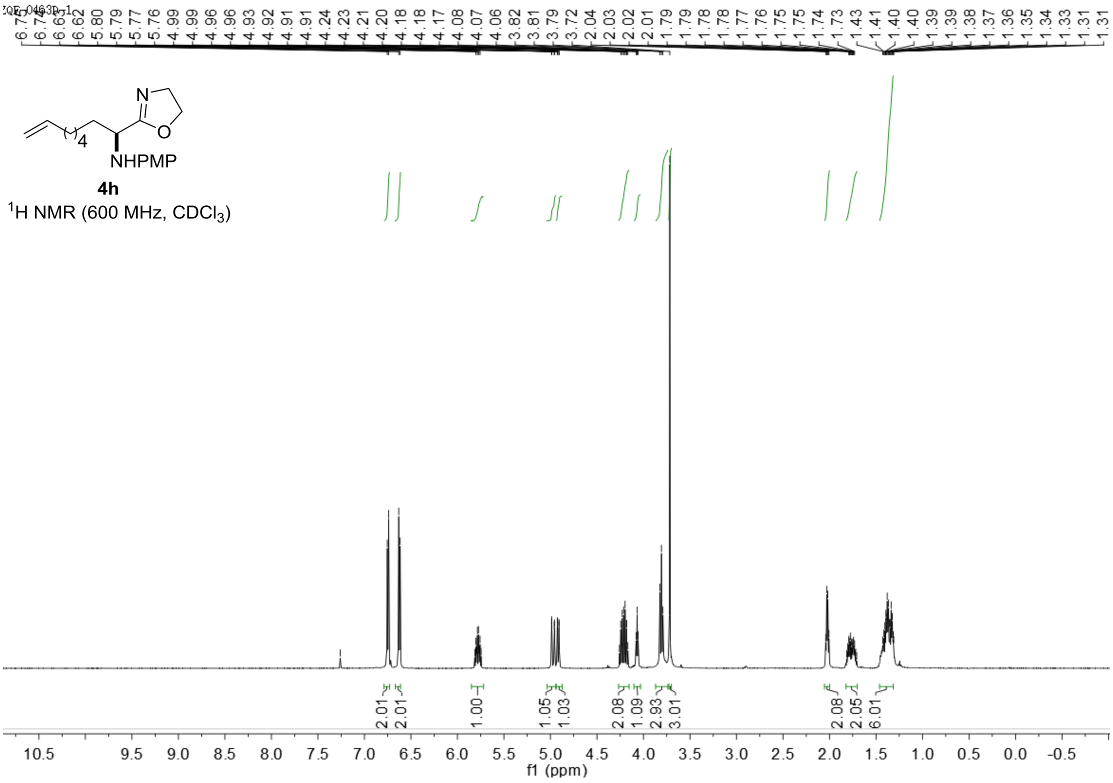


**4h**

$^{1}H$ NMR (600 MHz, $CDCl_3$)

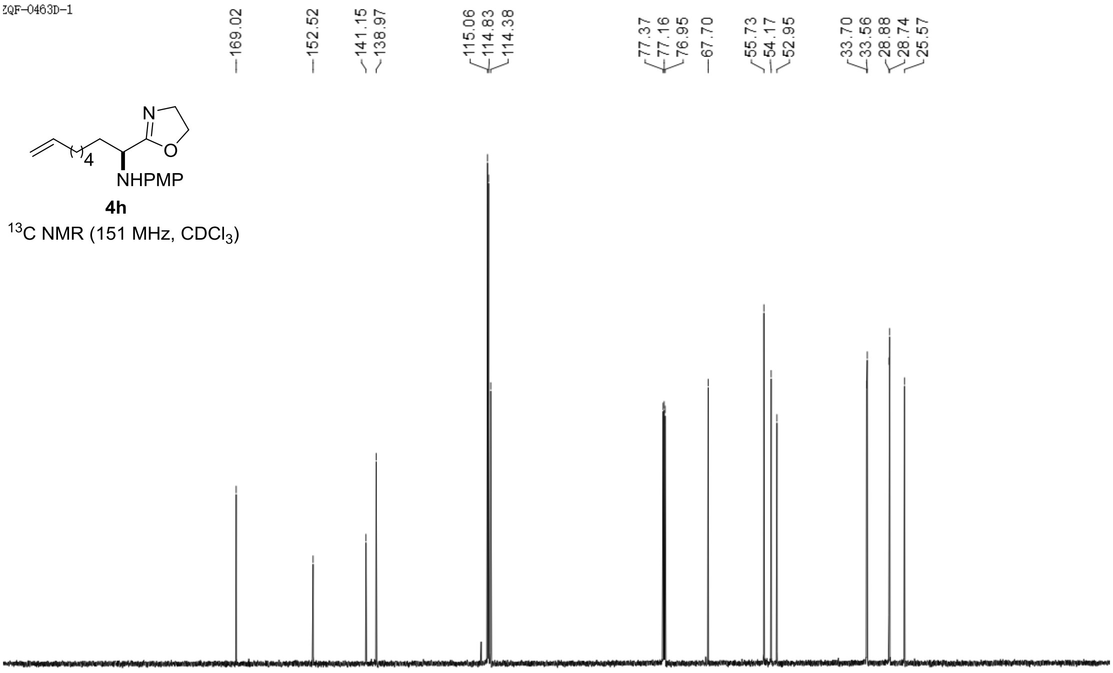


**4h**

$^{13}C$ NMR (151 MHz, $CDCl_3$)

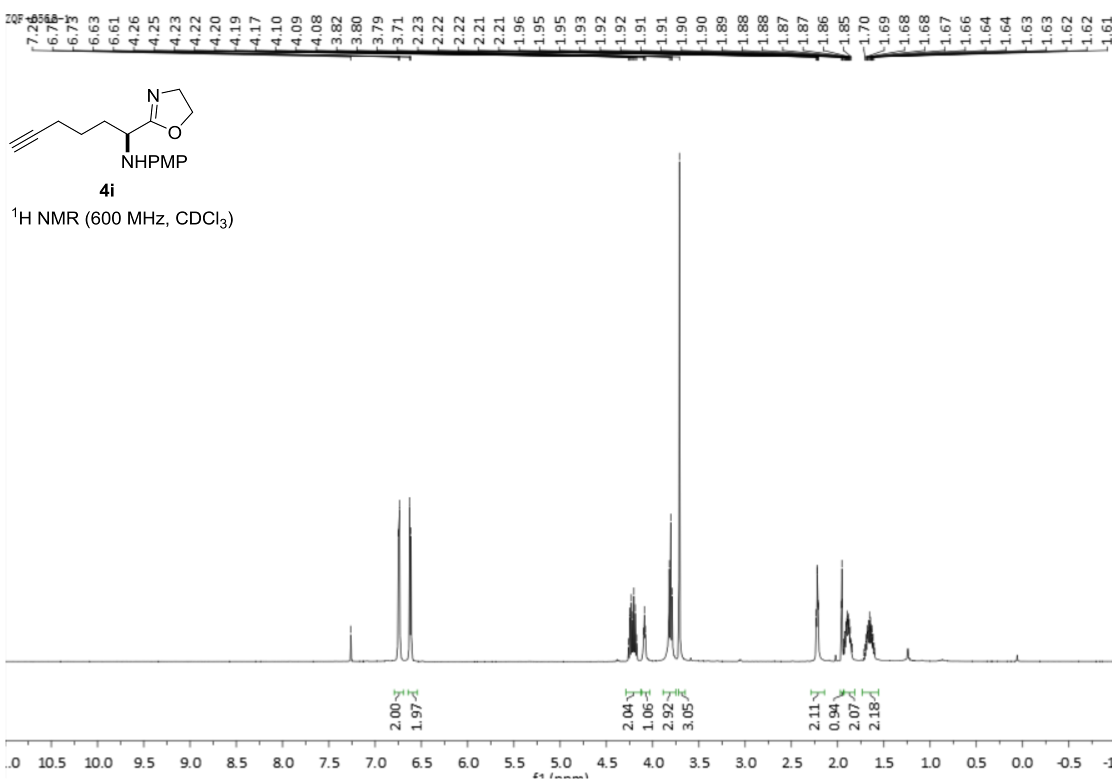

**4i**

$^{1}H$ NMR (600 MHz, $CDCl_3$)

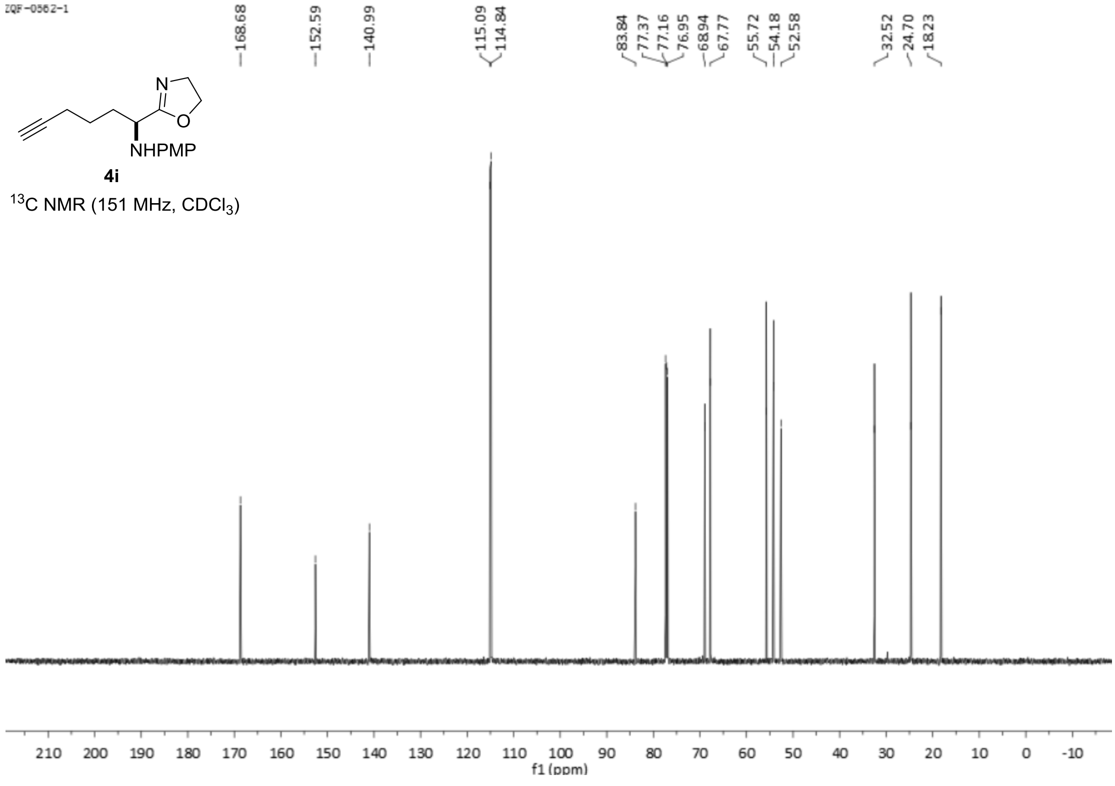

**4i**

$^{13}C$ NMR (151 MHz, $CDCl_3$)

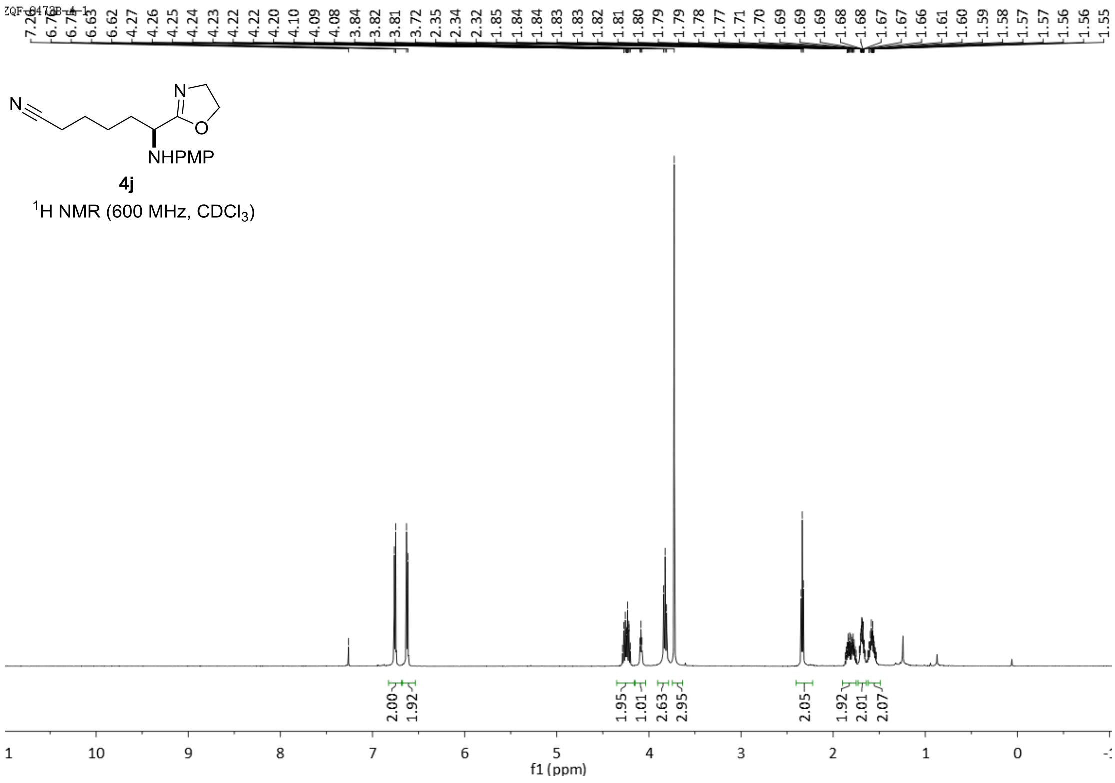
ZQF-0473B-4-1
7.26
6.75
6.74
6.63
6.62
4.27
4.26
4.25
4.24
4.23
4.22
4.22
4.20
4.10
4.09
4.08
3.84
3.82
3.81
3.72
2.35
2.34
2.32
1.85
1.84
1.84
1.83
1.83
1.82
1.81
1.80
1.79
1.79
1.78
1.77
1.71
1.70
1.69
1.69
1.69
1.68
1.68
1.67
1.67
1.66
1.61
1.60
1.59
1.58
1.57
1.56
1.56
1.55
N
N
O
NHPMP
4j
$^1$H NMR (600 MHz, CDCl$_3$)
2.00
1.92
1.95
1.01
2.63
2.95
2.05
1.92
2.01
2.07
f1 (ppm)

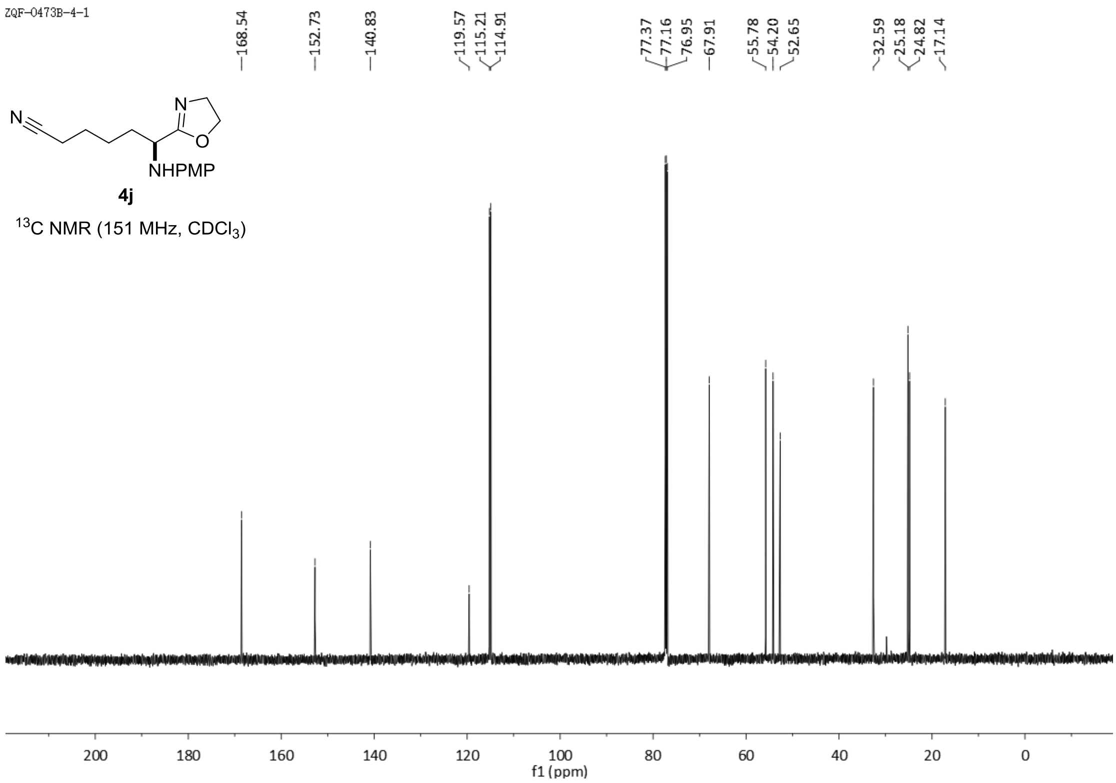
ZQF-0473B-4-1
168.54
152.73
140.83
119.57
115.21
114.91
77.37
77.16
76.95
67.91
55.78
54.20
52.65
32.59
25.18
24.82
17.14
N
N
O
NHPMP
4j
$^{13}$C NMR (151 MHz, CDCl$_3$)
f1 (ppm)

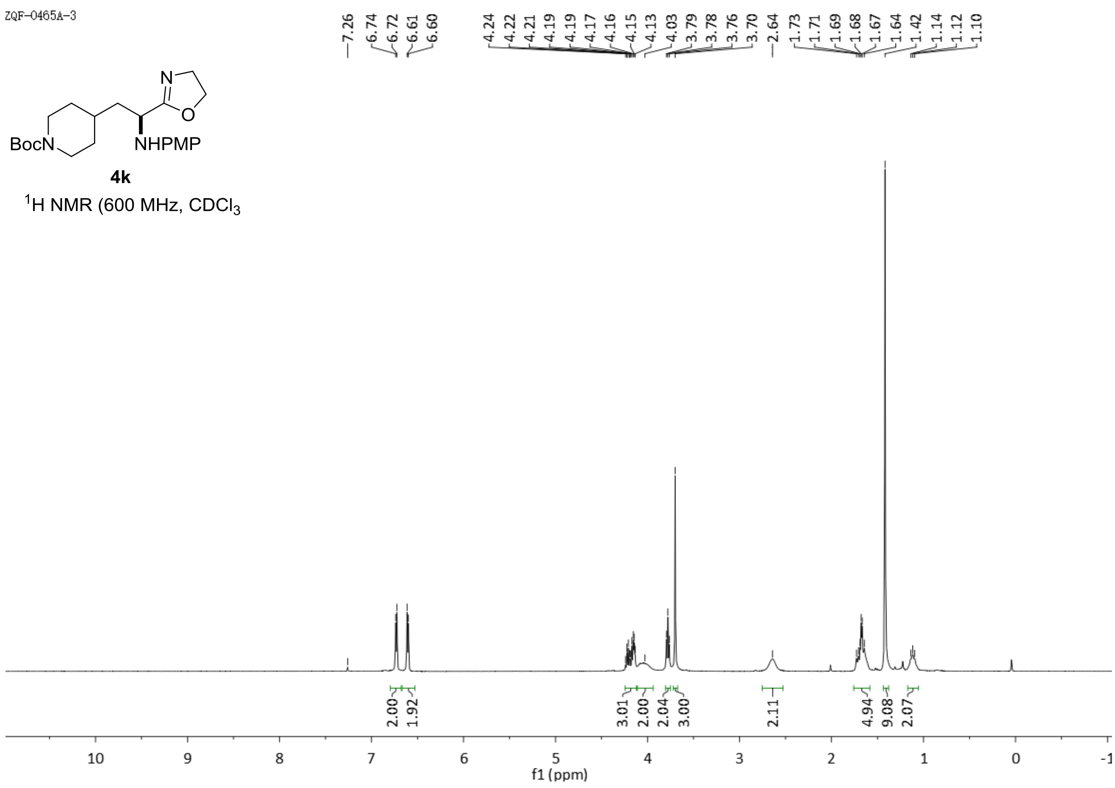
ZQF-0465A-3
7.26
6.74
6.72
6.61
6.60
4.24
4.22
4.21
4.19
4.19
4.17
4.16
4.15
4.13
4.03
3.79
3.78
3.76
3.70
2.64
1.73
1.71
1.69
1.68
1.67
1.64
1.42
1.14
1.12
1.10
N
O
BocN
NHPMP
4k
$^{1}H$ NMR (600 MHz, $CDCl_3$
2.00
1.92
3.01
2.00
2.04
3.00
2.11
4.94
9.08
2.07
f1 (ppm)

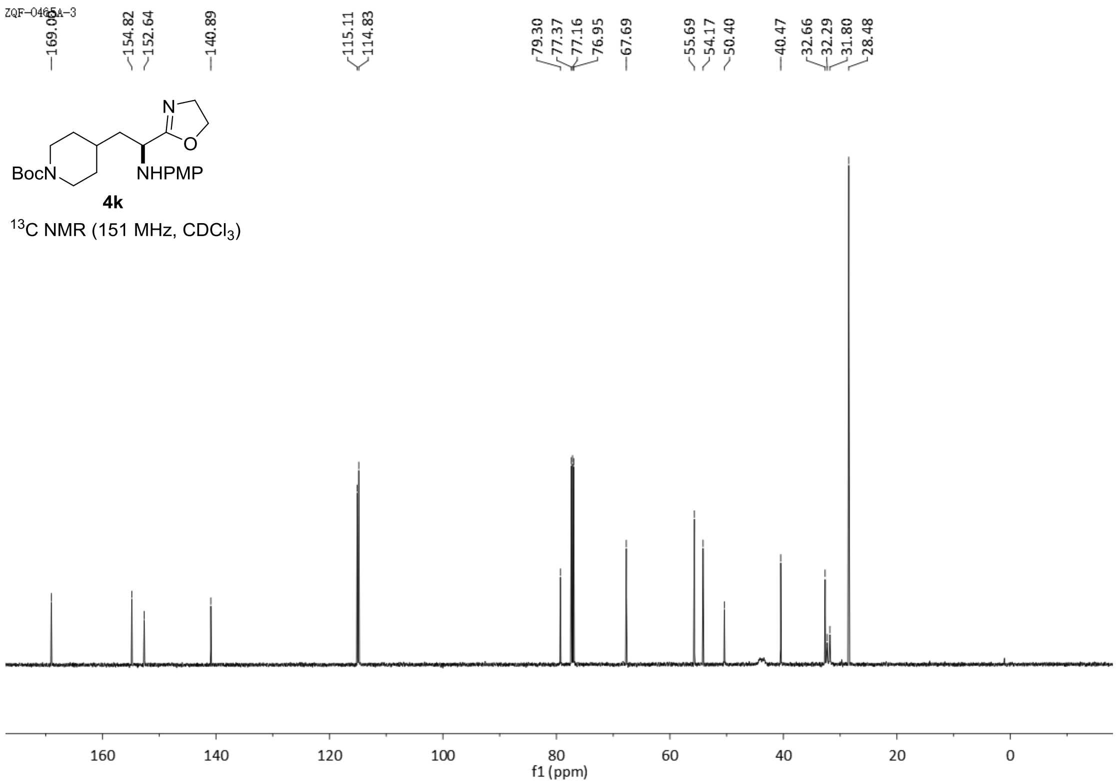
ZQF-0465A-3
169.06
154.82
152.64
140.89
115.11
114.83
79.30
77.37
77.16
76.95
67.69
55.69
54.17
50.40
40.47
32.66
32.29
31.80
28.48
N
O
BocN
NHPMP
4k
$^{13}C$ NMR (151 MHz, $CDCl_3$)
f1 (ppm)

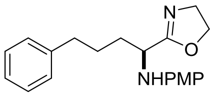

**4l**

$^{1}$H NMR (600 MHz, $CDCl_3$

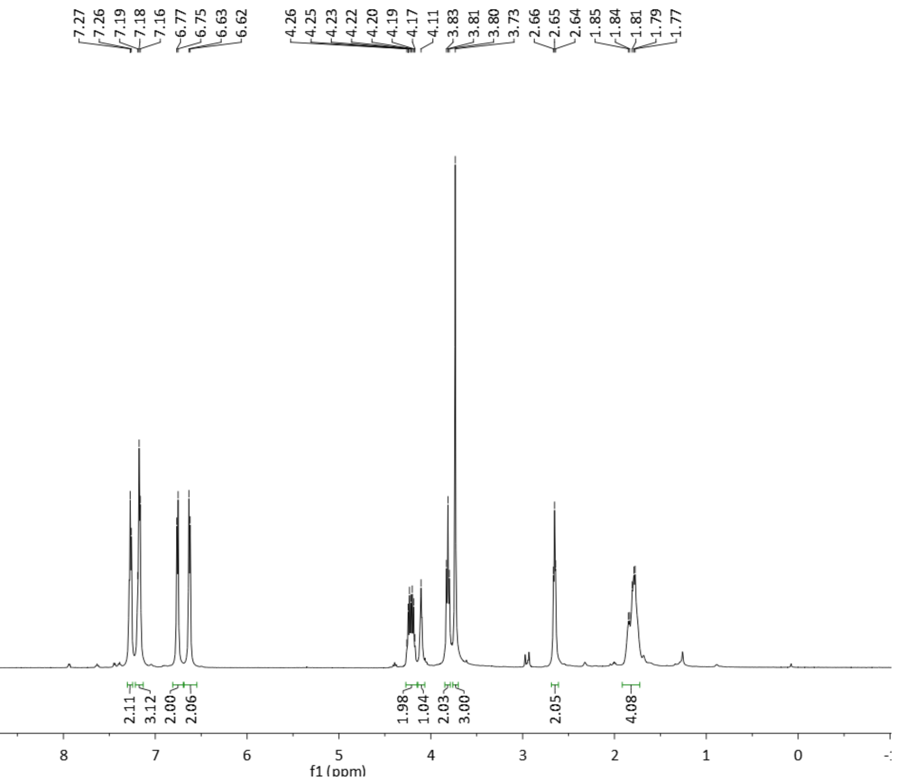


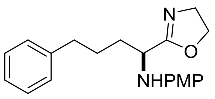

**4l**

$^{13}$C NMR (151 MHz, $CDCl_3$)

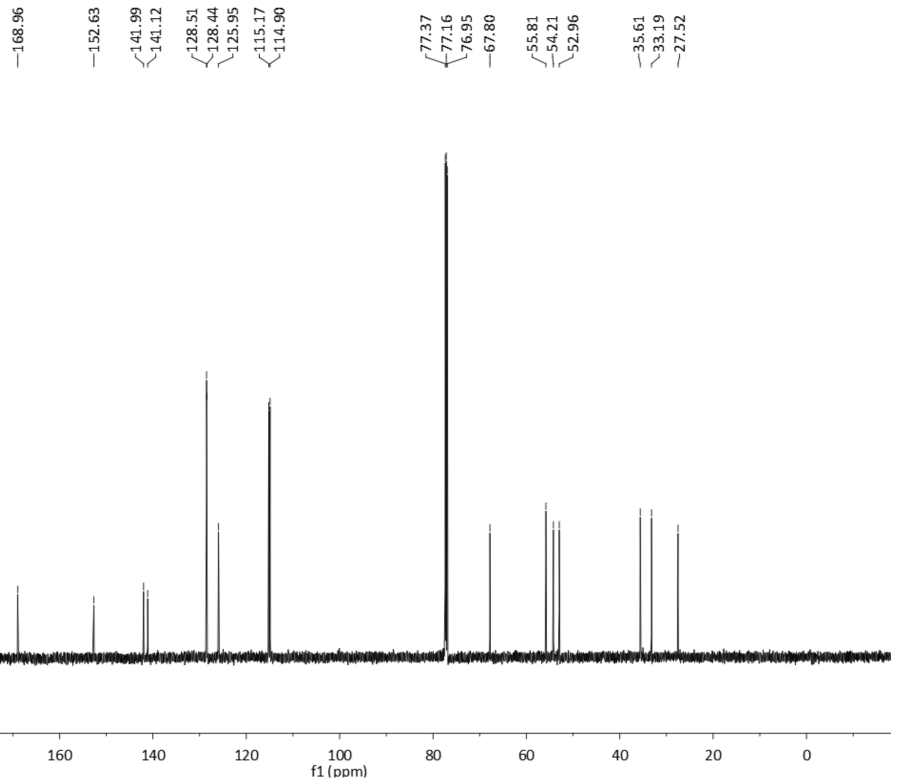

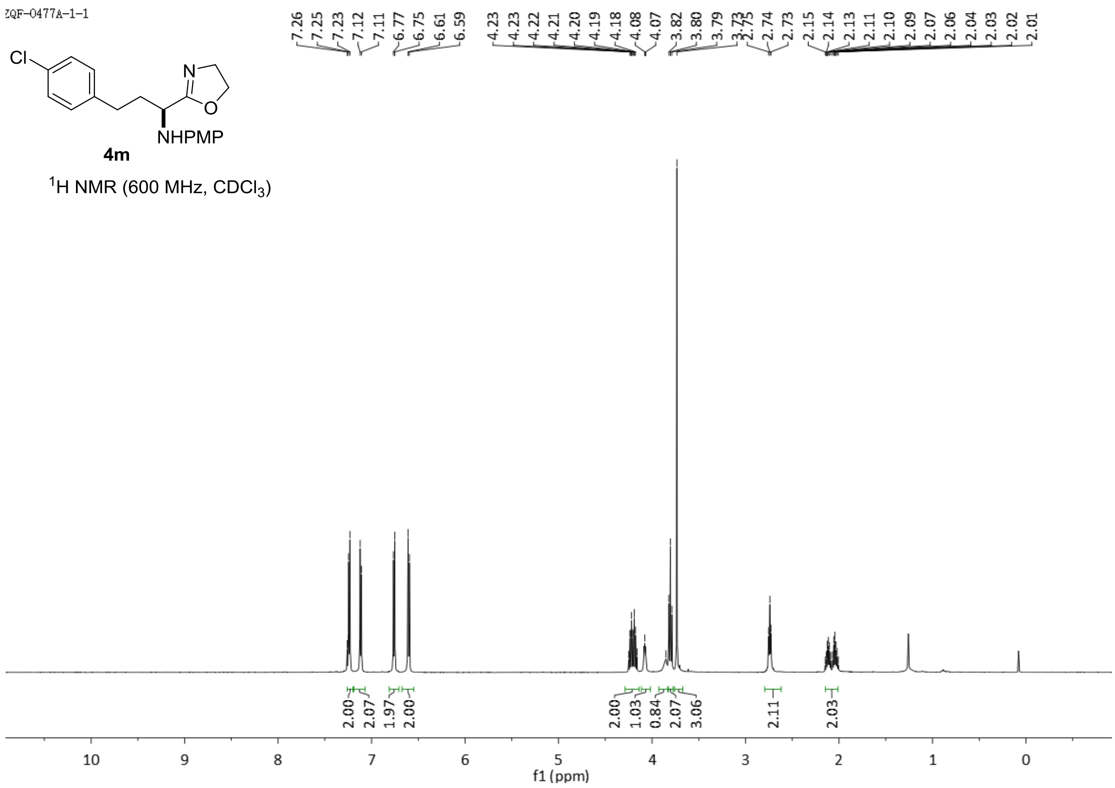
ZQF-0477A-1-1
7.26 7.25 7.23 7.12 7.11 6.77 6.75 6.61 6.59 4.23 4.23 4.22 4.21 4.20 4.19 4.18 4.08 4.07 3.82 3.80 3.79 3.73 3.75 2.74 2.73 2.15 2.14 2.13 2.11 2.10 2.09 2.07 2.06 2.04 2.03 2.02 2.01
Cl
N
O
NHPMP
4m
$^1$H NMR (600 MHz, CDCl$_3$)
2.00 2.07 1.97 2.00 2.00 1.03 0.84 2.07 3.06 2.11 2.03
10 9 8 7 6 5 4 3 2 1 0
f1 (ppm)

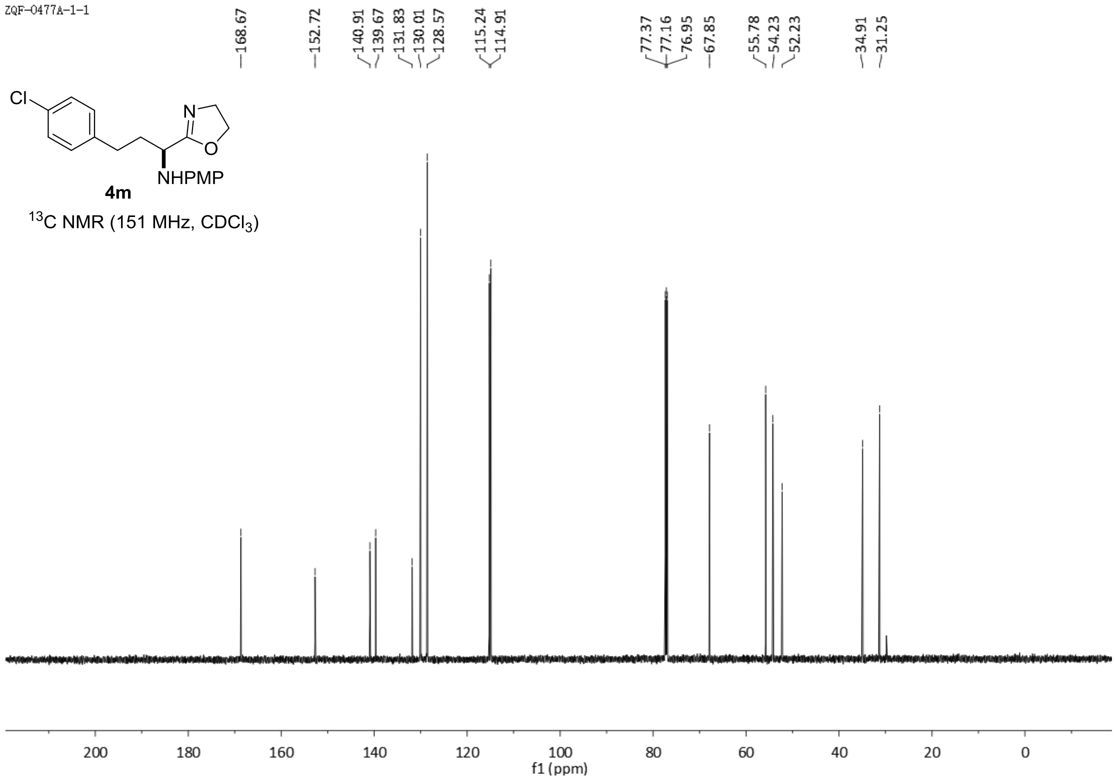
ZQF-0477A-1-1
168.67 152.72 140.91 139.67 131.83 130.01 128.57 115.24 114.91 77.37 77.16 76.95 67.85 55.78 54.23 52.23 34.91 31.25
Cl
N
O
NHPMP
4m
$^{13}$C NMR (151 MHz, CDCl$_3$)
200 180 160 140 120 100 80 60 40 20 0
f1 (ppm)

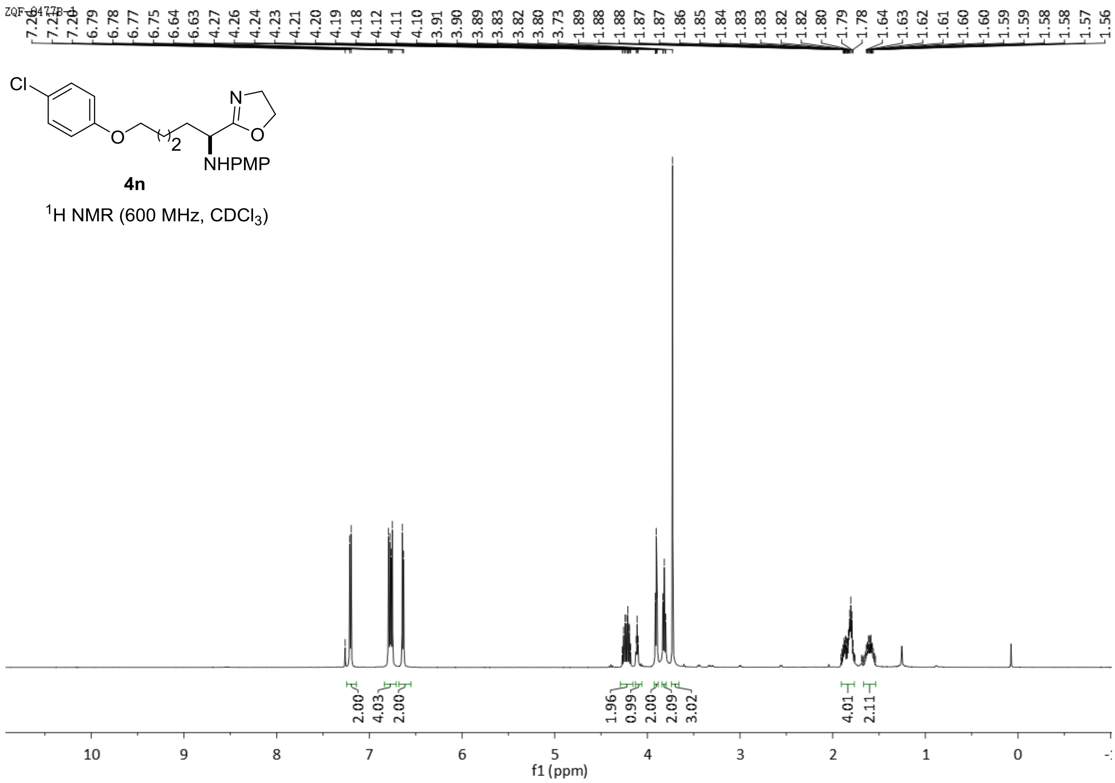
ZQF-0477B-1
7.26 7.21 7.20 6.79 6.78 6.77 6.75 6.64 6.63 4.27 4.26 4.24 4.23 4.21 4.20 4.19 4.18 4.12 4.11 4.10 3.91 3.90 3.89 3.83 3.82 3.80 3.73 1.89 1.88 1.88 1.87 1.87 1.86 1.85 1.84 1.83 1.83 1.82 1.82 1.80 1.79 1.78 1.64 1.63 1.62 1.61 1.60 1.60 1.59 1.59 1.58 1.58 1.57 1.56
Cl
O
N
O
2
NHPMP
4n
$^1$H NMR (600 MHz, CDCl$_3$)
2.00 4.03 2.00
1.96 0.99 2.00 2.09 3.02
4.01 2.11
10 9 8 7 6 5 4 3 2 1 0
f1 (ppm)

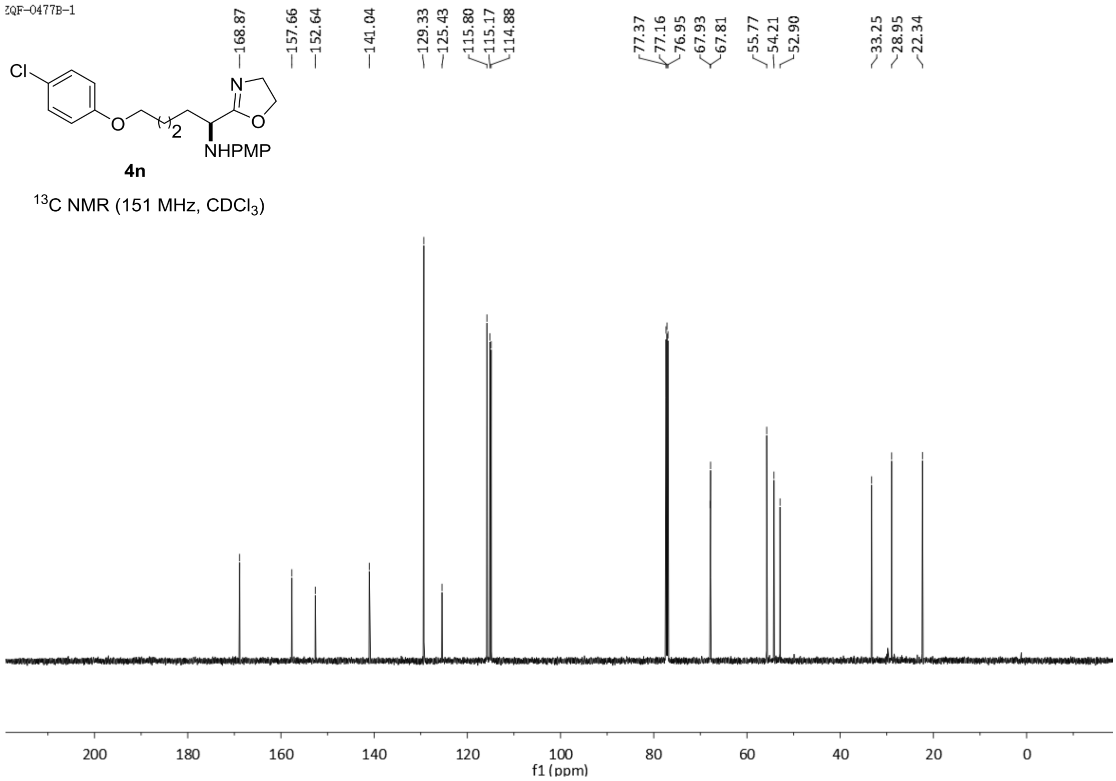
ZQF-0477B-1
168.87
157.66 152.64
141.04
129.33 125.43 115.80 115.17 114.88
77.37 77.16 76.95 67.93 67.81
55.77 54.21 52.90
33.25 28.95 22.34
Cl
O
N
O
2
NHPMP
4n
$^{13}$C NMR (151 MHz, CDCl$_3$)
200 180 160 140 120 100 80 60 40 20 0
f1 (ppm)

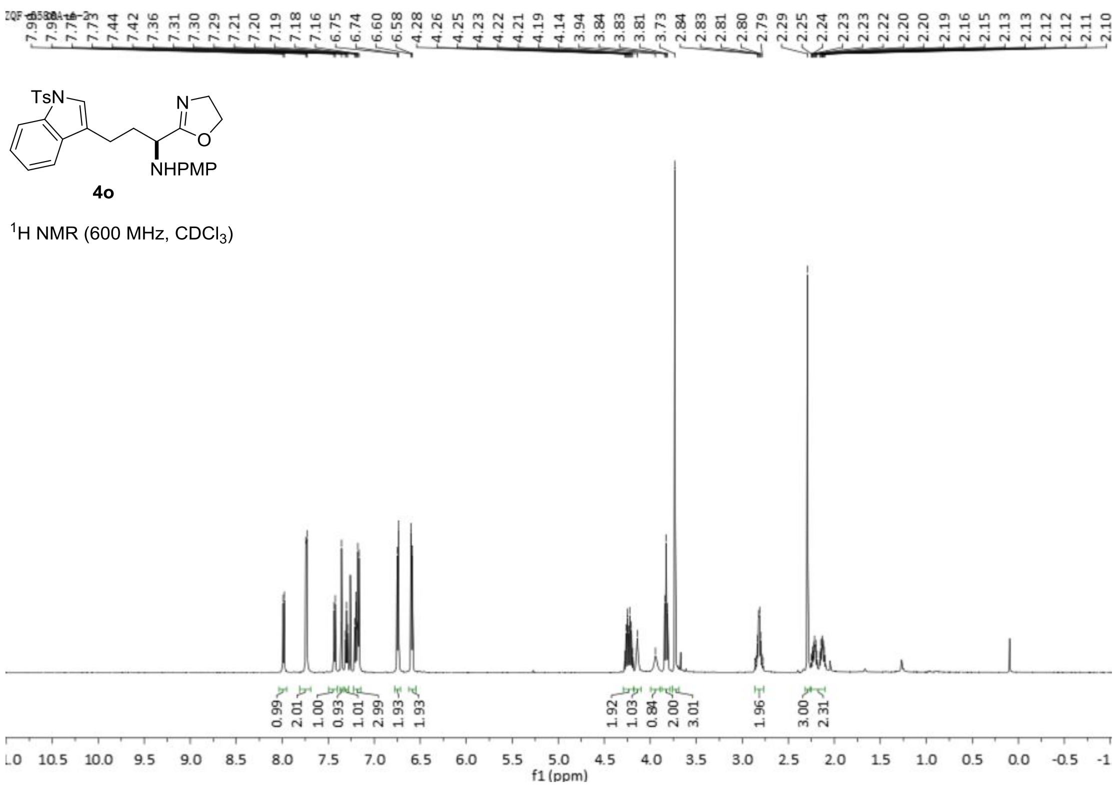

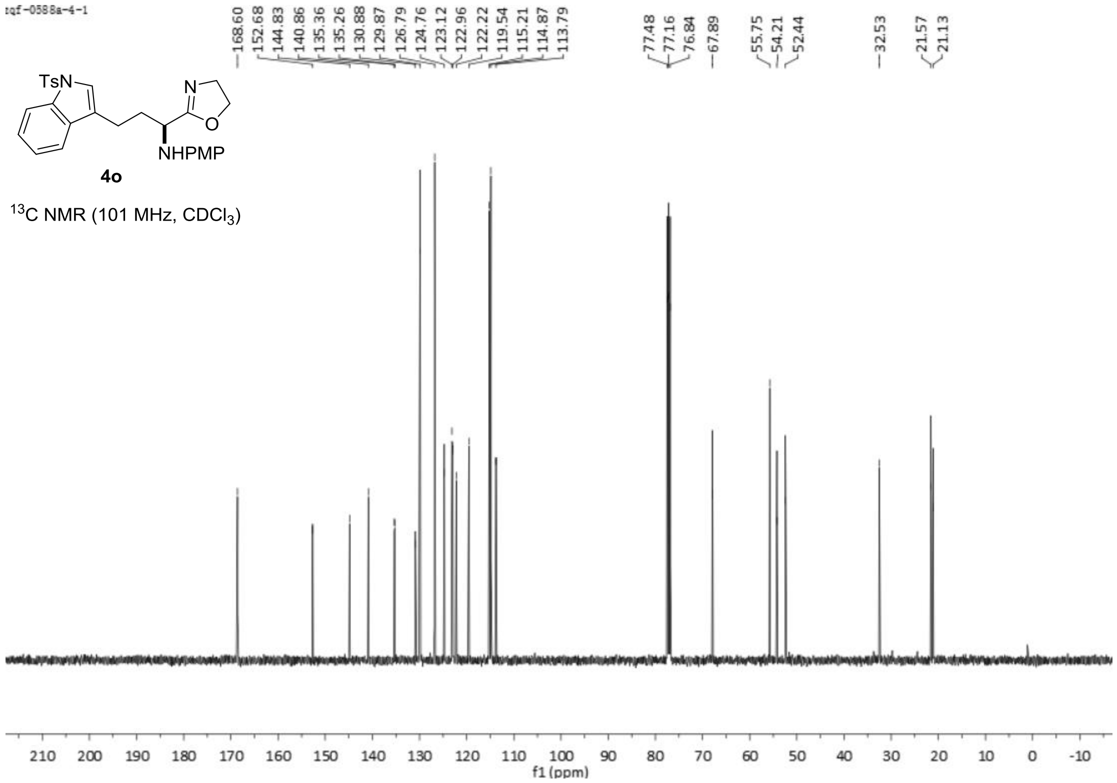

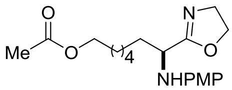

**4p**

$^{1}H$ NMR (600 MHz, $CDCl_3$)

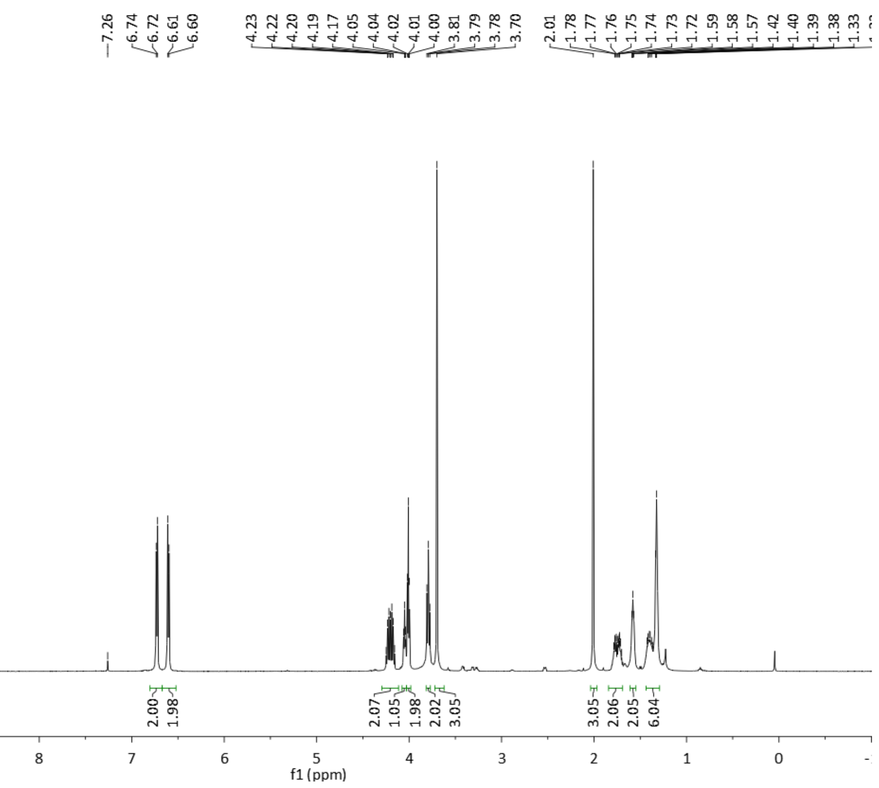


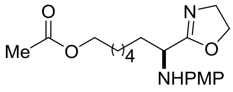

**4p**

$^{13}C$ NMR (151 MHz, $CDCl_3$)

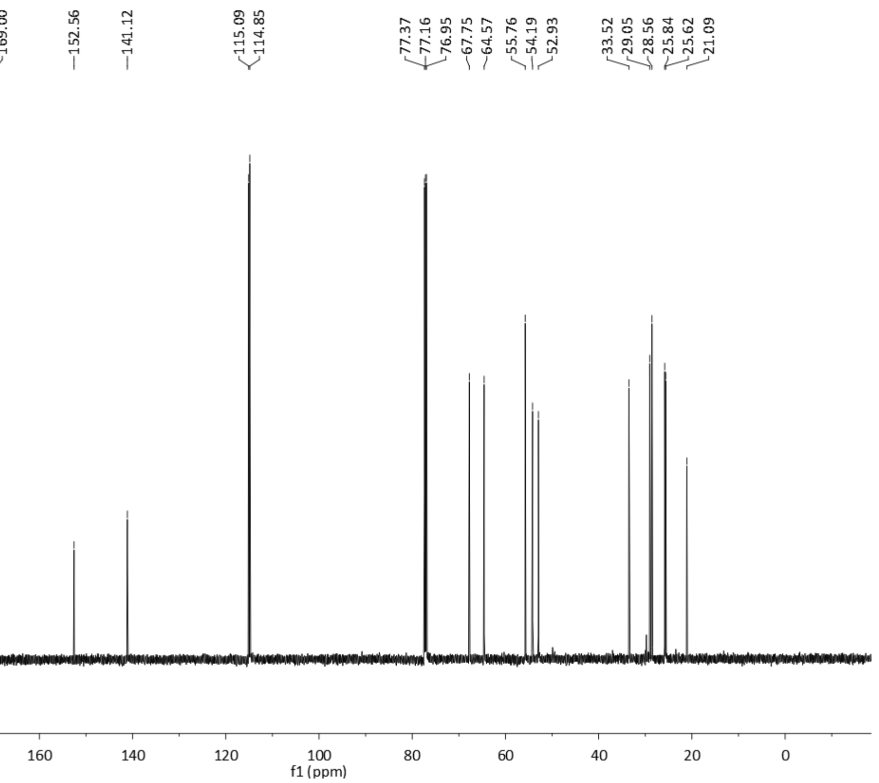

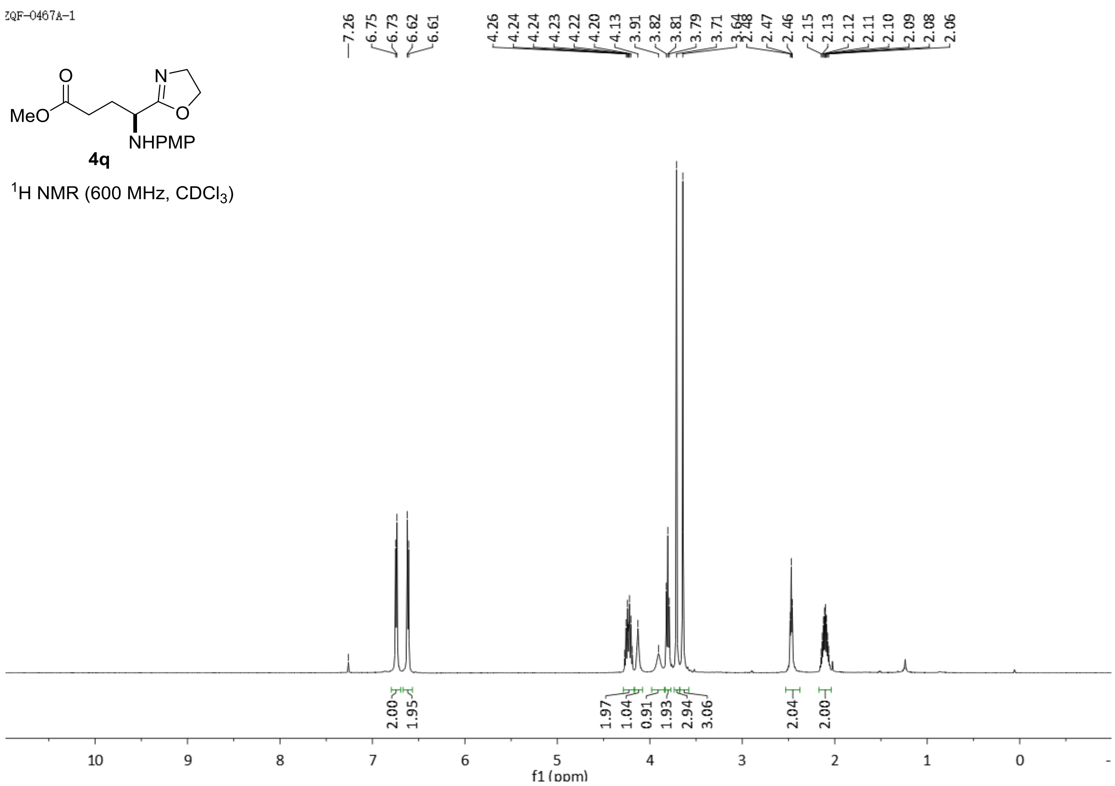
ZQF-0467A-1
7.26
6.75
6.73
6.62
6.61
4.26
4.24
4.24
4.23
4.22
4.20
4.13
3.91
3.82
3.81
3.79
3.71
3.64
2.48
2.47
2.46
2.15
2.13
2.12
2.11
2.10
2.09
2.08
2.06
MeO
O
N
O
NHPMP
4q
1H NMR (600 MHz, CDCl3)
2.00
1.95
1.97
1.04
0.91
1.93
2.94
3.06
2.04
2.00
10
9
8
7
6
5
4
3
2
1
0
f1 (ppm)

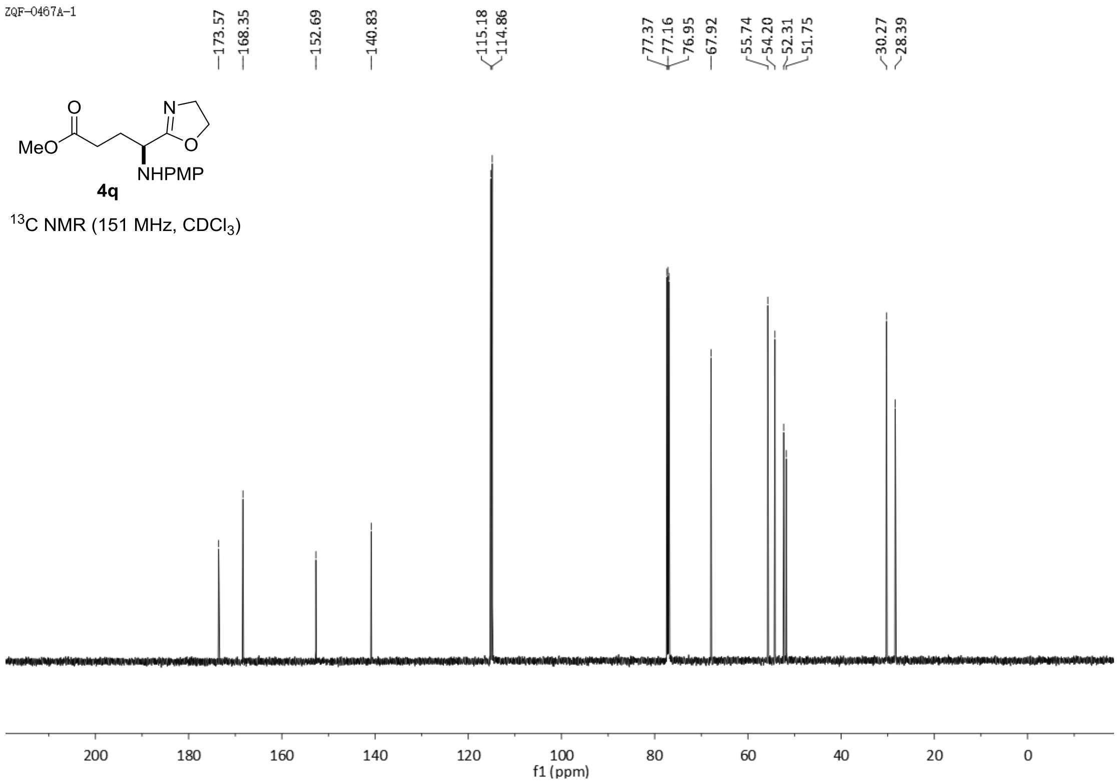
ZQF-0467A-1
173.57
168.35
152.69
140.83
115.18
114.86
77.37
77.16
76.95
67.92
55.74
54.20
52.31
51.75
30.27
28.39
MeO
O
N
O
NHPMP
4q
13C NMR (151 MHz, CDCl3)
200
180
160
140
120
100
80
60
40
20
0
f1 (ppm)

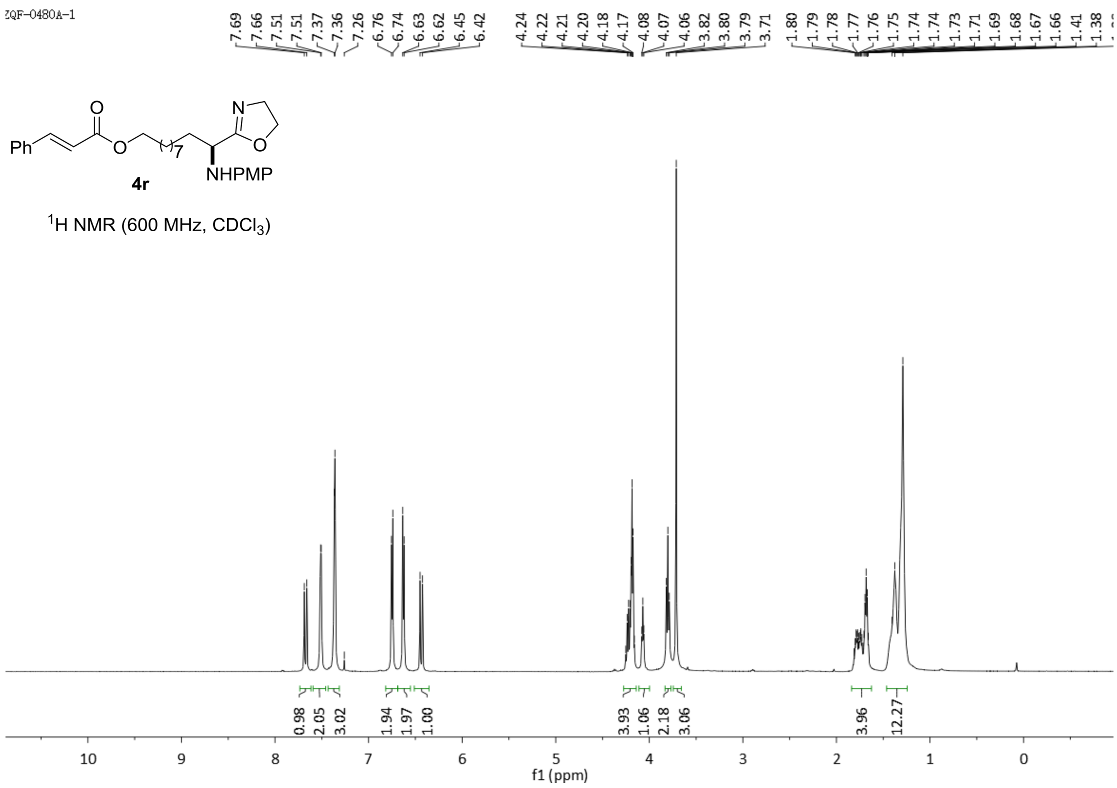
ZQF-0480A-1
7.69
7.66
7.51
7.51
7.37
7.36
7.26
6.76
6.74
6.63
6.62
6.45
6.42
4.24
4.22
4.21
4.20
4.18
4.17
4.08
4.07
4.06
3.82
3.80
3.79
3.71
1.80
1.79
1.78
1.77
1.76
1.75
1.74
1.74
1.73
1.71
1.69
1.68
1.67
1.66
1.41
1.38
Ph
O
O
7
N
O
NHPMP
4r
$^{1}$H NMR (600 MHz, CDCl$_3$)
0.98
2.05
3.02
1.94
1.97
1.00
3.93
1.06
2.18
3.06
3.96
12.27
f1 (ppm)

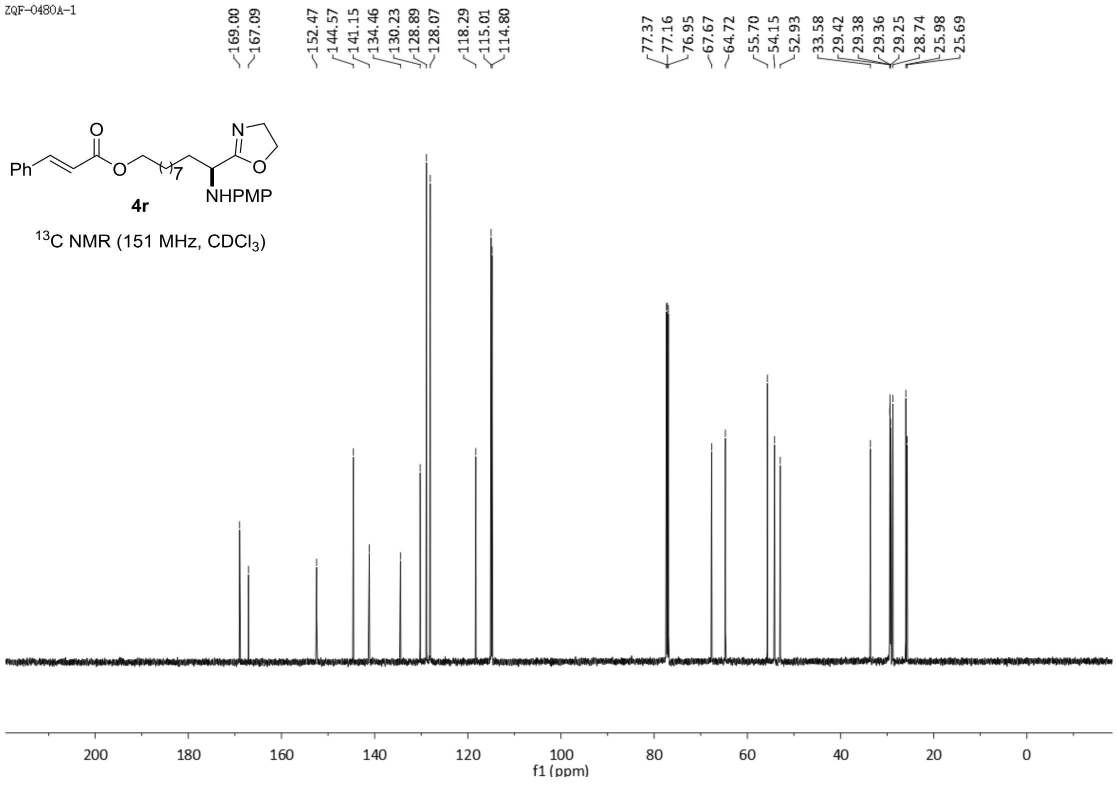
ZQF-0480A-1
169.00
167.09
152.47
144.57
141.15
134.46
130.23
128.89
128.07
118.29
115.01
114.80
77.37
77.16
76.95
67.67
64.72
55.70
54.15
52.93
33.58
29.42
29.38
29.36
29.25
28.74
25.98
25.69
Ph
O
O
7
N
O
NHPMP
4r
$^{13}$C NMR (151 MHz, CDCl$_3$)
f1 (ppm)

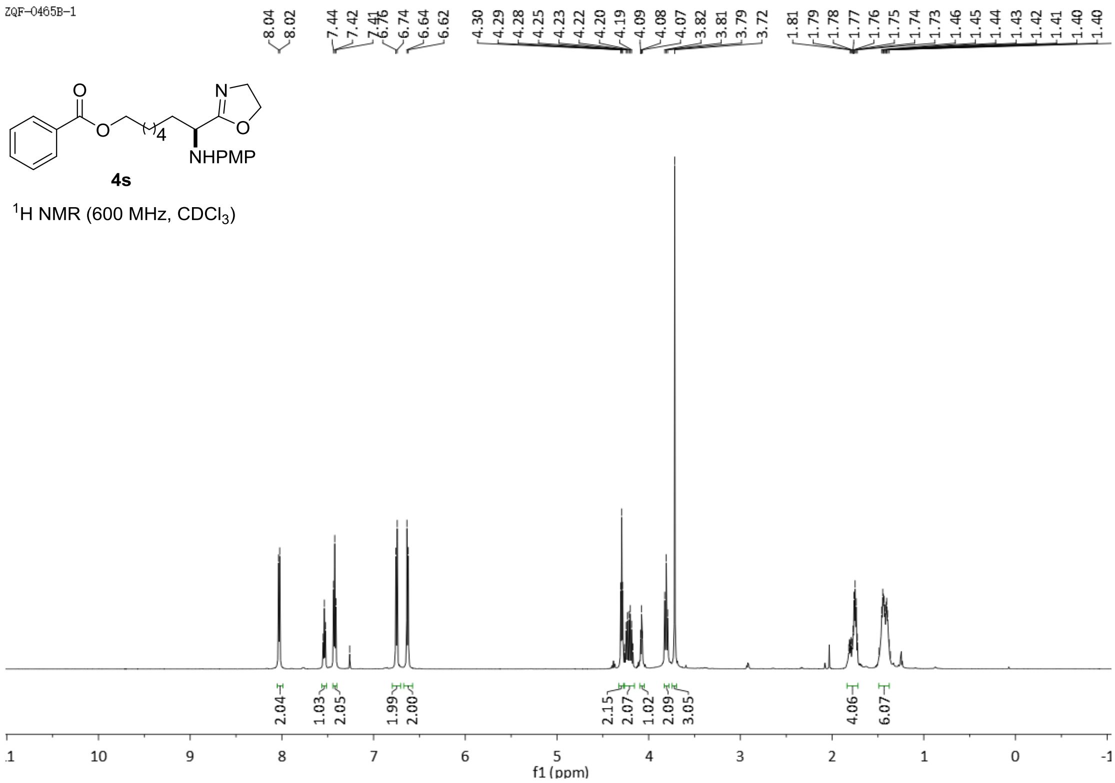
ZQF-0465B-1
8.04
8.02
7.44
7.42
7.41
6.76
6.74
6.64
6.62
4.30
4.29
4.28
4.25
4.23
4.22
4.20
4.19
4.09
4.08
4.07
3.82
3.81
3.79
3.72
1.81
1.79
1.78
1.77
1.76
1.75
1.74
1.73
1.46
1.45
1.44
1.43
1.42
1.41
1.40
1.40
O
N
O
O
4
NHPMP
4s
$^1$H NMR (600 MHz, CDCl$_3$)
2.04
1.03
2.05
1.99
2.00
2.15
2.07
1.02
2.09
3.05
4.06
6.07
f1 (ppm)

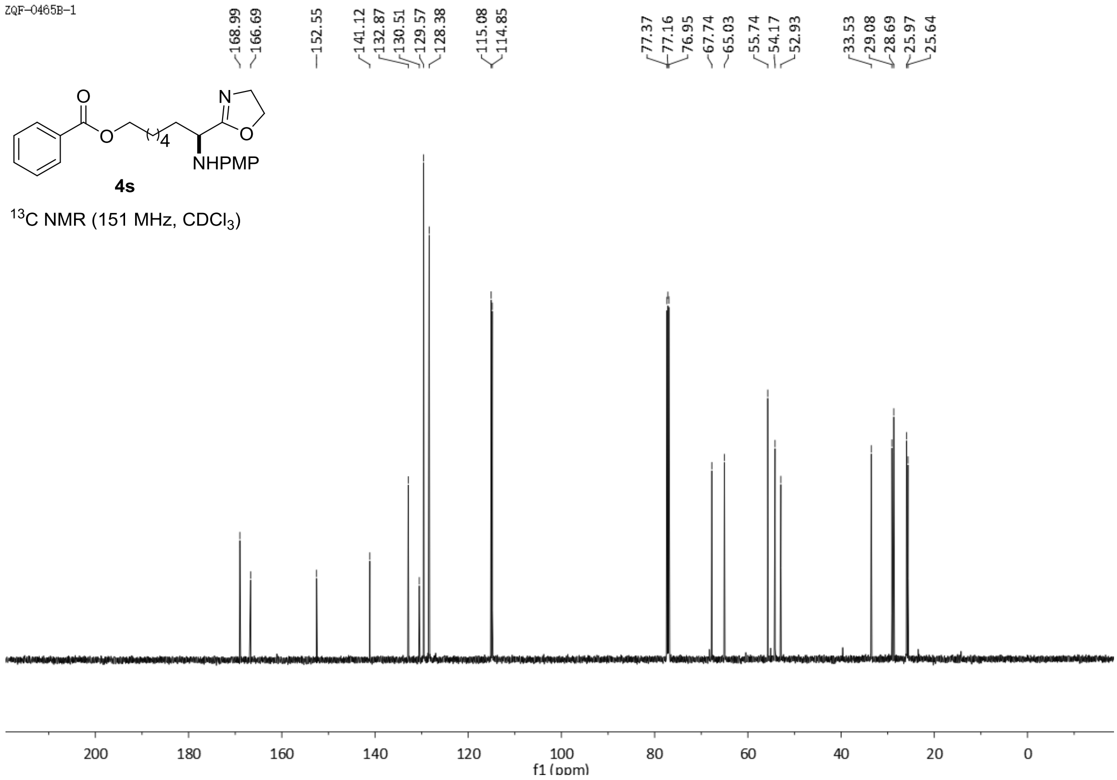
ZQF-0465B-1
168.99
166.69
152.55
141.12
132.87
130.51
129.57
128.38
115.08
114.85
77.37
77.16
76.95
67.74
65.03
55.74
54.17
52.93
33.53
29.08
28.69
25.97
25.64
O
N
O
O
4
NHPMP
4s
$^{13}$C NMR (151 MHz, CDCl$_3$)
f1 (ppm)

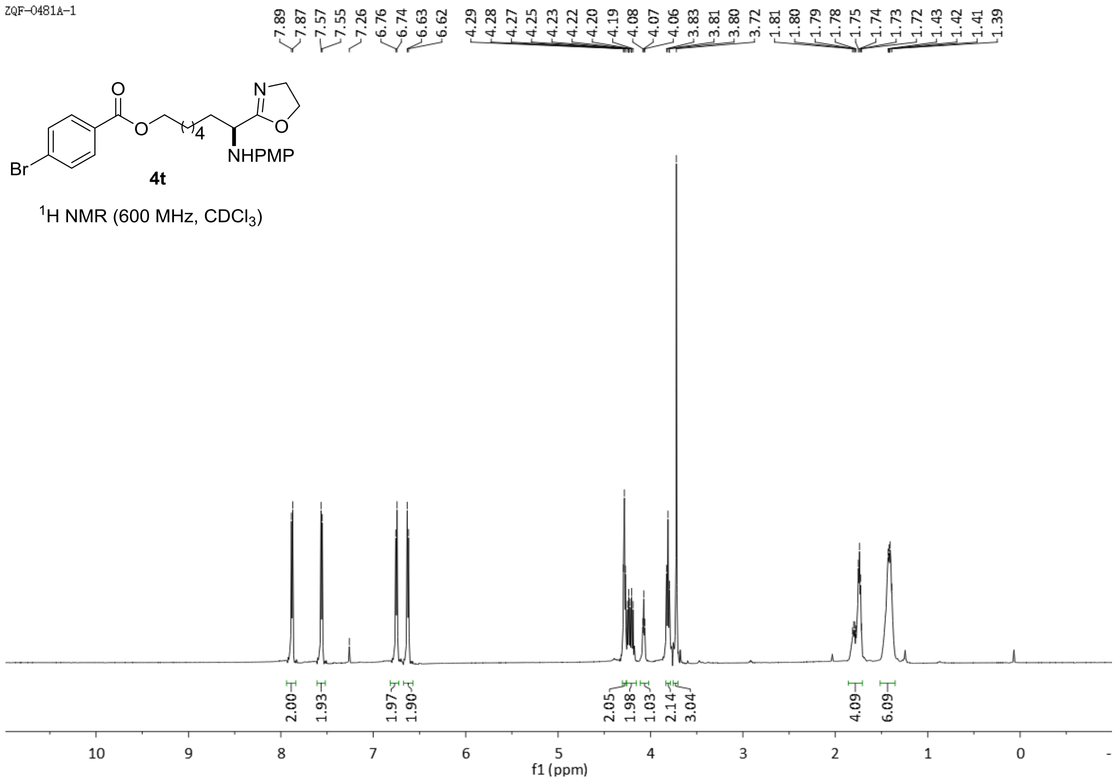
ZQF-0481A-1
7.89 7.87 7.57 7.55 7.26 6.76 6.74 6.63 6.62 4.29 4.28 4.27 4.25 4.23 4.22 4.20 4.19 4.08 4.07 4.06 3.83 3.81 3.80 3.72 1.81 1.80 1.79 1.78 1.75 1.74 1.73 1.72 1.43 1.42 1.41 1.39
O
N
O
O
4
Br
NHPMP
4t
$^{1}$H NMR (600 MHz, $CDCl_3$)
2.00 1.93 1.97 1.90 2.05 1.98 1.03 2.14 3.04 4.09 6.09
10 9 8 7 6 5 4 3 2 1 0
f1 (ppm)

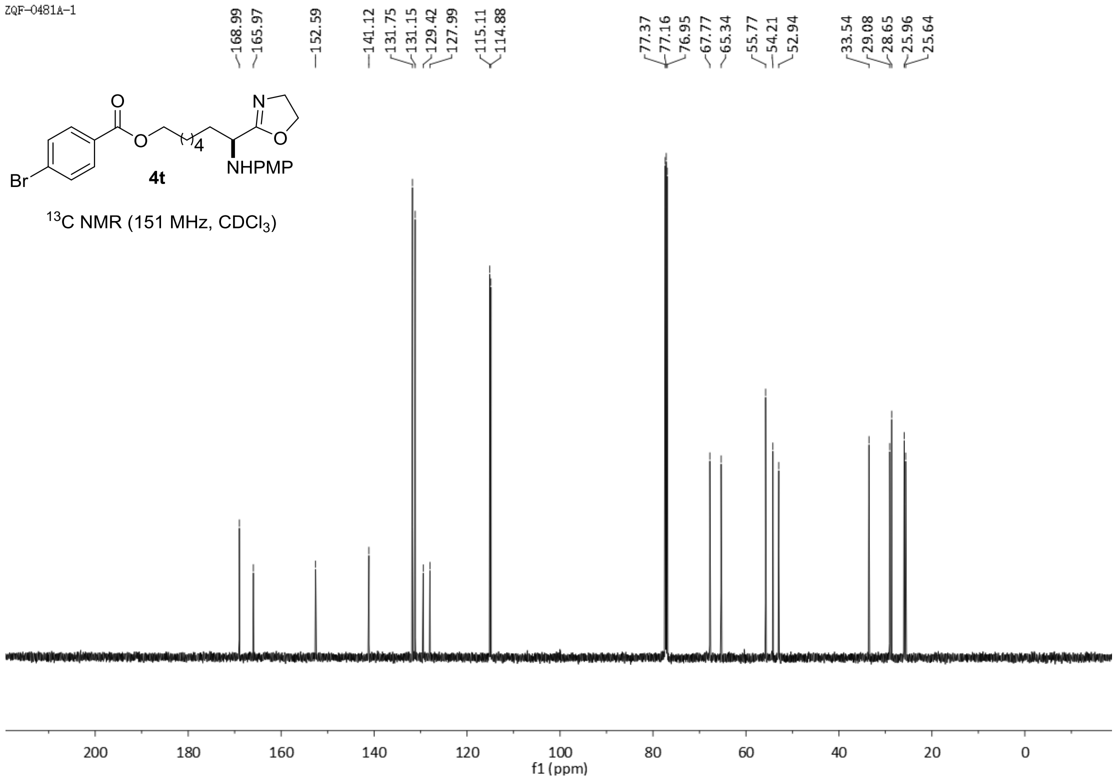
ZQF-0481A-1
168.99 165.97 152.59 141.12 131.75 131.15 129.42 127.99 115.11 114.88 77.37 77.16 76.95 67.77 65.34 55.77 54.21 52.94 33.54 29.08 28.65 25.96 25.64
O
N
O
O
4
Br
NHPMP
4t
$^{13}$C NMR (151 MHz, $CDCl_3$)
200 180 160 140 120 100 80 60 40 20 0
f1 (ppm)

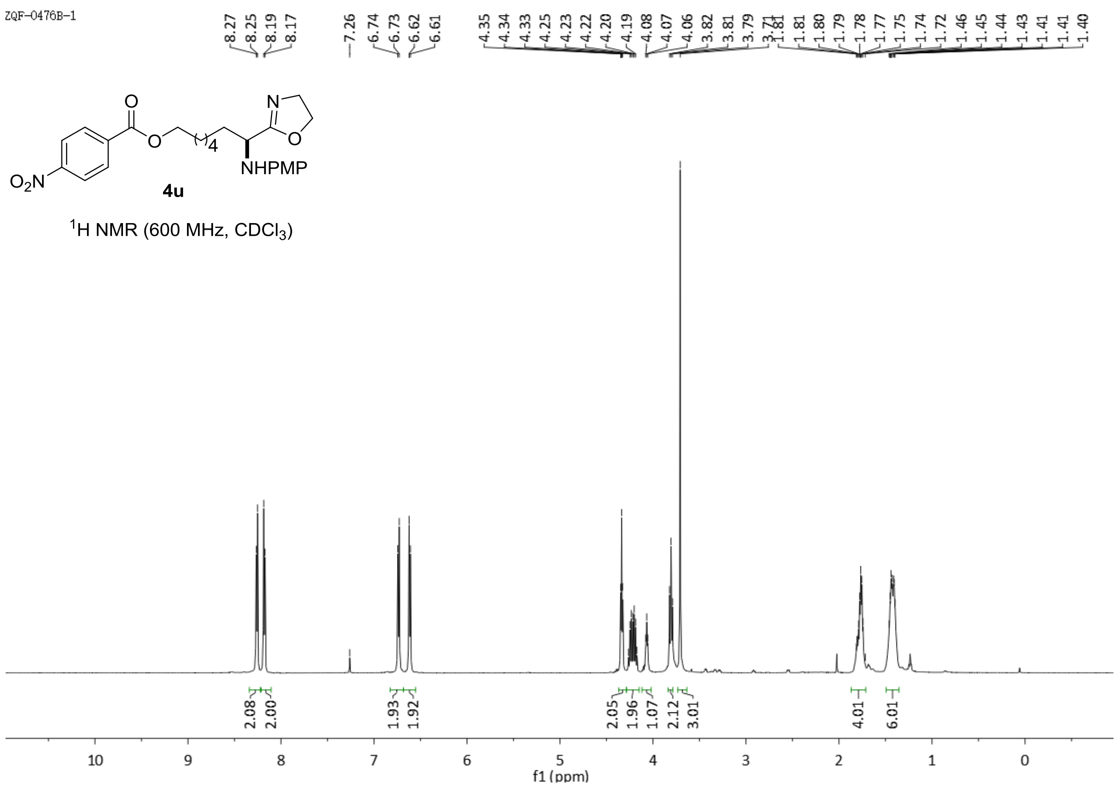
ZQF-0476B-1
8.27 8.25 8.19 8.17 7.26 6.74 6.73 6.62 6.61 4.35 4.34 4.33 4.25 4.23 4.22 4.20 4.19 4.08 4.07 4.06 3.82 3.81 3.79 3.71 1.81 1.80 1.79 1.78 1.77 1.75 1.74 1.72 1.46 1.45 1.44 1.43 1.41 1.40
O
N
O
O
4
O2N
NHPMP
4u
1H NMR (600 MHz, CDCl3)
2.08 2.00 1.93 1.92 2.05 1.96 1.07 2.12 3.01 4.01 6.01
10 9 8 7 6 5 4 3 2 1 0
f1 (ppm)

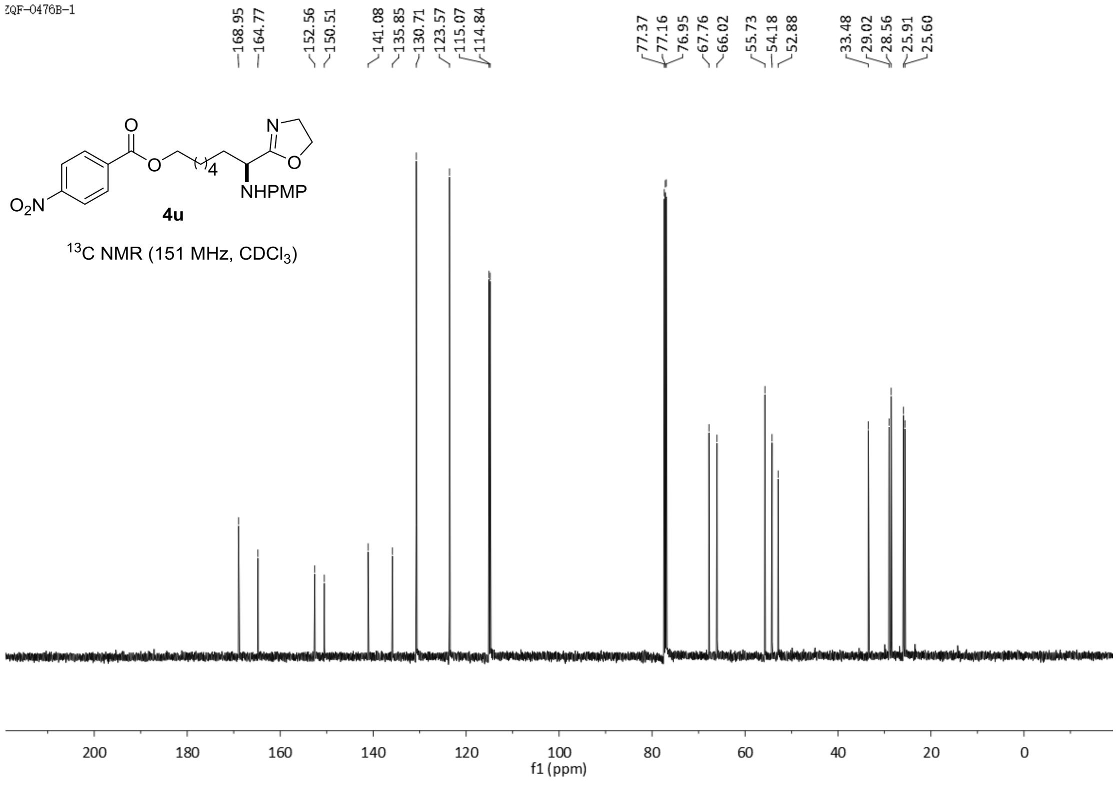
ZQF-0476B-1
168.95 164.77 152.56 150.51 141.08 135.85 130.71 123.57 115.07 114.84 77.37 77.16 76.95 67.76 66.02 55.73 54.18 52.88 33.48 29.02 28.56 25.91 25.60
O
N
O
O
4
O2N
NHPMP
4u
13C NMR (151 MHz, CDCl3)
200 180 160 140 120 100 80 60 40 20 0
f1 (ppm)

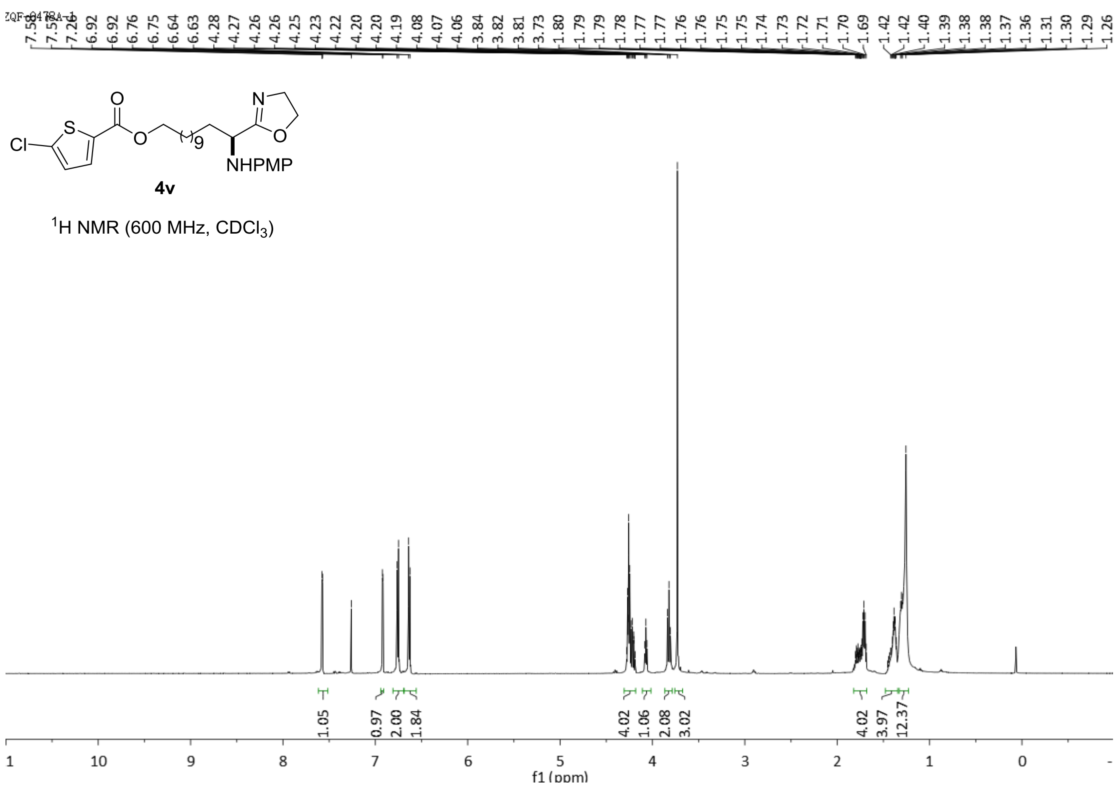


**4v**

$^1$H NMR (600 MHz, $CDCl_3$)

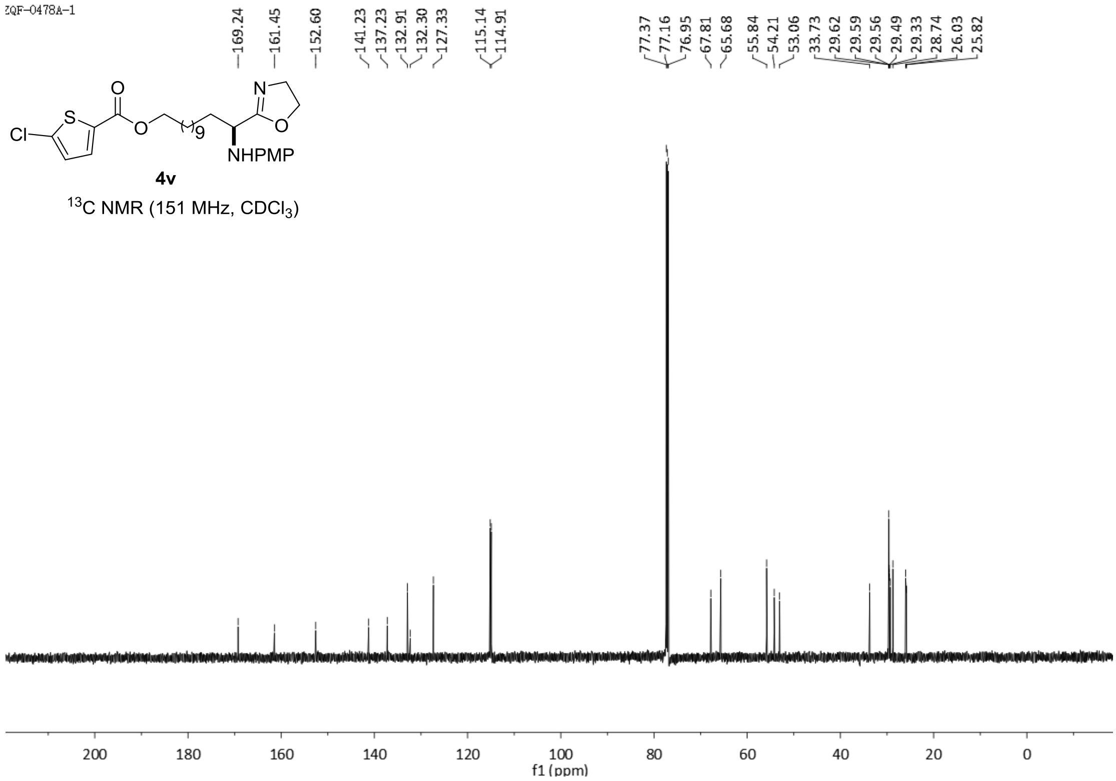


**4v**

$^{13}$C NMR (151 MHz, $CDCl_3$)

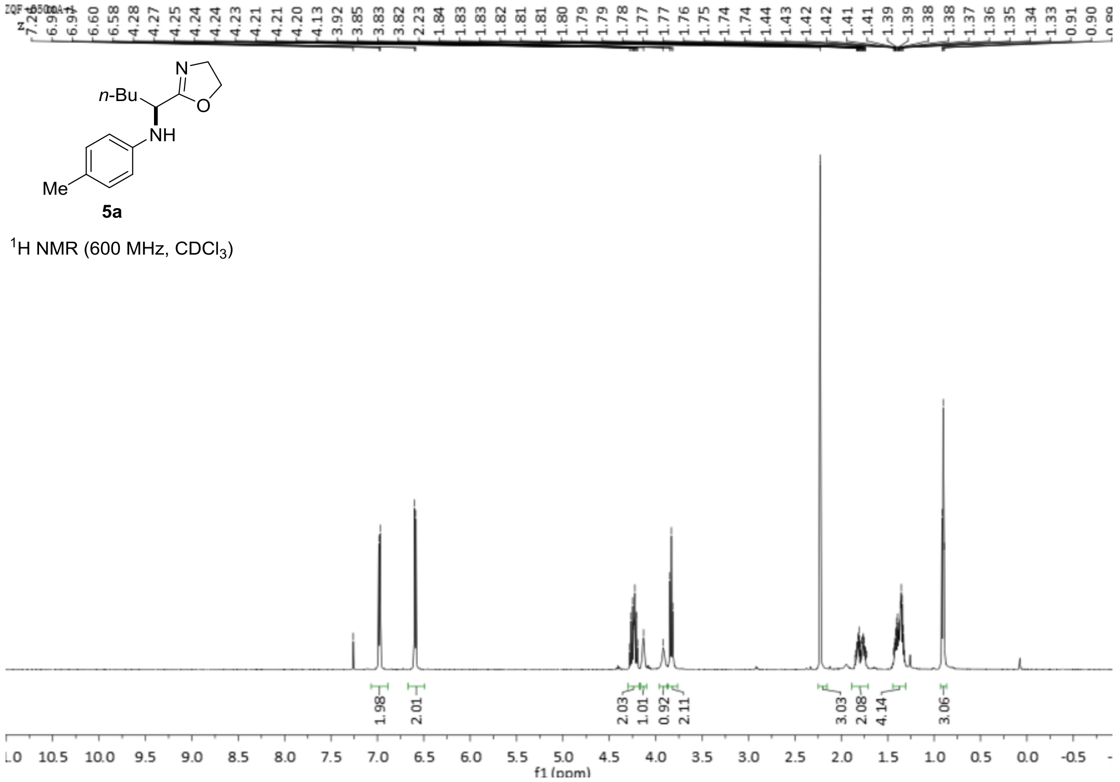
6.60 6.58 4.28 4.27 4.25 4.24 4.24 4.23 4.21 4.21 4.20 4.13 3.92 3.85 3.83 3.82 2.23 1.84 1.83 1.83 1.82 1.81 1.81 1.80 1.79 1.79 1.78 1.77 1.77 1.76 1.75 1.74 1.74 1.44 1.43 1.42 1.42 1.41 1.41 1.39 1.39 1.38 1.38 1.37 1.36 1.35 1.34 1.33 0.91 0.90
n-Bu
N
O
NH
Me
5a
$^1$H NMR (600 MHz, CDCl$_3$)
1.98 2.01 2.03 1.01 0.92 2.11 3.03 2.08 4.14 3.06
f1 (ppm)

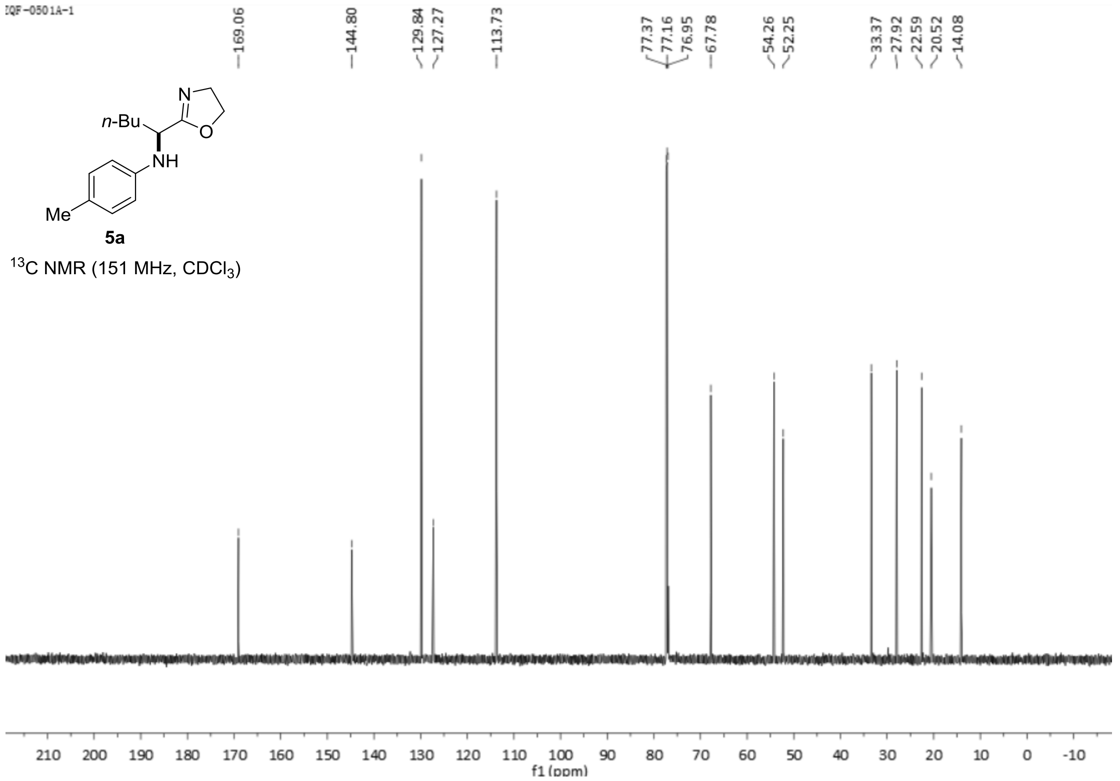
IQF-0501A-1
169.06 144.80 129.84 127.27 113.73 77.37 77.16 76.95 67.78 54.26 52.25 33.37 27.92 22.59 20.52 14.08
n-Bu
N
O
NH
Me
5a
$^{13}$C NMR (151 MHz, CDCl$_3$)
f1 (ppm)

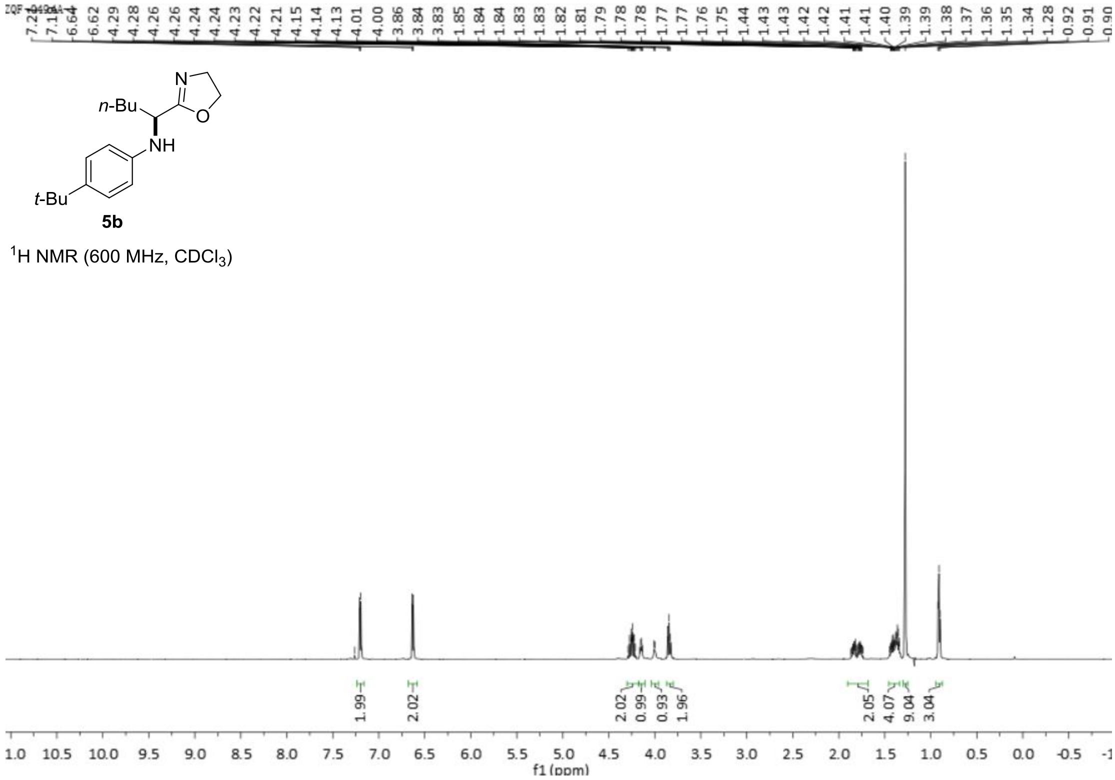


**5b**

$^1$H NMR (600 MHz, $CDCl_3$)

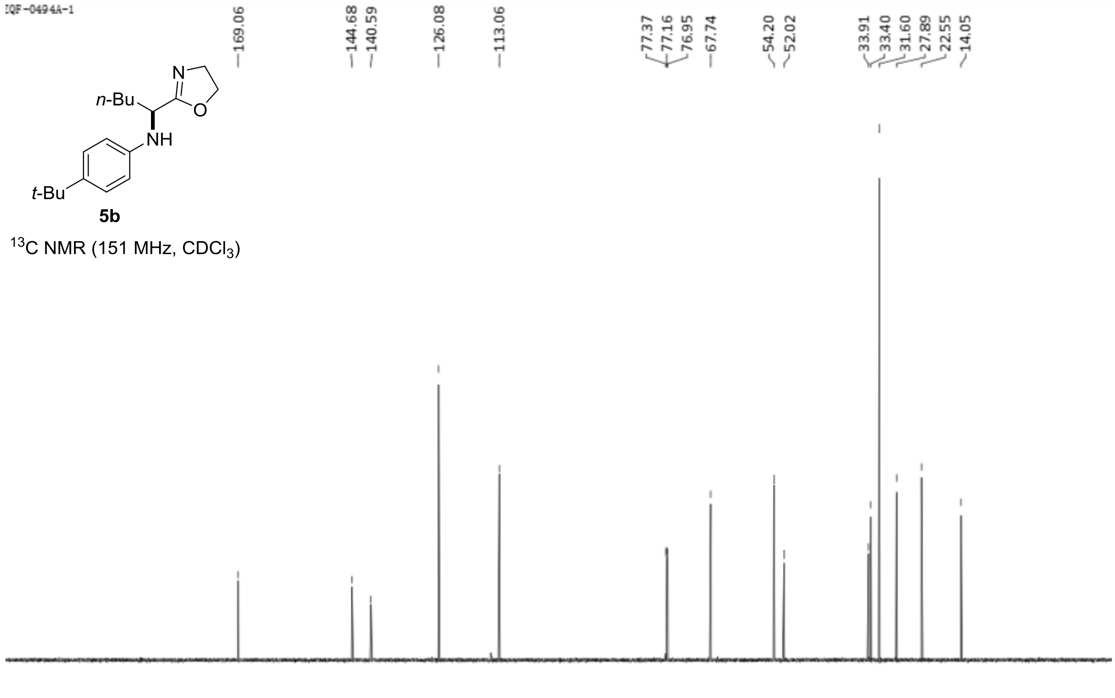


**5b**

$^{13}$C NMR (151 MHz, $CDCl_3$)

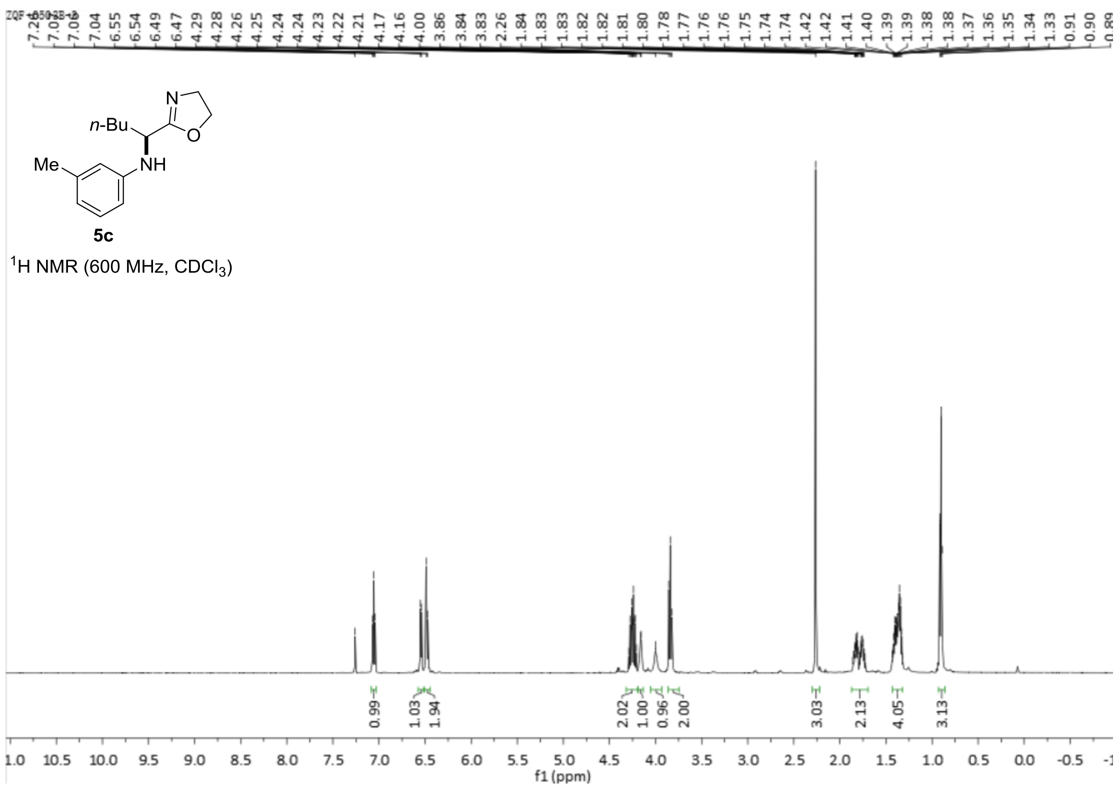
ZQF-0503B-2
7.26 7.06 7.04 7.04 6.55 6.54 6.49 6.47 4.29 4.28 4.26 4.25 4.24 4.24 4.23 4.22 4.21 4.17 4.16 4.00 3.86 3.84 3.83 2.26 1.84 1.83 1.83 1.82 1.82 1.81 1.80 1.78 1.77 1.76 1.76 1.75 1.74 1.74 1.42 1.42 1.41 1.40 1.39 1.39 1.38 1.38 1.37 1.36 1.35 1.34 1.33 0.91 0.90 0.89
n-Bu
N
O
Me
NH
5c
1H NMR (600 MHz, CDCl3)
0.99 1.03 1.94 2.02 1.00 0.96 2.00 3.03 2.13 4.05 3.13
f1 (ppm)

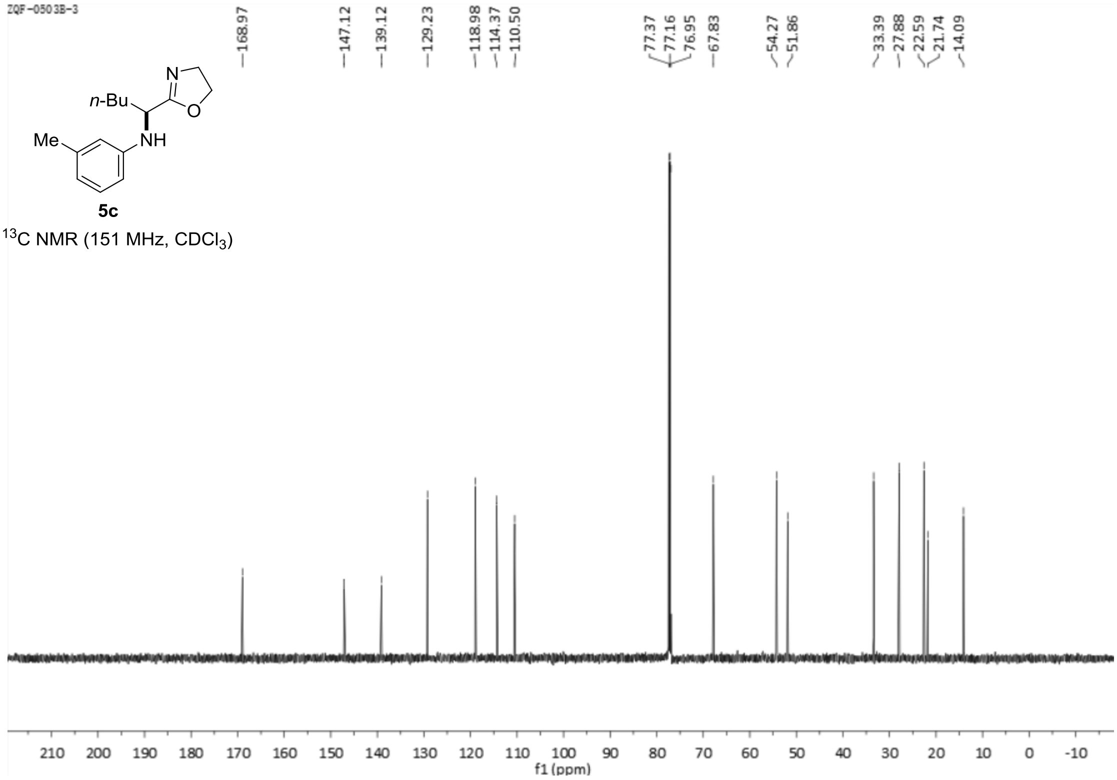
ZQF-0503B-3
168.97 147.12 139.12 129.23 118.98 114.37 110.50 77.37 77.16 76.95 67.83 54.27 51.86 33.39 27.88 22.59 21.74 14.09
n-Bu
N
O
Me
NH
5c
13C NMR (151 MHz, CDCl3)
f1 (ppm)

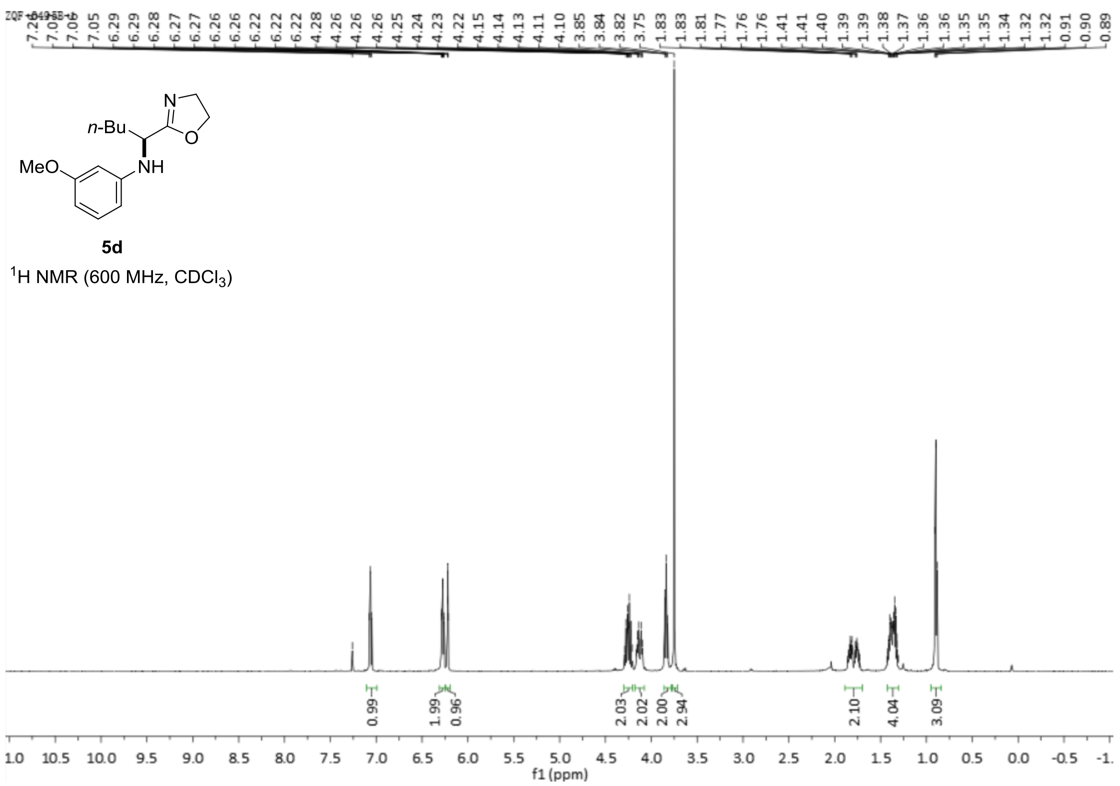

ZQF-0495B-1
n-Bu
N
O
MeO
NH
5d
1H NMR (600 MHz, CDCl3)
0.99
1.99
0.96
2.03
2.02
2.00
2.94
2.10
4.04
3.09
f1 (ppm)


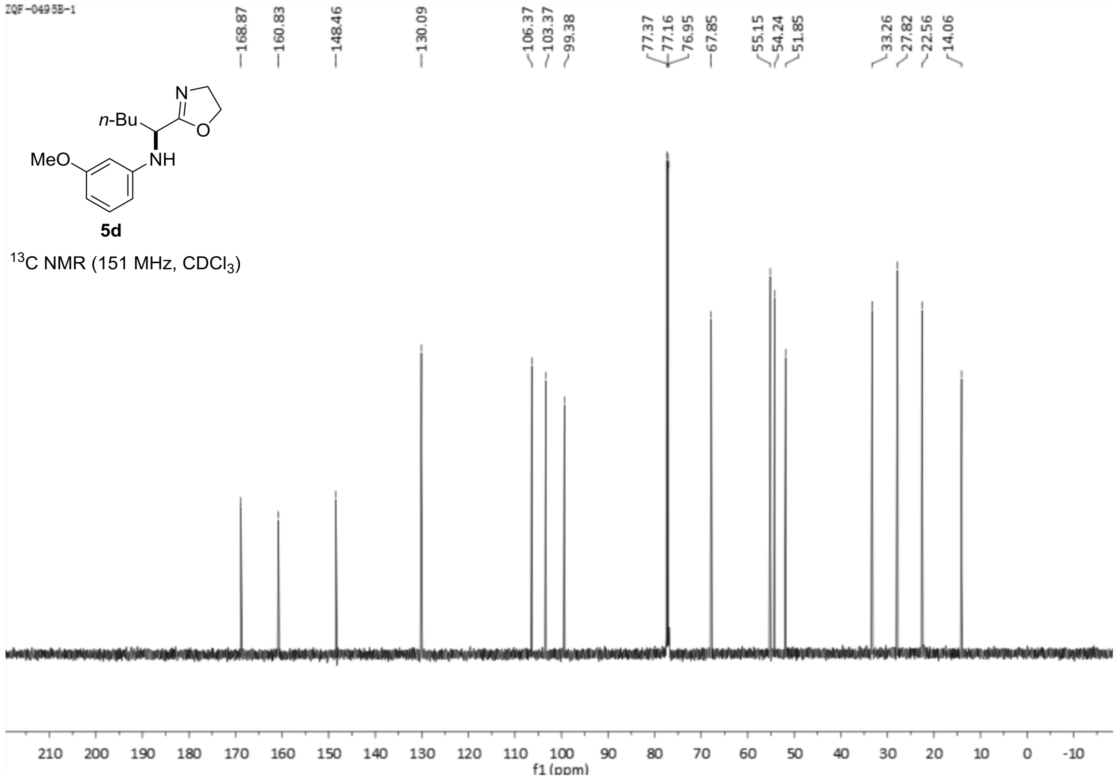

ZQF-0495B-1
168.87
160.83
148.46
130.09
106.37
103.37
99.38
77.37
77.16
76.95
67.85
55.15
54.24
51.85
33.26
27.82
22.56
14.06
n-Bu
N
O
MeO
NH
5d
13C NMR (151 MHz, CDCl3)
f1 (ppm)

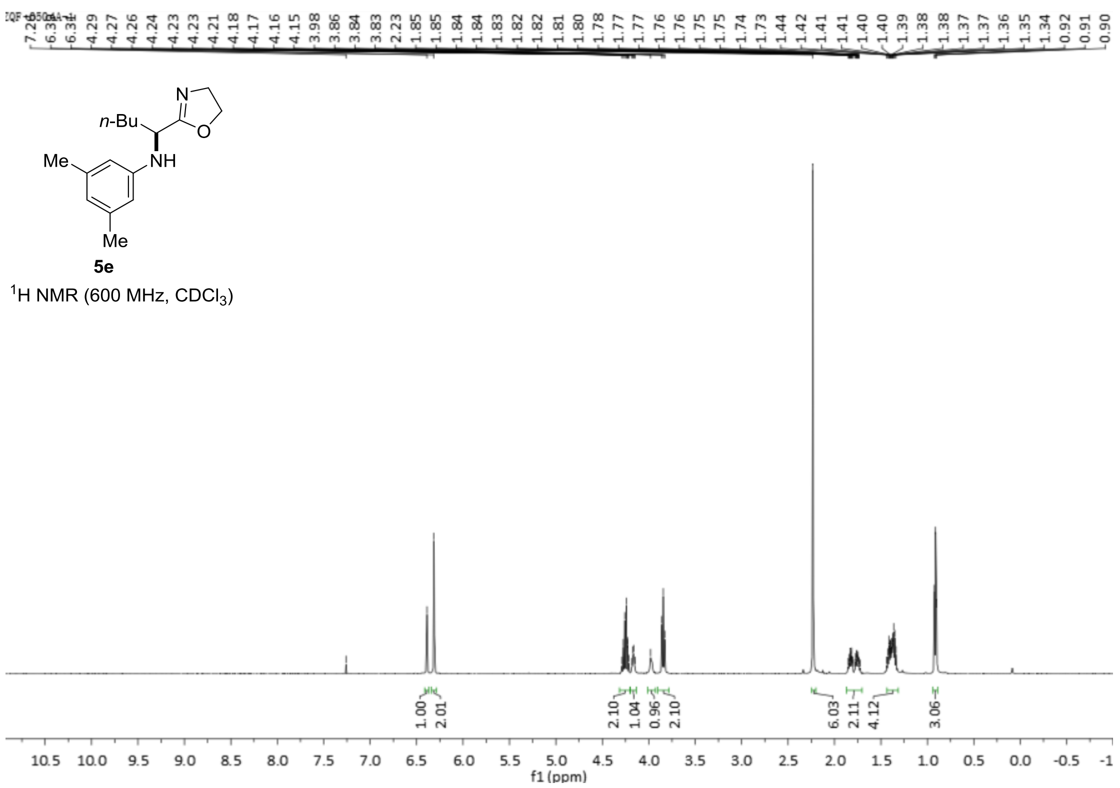
n-Bu
N
O
NH
Me
Me
5e
$^{1}$H NMR (600 MHz, $CDCl_3$)
f1 (ppm)

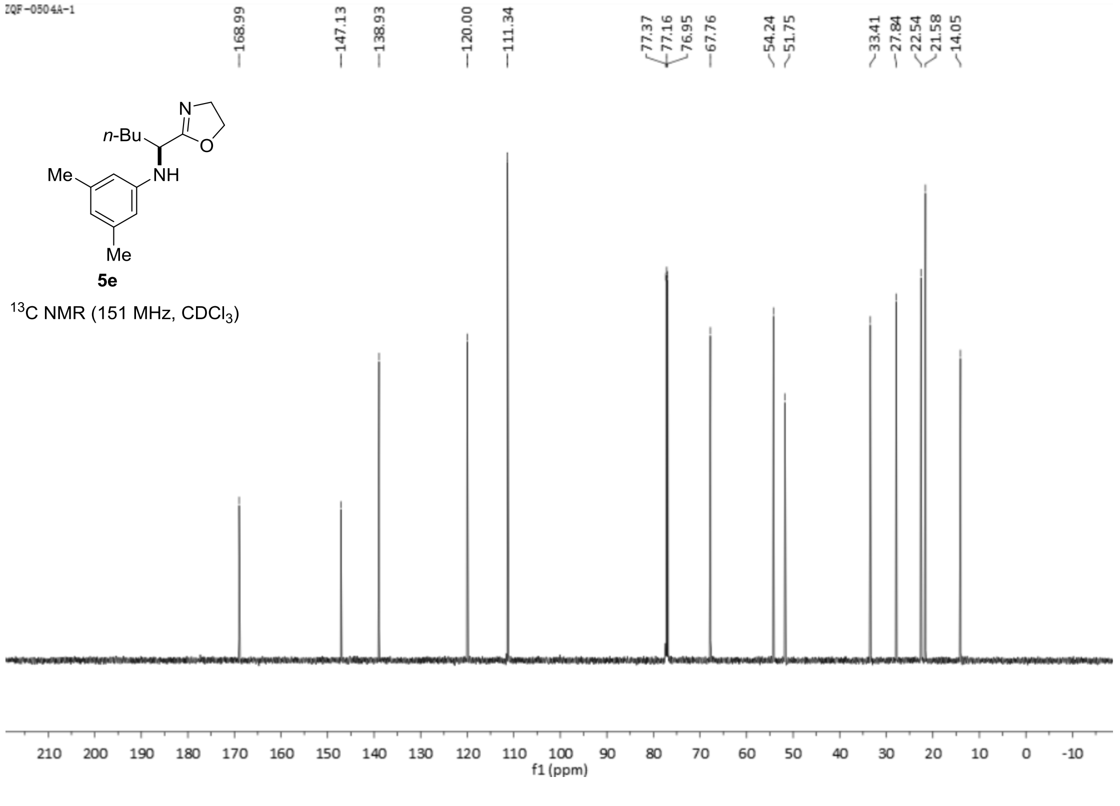
n-Bu
N
O
NH
Me
Me
5e
$^{13}$C NMR (151 MHz, $CDCl_3$)
f1 (ppm)

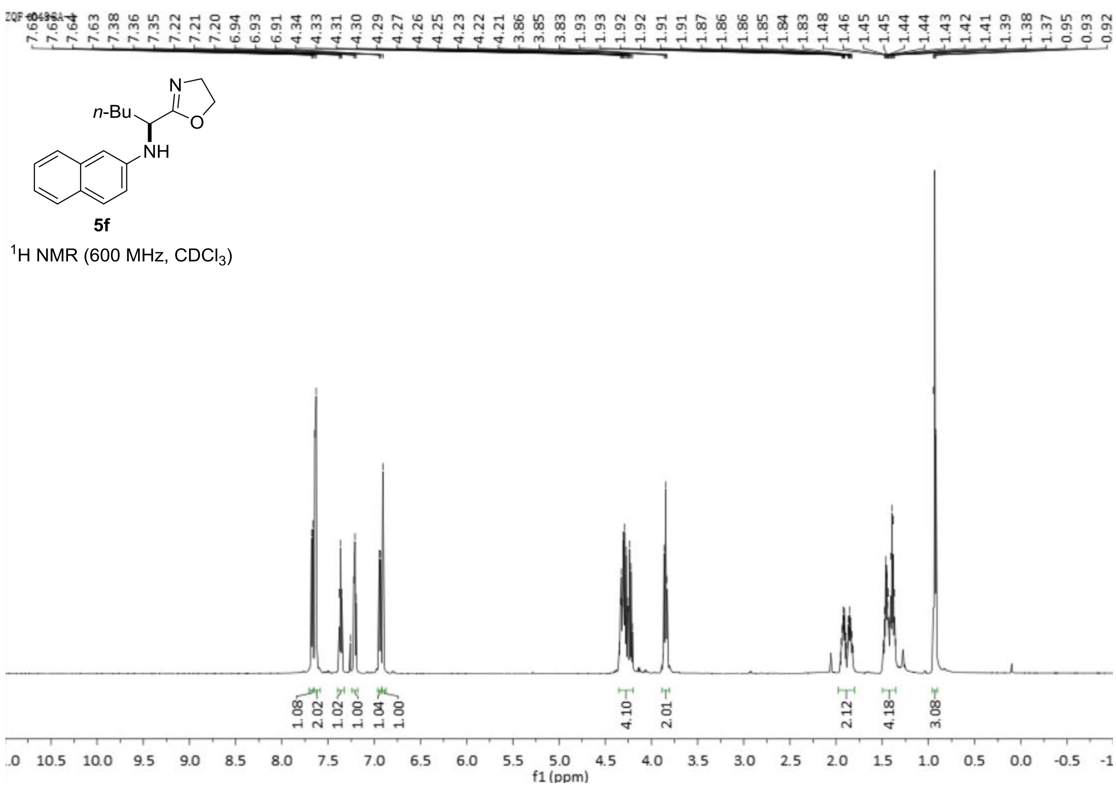

5f

$^1H$ NMR (600 MHz, $CDCl_3$)

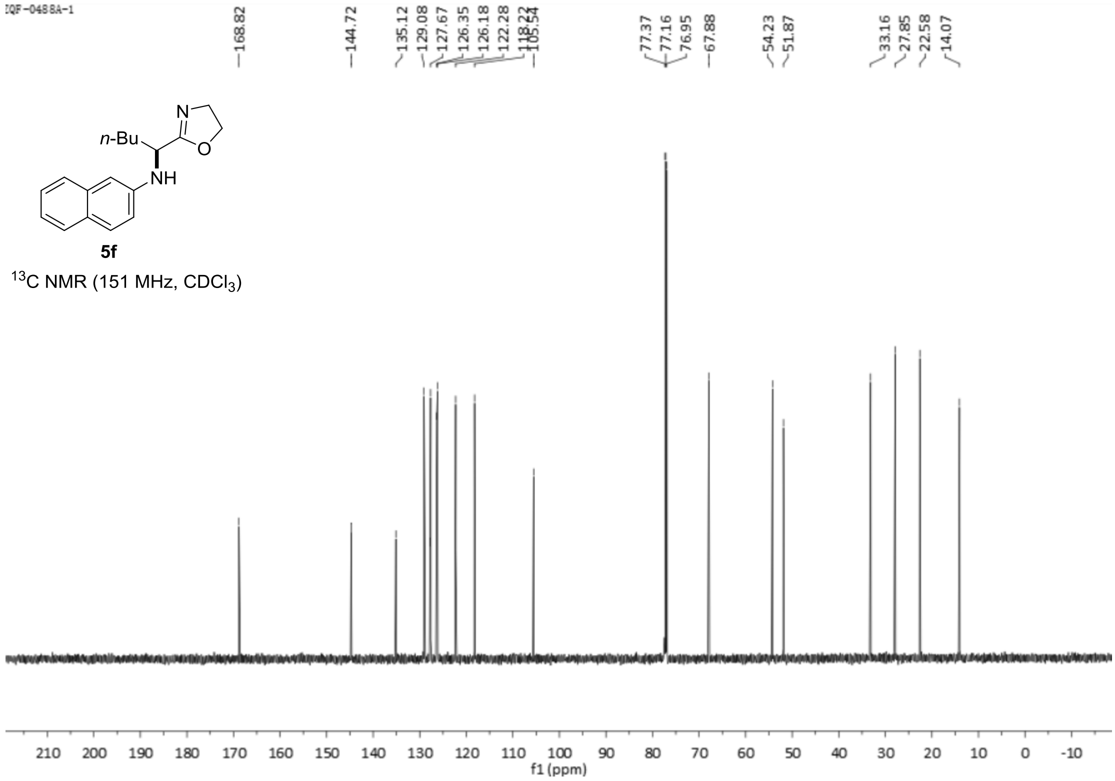

5f

$^{13}C$ NMR (151 MHz, $CDCl_3$)

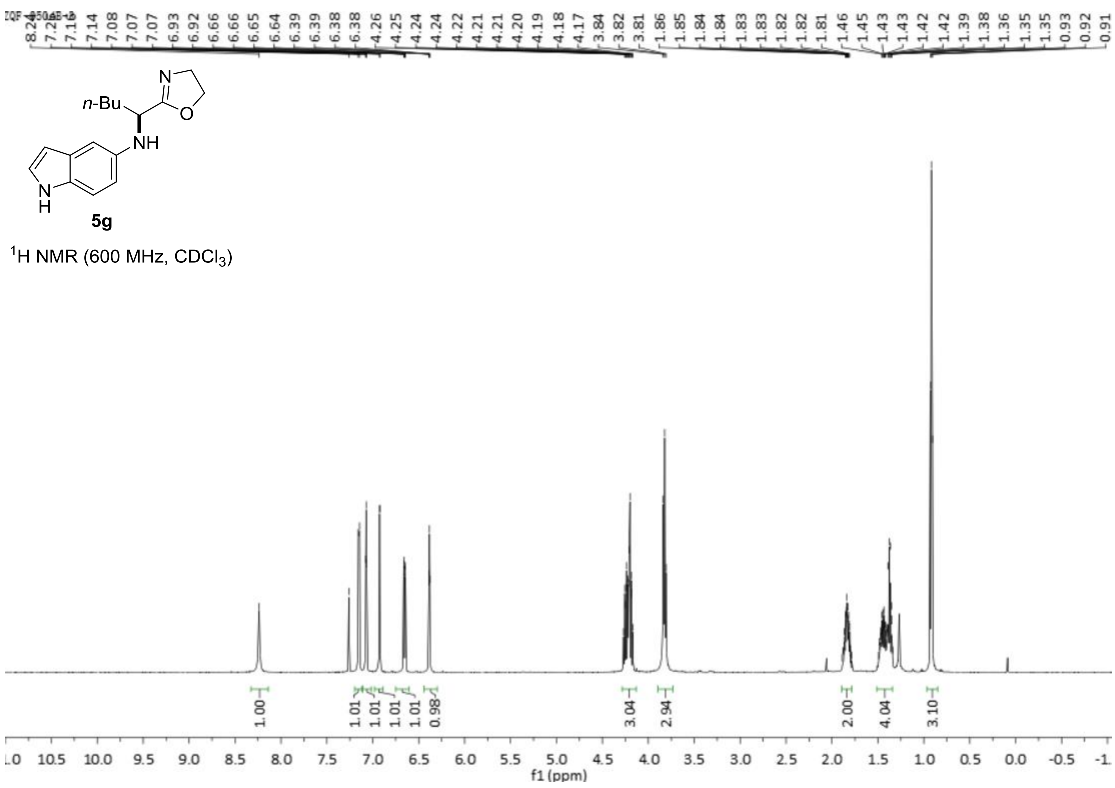

8.24
7.16
7.14
7.08
7.07
7.07
6.93
6.92
6.66
6.66
6.65
6.64
6.39
6.39
6.38
6.38
4.26
4.25
4.24
4.24
4.22
4.21
4.21
4.20
4.19
4.18
4.17
3.84
3.82
3.81
1.86
1.85
1.84
1.84
1.83
1.83
1.82
1.82
1.81
1.46
1.45
1.43
1.43
1.42
1.42
1.39
1.38
1.36
1.35
0.93
0.92
0.91
n-Bu
N
O
NH
N
H
5g
1H NMR (600 MHz, CDCl3)
1.00
1.01
1.01
1.01
1.01
0.98
3.04
2.94
2.00
4.04
3.10
f1 (ppm)


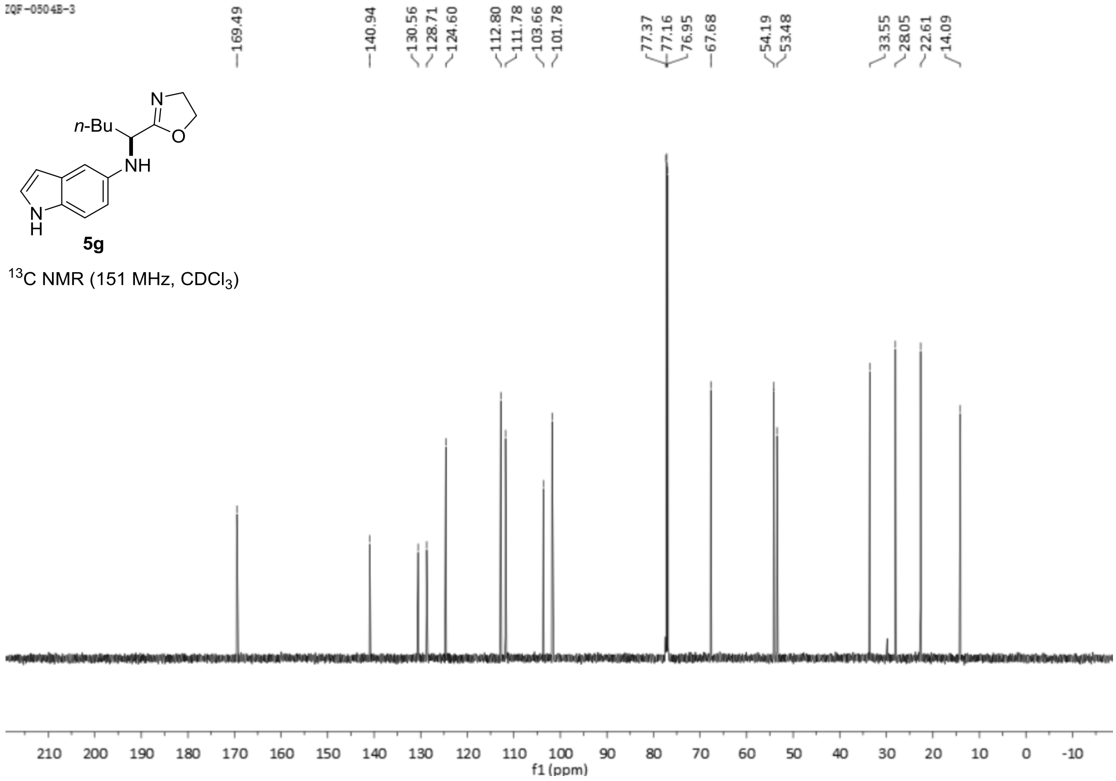

ZQF-0504B-3
169.49
140.94
130.56
128.71
124.60
112.80
111.78
103.66
101.78
77.37
77.16
76.95
67.68
54.19
53.48
33.55
28.05
22.61
14.09
n-Bu
N
O
NH
N
H
5g
13C NMR (151 MHz, CDCl3)
f1 (ppm)

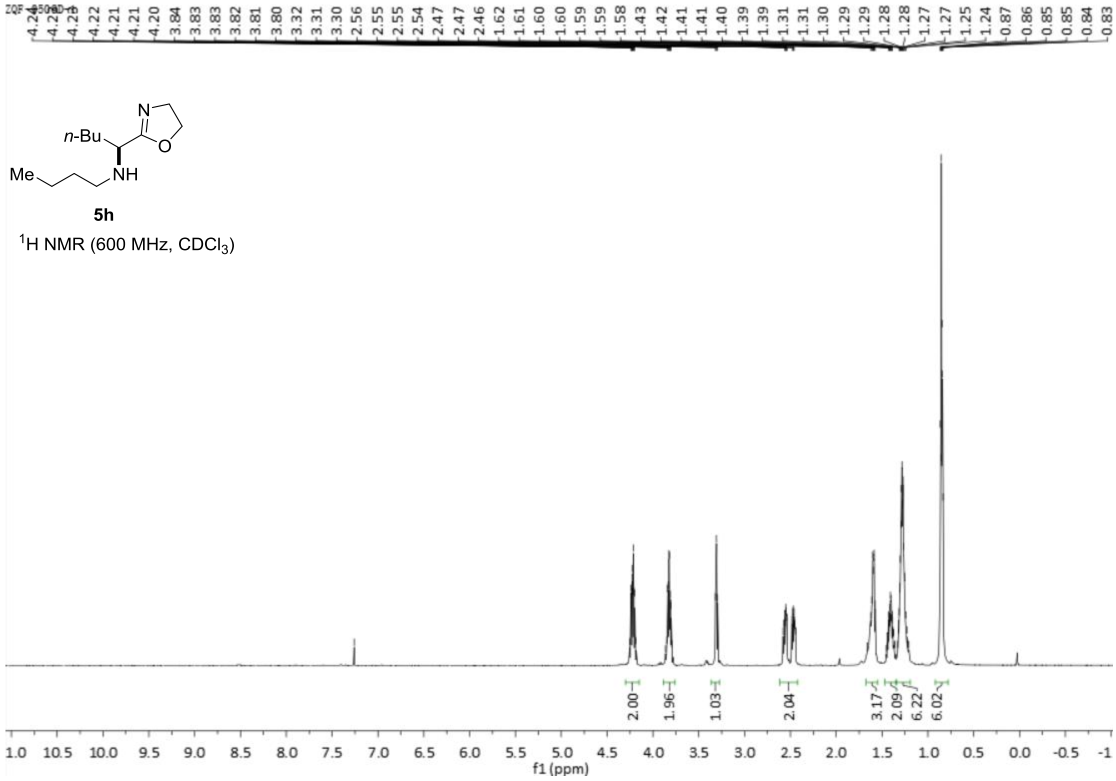
n-Bu
N
O
Me
NH
5h
1H NMR (600 MHz, CDCl3)
f1 (ppm)

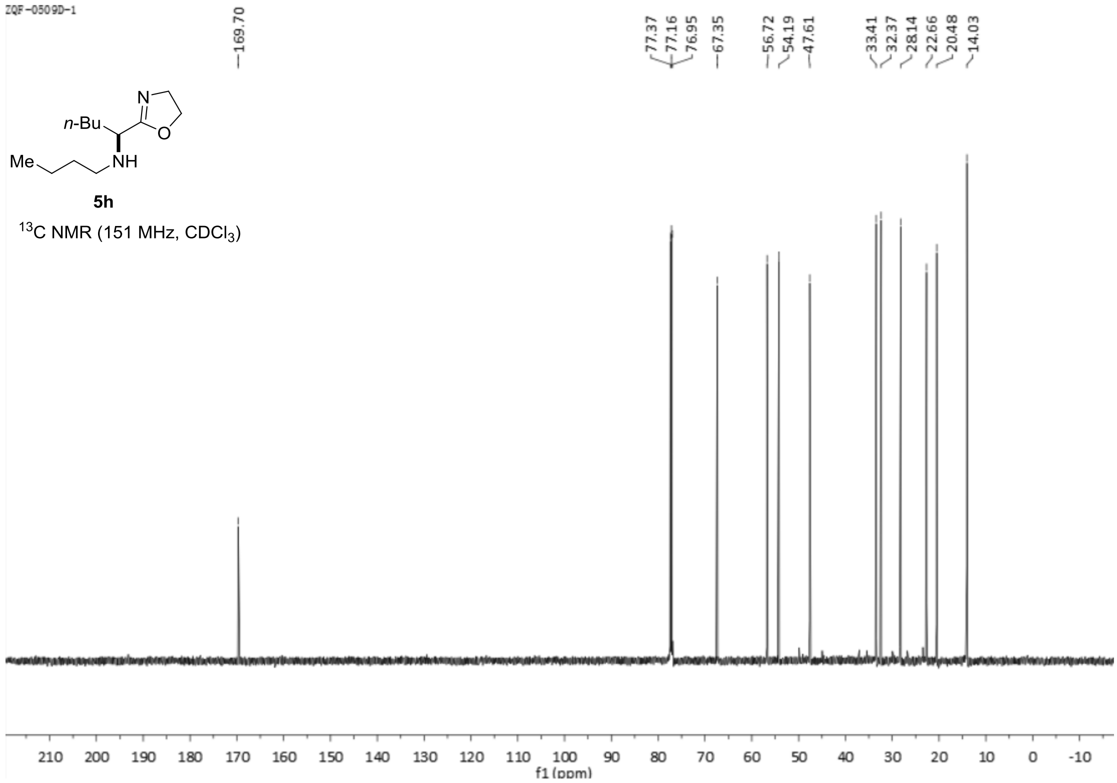
ZQF-0509D-1
169.70
77.37
77.16
76.95
67.35
56.72
54.19
47.61
33.41
32.37
28.14
22.66
20.48
14.03
n-Bu
N
O
Me
NH
5h
13C NMR (151 MHz, CDCl3)
f1 (ppm)

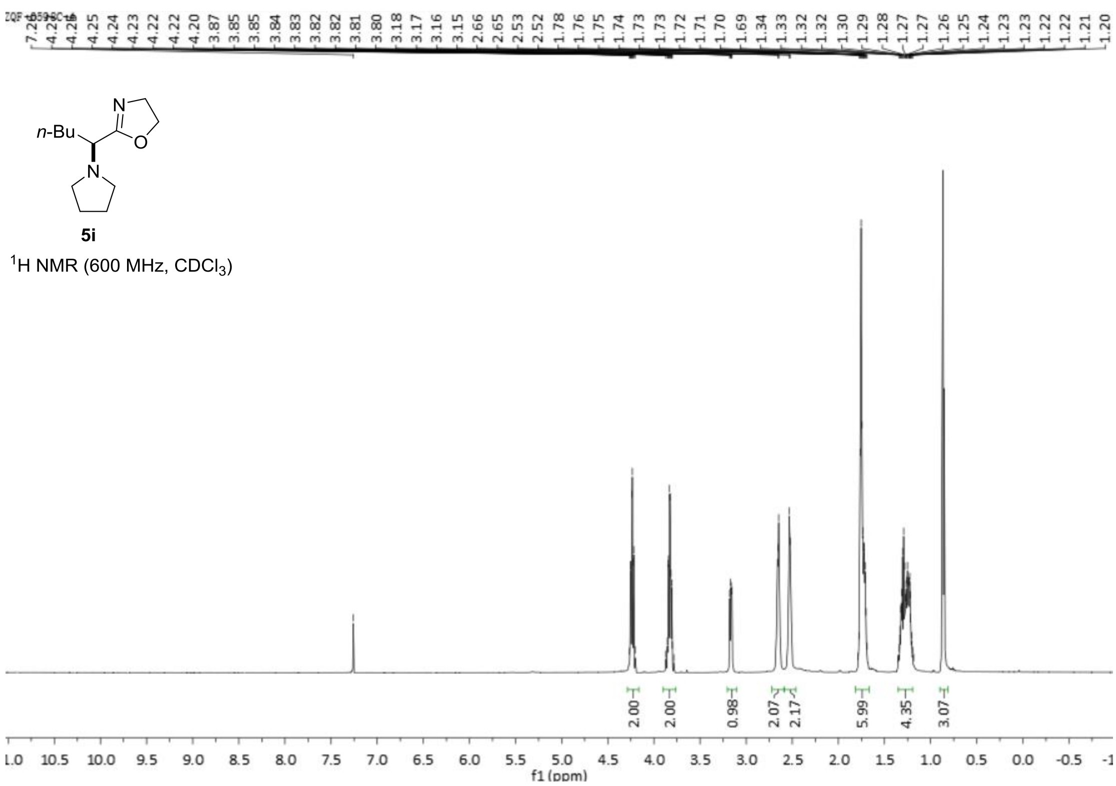


**5i**

$^{1}$H NMR (600 MHz, $CDCl_3$)

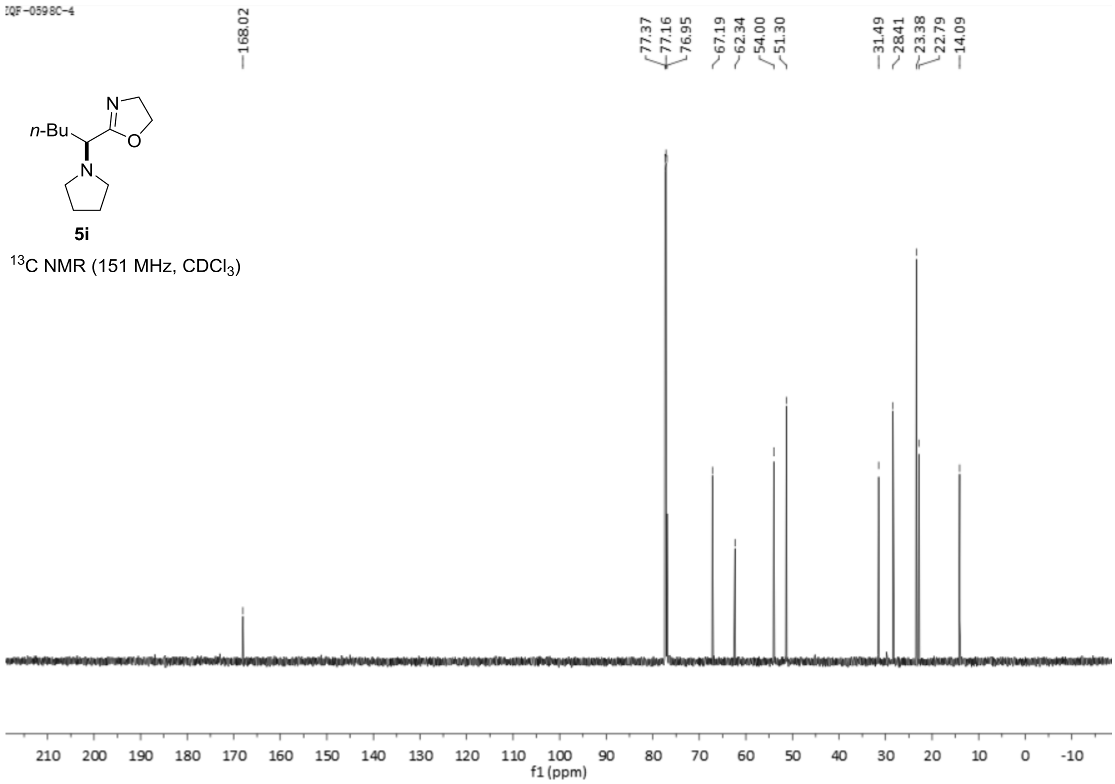


**5i**

$^{13}$C NMR (151 MHz, $CDCl_3$)

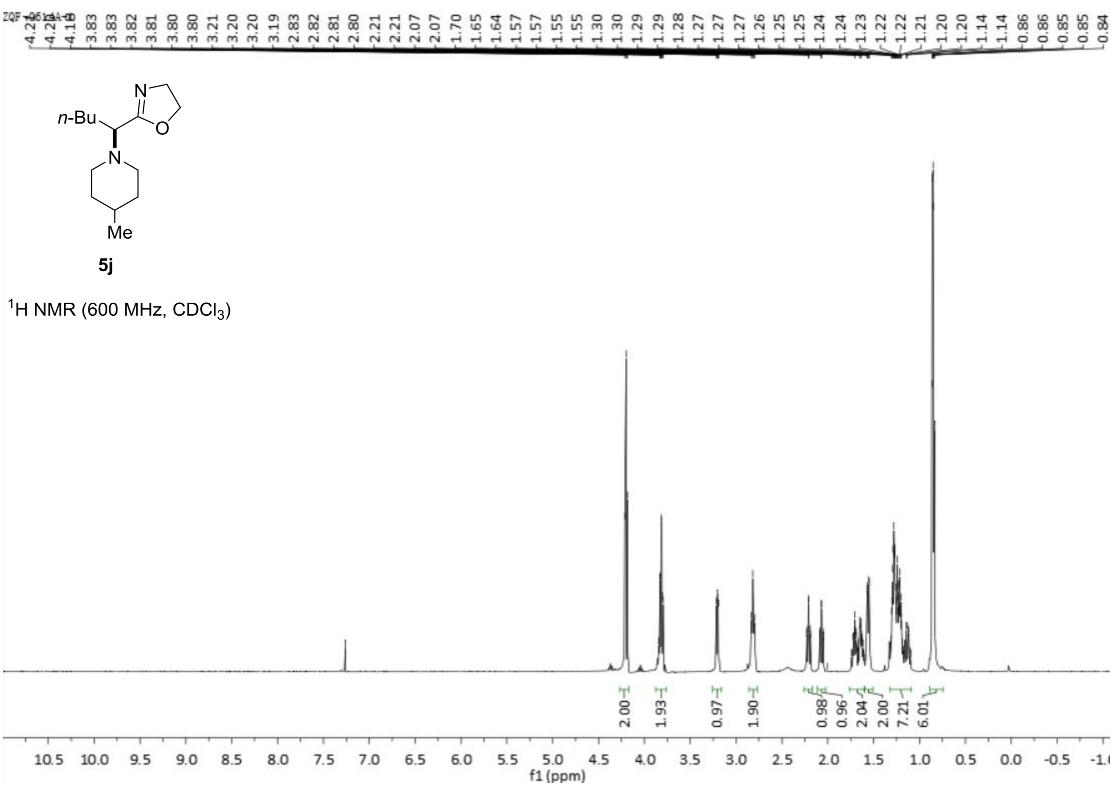

3.83
3.83
3.82
3.81
3.80
3.80
3.21
3.20
3.20
3.19
2.83
2.82
2.81
2.80
2.21
2.21
2.07
2.07
1.70
1.65
1.64
1.57
1.57
1.55
1.55
1.30
1.30
1.29
1.28
1.27
1.27
1.27
1.26
1.25
1.25
1.24
1.24
1.23
1.22
1.22
1.21
1.20
1.20
1.14
1.14
0.86
0.85
0.85
0.84
n-Bu
N
O
Me
5j
1H NMR (600 MHz, CDCl3)
2.00
1.93
0.97
1.90
0.98
0.96
2.04
2.00
7.21
6.01
f1 (ppm)


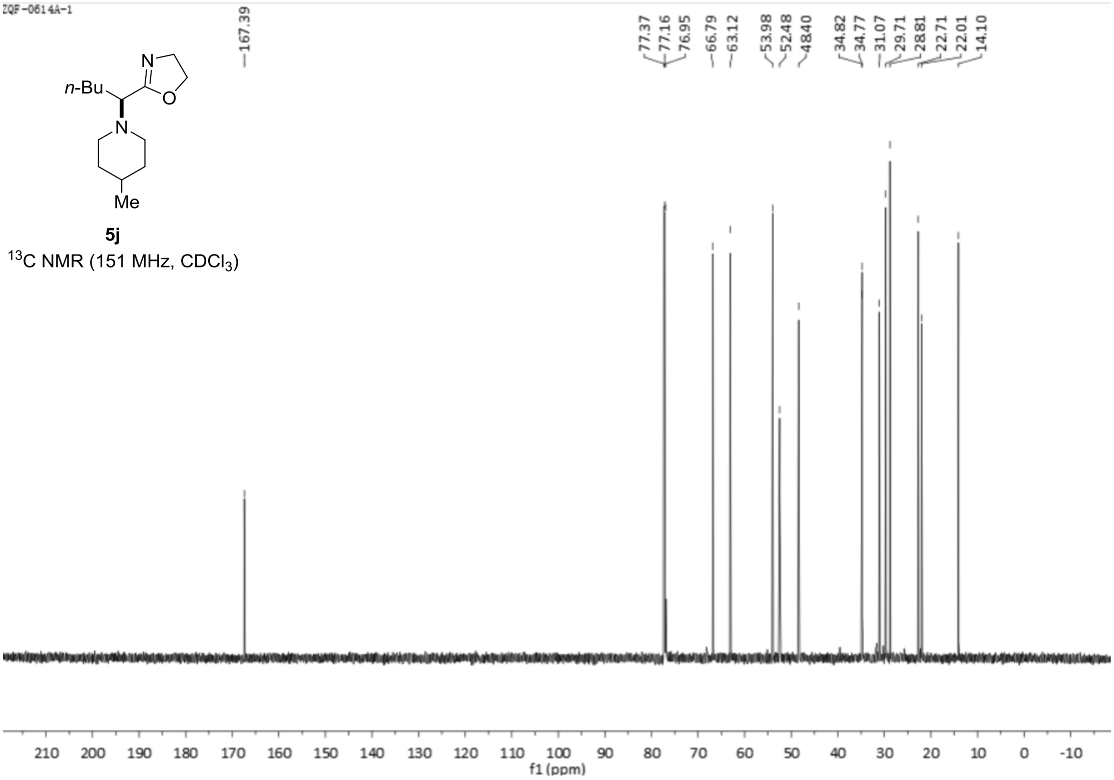

ZQF-0614A-1
167.39
77.37
77.16
76.95
66.79
63.12
53.98
52.48
48.40
34.82
34.77
31.07
29.71
28.81
22.71
22.01
14.10
n-Bu
N
O
Me
5j
13C NMR (151 MHz, CDCl3)
f1 (ppm)

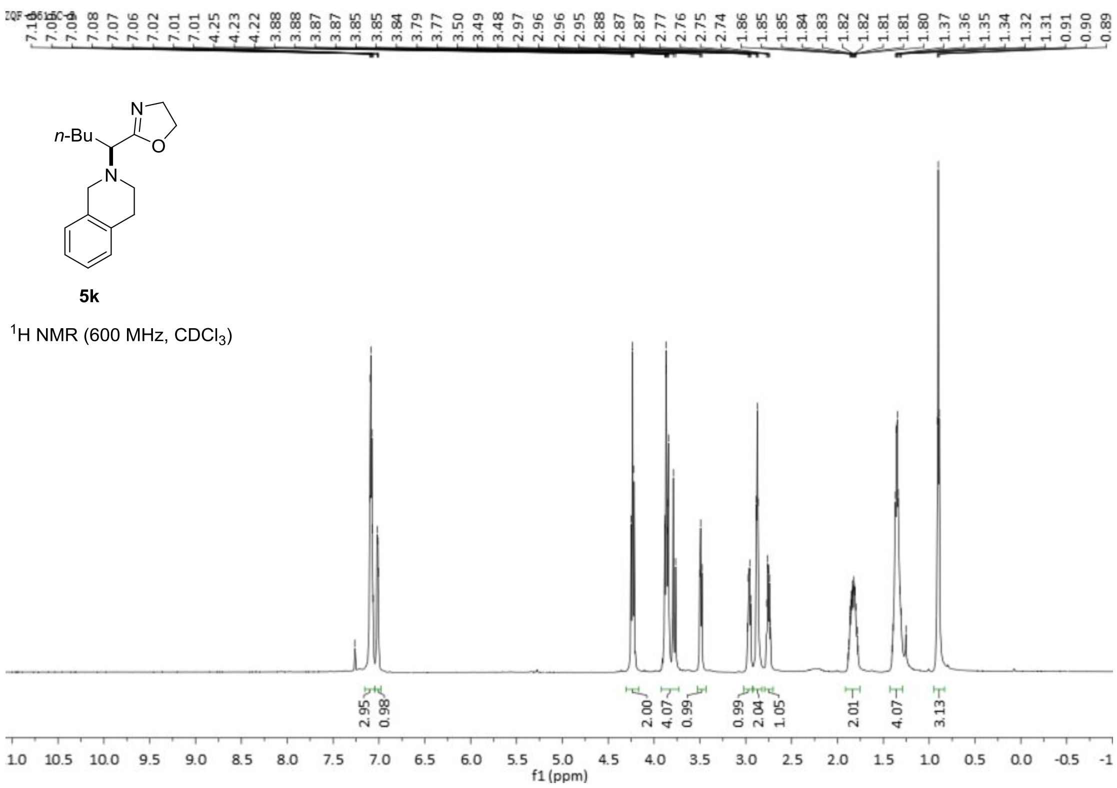


**5k**

$^{1}$H NMR (600 MHz, $CDCl_3$)

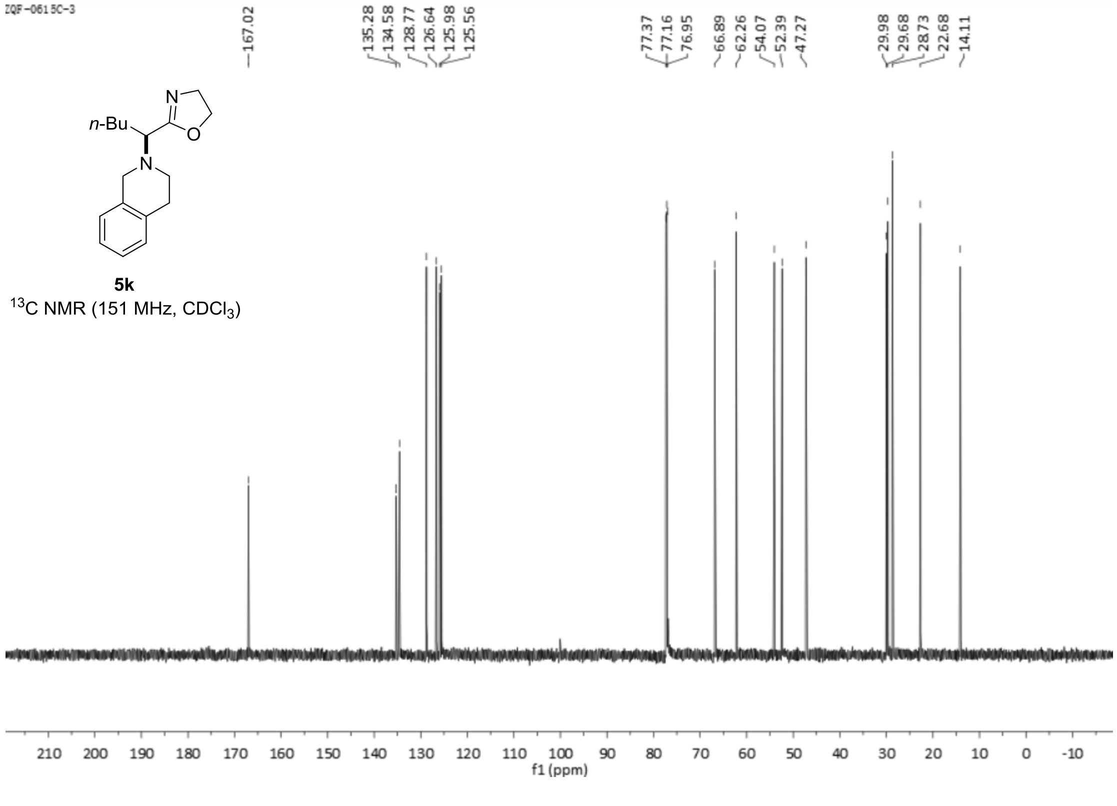


**5k**

$^{13}$C NMR (151 MHz, $CDCl_3$)

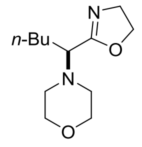

**5l**

$^1H$ NMR (600 MHz, $CDCl_3$)

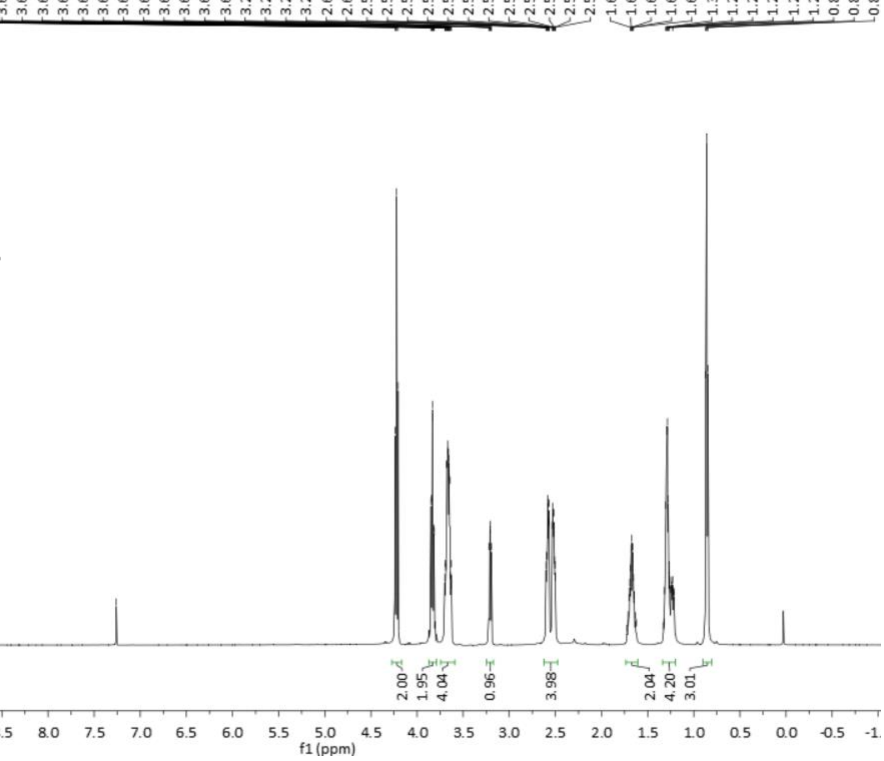


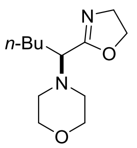

**5l**

$^{13}C$ NMR (151 MHz, $CDCl_3$)

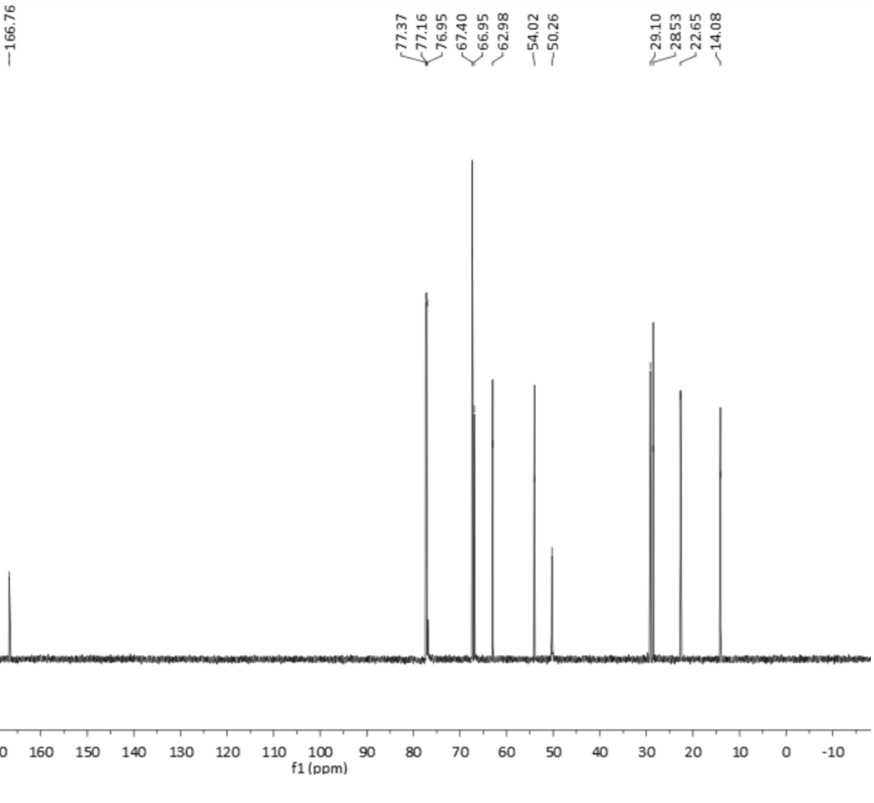

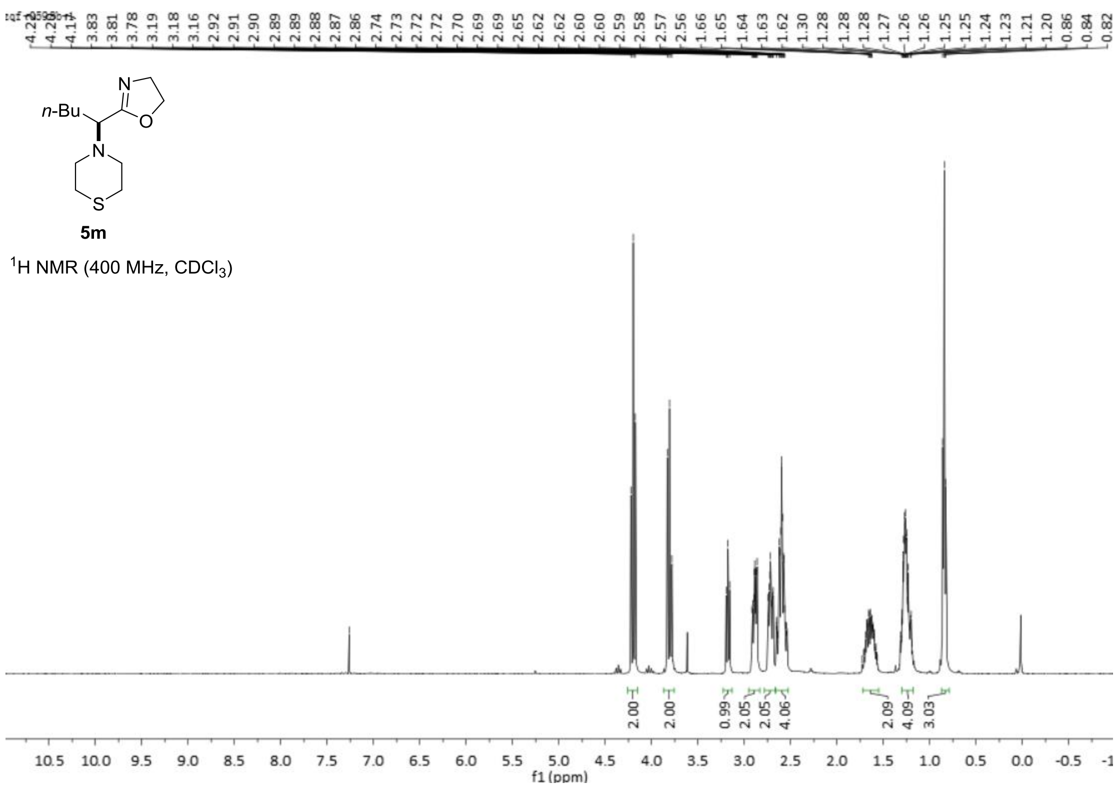

5m

$^{1}H$ NMR (400 MHz, $CDCl_3$)

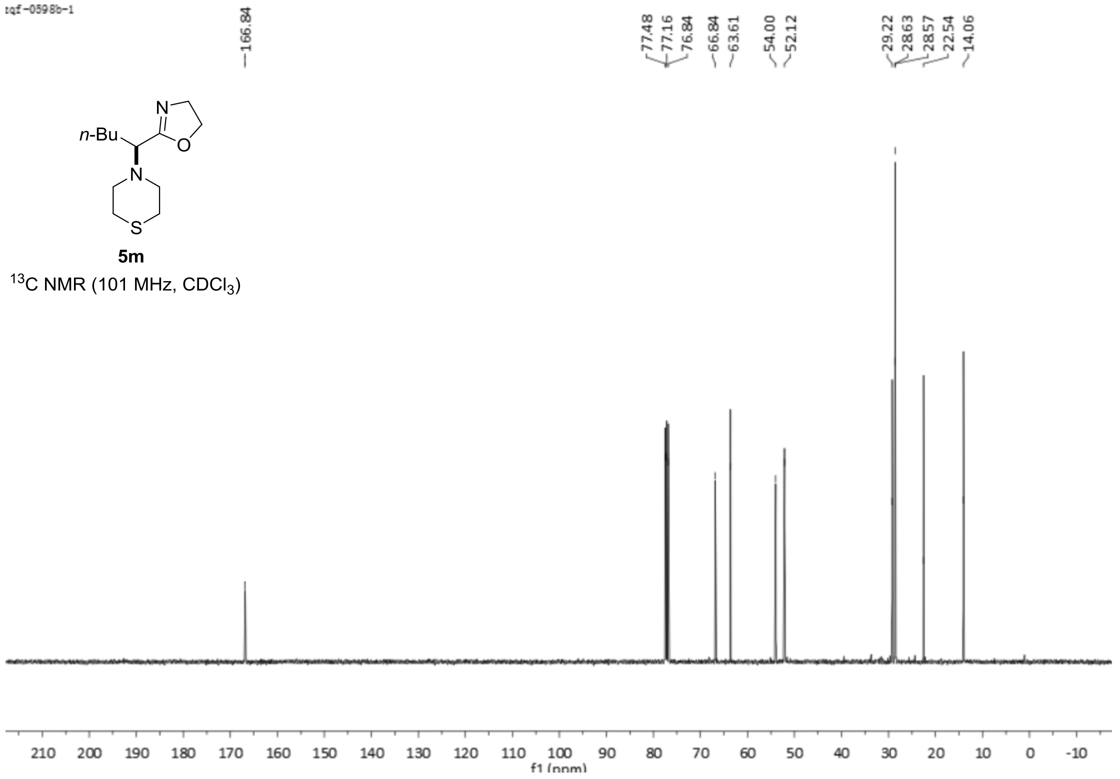

5m

$^{13}C$ NMR (101 MHz, $CDCl_3$)

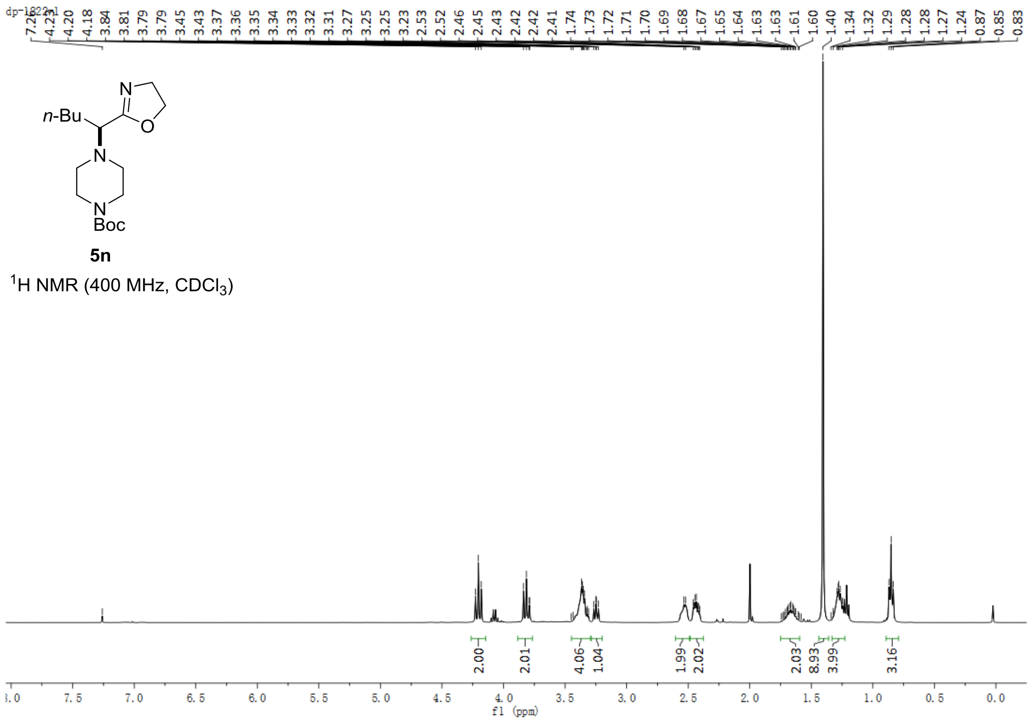


**5n**

$^{1}H$ NMR (400 MHz, $CDCl_3$)

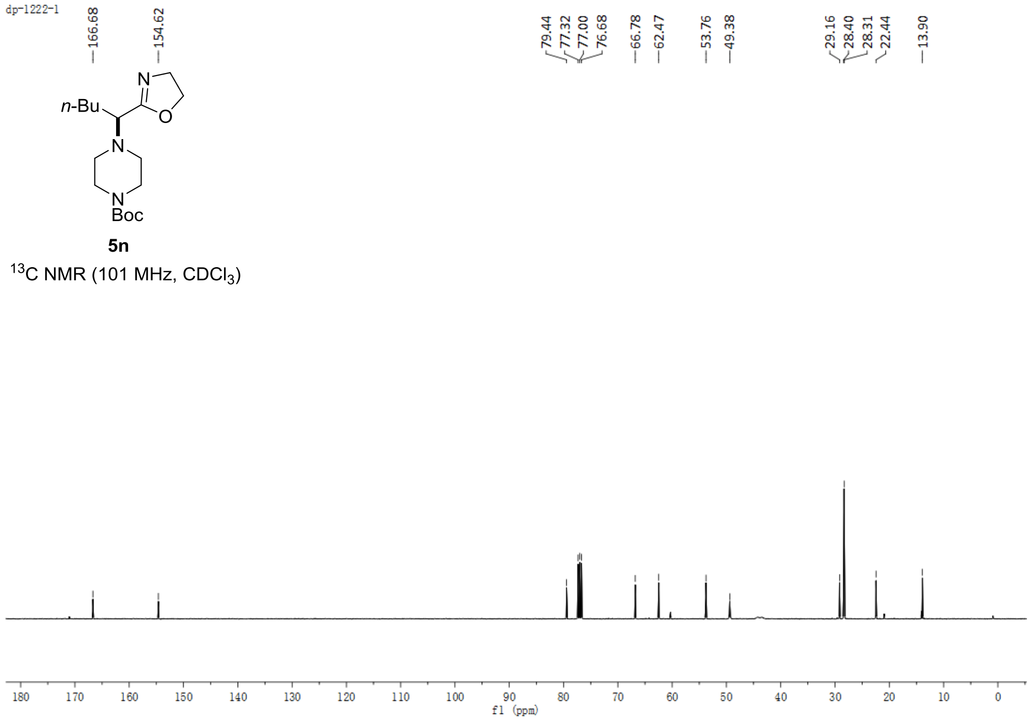


**5n**

$^{13}C$ NMR (101 MHz, $CDCl_3$)

dp-1222-2

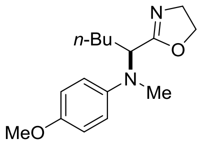


**5o**

$^{1}$H NMR (400 MHz, $CDCl_3$)

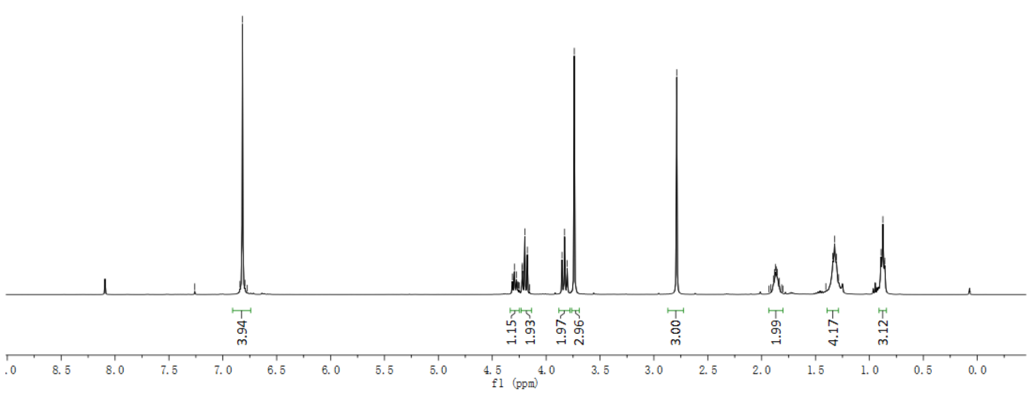


dp-1222-2

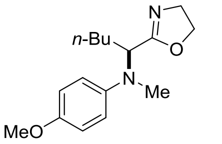


**5o**

$^{13}$C NMR (101 MHz, $CDCl_3$)

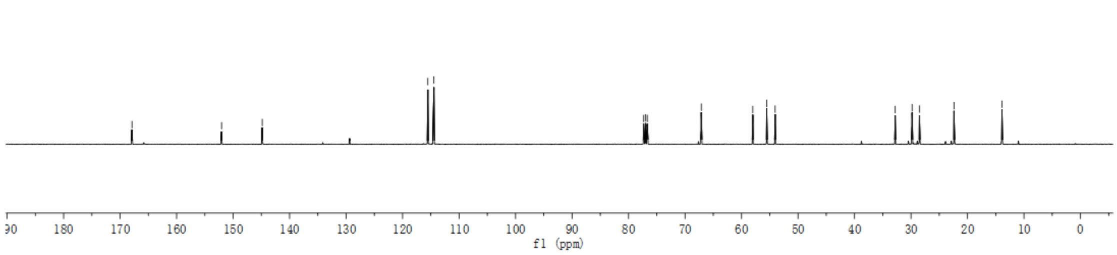

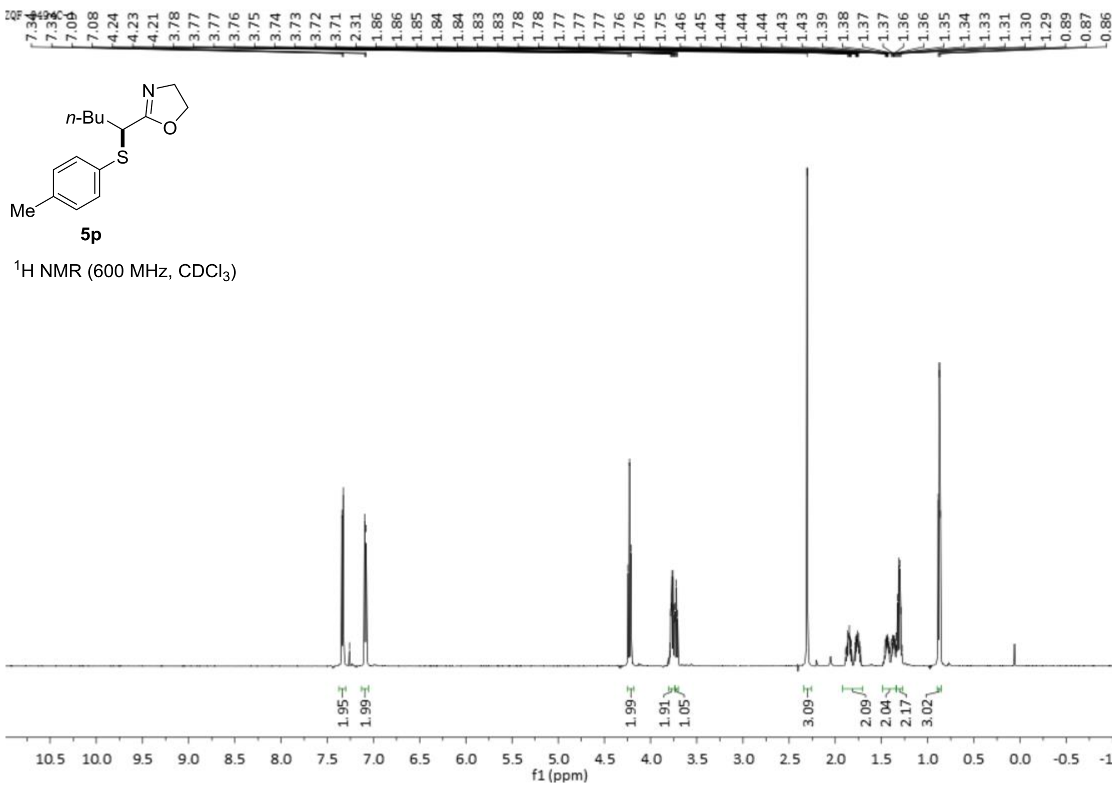
n-Bu
N
O
S
Me
5p
$^{1}$H NMR (600 MHz, $CDCl_3$)
f1 (ppm)

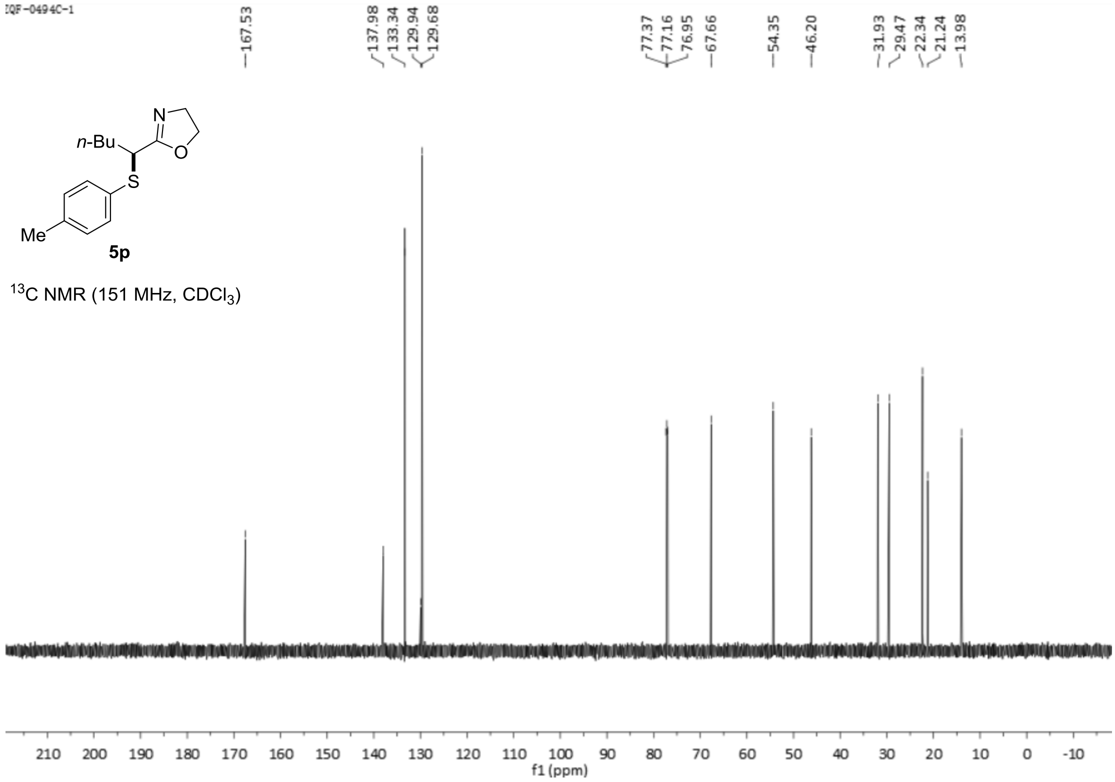
167.53
137.98
133.34
129.94
129.68
77.37
77.16
76.95
67.66
54.35
46.20
31.93
29.47
22.34
21.24
13.98
n-Bu
N
O
S
Me
5p
$^{13}$C NMR (151 MHz, $CDCl_3$)
f1 (ppm)

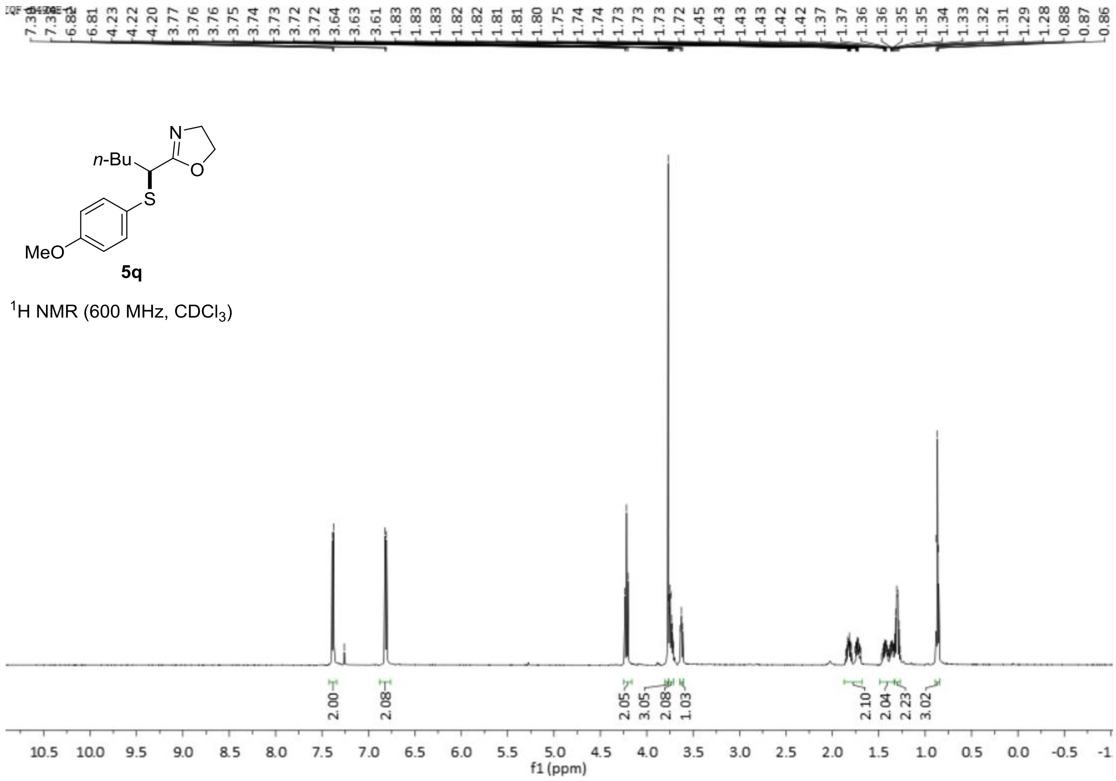


$^{1}$H NMR (600 MHz, $CDCl_3$)

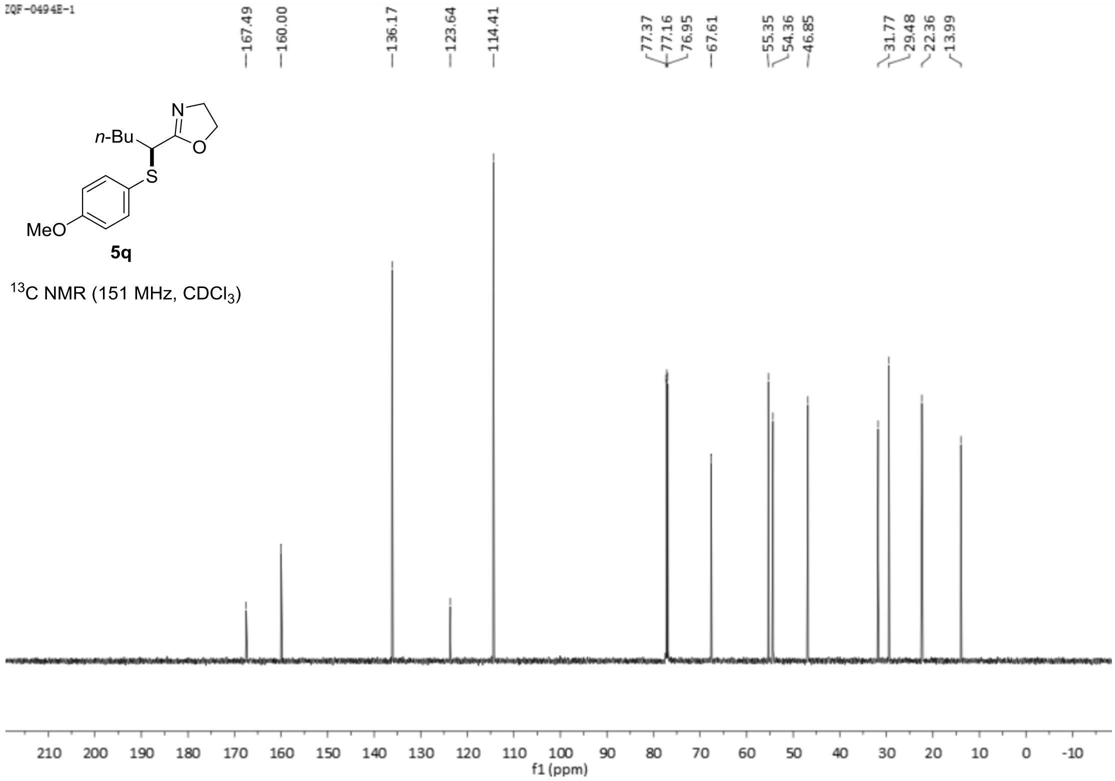


$^{13}$C NMR (151 MHz, $CDCl_3$)

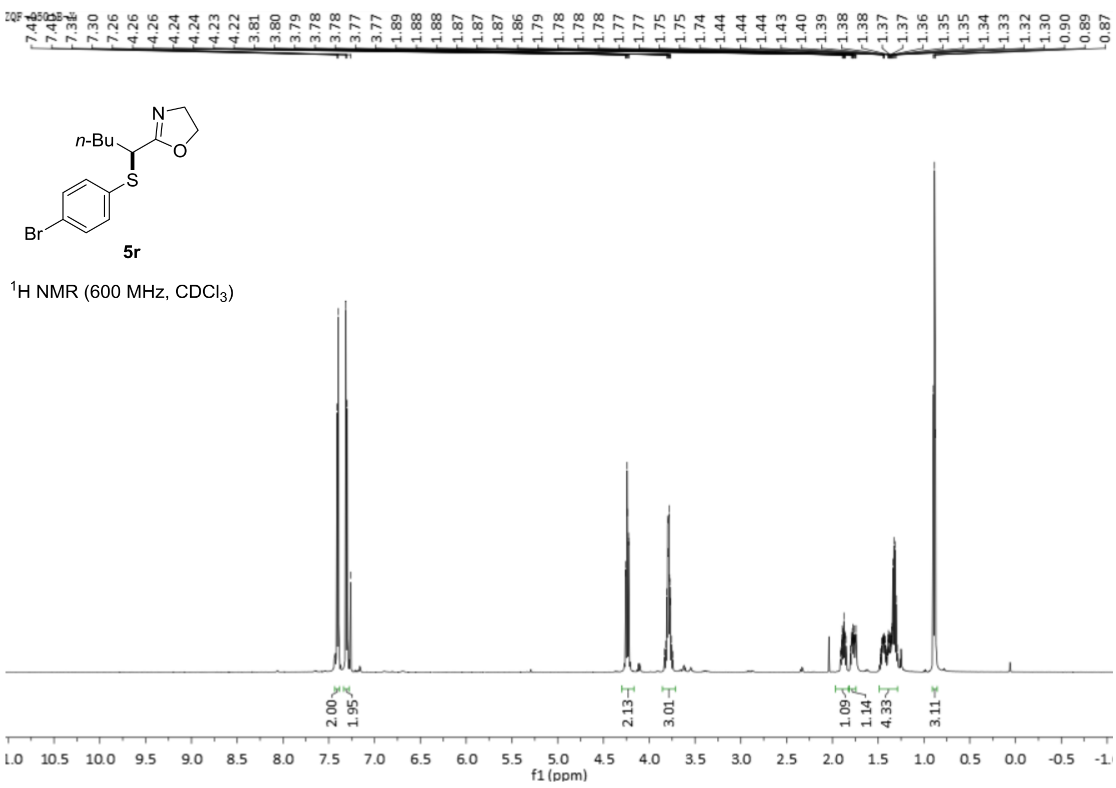
ZQF-0501B-H
7.41
7.40
7.31
7.30
7.26
4.26
4.26
4.24
4.24
4.23
4.22
3.81
3.80
3.79
3.78
3.78
3.77
3.77
1.89
1.88
1.88
1.87
1.87
1.87
1.86
1.79
1.78
1.78
1.78
1.77
1.77
1.75
1.75
1.74
1.44
1.44
1.44
1.43
1.40
1.39
1.38
1.38
1.37
1.37
1.36
1.35
1.35
1.34
1.33
1.32
1.30
0.90
0.89
0.87
N
O
n-Bu
S
Br
5r
$^{1}$H NMR (600 MHz, $CDCl_3$)
2.00
1.95
2.13
3.01
1.09
1.14
4.33
3.11
f1 (ppm)

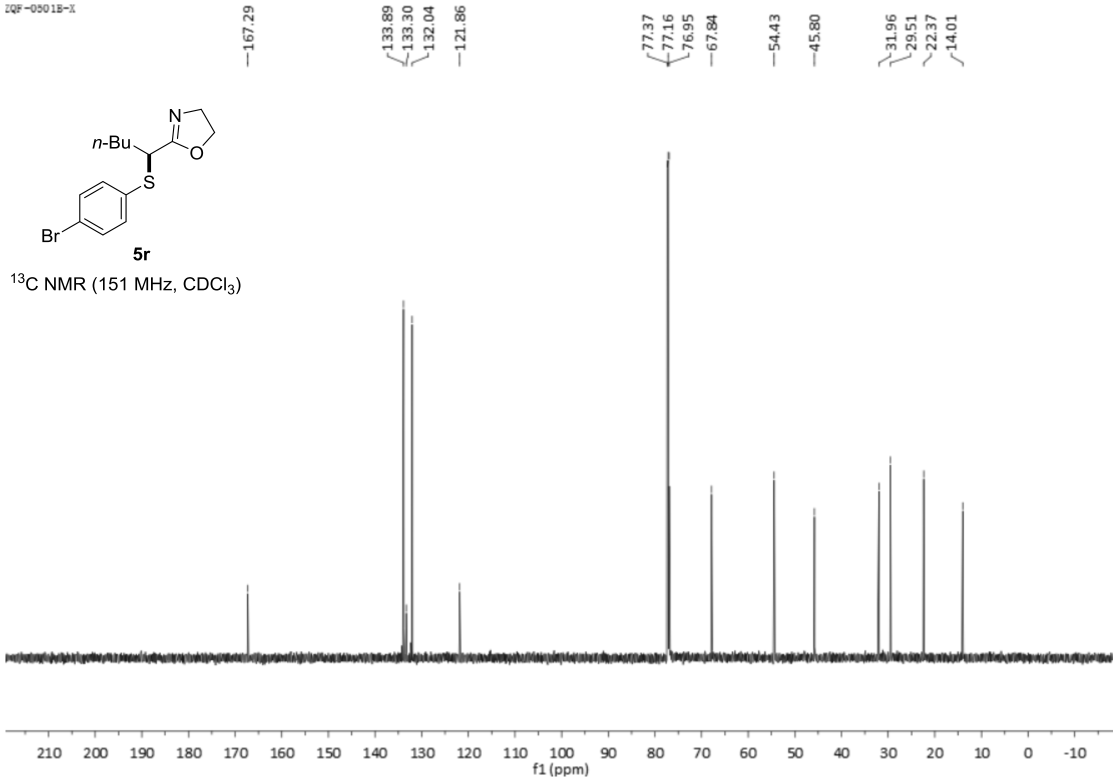
ZQF-0501B-X
167.29
133.89
133.30
132.04
121.86
77.37
77.16
76.95
67.84
54.43
45.80
31.96
29.51
22.37
14.01
N
O
n-Bu
S
Br
5r
$^{13}$C NMR (151 MHz, $CDCl_3$)
f1 (ppm)

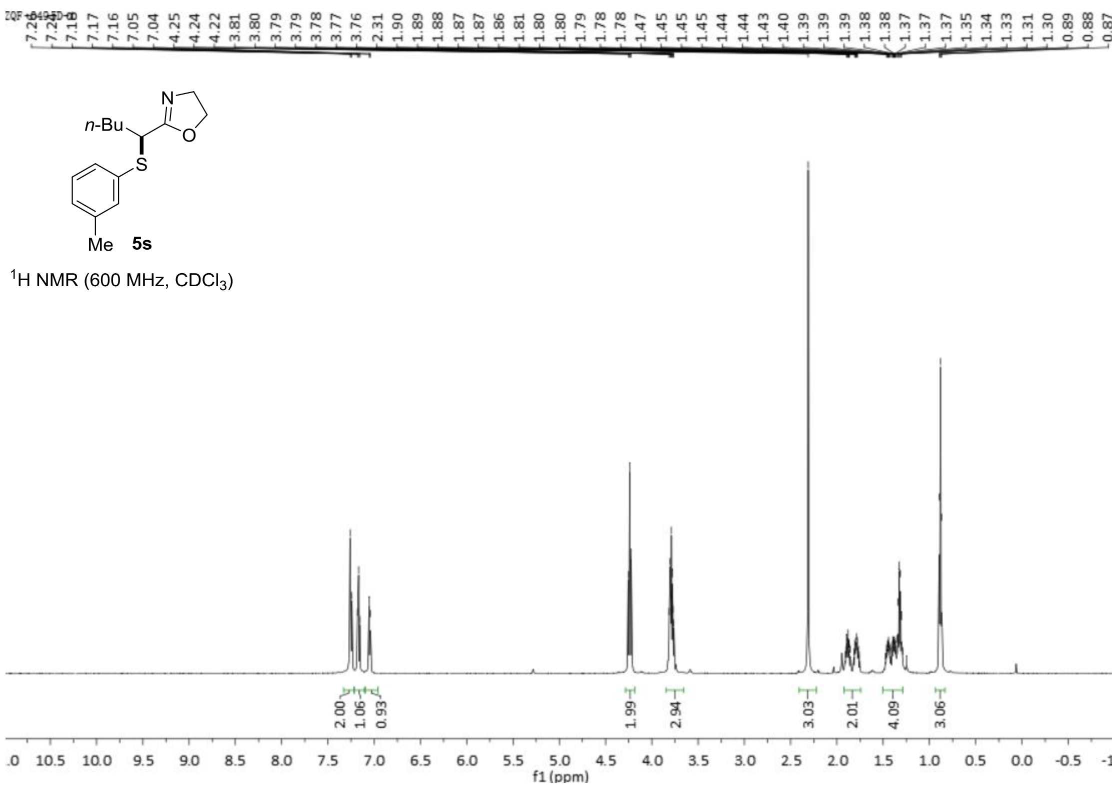

$^{1}H$ NMR (600 MHz, $CDCl_3$)

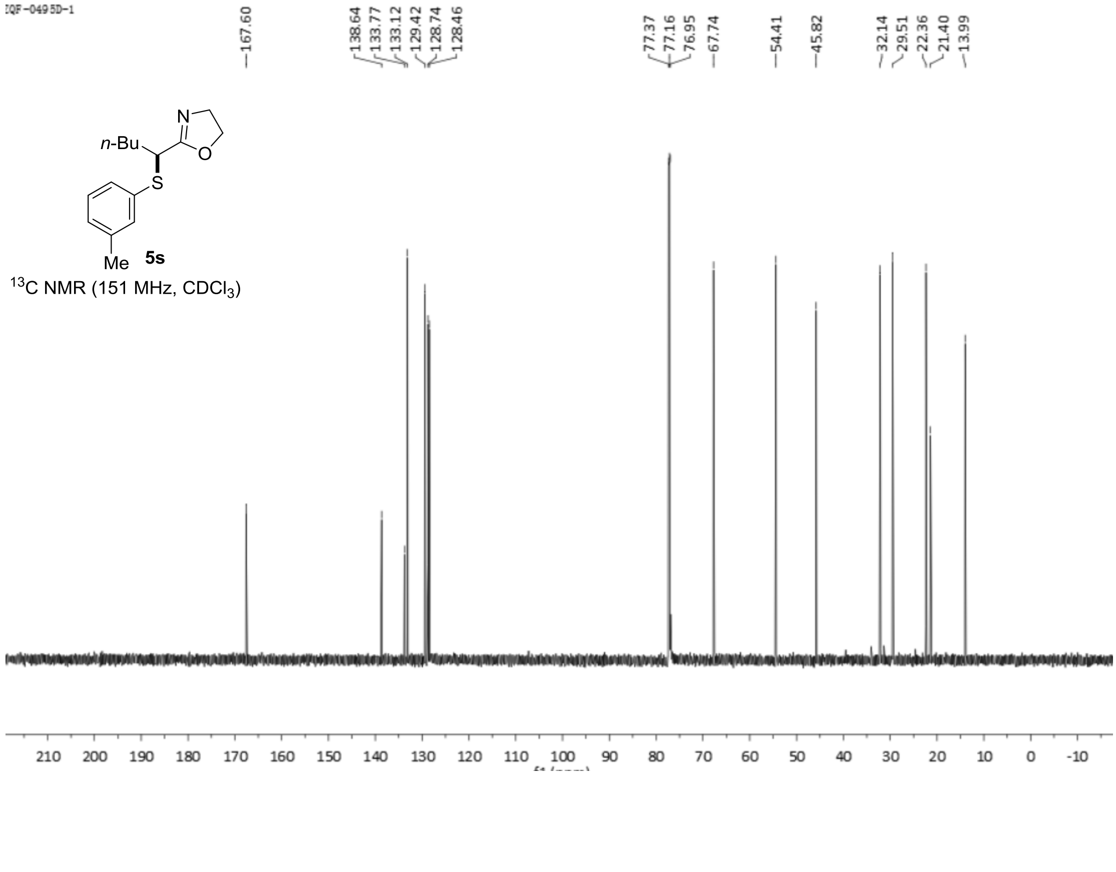

$^{13}C$ NMR (151 MHz, $CDCl_3$)

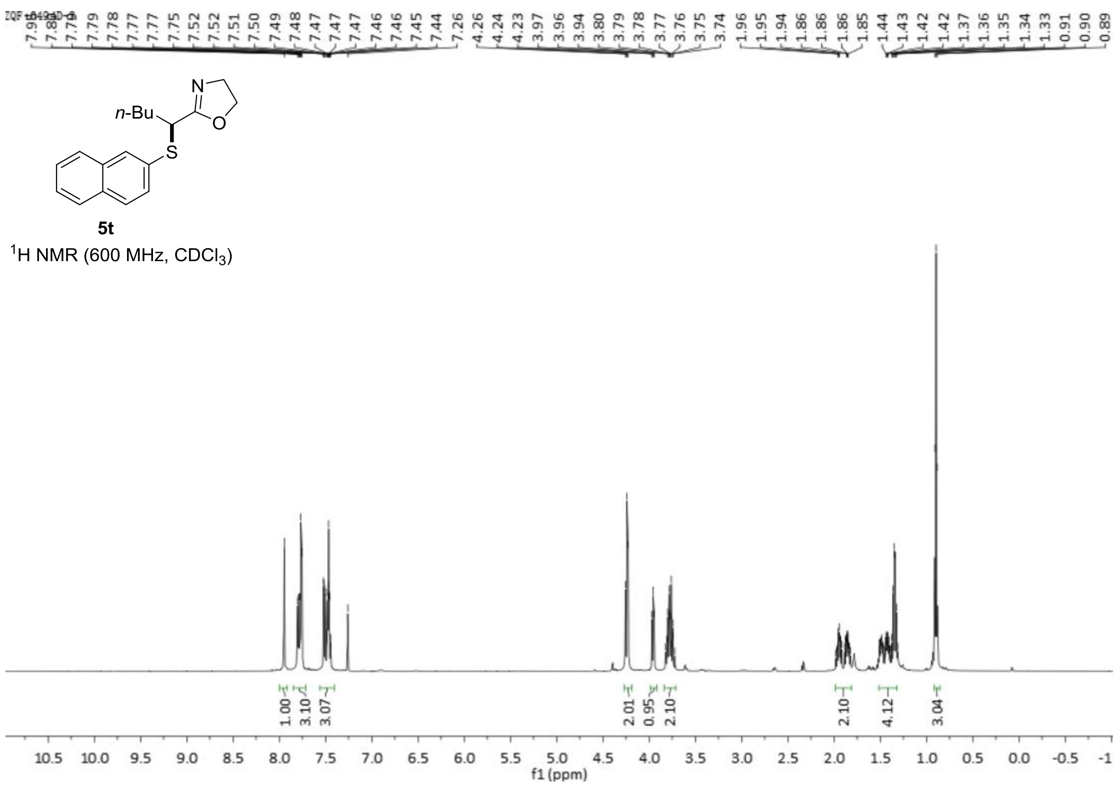


**5t**

$^{1}$H NMR (600 MHz, $CDCl_3$)

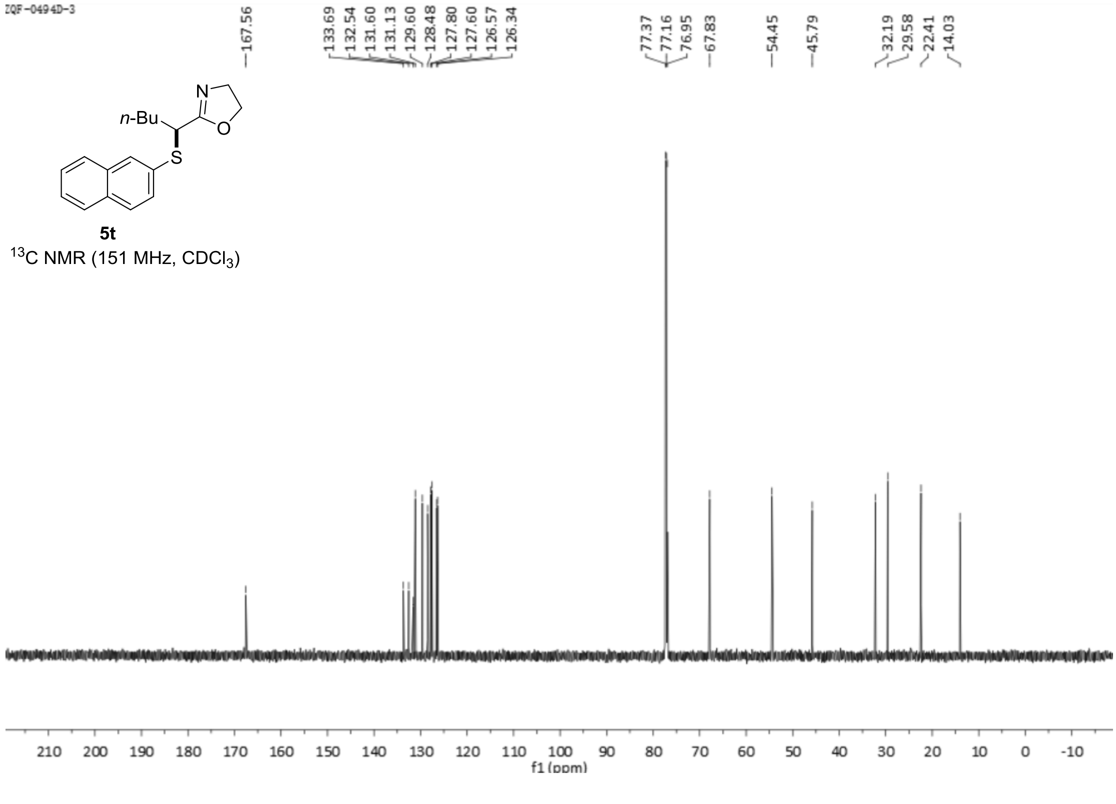


**5t**

$^{13}$C NMR (151 MHz, $CDCl_3$)

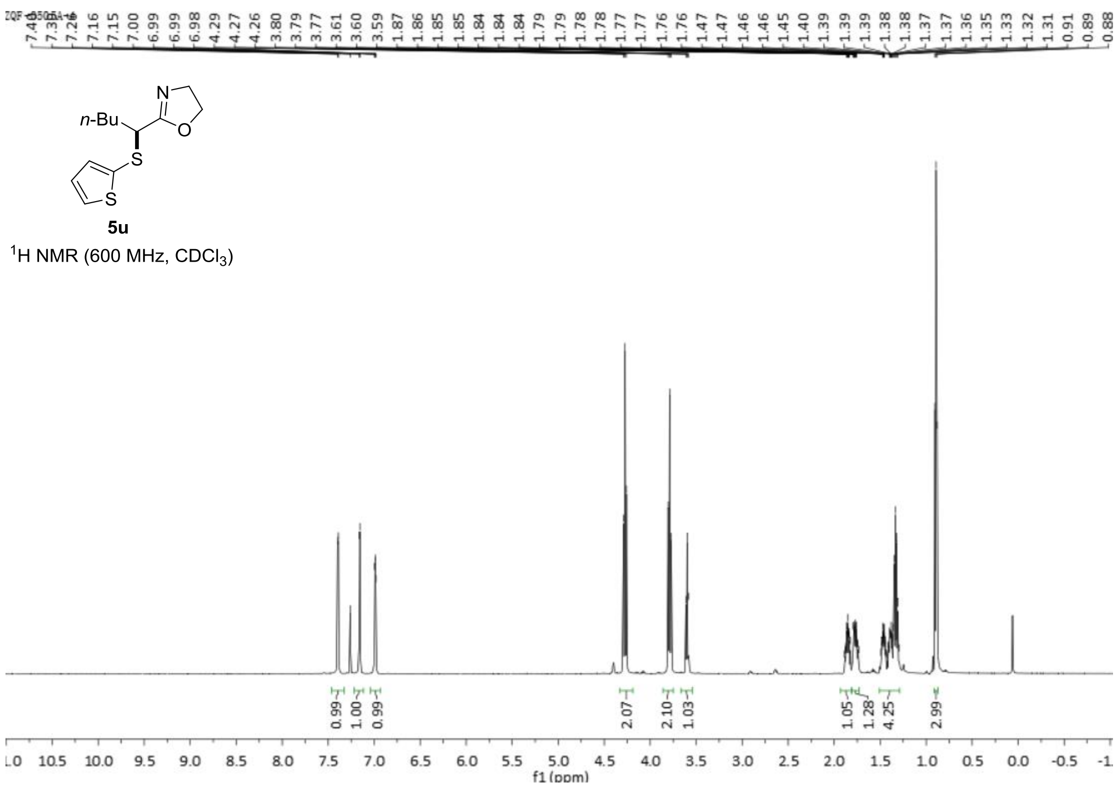
ZQF-0505A-4
7.40 7.39 7.26 7.16 7.15 7.00 6.99 6.99 6.98 4.29 4.27 4.26 3.80 3.79 3.77 3.61 3.60 3.59 1.87 1.86 1.85 1.85 1.84 1.84 1.84 1.79 1.79 1.78 1.78 1.77 1.77 1.76 1.76 1.47 1.47 1.46 1.45 1.40 1.39 1.39 1.39 1.38 1.38 1.37 1.37 1.36 1.35 1.33 1.32 1.31 0.91 0.89 0.88
n-Bu
N
O
S
S
5u
1H NMR (600 MHz, CDCl3)
0.99 1.00 0.99 2.07 2.10 1.03 1.05 1.28 4.25 2.99
f1 (ppm)

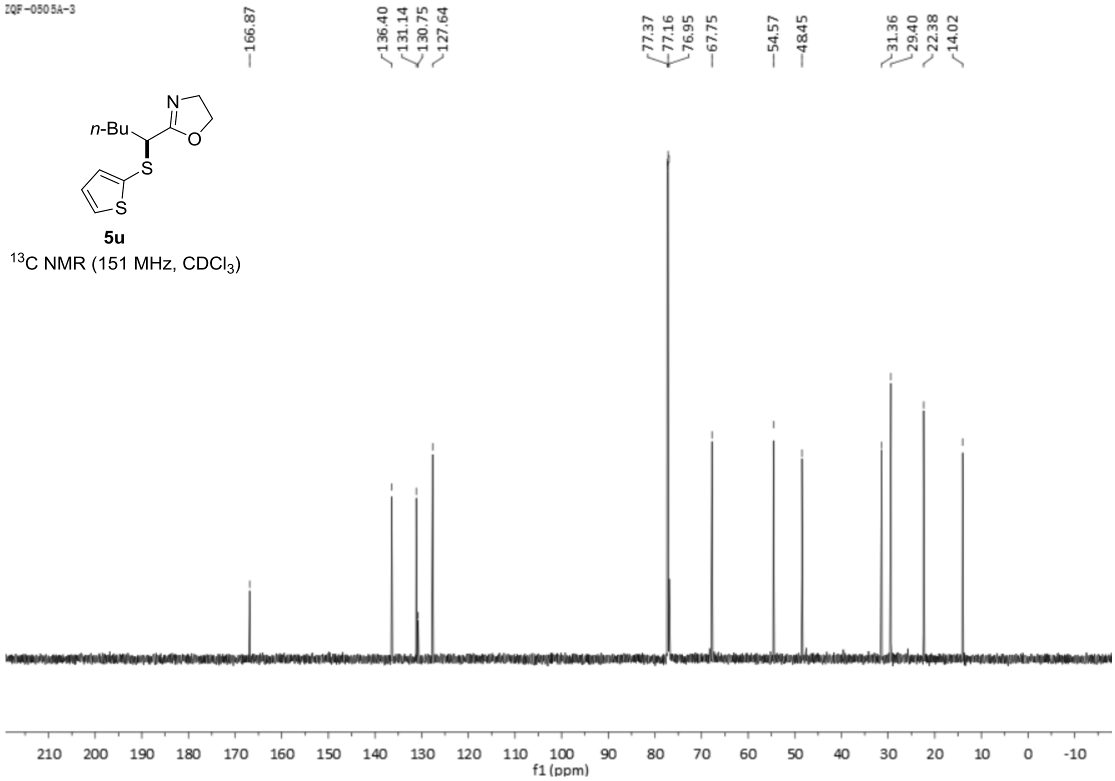
ZQF-0505A-3
166.87
136.40 131.14 130.75 127.64
77.37 77.16 76.95 67.75
54.57
48.45
31.36 29.40 22.38 14.02
n-Bu
N
O
S
S
5u
13C NMR (151 MHz, CDCl3)
f1 (ppm)

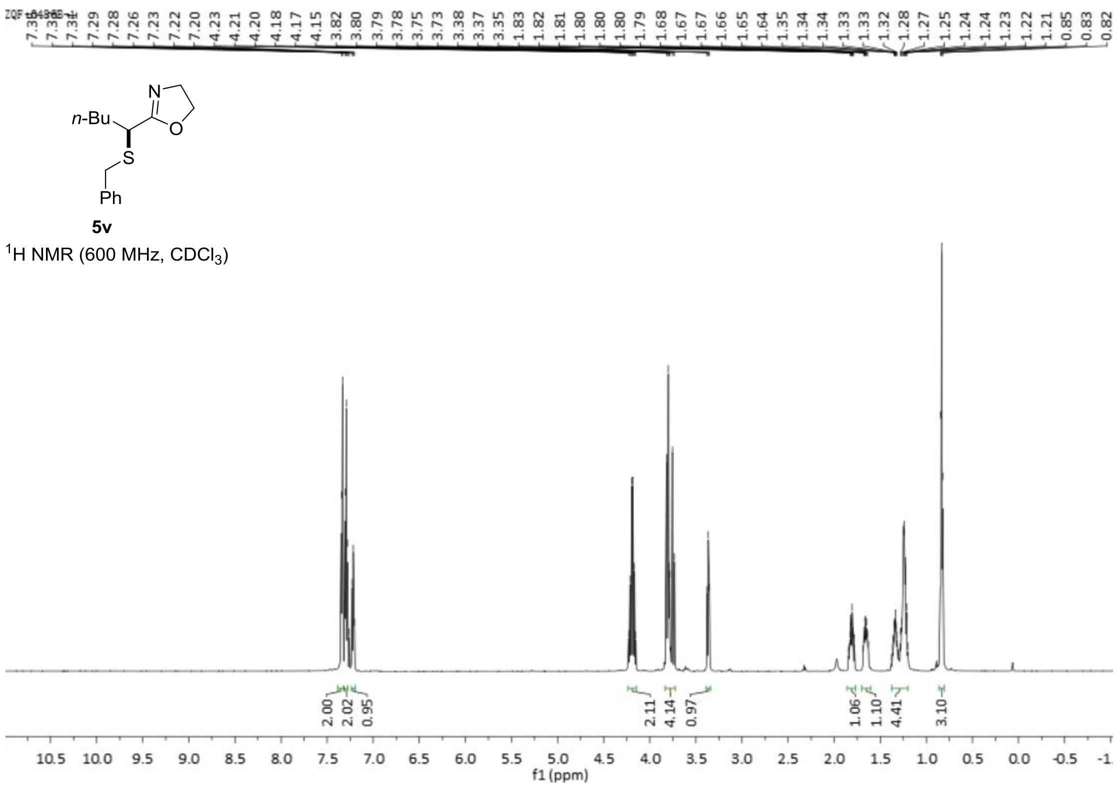


**5v**

$^1$H NMR (600 MHz, $CDCl_3$)

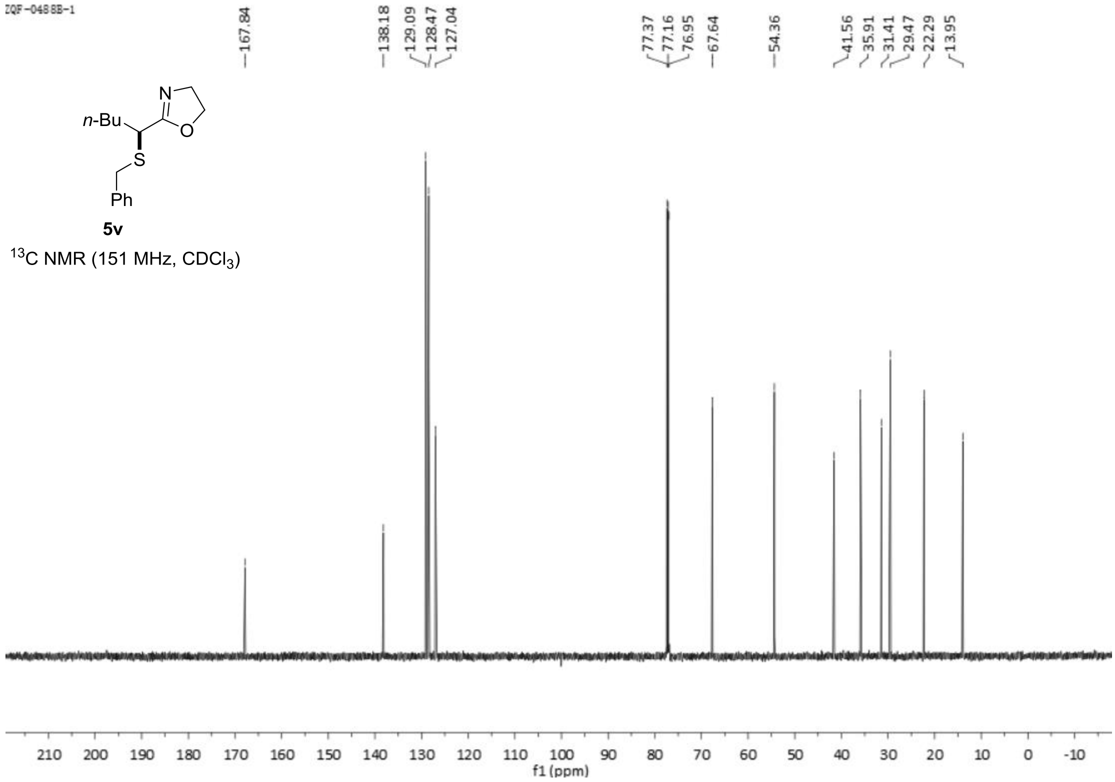


**5v**

$^{13}$C NMR (151 MHz, $CDCl_3$)

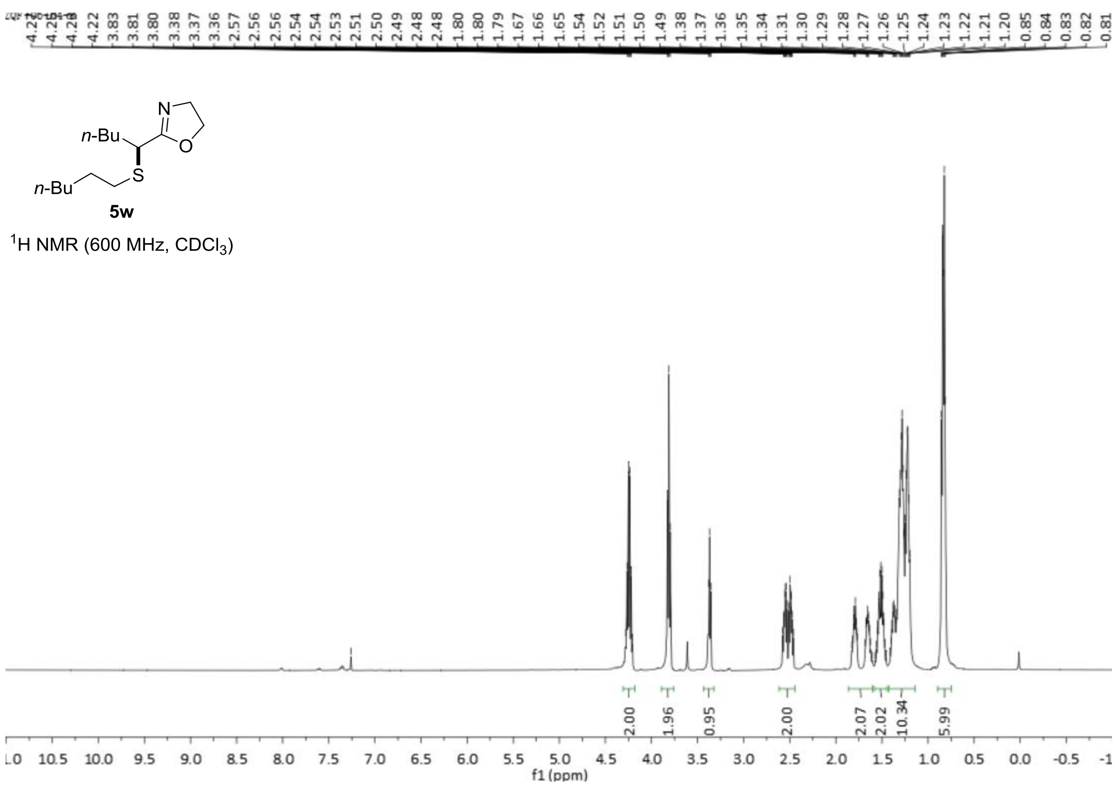
n-Bu
n-Bu
5w
$^1$H NMR (600 MHz, $CDCl_3$)
f1 (ppm)

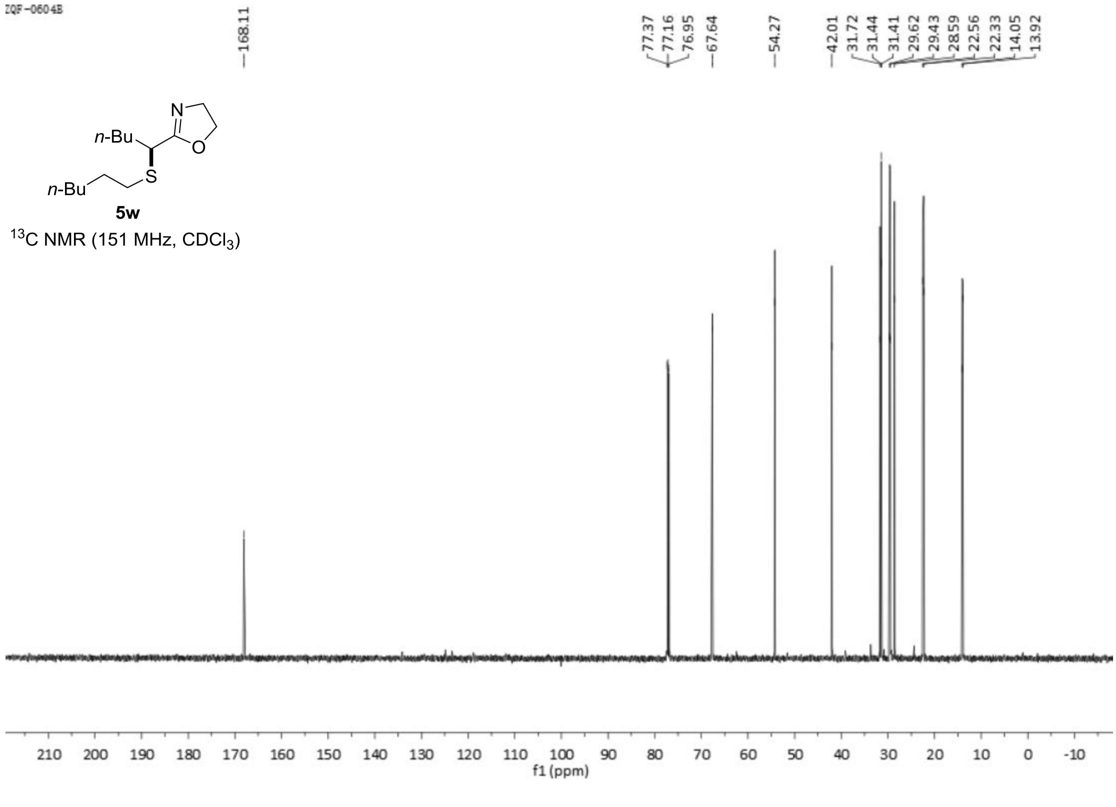
ZQF-0604B
n-Bu
n-Bu
5w
$^{13}$C NMR (151 MHz, $CDCl_3$)
f1 (ppm)

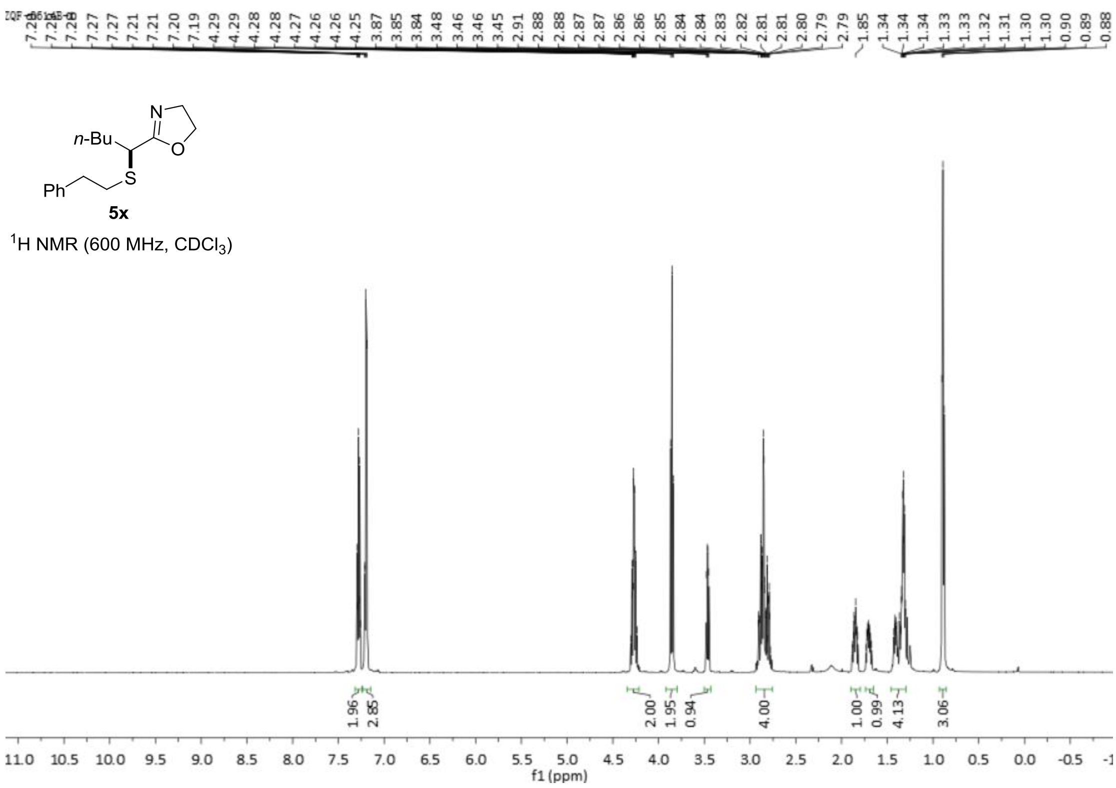

n-Bu
N
O
S
Ph
5x
1H NMR (600 MHz, CDCl3)
1.96
2.85
2.00
1.95
0.94
4.00
1.00
0.99
4.13
3.06
f1 (ppm)


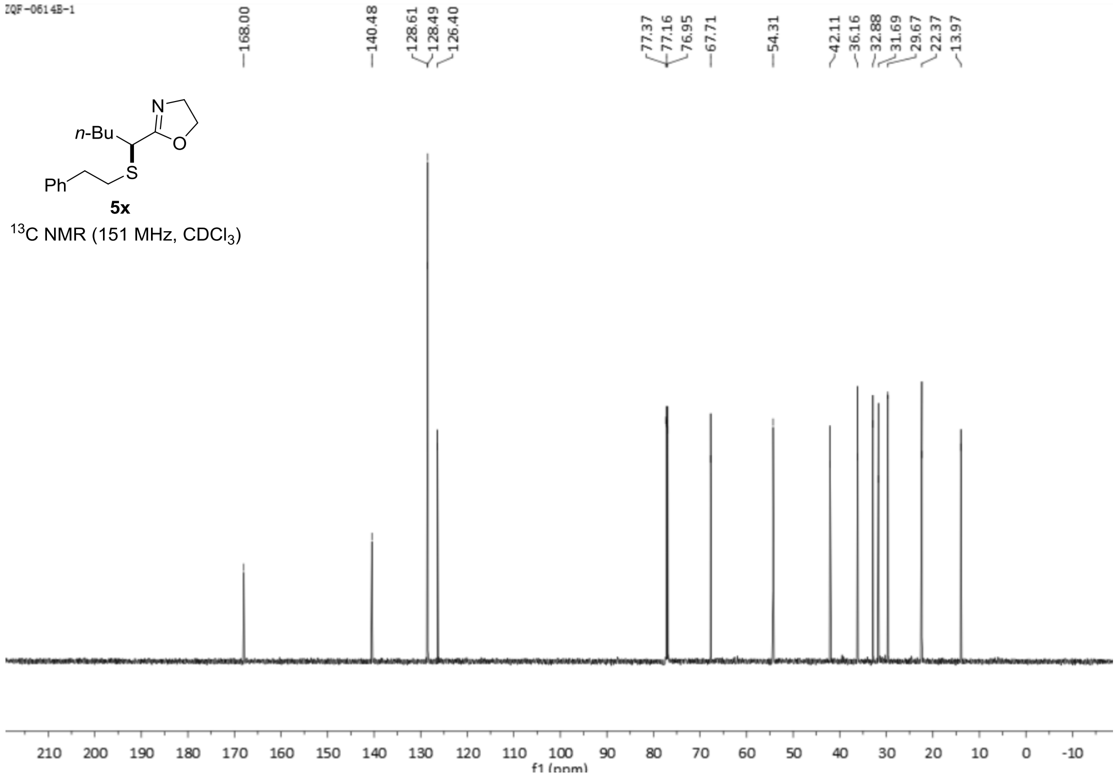

ZQF-0614B-1
168.00
140.48
128.61
128.49
126.40
77.37
77.16
76.95
67.71
54.31
42.11
36.16
32.88
31.69
29.67
22.37
13.97
n-Bu
N
O
S
Ph
5x
13C NMR (151 MHz, CDCl3)
f1 (ppm)

**5y**

$^1$H NMR (600 MHz, $CDCl_3$)

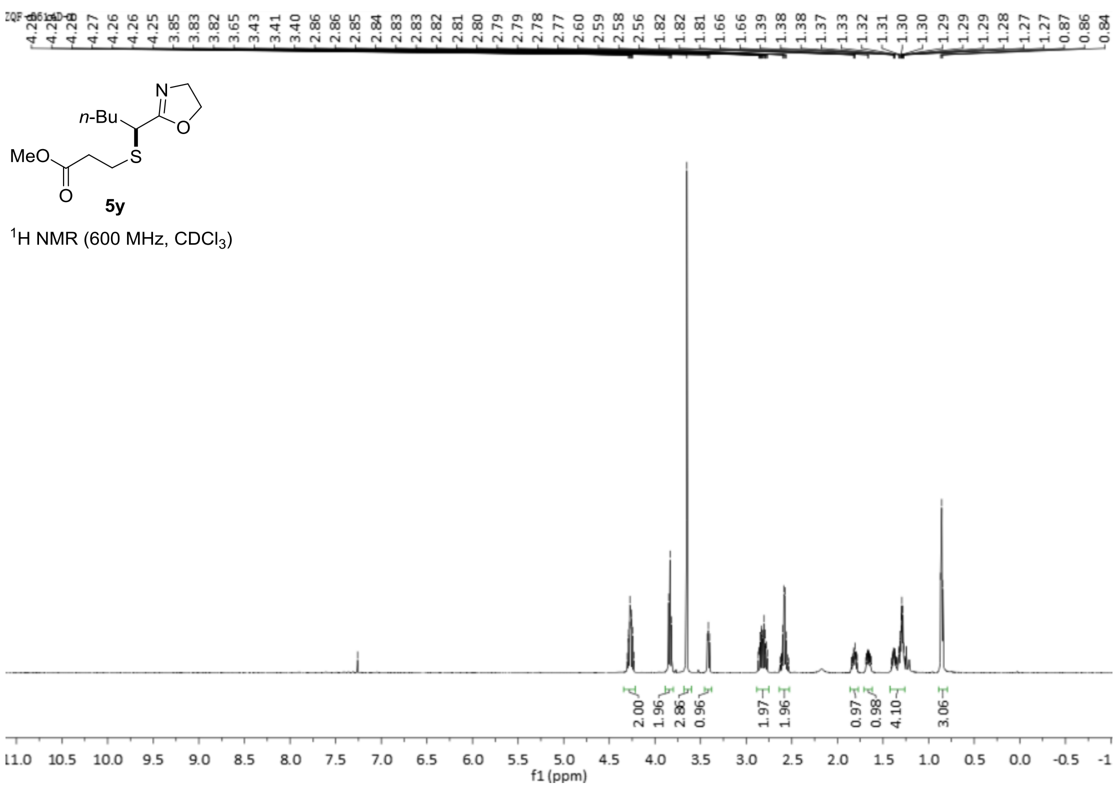


**5y**

$^{13}$C NMR (151 MHz, $CDCl_3$)

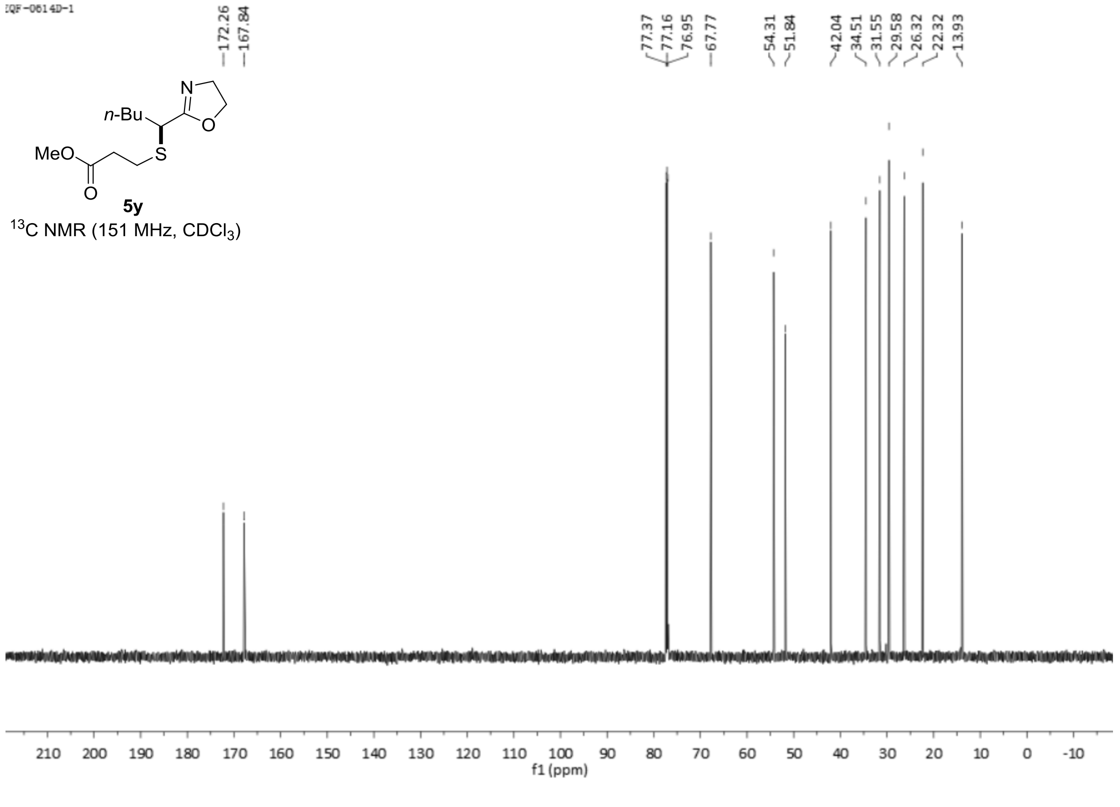

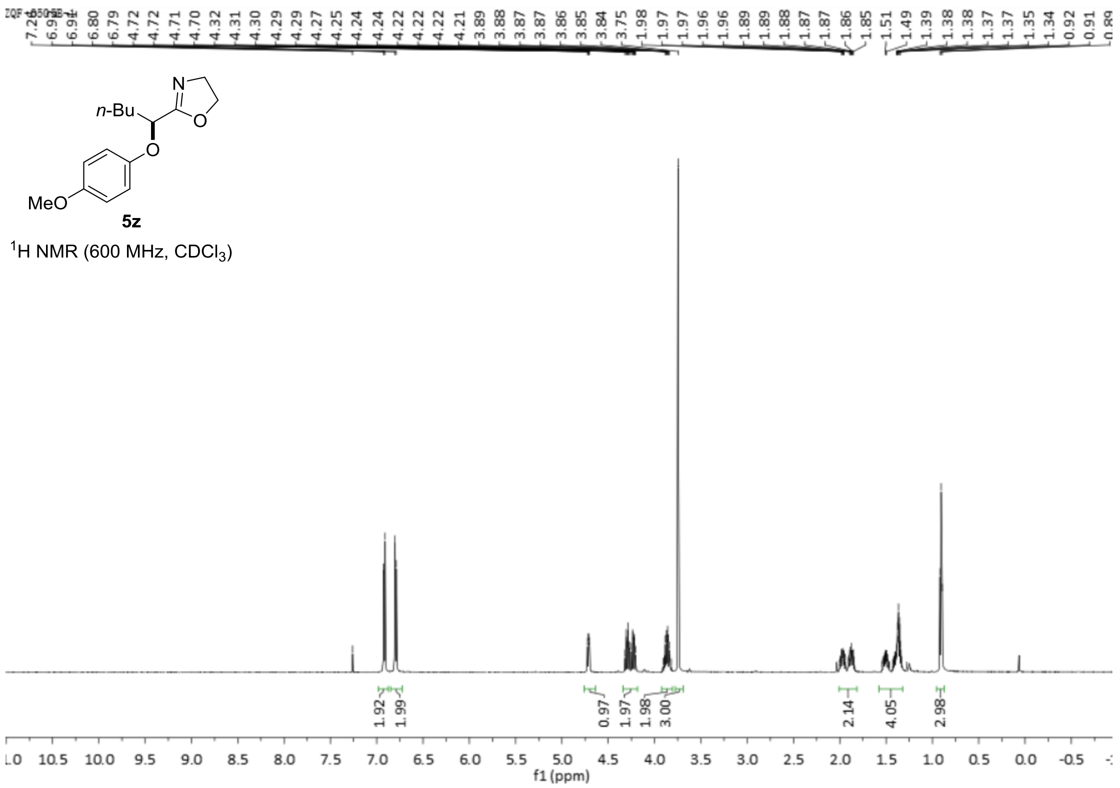


**5z**

$^{1}$H NMR (600 MHz, $CDCl_3$)

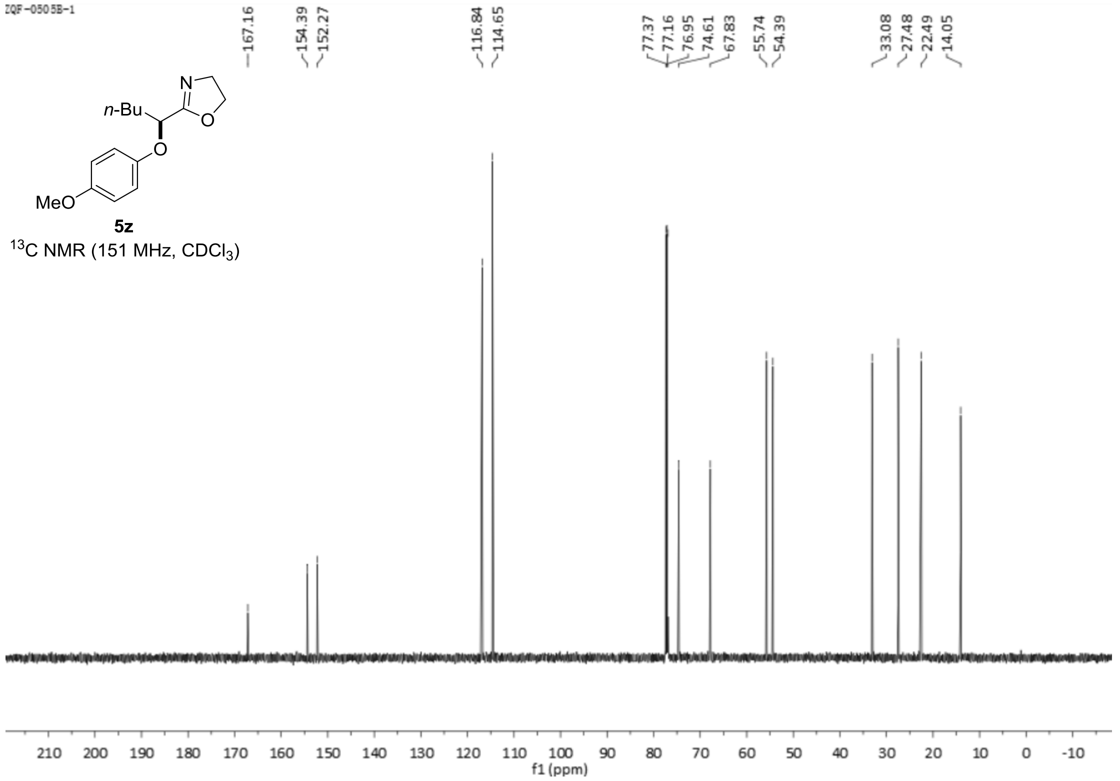


**5z**

$^{13}$C NMR (151 MHz, $CDCl_3$)

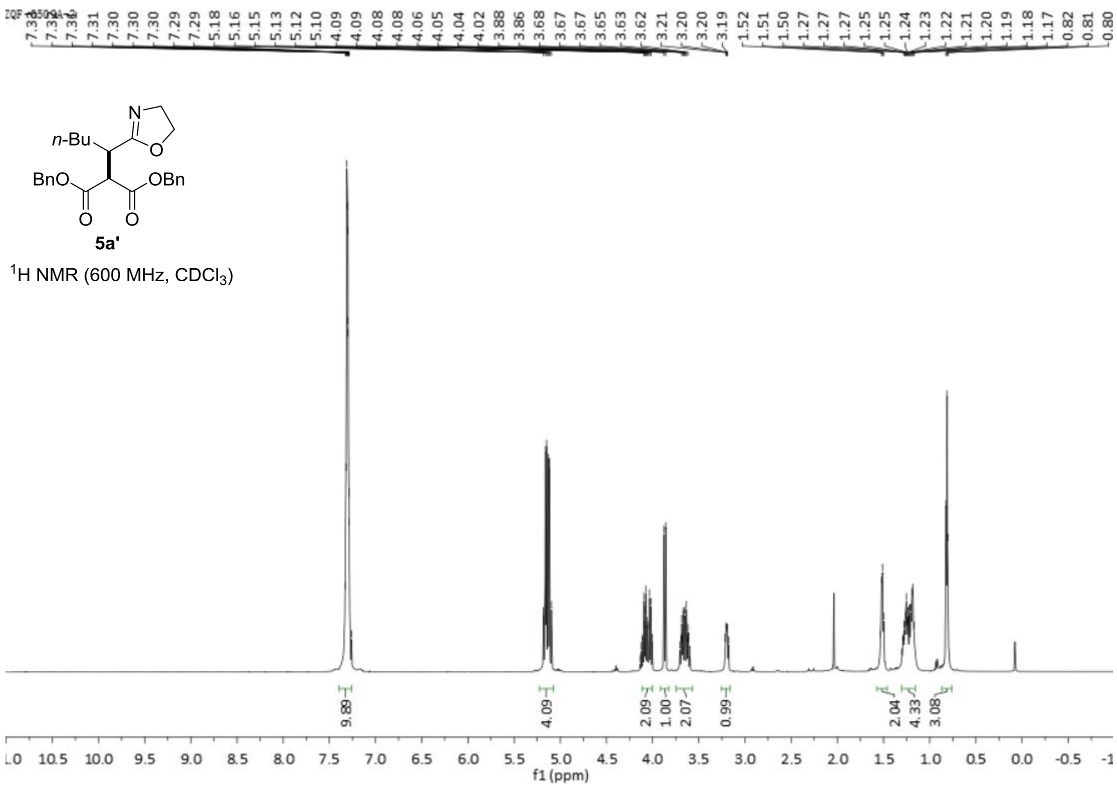

n-Bu
BnO
OBn
5a'
1H NMR (600 MHz, CDCl3)
f1 (ppm)


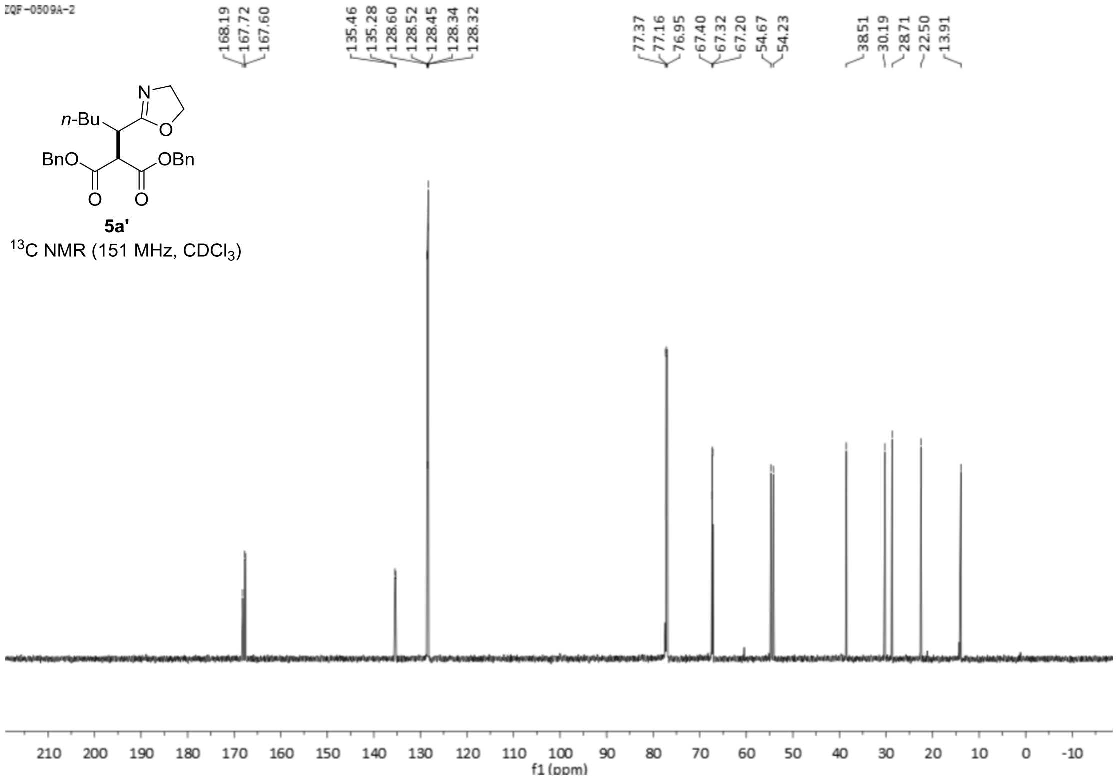

n-Bu
BnO
OBn
5a'
13C NMR (151 MHz, CDCl3)
f1 (ppm)

dp-0111-1

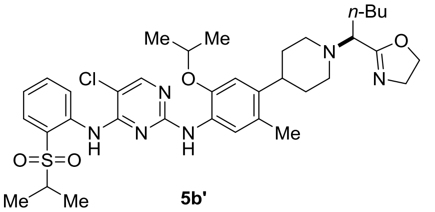


**5b'**

$^{1}H$ NMR (400 MHz, $CDCl_3$)

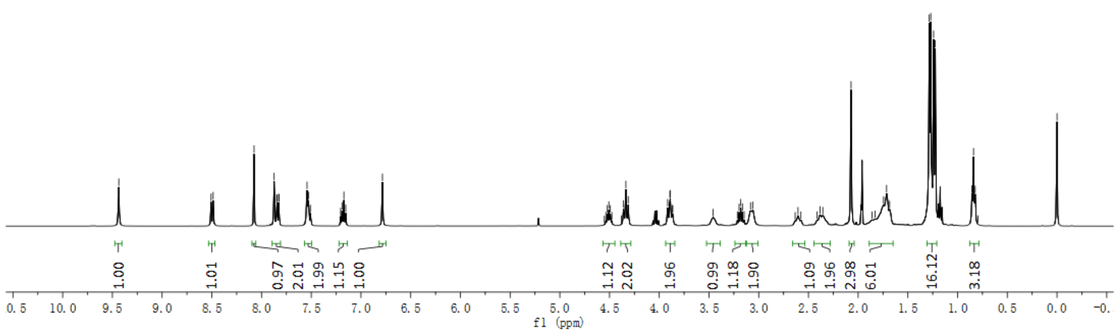


dp-0111-1

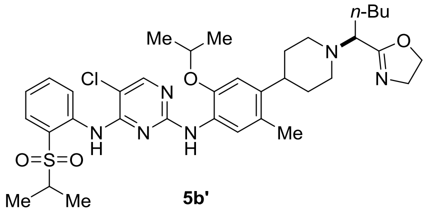


**5b'**

$^{13}C$ NMR (101 MHz, $CDCl_3$)

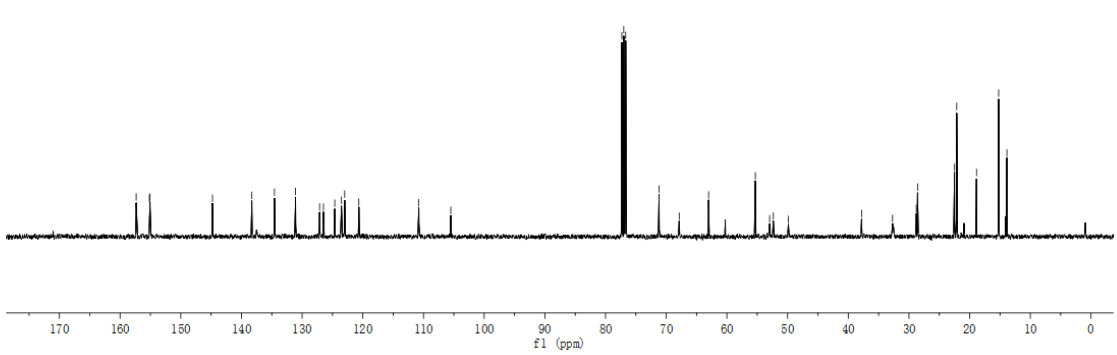

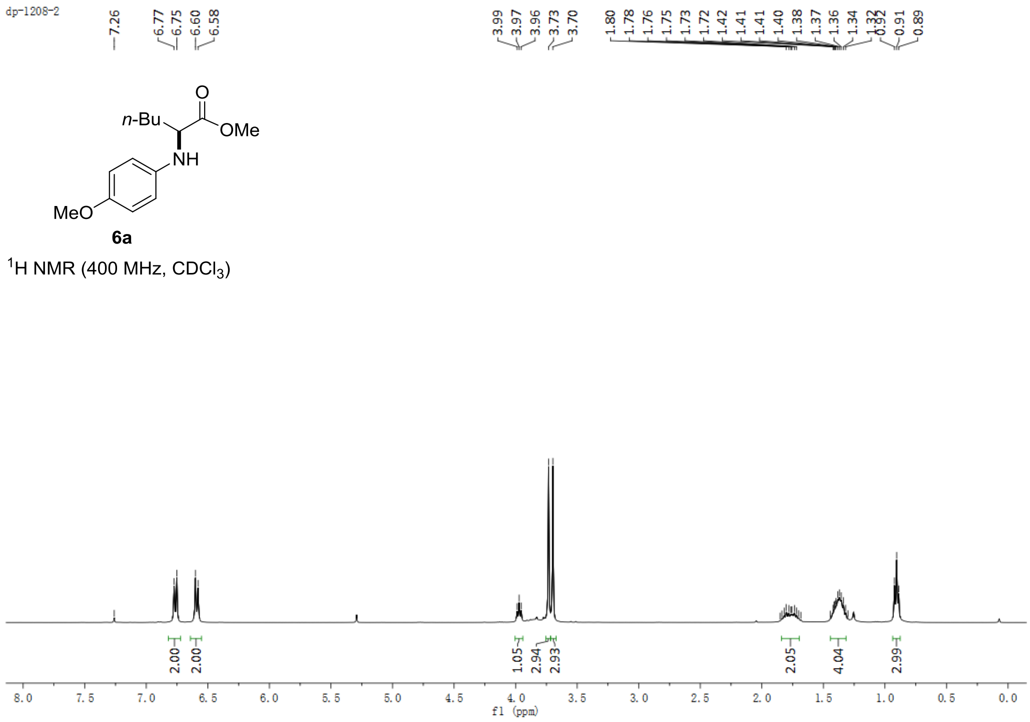


**6a**

$^1$H NMR (400 MHz, $CDCl_3$)

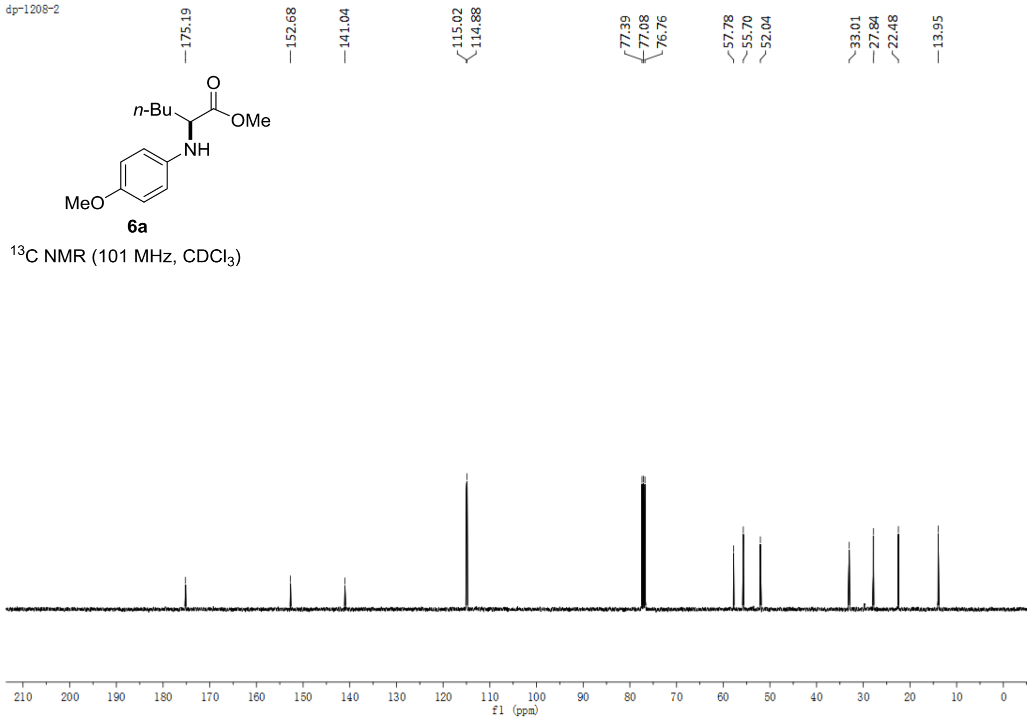


**6a**

$^{13}$C NMR (101 MHz, $CDCl_3$)

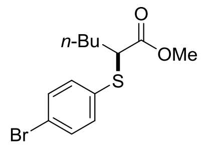

**6b**

$^{1}$H NMR (400 MHz, $CDCl_3$)

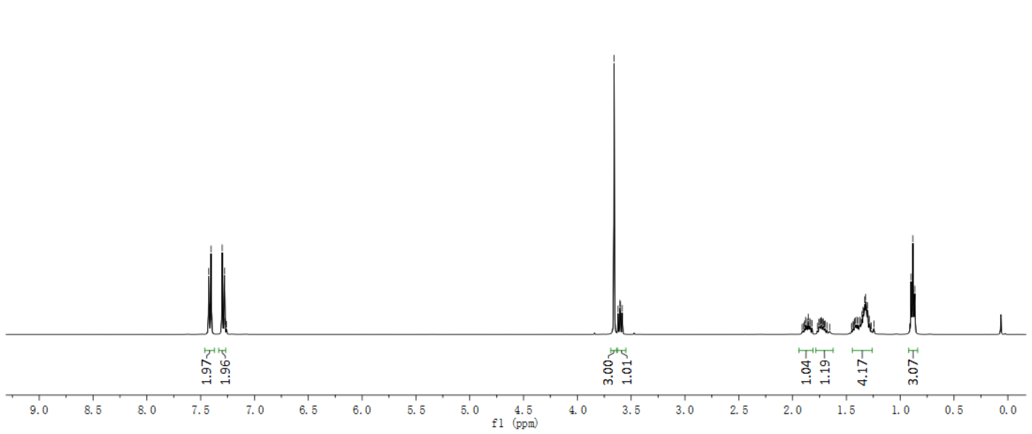


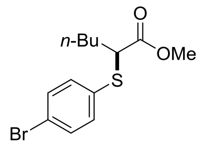

**6b**

$^{13}$C NMR (101 MHz, $CDCl_3$)

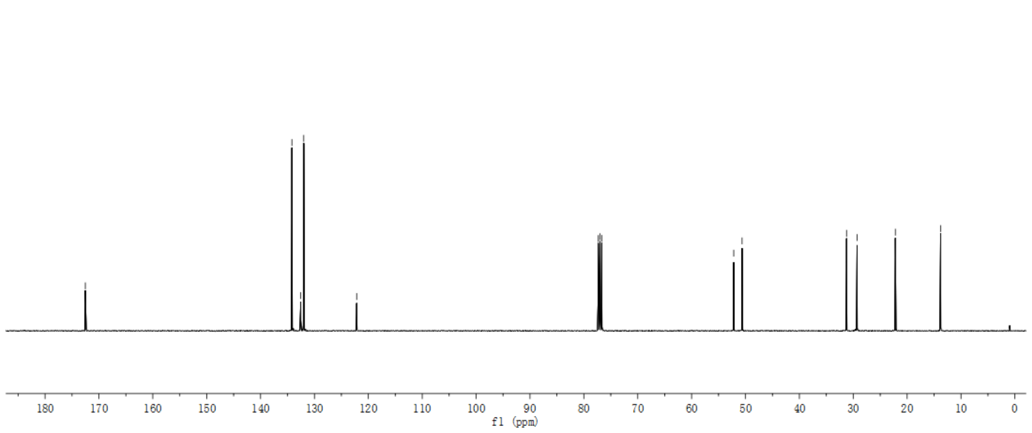

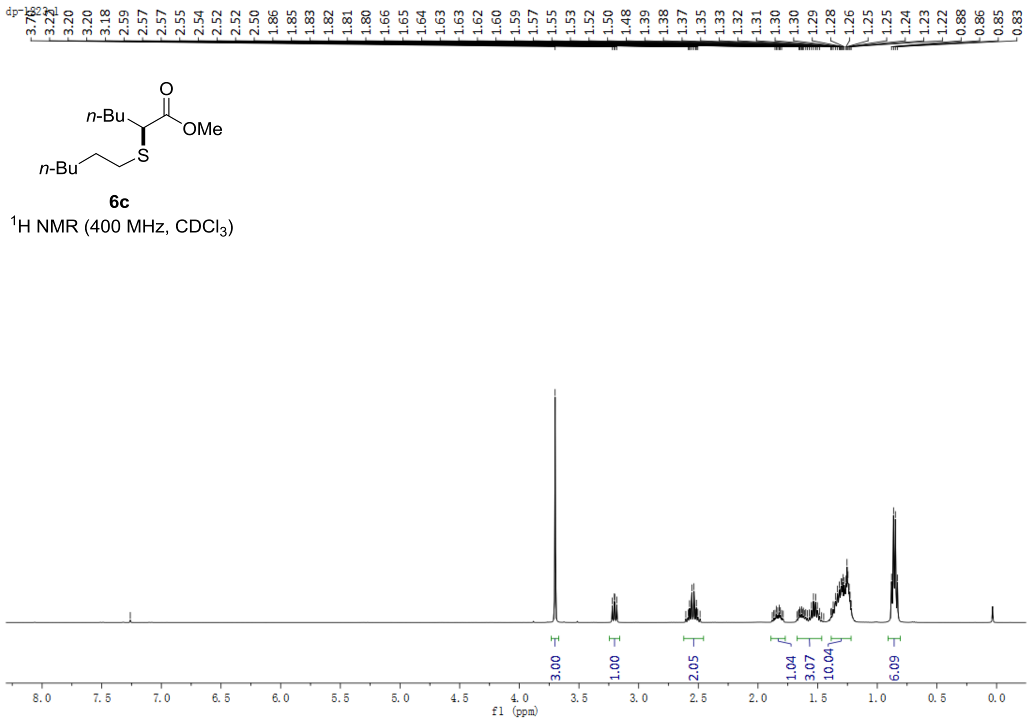


**6c**

$^1$H NMR (400 MHz, $CDCl_3$)

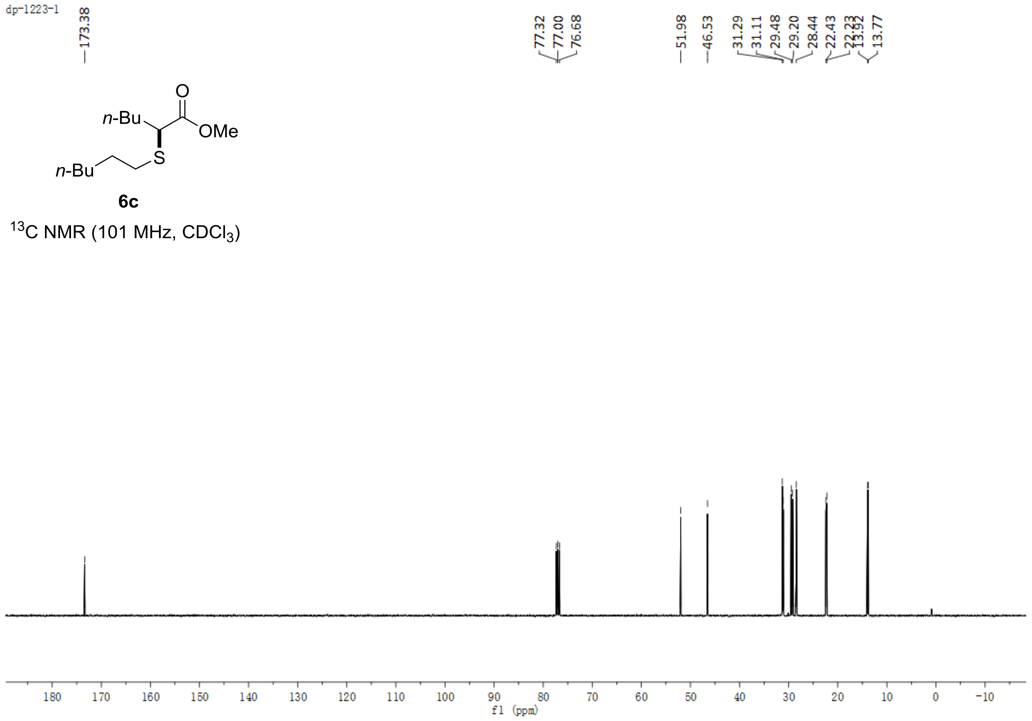


**6c**

$^{13}$C NMR (101 MHz, $CDCl_3$)

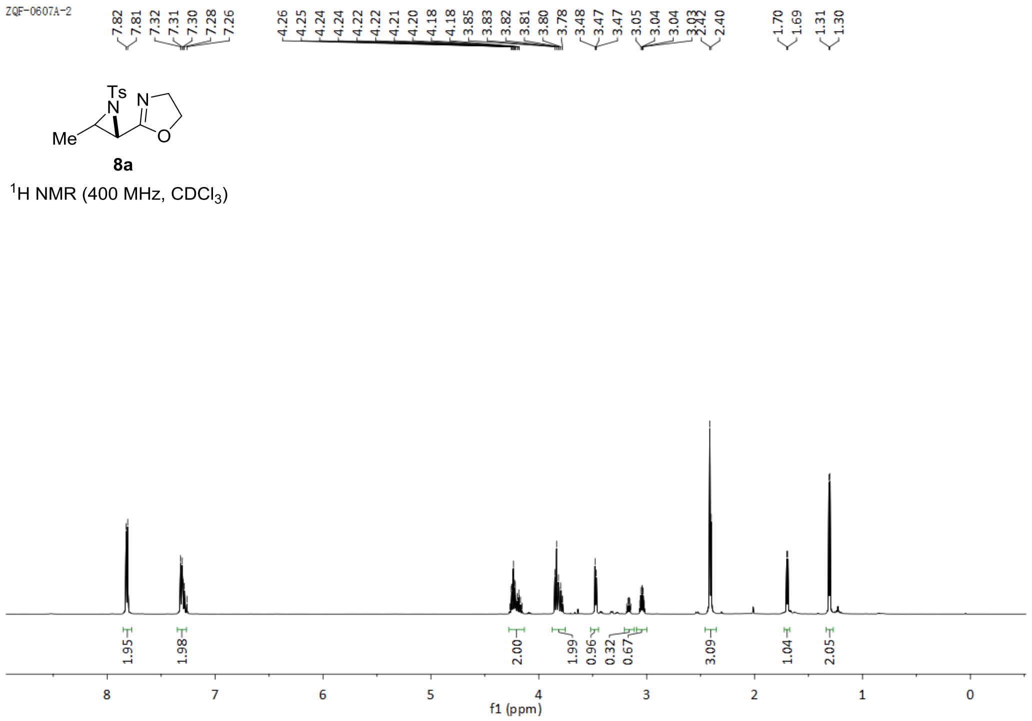


**8a**

$^1$H NMR (400 MHz, $CDCl_3$)

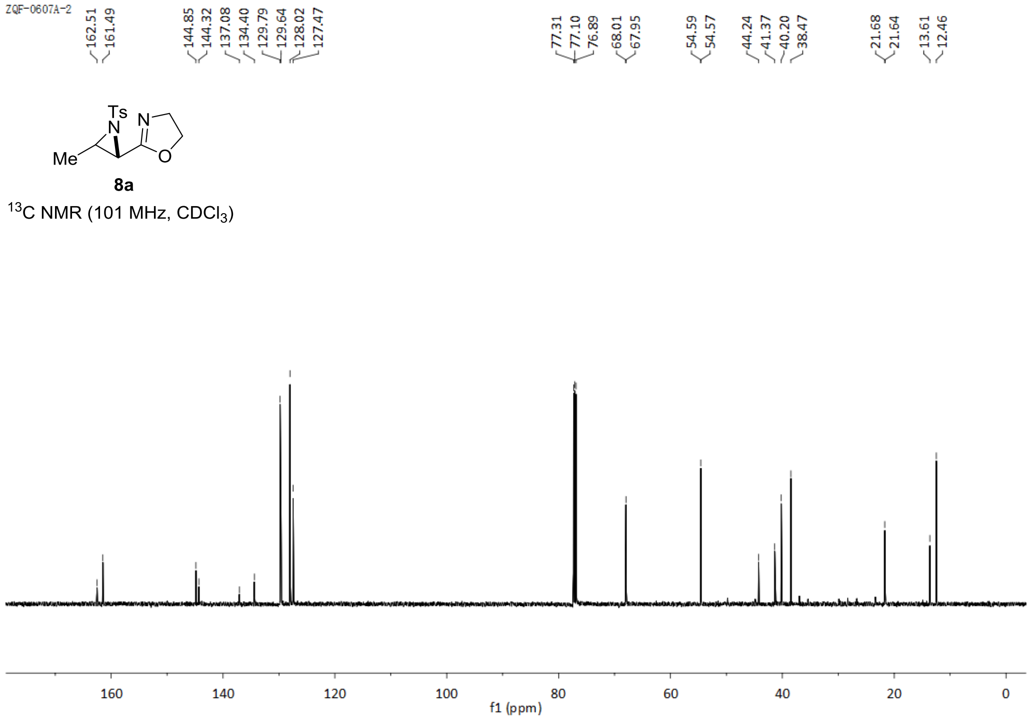


**8a**

$^{13}$C NMR (101 MHz, $CDCl_3$)

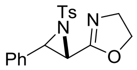

**8**b

$^{1}$H NMR (400 MHz, $CDCl_3$)

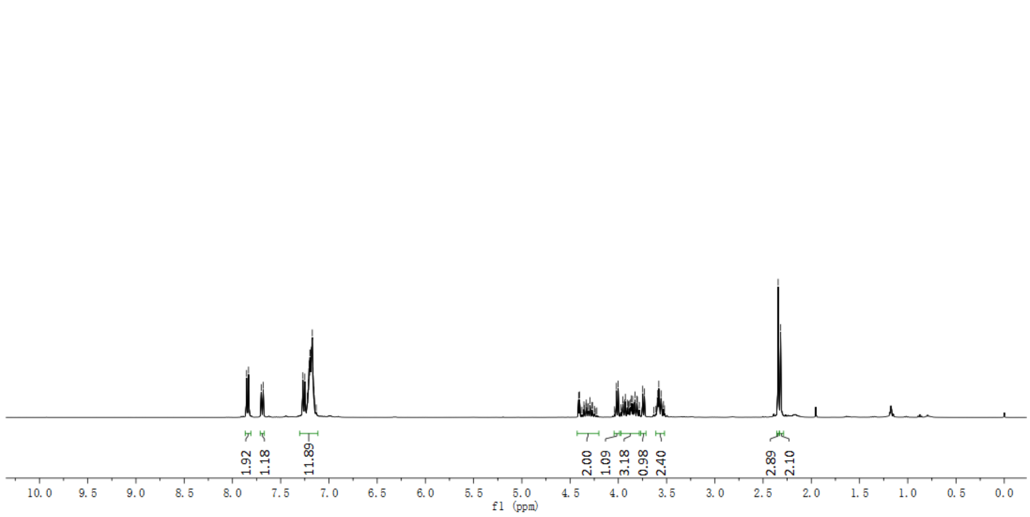


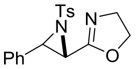

**8b**

$^{13}$C NMR (101 MHz, $CDCl_3$)

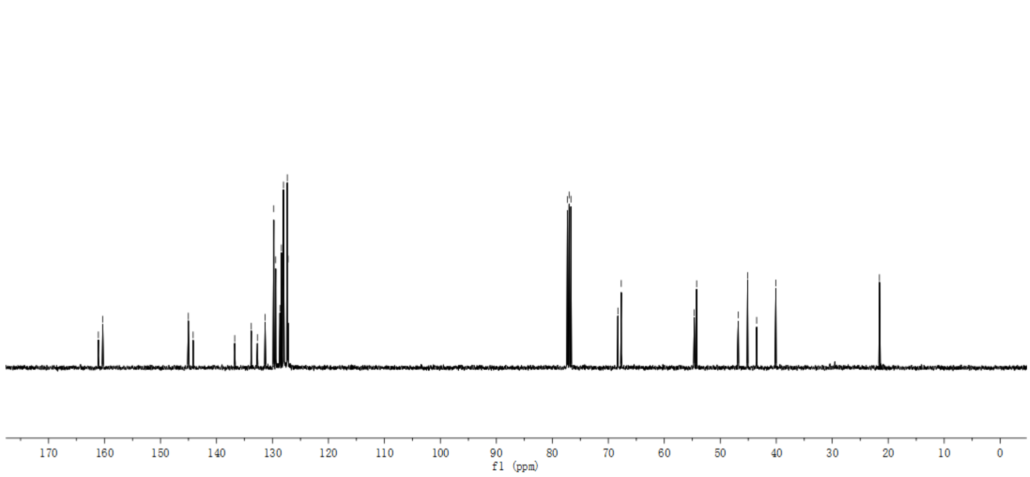

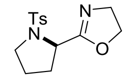

**8c**

$^{1}H$ NMR (400 MHz, $CDCl_3$)

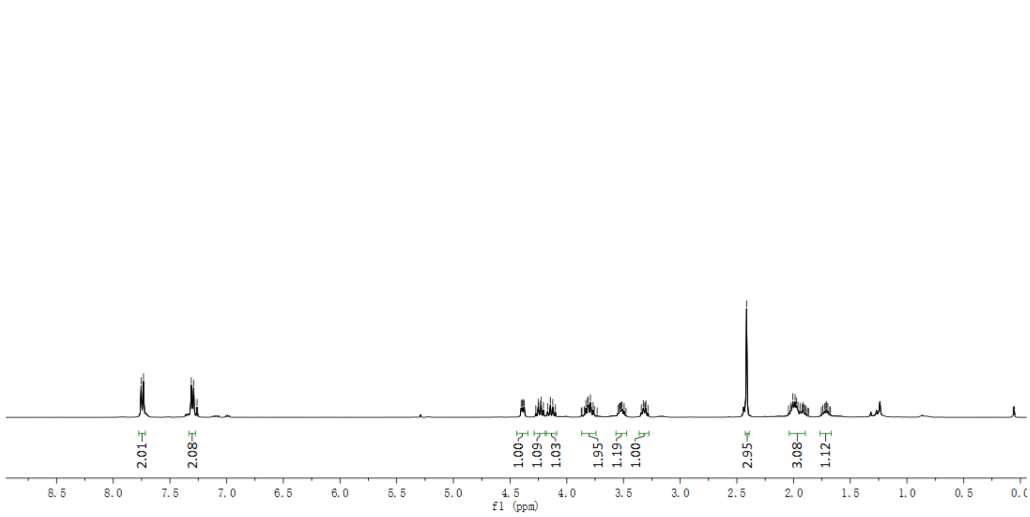


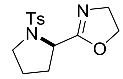

**8c**

$^{13}C$ NMR (101 MHz, $CDCl_3$)

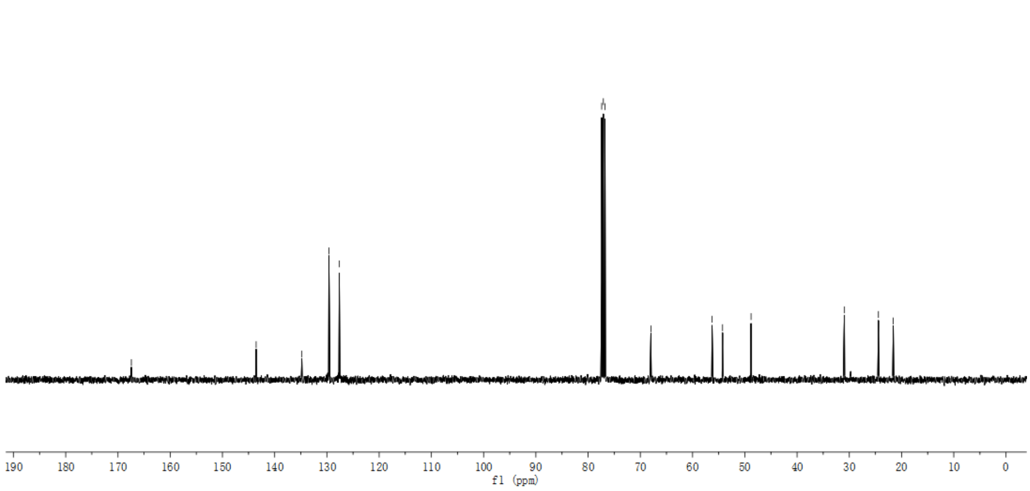

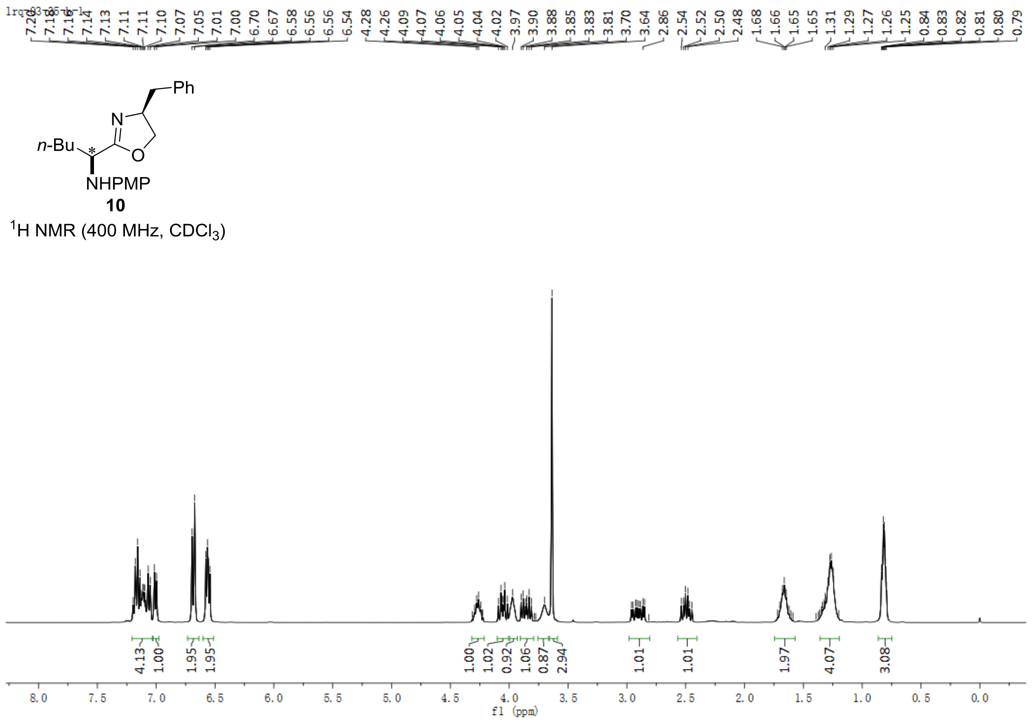


$^{1}$H NMR (400 MHz, $CDCl_3$)

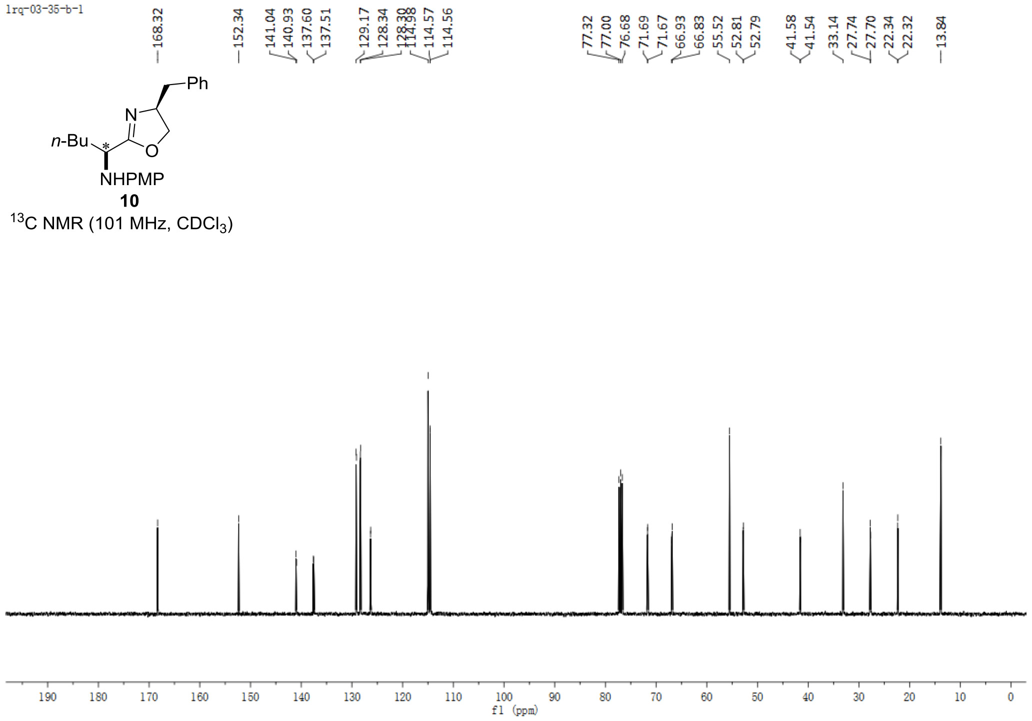


$^{13}$C NMR (101 MHz, $CDCl_3$)
